# Diastereodivergent Synthesis of Cyclopentyl Boronic Esters Bearing Contiguous Fully Substituted Stereocenters

**DOI:** 10.1002/anie.202205816

**Published:** 2022-06-23

**Authors:** Molly E. Fairchild, Adam Noble, Varinder K. Aggarwal

**Affiliations:** ^1^ School of Chemistry University of Bristol Cantock's Close Bristol BS8 1TS UK

**Keywords:** Boronic Esters, Natural Products, Ring Contraction, Stereocontrol, Stereodivergent

## Abstract

The synthesis of molecules bearing two or more contiguous, quaternary stereocenters is challenging, owing to the difficulty in controlling stereochemistry whilst simultaneously constructing a sterically congested motif. Herein, we report the electrophile‐induced ring contractive 1,2‐metallate rearrangement of 6‐membered cyclic alkenyl boronate complexes for the synthesis of cyclopentyl boronic esters bearing two contiguous, fully substituted stereocenters with high levels of stereocontrol. Remarkably, simple variation of the reaction solvent enabled their diastereodivergent construction with facile access to complementary diastereomeric pairs. The utility of our methodology is demonstrated in the asymmetric total synthesis of (+)‐herbertene‐1,14‐diol.

## Graphical abstract

An electrophile‐induced ring contractive 1,2‐metallate rearrangement of 6‐membered cyclic alkenyl boronate complexes enables the synthesis of cyclopentyl boronic esters bearing two contiguous, fully substituted stereocenters with excellent levels of enantio‐ and diastereocontrol. The utility of our methodology is demonstrated in the asymmetric total synthesis of (+)‐herbertene‐1,14‐diol.

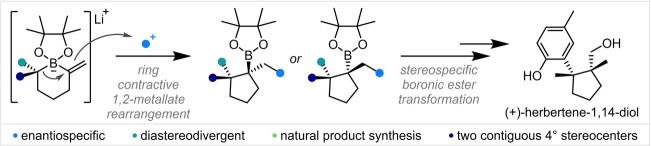

Natural products containing two or more contiguous, quaternary stereocenters at the perimeter of a cyclic core are ubiquitous in nature (Scheme 1a).^[1]^ They present a particular challenge to synthesis, not only in the construction of such a sterically congested motif but also in the control of stereochemistry.^[2]^

**Scheme 1:**
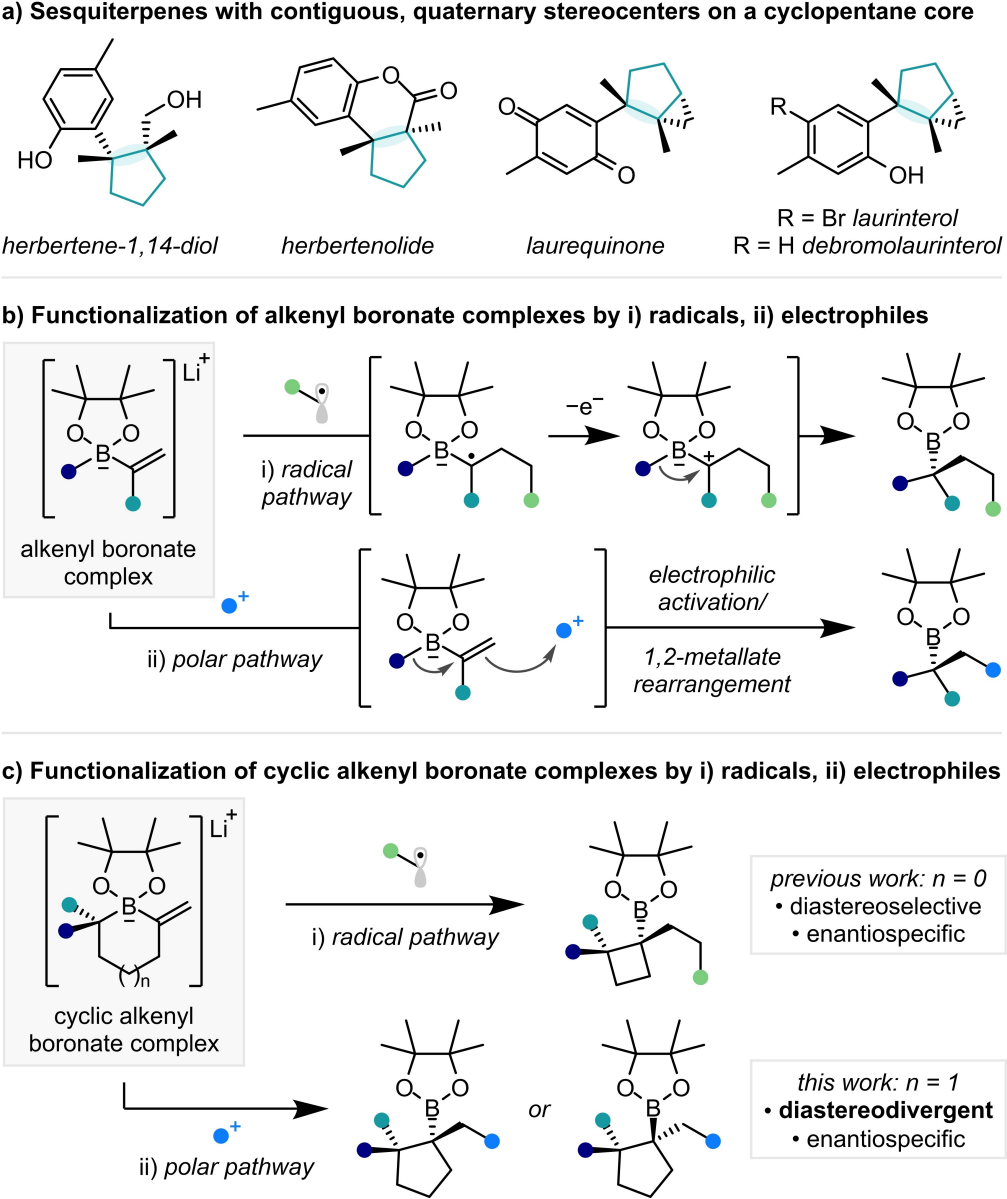
a) Natural products containing contiguous, quaternary stereocenters on a cyclopentane core. Functionalization of b) acyclic and c) cyclic alkenyl boronate complexes.

We envisaged that the stereocontrolled synthesis of densely substituted cycloalkyl boronic esters could provide rapid access to such highly‐substituted, cyclic frameworks, owing to the propensity of the C−B bond in a tertiary boronic ester to be directly and stereospecifically transformed into a C−C bond for the formation of a quaternary stereocenter.^[3]^ In particular, we noted that few methods exist for the synthesis of substituted cyclopentyl boronic esters^[4]^ and sought a generalized methodology towards α,β,β‐trisubstituted cyclopentyl boronic esters, bearing two contiguous, fully substituted stereocenters.

An attractive method for the synthesis of functionalized boronic esters harnesses the ability of alkenyl boronate complexes to undergo 1,2‐metallate rearrangements when activated by suitable electrophiles (Scheme 1b). Electrophilic, carbon‐centered radicals have proven excellent coupling partners, where oxidation of an α‐boryl radical triggers the 1,2‐metallate rearrangement in a radical/polar crossover pathway.^[5]^ A polar pathway, where activation of the same alkenyl boronate complex with an electrophilic species occurs with 1,2‐metallate rearrangement, allows the synthesis of complementary products.[^6^, ^7^, ^8^]

1,2‐Metallate rearrangements of cyclic boronate complexes have rarely been explored yet could provide expedient access to highly functionalized cycloalkyl boronic esters (Scheme 1c).^[9]^ We recently disclosed a method by which cyclobutyl boronic esters could be accessed with high diastereoselectivity and complete enantiofidelity from the addition of electrophilic radicals to cyclic alkenyl boronate complexes.^[10]^ We sought to further explore the utility of this ring contractive 1,2‐metallate rearrangement strategy through a polar pathway with a broad set of electrophiles. We now report that this strategy not only provides access to sterically congested cyclopentyl boronic esters in a stereocontrolled manner, but either diastereomer of the product can be obtained selectively from the same starting material in solvent‐controlled diastereodivergent reactions.

Our investigations began by formation of 6‐membered cyclic alkenyl boronate complex **2 a** (Table 1). Reaction monitoring by ^11^B NMR spectroscopy confirmed that **2 a** could be accessed quantitatively upon addition of *tert‐*butyllithium to boronic ester **1 a** to enact a lithium–halogen exchange and cyclization sequence. Pleasingly, addition of Eschenmoser's salt **4** to boronate complex **2 a** gave a high yield of boronic ester **3 a**, which possesses two contiguous, fully substituted stereocenters (entry 1). High levels of diastereocontrol were observed in this sterically demanding transformation (89 : 11 in favor of diastereomer **d^1^**) and the reaction proceeded with 100 % enantiospecificity.

**Table 1:** Optimization of the ring contractive 1,2‐metallate rearrangement.

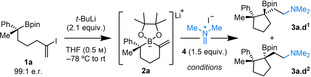				
	Conditions^[a]^	Yield^[b]^ [%]	**d^1^**/**d^2^** ^[c]^	e.r.^[d]^
1	THF (0.5 m), −78 °C to rt	70 (73)^[e]^	89 : 11	99 : 1
2	1 : 1 THF/MeCN (0.25 m), −40 °C to rt	78	81 : 19	–
3	1 : 1 THF/DMF (0.25 m), −40 °C to rt	56	86 : 14	–
4	1 : 1 THF/MeOH (0.25 m), −78 °C to rt	64	28 : 72	–
5	1 : 1 THF/HFIP (0.25 m), −40 °C to rt	83	10 : 90	–
6	1 : 1 THF/TFE (0.25 m), −78 °C to rt	96 (99)^[e]^	14 : 86	99 : 1

All reactions carried out using **1 a** (0.1 mmol). [a] Reactions stirred for 2 h at the given temperature, followed by slow warming to rt overnight. [b] Yields determined by ^1^H NMR analysis using CH_2_Br_2_ as an internal standard. [c] Diastereomeric ratio (d.r., **d^1^**/**d^2^**) determined by ^1^H NMR analysis. [d] Enantiomeric ratio (e.r.) determined by ^1^H NMR analysis using Pirkle's chiral solvating agent. [e] 0.2 mmol scale. Isolated as the hydrochloride salt.

During the reaction, we noted that **4** was sparingly soluble in THF at low temperature and dissolution only occurred upon warming to room temperature. Therefore, we screened different solvent additives to improve the solubility of **4**, with the expectation that this would allow the reaction to proceed at lower temperature and improve the diastereoselectivity (entries 2–6; see the Supporting Information for complete optimization results). Interestingly, the addition of protic solvents, including methanol, 1,1,1,3,3,3‐hexafluoro‐2‐propanol (HFIP) or 2,2,2‐trifluoroethanol (TFE), led to an inversion of the diastereoselectivity of the reaction. The effect was most pronounced with fluorinated alcohols, with TFE yielding boronic ester **3 a** in a near quantitative yield with diastereomer **d^2^** favored over **d^1^** in an 86 : 14 ratio and with 100 % enantiospecificity (entry 6). This diastereodivergence is remarkable; the substrate bias for the formation of one diastereomer was overturned simply by the addition of a co‐solvent. Such asymmetric diastereodivergent reactions are attractive,^[11]^ as they allow access to all possible stereoisomeric products. Although the origin for the reversal in diastereoselectivity in the formation of **3 a** is unclear, previous reports have postulated that hydrogen bonding interactions between an alcohol and the Lewis basic pinacol oxygens in the boronate complex modulate the nucleophilicity of the C−B bonds, altering the electronics of the boronate complex.[^7a^, ^12^] Additionally, such complexation could change the steric environment around the reacting boronate complex.

Having established two sets of electrophile addition conditions that gave diastereodivergent outcomes, we investigated the scope of the reaction with respect to the boronate complex to generate a range of γ‐amino boronic esters (Scheme 2). We were delighted to find that our methodology enabled the efficient and enantiospecific synthesis of α,β,β‐trisubstituted cyclopentyl boronic esters **3 a**, **3 b** and **3 c** using tertiary benzylic boronic ester starting materials bearing various aryl substitution, which were accessible in high enantiopurity through lithiation–borylation methodology.^[13]^ Gratifyingly, in all cases solvent‐induced diastereodivergency was observed; the addition of a TFE co‐solvent was the only modification to the protocol necessary to allow access to the complementary diastereomeric partner with high diastereoselectivity. Interestingly, this methodology also provided access to a structurally complex spirocyclic cyclopentyl boronic ester **3 d** as a single diastereomer, installing a challenging, all‐carbon quaternary spirocenter.^[14]^ Secondary boronic esters bearing α‐methyl and α‐isopropyl substitution proved competent substrates, affording α,β‐disubstituted boronic esters **3 e** and **3 f** in excellent yields and as essentially single diastereomers. Unfortunately, solvent‐induced diastereodivergency was not observed in the formation of **3 d**, **3 e** and **3 f**, with conditions A no longer leading to the reversed diastereochemical outcome but instead providing similar results to conditions B. The parent boronic ester, with no α‐carbon substituents, also underwent the ring contractive 1,2‐metallate rearrangement to give **3 g**.

**Scheme 2:**
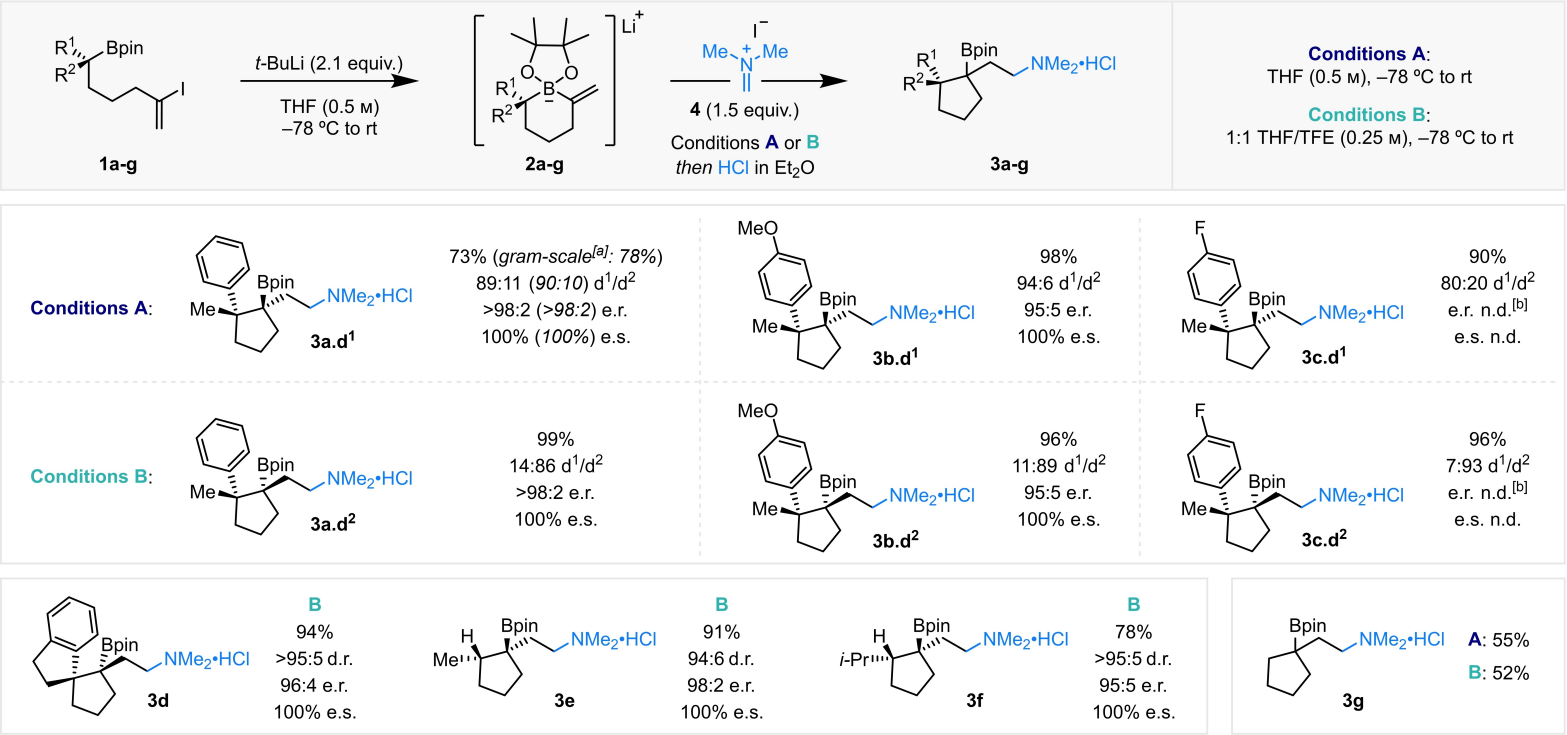
Cyclic alkenyl boronate complex scope for the ring contractive 1,2‐metallate rearrangement with Eschenmoser's salt **4**. All reactions carried out using **1** (0.2 mmol). Yields are of isolated products; d.r. determined by ^1^H NMR analysis; e.r. determined by ^1^H NMR analysis using Pirkle's chiral solvating agent. [a] Using **1 a** (2.35 mmol). [b] The e.r. could not be determined by chiral HPLC, chiral SFC or using a chiral solvating agent.

We then proceeded to explore the reactivity of a diverse range of electrophiles with boronate complex **2 a** to yield products with two contiguous, fully substituted stereocenters and forge valuable C−C or C−heteroatom bonds (Scheme 3). Iminium salts proved very efficient electrophiles in this reaction, introducing an amine functionality into boronic ester products **3 a** and **3 h**. In each case, the diastereoselectivity was high and the diastereochemical bias was reversed with TFE as a co‐solvent. Other cationic, carbon‐based electrophiles were then investigated, including tropylium, which successfully introduced the cycloheptatriene functionality into boronic ester **3 i**. Interestingly, under conditions A, two products derived from the reaction of boronate complex **2 a** with the electrophile were observed. The desired product **3 i** was generated in 44 % yield and 94 : 6 d.r., alongside 21 % of side product **5**, which originates from reaction of the electrophile with the nucleophilic C(sp^3^)−B bond.^[15]^ Under conditions B, where the nucleophilicity of the C(sp^3^)−B bond is modulated by the presence of hydrogen bond donor TFE, side‐product **5** was not observed and instead an increased yield of **3 i** was seen, favoring the opposing diastereomer. The use of 1,3‐benzodithiolylium as the electrophile incorporated the benzodithiole functional group into boronic ester **3 j**, albeit in lower yield. Unfortunately, under conditions B the d.r. of **3 j** was altered but not reversed.

**Scheme 3:**
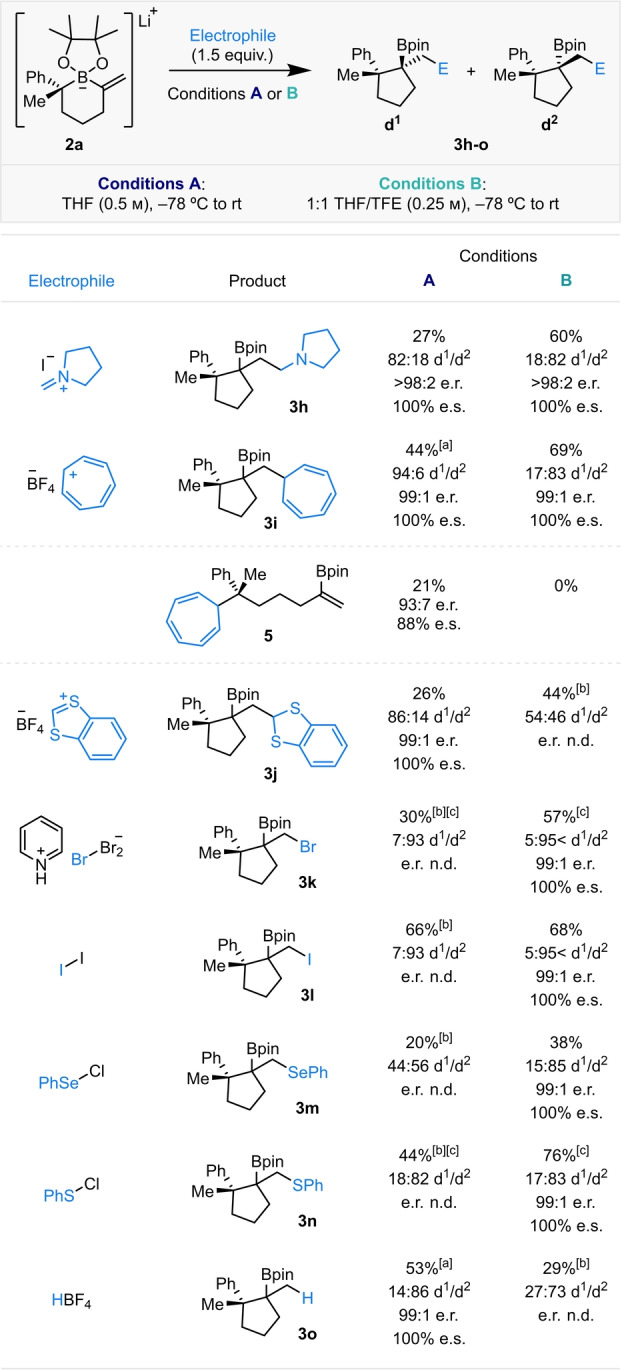
Electrophile scope for the ring contractive 1,2‐metallate rearrangement of boronate complex **2 a**, derived from alkenyl iodide **1 a** (0.2 mmol, 99 : 1 e.r.). Yields are of isolated products; d.r. determined by ^1^H NMR analysis; e.r. determined by either chiral HPLC/SFC analysis or ^1^H NMR analysis using Pirkle's chiral solvating agent. [a] Modified conditions A: 1 : 1 THF/MeCN (0.25 m). [b] Using racemic **1 a** (0.1 mmol). [c] Boronate complex **2 a** derived from alkenyl bromide **1 h** (0.2 mmol, 99 : 1 e.r.) (see the Supporting Information for more details).

New C−heteroatom bonds could also be constructed in this reaction sequence. Under conditions B, β‐bromo (**3 k**) and β‐iodo (**3 l**) boronic esters were formed as single diastereomers in good yields, with negligible competing Zweifel‐type elimination.^[6]^ Our methodology also allowed the diastereoselective formation of β‐selenyl (**3 m**) and β‐sulfenyl (**3 n**) boronic esters in good yields. In all cases, conditions B always proved superior in terms of yield and diastereoselectivity. No diastereodivergency was observed with these electrophiles, which differ from those previously discussed since they interact with boronate complex **2 a** to give 3‐membered heterocyclic onium ions that are ring‐opened on 1,2‐metallate rearrangement. Pleasingly, a simple proton could act as the electrophilic activator; boronic ester **3 o** was obtained in 53 % yield and 86 : 14 d.r., favoring the diastereomer with two *cis* methyl groups. Under conditions B, the reaction became less efficient.

The relative stereochemistry of the α,β,β‐trisubstituted cyclopentyl boronic esters was established on the basis of the ^1^H NMR signals of homobenzylic quaternary carbon substituents, which displayed remarkable variation in chemical shift between each diastereomer as a result of the shielding influence of the aryl group.^[16]^ In the case of α,β‐disubstituted boronic esters, 1D NOESY experiments allowed the elucidation of relative stereochemistry (see the Supporting Information for full details).

A tentative model is proposed to account for the diastereoselectivity observed (Scheme 4). Alkenyl boronate complexes bearing an α‐tertiary stereocenter can adopt conformation **I** or **II**. However, **II** suffers from destabilizing gauche interactions between the large R group and the methyl group of the pinacol ester. Reaction through either a concerted (blue arrows) or stepwise (grey arrows) mechanism via conformation **I** leads to the observed major product. The energy differences between the reactive conformations of alkenyl boronate complexes bearing an α‐quaternary stereocenter are more finely balanced, and so the influence on diastereoselectivity of both the electrophile and solvent is more difficult to rationalize.^[17]^

**Scheme 4:**
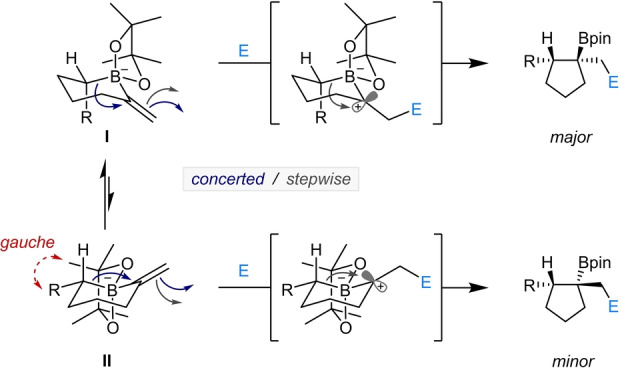
Proposed stereochemical model for the addition of electrophiles to alkenyl boronate complexes bearing an α‐tertiary stereocenter.

Finally, we sought to demonstrate the utility of our methodology in the construction of cyclopentane‐containing natural product (+)‐herbertene‐1,14‐diol,[^1a^, ^18^] assembling the two contiguous, fully substituted stereocenters with high diastereo‐ and enantiocontrol (Scheme 5). We envisaged that tertiary boronic ester **1 i** could be accessed from enantioenriched carbamate **9** and boronic ester **10** through lithiation–borylation methodology. Carbamate **9** was synthesized in 96 : 4 e.r. over three high yielding steps from ketone **6**: protection of the phenol as the methyl ether, Noyori asymmetric reduction to give enantioenriched alcohol **8**, and carbamoylation.^[19]^ The lithiation–borylation reaction of **9** with **10** proceeded in high yield and with 100 % enantiospecificity. Subsequent alkenyl boronate complex (**2 h**) formation followed by Brønsted acid‐induced ring contractive 1,2‐metallate rearrangement gave cyclopentyl boronic ester **3 p** in moderate yield and as an 85 : 15 mixture of diastereomers, separable by column chromatography. In order to incorporate the carbinol moiety we considered employing the Matteson homologation reaction,^[20]^ followed by boronic ester oxidation. However, this was unsuccessful due to the severe steric hindrance of boronic ester **3 p**, resulting in decomposition of the carbenoid outcompeting boronate complex formation. Fortunately, boronic ester **3 p** could undergo Zweifel olefination to give **11**, introducing a vinyl group and a second all‐carbon quaternary center. Ozonolysis of **11** followed by reduction by NaBH_4_ afforded alcohol **12** in 96 : 4 e.r., confirming that the reaction sequence proceeded with 100 % enantiospecificity. Direct demethylation of the highly hindered phenol initially proved challenging and was unsuccessful using standard conditions (EtSNa or BBr_3_), which is likely why a previous synthesis used an alternative three‐step sequence.^[21]^ We ultimately found that direct demethylation of **12** could be achieved using HPPh_2_ and *t*‐BuOK,^[22]^ completing the total synthesis of (+)‐herbertene‐1,14‐diol.

**Scheme 5:**
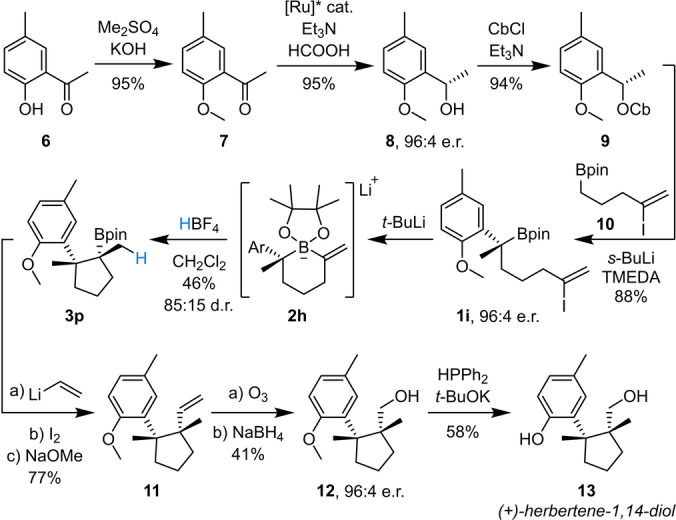
Asymmetric total synthesis of (+)‐herbertene‐1,14‐diol. [Ru]*=RuCl(*p*‐cymene)[(*S*,*S*)*‐*Ts‐DPEN]. Cb=*N*,*N*‐diisopropylcarbamoyl.

In conclusion, the electrophile‐induced ring contractive 1,2‐metallate rearrangement of 6‐membered cyclic alkenyl boronate complexes has been developed. Enantioenriched boronate complexes could be activated with both carbon‐ and heteroatom‐based electrophiles to undergo enantiospecific and highly diastereoselective ring contraction, allowing the synthesis of cyclopentyl boronic esters bearing contiguous, fully substituted stereocenters. Remarkable solvent‐induced diastereodivergency was observed in the case of some carbon‐based electrophiles, allowing access to complementary diastereomeric pairs from the same starting materials. The methodology has been applied in the asymmetric total synthesis of (+)‐herbertene‐1,14‐diol.

## Conflict of interest

The authors declare no conflict of interest.

## Supporting information

As a service to our authors and readers, this journal provides supporting information supplied by the authors. Such materials are peer reviewed and may be re‐organized for online delivery, but are not copy‐edited or typeset. Technical support issues arising from supporting information (other than missing files) should be addressed to the authors.

Supporting Information

## Data Availability

The data that support the findings of this study are available in the Supporting Information of this article.
