# Supplementary material for: Diastereodivergent Synthesis of Cyclopentyl Boronic Esters Bearing Contiguous Fully Substituted Stereocenters
Source: Angew Chem Int Ed Engl. 2022 Jun 23;61(35):e202205816. doi: 10.1002/anie.202205816 (PMC9542923; doi:10.1002/anie.202205816)

Angewandte Chemie
Eine Zeitschrift der Gesellschaft Deutscher Chemiker

Supporting Information

Diastereodivergent Synthesis of Cyclopentyl Boronic Esters Bearing Contiguous Fully Substituted Stereocenters

*M. E. Fairchild, A. Noble, V. K. Aggarwal**

TABLE OF CONTENTS

LIST OF SUPPLEMENTARY SCHEMES, FIGURES AND TABLES 2

TABLE OF CHARACTERISED PRODUCTS WITH HYPERLINKS 3

1. MATERIALS AND GENERAL METHODS 6

1.1. Glassware, Solvents and Reagents 6

1.2. Chromatography and Instrumentation 6

1.3. Naming of Compounds 7

2. EXPERIMENTAL DATA 8

2.1 Reaction Optimisation 8

2.1.1. Reaction Solvent and the Effect on Diastereoselectivity 8

2.2. Synthesis of Starting Boronic Esters 11

2.2.1. General Procedures 11

2.3. Synthesis of Cyclopentyl Boronic Esters 28

2.3.1. General Procedures 28

2.3.2. Substrate Scope 30

2.3.3. Electrophile Scope 48

2.4. Application to the Total Synthesis of (+)-Herbertene-1,14-diol 69

2.4.1 Optimisation of Conditions for the Ring Contractive 1,2-Metallate Rearrangement 78

2.4.2 An Alternative Boronic Ester Functionalisation: Homologation 79

2.4.3 Screening of Conditions for the Demethylation of the Aryl Methyl Ether 79

3. DETERMINATION OF RELATIVE STEREOCHEMISTRY 80

3.1. Ring Current Effects for the Determination of Relative Stereochemistry 80

3.2. One Dimensional nOe NMR Experiments for the Determination of Relative Stereochemistry............84

4. PROPOSED MODEL FOR DIASTEREOSELECTIVITY 86

4.1. α-Tertiary Stereocentres 86

4.2. α-Quaternary Stereocentres 86

5. REFERENCES 88

6. SPECTROSCOPIC DATA 90

LIST OF SUPPLEMENTARY SCHEMES, FIGURES AND TABLES

Scheme S1: ^{11}B NMR spectrum showing full formation of boronate complex **2a**. 8
Table S1: Electrophile addition conditions screening. 9
Scheme S2: Unsuccessful electrophiles under conditions A. 68
Scheme S3: The influence of the identity of the vinyl halide on product formation. 68
Table S2: Total synthesis of (+)-herbertene-1,14-diol – Electrophile addition conditions screening. 78
Table S3: Total synthesis of (+)-herbertene-1,14-diol – Boronic ester homologation attempts. 79
Table S4: Total synthesis of (+)-herbertene-1,14-diol – Demethylation attempts. 79
Figure S1: The ring current in an aromatic ring and its influence on adjacent nuclei. 80
Figure S2: The influence of ring currents in a 5-membered lactone. 80
Figure S3: The influence of ring currents in a silyl diether compounds with a 5-membered chelate structure. 81
Figure S4: Comparison between the chemical shifts of the methylene and methyl protons in herbertene-1,14-diol and herbertene-1,15-diol. 81
Figure S5: (a) Comparison between the chemical shifts of the alcohol and methyl protons in the diastereomers of **3o'**; (b) Comparison between the chemical shifts of the pinacol and methyl protons in the diastereomers of **3o**. 81
Figure S6: Electrophile scope – Comparison between the chemical shifts of the pinacol and methylene protons in cyclopentyl boronic esters. 82
Figure S7: Substrate scope – Comparison between the chemical shifts of the pinacol and methylene protons in cyclopentyl boronic esters. 83
Figure S8: Total synthesis of (+)-herbertene-1,14-diol – Comparison between the chemical shifts of the pinacol and methyl protons in intermediate boronic ester **3p**. 83
Scheme S4: Proposed stereochemical model for the addition of electrophiles to boronate complexes bearing an α-tertiary stereocentre. 86
Scheme S5: Proposed stereochemical model for the addition of electrophiles to boronate complexes bearing an α-quaternary stereocentre. 87

TABLE OF CHARACTERISED PRODUCTS WITH HYPERLINKS

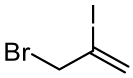

14

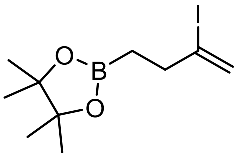

15

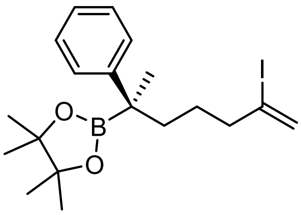

1a

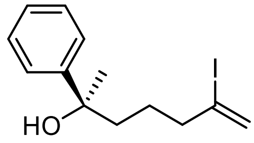

1a'

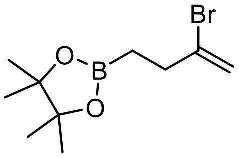

16

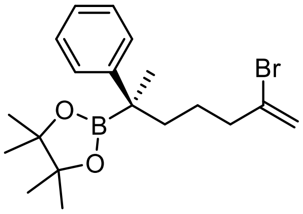

1h

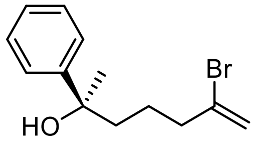

1h'

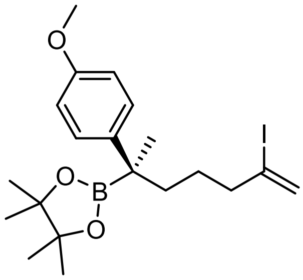

1b

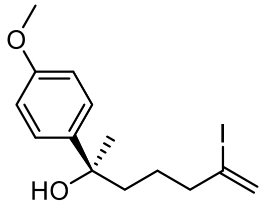

1b'

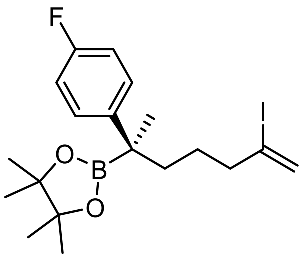

1c

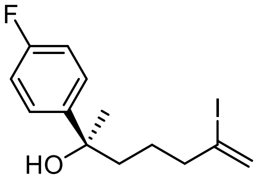

1c'

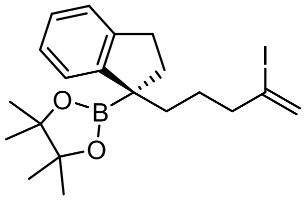

1d

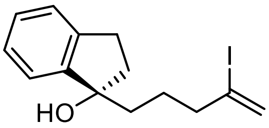

1d'

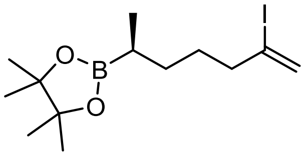

1e

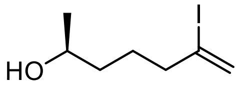

1e'

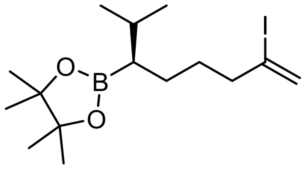

1f

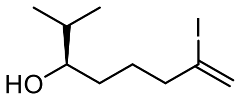

1f'

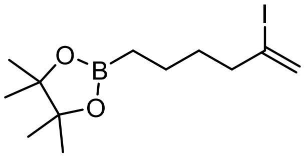

1g

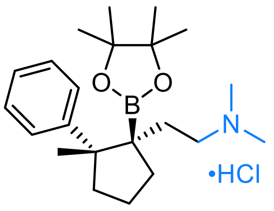

3a.d[1]

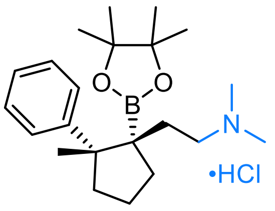

3a.d[2]

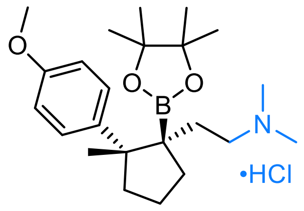

3b.d[1]

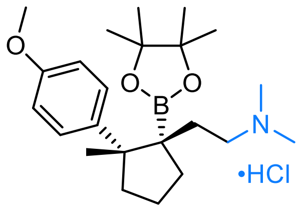

3b.d[2]

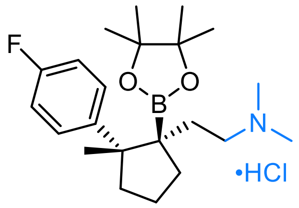

3c.d[1]

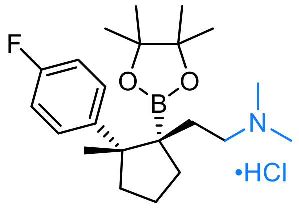

3c.d[2]

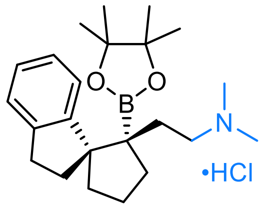

3d.d[2]

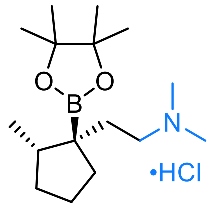

3e

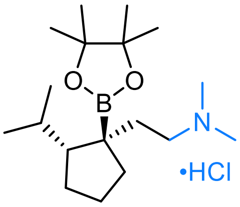

3f

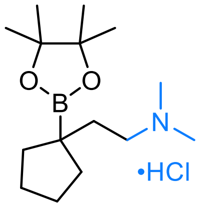

3g

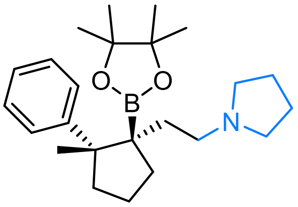

3h.d[1]

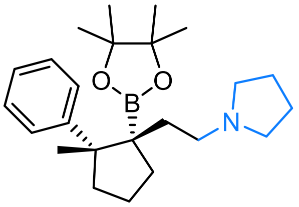

3h.d[2]

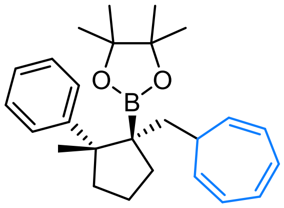

3i.d[1]

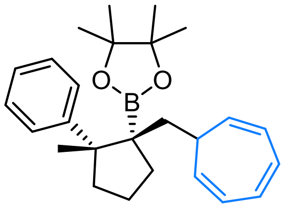

3i.d[2]

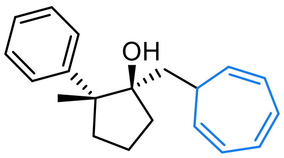

3i'.d[1]

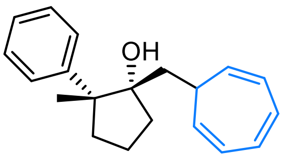

3i'.d[2]

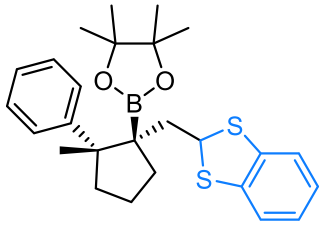

3j.d[1]

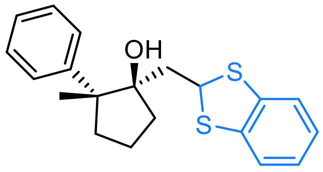

3j'.d[1]

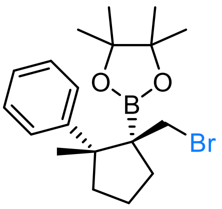

3k.d[2]

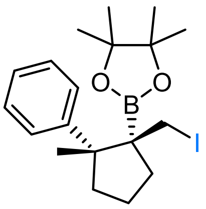

3l.d[2]

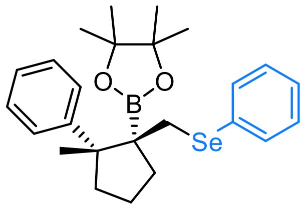

3m.d[2]

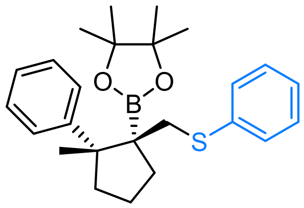

3n.d[2]

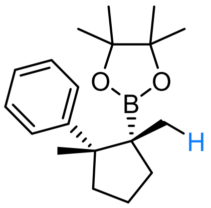

3o.d[2]

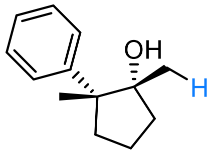

3o'.d[2]

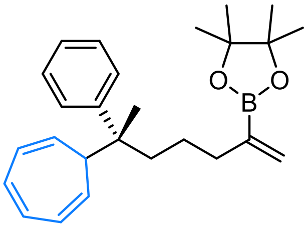

5

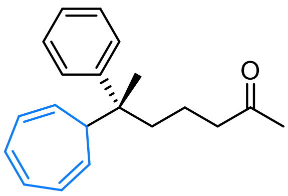

5'

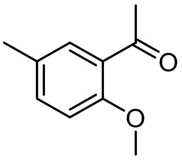

7

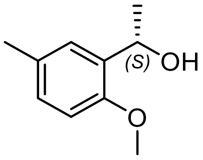

8

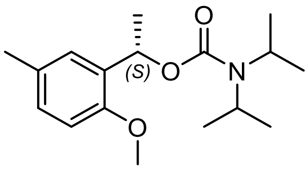

9

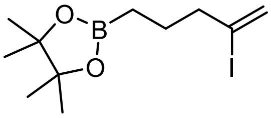

10

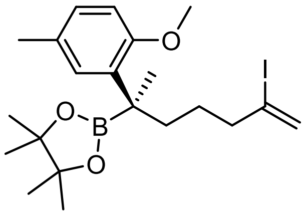

1i

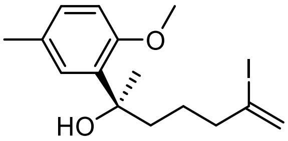

1i'

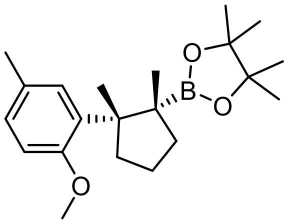

3p

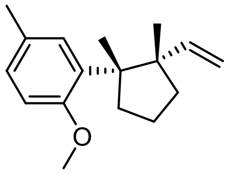

11

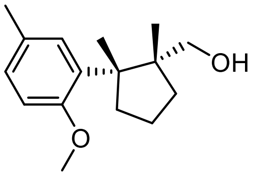

12

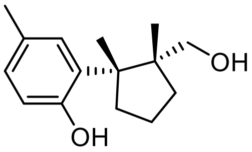

13

1. MATERIALS AND GENERAL METHODS

1.1. Glassware, Solvents and Reagents

All manipulations were performed with oven-dried (130 °C for a minimum of 12h) or flame-dried glassware using standard Schlenk techniques under an atmosphere of nitrogen, unless otherwise stated.

All anhydrous solvents were commercially supplied or dried using an Anhydrous Engineering alumina column drying system (THF, toluene, Et_2O, MeCN, CH_2Cl_2). Reagents were purchased from commercial sources and used as received. ***Exceptions***: *N*-bromosuccinimide (NBS) was recrystallised from boiling water. N,N,N′,N′-tetramethylethylenediamine (TMEDA), triethylamine, and propionitrile (EtCN) were distilled over CaH_2 under an inert atmosphere at standard pressure. All carbamates were prepared according to literature procedures and are referenced in the text. All organolithium reagents were titrated against *N*-benzylbenzamide.[1]

1.2. Chromatography and Instrumentation

Thin layer chromatography (TLC) was performed using Merck Kieselgel 60 F254 fluorescent treated silica, which was visualised under UV light, or by staining with aqueous basic potassium permanganate followed by heating, *p*-anisaldehyde solution followed by heating, Hanessian's stain (CAM stain) followed by heating, or an ethanolic solution of phosphomolybdic acid followed by heating.

Flash column chromatography was conducted using Sigma-Aldrich silica gel (60 Å, 230–400 mesh, 40-63 µm).

NMR spectra were recorded at various field strengths, as indicated, using Bruker 400 MHz, Varian VNMR 400 MHz, Varian VNMR 500 MHz, or Bruker Cryo 500 MHz for ^{1}H, ^{11}B, ^{13}C and ^{19}F acquisitions. All NMR spectra were recorded at 25 °C unless otherwise stated. Chemical shifts (δ) are reported in parts per million (ppm) and referenced $CDCl_3$ (^{1}H: 7.26 ppm; ^{13}C: 77.0 ppm) or CD_2Cl_2 (^{1}H: 5.32 ppm; ^{13}C: 53.5 ppm). Coupling constants (*J*) are given in Hertz (Hz) and refer to apparent multiplicities (s = singlet, d = doublet, t = triplet, q = quartet, quin = quintet, hex = hextet, hept = heptet, m = multiplet, br = broad signal, dd = doublet of doublets, etc.). The ^{1}H NMR spectra are reported as follows: chemical shift (multiplicity, coupling constants, number of protons). NMR yields are determined, unless otherwise stated, using dibromomethane (CH_2Br_2) as the internal standard.

High resolution mass spectra (HRMS) were recorded on a Bruker Daltonics MicrOTOF II by Electrospray Ionisation (ESI); a Thermo Scientific QExactive by Electron Ionisation (EI); a Thermo Scientific Orbitrap Elite by ESI or Atmospheric Pressure Chemical Ionisation (APCI); or a Bruker UltrafleXtreme by Matrix-assisted Laser Desorption/Ionisation (MALDI).

IR spectra were recorded neat as a thin film on a Perkin Elmer Spectrum One FT-IR. Selected absorption maxima (ν_{max}) are reported in wavenumbers (cm^{-1}) and broad maxima are indicated (br).

Chiral high-performance liquid chromatography (HPLC) analyses were performed on an Agilent 1100 system with Daicel Chiralpak IA, IB and IC columns with the isocratic gradient specified.

Chiral supercritical fluid chromatography (SFC) analyses were performed on a Waters (Thar) system with a Whelk-O1 column with the isocratic gradient specified.

Optical rotations ($[\alpha]_D^T$) were measured in CH_2Cl_2 (unless otherwise indicated) using a Bellingham & Stanley ADP 220 Polarimeter.

1.3. Naming of Compounds

Compound names are those generated by ChemDraw Professional 20.0 software (PerkinElmer), following the IUPAC nomenclature.

2. EXPERIMENTAL DATA

2.1 Reaction Optimisation

2.1.1. Reaction Solvent and the Effect on Diastereoselectivity

Boronic ester **1a** was subjected to lithium-halogen exchange conditions, employing 2.1 equivalents of *tert*-butyl lithium.[2] Full formation of boronate complex **2a** was confirmed by ^{11}B NMR, with a characteristic peak observed at 6.7 ppm (Scheme S1).

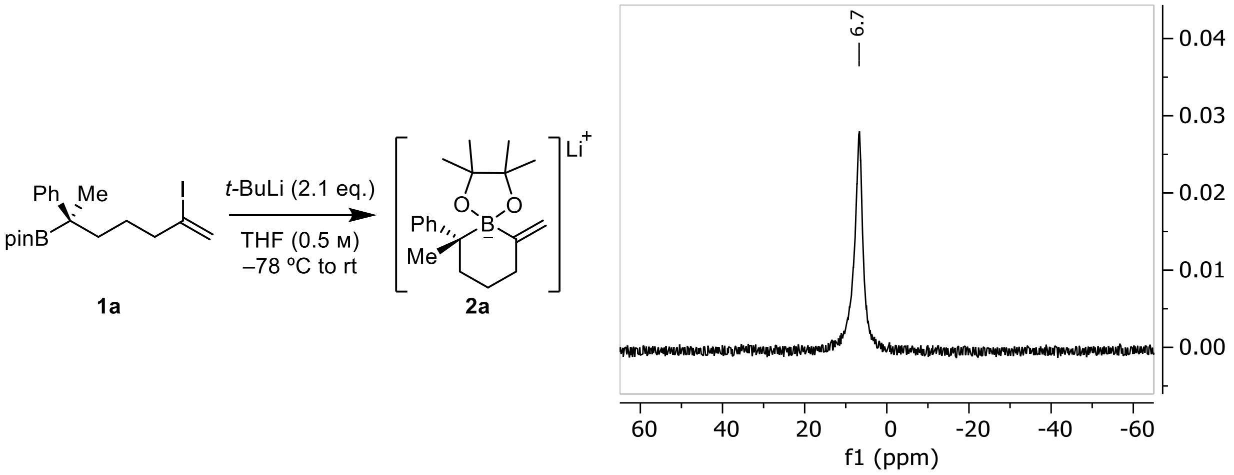

Scheme S1: ^{11}B NMR spectrum showing full formation of boronate complex 2a.

A screen of electrophile addition conditions was then conducted, varying the reaction solvent and temperature (Table S1a and S1b).

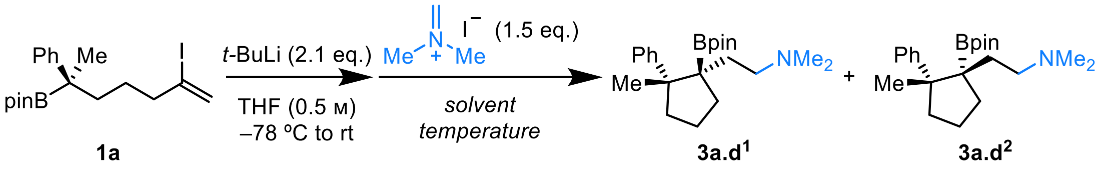

entry	solvent (x M)	temperature (°C)	^{1}H NMR yield (%)[a]	d^1/d^2 [b]
**1**	**THF (0.5 M)**	**–78 °C 2h, then rt[c]**	**70 (73)[d]**	**89:11**
2	EtCN (0.5 M)	–78 °C 2h, then rt[c]	91	73:27
3	MeCN (0.5 M)	–40 °C 2h, then rt[c]	73	68:32
4	DMF (0.5 M)	–40 °C 2h, then rt[c]	39	80:20
5	CH_2Cl_2 (0.5 M)	–78 °C 2h, then rt[c]	50	75:25
6	Et_2O (0.5 M)	–78 °C 2h, then rt[c]	62	84:16
7	THF (0.5 M)	–78 °C	0	-
8	THF (0.5 M)	–40 °C	0	-
9	THF (0.5 M)	rt	72	84:16
10	1:1 THF/MeCN (0.25 M)	–40 °C 2h, then rt[c]	78	81:19

11	1:1 THF/DMF (0.25 м)	–40 °C 2h, then rt[c]	56	86:14
12	1:1 THF/MeOH (0.25 м)	–78 °C 2h, then rt[c]	64	28:72
13	1:1 THF/HFIP (0.25 м)	–40 °C 2h, then rt[c]	83	10:90
14	10% *t*-BuOH in THF (0.25 м)	–78 °C 2h, then rt[c]	84	70:30
**15**	**1:1 THF/TFE (0.25 м)**	**–78 °C 2h, then rt[c]**	**96 (99)[d]**	**14:86**
16	10% TFE in THF (0.25 м)	–78 °C 2h, then rt[c]	82	37:63
17	5:1 TFE/THF (0.25 м)	–60 °C 2h, then rt[c]	76	11:89
18	TFE (0.25 м)	–40 °C 2h, then rt[c]	*messy*	-
19	1:1 THF/TFE (0.25 м)	–60 °C	17	13:87
20	1:1 THF/TFE (0.25 м)	–40 °C	32	12:88

Table S1: Electrophile addition conditions screening.

All reactions conducted on a 0.1 mmol scale unless otherwise indicated. [a] Yield determined by ^{1}H NMR analysis using CH_2Br_2 as an internal standard. Isolated yields are given in parentheses. [b] Ratio determined by ^{1}H NMR analysis. [c] The reaction mixture was allowed to slowly warm to rt overnight in the cooling bath (either the cryostat cooling device was turned off or the replenishment of dry ice was halted). [d] 0.2 mmol scale. Isolated as the hydrochloride salt.

Key observations:

- THF is the optimal solvent for the electrophile addition step in terms of yield and diastereoselectivity.
- The slow warming sequence is necessary for reactivity, likely due to poor electrophile solubility at low temperature.
- The addition of the electrophile at low temperatures followed by warming to room temperature is not necessary for good diastereoselectivity in this system. However it was maintained to account for variations in electrophile solubility.
- The addition of acetonitrile and *N*,*N*-dimethylformamide (DMF) co-solvents (to the boronate complex solved in THF at –40 °C prior to electrophile addition) did not improve yield nor diastereoselectivity with respect to pure THF but did with respect to the pure solvent.
- The addition of protic co-solvents (to the boronate complex solved in THF at –78/–40 °C prior to electrophile addition) reverses the diastereoselectivity of the reaction (except in entry 14). The origin of this reversal is not clear. In similar chemistry, where alkenyl boronate complexes[3] or bicyclo[1.1.0]butyl boronate complexes[4] are reacted with electrophiles, the addition of a co-solvent with hydrogen bond donating sites (such as methanol, 2,2,2-trifluoroethanol (TFE), 1,1,1,3,3,3-hexafluoro-2-propanol (HFIP), and *tert*-butyl alcohol) altered the diastereoselectivity of the reaction. It is thought that the alcohol hydrogen bonds to the Lewis basic pinacol oxygens in the boronate complex, modulating the nucleophilicity of the C–B bonds and allowing the 1,2-metallate rearrangement to occur with improved selectivity. In this case, the addition of the hydrogen bond donating solvent could both alter the electronics and the sterics of the boronate complex, both known to exert influence over the diastereoselectivity of a transformation.
- TFE is the optimal co-solvent for the electrophile addition step and a 1:1 THF/TFE ratio is both the

highest yielding and most operationally straight-forward.

- The slow warming sequence with 1:1 THF/TFE is necessary for high yields but seems to have no observable effect on diastereoselectivity in this system.

2.2. Synthesis of Starting Boronic Esters

2.2.1. General Procedures

2.2.1.1. General Procedure A: the synthesis of α-aryl boronic esters

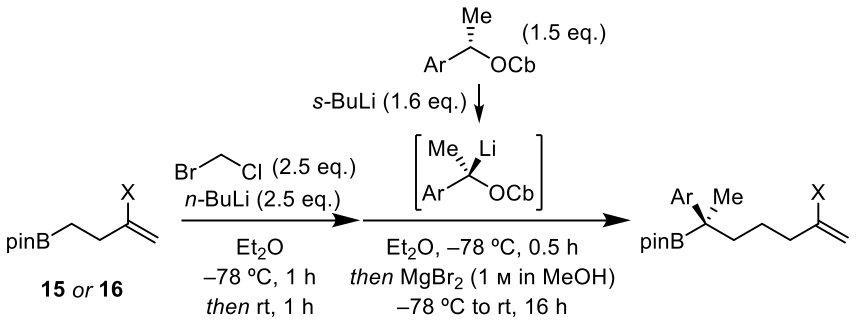

According to a combination of modified literature procedures,[2,5,6] boronic ester **15** or **16** (1.0 eq.) was solved in Et_2O (0.1 м) and cooled to –78 °C (dry ice/acetone). Bromochloromethane (2.5 eq.) was added dropwise to the solution, followed by *n*-BuLi (1.6 м in hexanes; 2.5 eq.) *via* syringe pump (0.03 mL/min). Following the addition, the reaction mixture was stirred at –78 °C for 1 h and then warmed to rt and stirred at this temperature for 1 h. The reaction mixture was filtered through a silica plug (eluting Et_2O) and concentrated *in vacuo*. The crude product was used without further purification.

The diisopropylcarbamate (*racemic* or *enantioenriched*; 1.5 eq.) was solved in anhydrous Et_2O (0.2 м) and the solution cooled to –78 °C (dry ice/acetone). *s*-BuLi (1.3 м in cyclohexane/hexane 92:8; 1.6 eq.) was added dropwise to the solution. The reaction mixture was stirred at –78 °C for 30 minutes whereupon a 1 м solution in Et_2O of the crude boronic ester was added dropwise. Following subsequent stirring at –78 °C for 30 minutes, a 1 м solution of $MgBr_2$ in MeOH (1.8 eq.) was added at this temperature. The reaction mixture was then warmed to rt and stirred at this temperature overnight. Confirmation of full conversion to the boronic ester by ^{11}B NMR preceded quenching with water (10 mL/mmol) and the aqueous layer extracted with Et_2O (3 × 10 mL/mmol). The combined organics were washed with brine, dried over $MgSO_4$ and concentrated *in vacuo*. The crude product was purified by flash column chromatography to give the product.

2.2.1.2. General Procedure B: the synthesis of α-aryl boronic esters using TMEDA in lithiation

As with General Procedure **A**, but with the addition of TMEDA (1.8 eq.) to the solution of diisopropylcarbamate in Et_2O, prior to lithiation.

2.2.1.3. General Procedure C: the synthesis of α-alkyl boronic esters

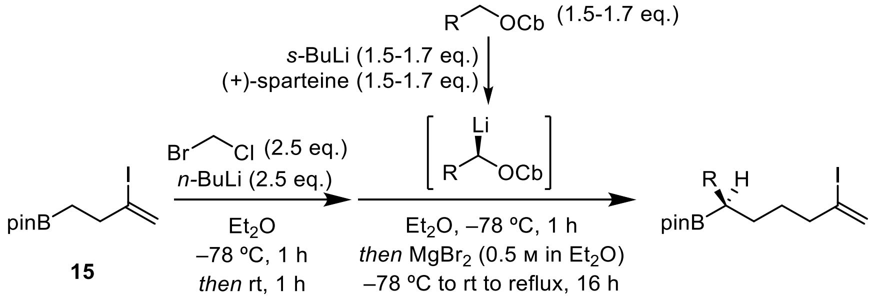

According to a combination of modified literature procedures,[2,7] boronic ester **15** (1.0 eq.) was solved in Et_2O (0.1 м) and cooled to –78 °C (dry ice/acetone). Bromochloromethane (2.5 eq.) was added dropwise to the solution, followed by *n*-BuLi (1.6 м in hexanes; 2.5 eq.) *via* syringe pump (0.03 mL/min). Following the addition, the reaction mixture was stirred at –78 °C for 1 h and then warmed to rt and stirred at this temperature for 1 h. The reaction mixture was filtered through a silica plug (eluting Et_2O) and concentrated *in vacuo*. The crude product was used without further purification.

Enantioenriched: The carbamate (1.5 eq.) and (+)-sparteine (1.5 eq.) were solved in Et_2O (0.1 м) and cooled to –78 °C (dry ice/acetone). *s*-BuLi (1.3 м in cyclohexane/hexane 92:8; 1.5 eq.) was added dropwise to this mixture. The resultant mixture was stirred at –78 °C for 5 h whereupon a 1 м solution in Et_2O of the crude boronic ester was added dropwise. Following subsequent stirring at –78 °C for 1 h, a solution of $MgBr_2$ in Et_2O [freshly made from Mg turnings (2.5 eq.) and 1,2-dibromoethane (1.5 eq.) in Et_2O (0.5 м)] was added at this temperature. The reaction mixture was stirred at –78 °C a further 30 minutes whereupon it was warmed to rt and heated to reflux overnight. Confirmation of full conversion to the boronic ester by ^{11}B NMR preceded quenching the reaction mixture with 2 м aqueous HCl (10 mL/mmol). The organic layer was washed with 2 м aqueous HCl (4 × 10 mL/mmol) for the recovery of the (+)-sparteine and the combined aqueous washes were extracted with Et_2O (4 × 10 mL/mmol). The combined organics were washed with brine, dried over $MgSO_4$ and concentrated *in vacuo*. The crude product was purified by flash column chromatography to give the product.

Racemic: As above, replacing (+)-sparteine (1.5 eq.) with TMEDA (1.5 eq.). The work up procedure can be simplified: The reaction mixture was quenched with water (10 mL/mmol) and the aqueous layer extracted with Et_2O (3 × 10 mL/mmol). The combined organics were washed with brine, dried over $MgSO_4$ and concentrated *in vacuo*.

2.2.1.4. General Procedure D: the oxidation of boronic esters to alcohols

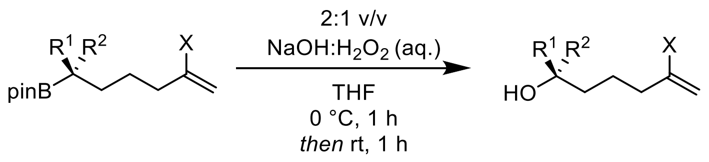

According to a literature procedure,[2] the boronic ester (0.05 mmol, 1.0 eq.) was solved in THF (0.33 mL) and cooled to 0 °C (ice water). A 2:1 v/v solution of 2 м aqueous NaOH (0.3 mL) and 30% w/w aqueous H_2O_2 (0.17 mL) was added dropwise to the solution. The reaction mixture was allowed to stir at this temperature for 1 h, followed by warming to rt and stirring at rt for 1 h. Completion of the reaction was monitored by TLC, whereupon the reaction mixture was diluted with EtOAc (1 mL) and quenched with saturated aqueous $Na_2S_2O_3$ (1 mL). The aqueous layer was extracted with EtOAc (3 × 1 mL). The combined organics were washed with brine, dried over $MgSO_4$ and concentrated *in vacuo*. The crude product was purified by flash column chromatography to give the product.

3-Bromo-2-iodoprop-1-ene (14)

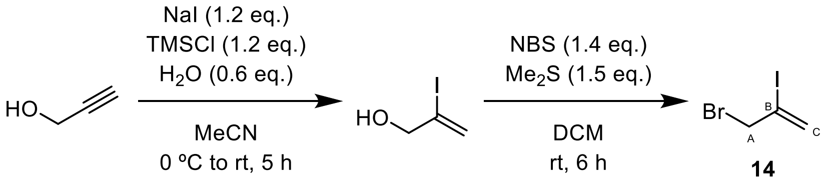

14

According to a literature procedure,[2] sodium iodide (26 g, 103 mmol, 1.2 eq.) was solved in anhydrous acetonitrile (38 mL) and cooled to 0 °C (ice water). TMSCl (13 mL, 103 mmol, 1.2 eq.) was added dropwise and the resultant solution stirred at 0 °C for 15 minutes. After which time, H_2O (0.95 mL, 50 mmol, 0.6 eq.) was added dropwise, followed by propargyl alcohol (5 mL, 86 mmol, 1.0 eq.) dropwise. The reaction mixture was warmed to rt over 1 h (by halting the replenishment of the ice in the cooling bath) and then stirred at rt for 4 h whereupon it was quenched with saturated aqueous $NaHCO_3$ and saturated aqueous $NaHSO_3$. The aqueous phase was extracted with Et_2O (3 × 50 mL) and the combined organics were washed with brine, dried over $MgSO_4$ and concentrated *in vacuo*. The crude product was purified by flash column chromatography (pentane/Et_2O 20:1 to 10:1) to give 2-iodoprop-2-en-1-ol as a brown oil. NBS (11.2 g, 63 mmol, 1.4 eq.) was solved in CH_2Cl_2 (16 mL) and cooled to -25 °C (cryostat). To this solution Me_2S (4.9 mL, 67 mmol, 1.5 eq.) was added dropwise. The reaction mixture was stirred at this temperature for 20 minutes whereupon 2-iodoprop-2-en-1-ol (8.24 g, 45 mmol, 1.0 eq.) in CH_2Cl_2 (4.5 mL) was added dropwise and the resultant mixture warmed to rt. After 3 h stirring at rt, the reaction mixture was diluted with pentane (20 mL) and poured over ice water. The organic phase was washed with brine, dried over $MgSO_4$ and concentrated *in vacuo*. The crude product was purified by flash column chromatography (pentane) to give vinyl iodide **14** as a bright red oil (7.27 g, 33 mmol) in 38% yield over 2 steps.

NMR Spectroscopy:

^{1}H NMR (400 MHz, $CDCl_3$): δ_H 6.45 (dt, J = 1.9, 1.3 Hz, 1H, H_C), 5.88 (dt, J = 1.9, 0.5 Hz, 1H, H_C), 4.23 (dd, J = 1.3, 0.5 Hz, 2H, H_A) ppm;

^{13}C NMR (101 MHz, $CDCl_3$): δ_C 129.8 (C_C), 102.9 (C_B), 41.4 (C_A) ppm.

All recorded spectroscopic data matched those previously reported in the literature.[2]

2-(3-Iodobut-3-en-1-yl)-4,4,5,5-tetramethyl-1,3,2-dioxaborolane (15)

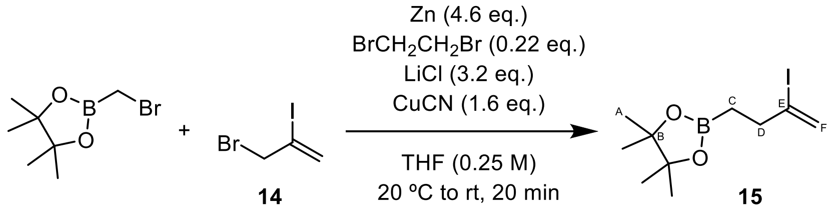

According to a literature procedure,[2] zinc powder (4.87 g, 75 mmol, 4.6 eq.) was suspended in THF (9 mL) at rt and 1,2-dibromoethane (0.31 mL, 3.6 mmol, 0.22 eq.) was added dropwise. To this suspension, (bromomethyl)boronic acid pinacol ester (4.3 mL, 24 mmol, 1.5 eq.) in THF (24 mL) was added dropwise *via* syringe pump (1.0 mL/min). Following this addition, the suspension was stirred for 30 minutes and then stirring was stopped and the zinc allowed to settle. In the meantime, dry lithium chloride (2.20 g, 52 mmol, 3.2 eq.; dried

under vacuum at 200 °C) and copper(I) cyanide (2.32 g, 26 mmol, 1.6 eq.) were added to a flask which was then set under a nitrogen atmosphere, solved in THF (32 mL) and cooled to –20 °C (cryostat). To this solution the organozinc solution was added *via* syringe pump (1.00 mL/min). Following the addition, the reaction mixture was stirred at 5 °C (ice water) for 5 minutes and then cooled back to –20 °C whereupon vinyl iodide **14** (4.00 g, 16 mmol, 1.0 eq.) was added *via* syringe pump (0.1 mL/min). After the addition was completed, the reaction mixture was warmed to rt and stirred at rt for 20 minutes and then quenched with water (120 mL) and Et_2O (60 mL). The aqueous phase was washed with Et_2O (2 × 30 mL). The combined organics were washed with brine, dried over $MgSO_4$ and concentrated *in vacuo*. The crude product was purified by flash column chromatography (pentane/Et_2O 50:1, 25:1, 20:1) to give boronic ester **15** as a pale-yellow oil (3.89 g, 12 mmol) in 78% yield.

NMR Spectroscopy:

^{1}H NMR (400 MHz, $CDCl_3$): δ_H 6.02 (apparent q, *J* = 1.6 Hz, 1H, H_F), 5.64 (dt, *J* = 1.7, 0.8 Hz, 1H, H_F), 2.65–2.37 (m, 2H, H_D), 1.25 (s, 12H, H_A), 1.06–0.90 (m, 2H, H_C) ppm;

^{13}C NMR (101 MHz, $CDCl_3$): δ_C 124.0 (C_F), 114.7 (C_E), 83.3 (C_B), 40.3 (C_D), 24.8 (C_A) ppm. C_C not observed due to quadrupolar relaxation.

All recorded spectroscopic data matched those previously reported in the literature.[2]

(*S*)-2-(6-Iodo-2-phenylhept-6-en-2-yl)-4,4,5,5-tetramethyl-1,3,2-dioxaborolane (1a)

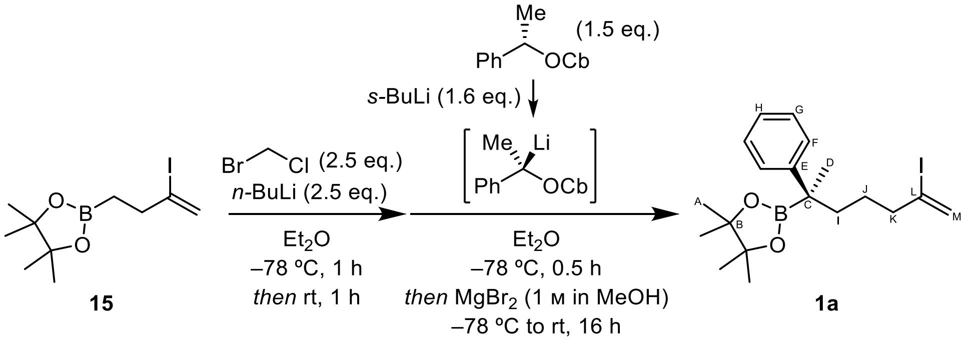

According to General Procedure A, boronic ester **15** (0.308 g, 1.0 mmol, 1.0 eq.) was reacted with bromochloromethane (0.16 mL, 2.5 mmol, 2.5 eq.) and *n*-BuLi (1.6 M in hexanes; 1.6 mL, 2.5 mmol, 2.5 eq.). The crude product was then reacted with (*S*)-1-phenylethyl diisopropylcarbamate[6] (0.374 g, 1.5 mmol, 1.5 eq.) and *s*-BuLi (1.3 M in cyclohexane/hexane 92:8; 1.2 mL, 1.6 mmol, 1.6 eq.). The crude product was purified by flash column chromatography (pentane/Et_2O 200:1 to 100:1) to give the product as a colorless oil (0.270 g, 0.63 mmol, 99:1 e.r.) in 63% yield over 2 steps.

The e.r. of boronic ester **1a** could not be determined by chiral HPLC/SFC. Instead, the boronic ester was oxidised and the e.r. of alcohol **1a'** was determined.

R_f = 0.4 (pentane/Et_2O 98:2).

$[\alpha]_D^{25}$: –1 (*c* = 5.4).

NMR Spectroscopy (*see spectra*):

^{1}H NMR (400 MHz, $CDCl_3$): δ_H 7.35–7.21 (m, 5H, $H_{F,\ G}$), 7.17–7.08 (m, 1H, H_H), 5.98 (apparent q, *J* = 1.4 Hz, 1H, H_M), 5.69–5.61 (m, 1H, $H_{M'}$), 2.41–2.32 (m, 2H, H_K), 1.86–1.60 (m, 2H, H_I), 1.52–1.38 (m, 2H, H_J), 1.36 (s, 3H, H_D), 1.22 (s, 6H, H_A), 1.21 (s, 6H, $H_{A'}$) ppm;

^{13}C NMR (101 MHz, $CDCl_3$): δ_C 147.1 (C_E), 128.1 (C_F *or* C_G), 126.8 (C_F *or* C_G), 125.2 (C_H *or* C_M), 125.1 (C_H *or* C_M), 112.6 (C_L), 83.4 (C_B), 46.0 (C_K), 38.1 (C_I), 25.3 (C_J), 24.6 (C_A), 21.6 (C_D) ppm; C_C not observed due to quadrupolar relaxation;

^{11}B NMR (128 MHz, $CDCl_3$): δ_B 32.9 ppm.

IR (film): ν_{max} 2977, 2925, 2852, 1616, 1467, 1379, 1312, 1140 cm^{-1}.

HRMS (ESI): m/z calc'd for $C_{19}H_{28}{}^{11}BINaO_2$ $[M+Na]^+$ 449.1123 found 449.1138.

(*R*)-6-Iodo-2-phenylhept-6-en-2-ol (1a')

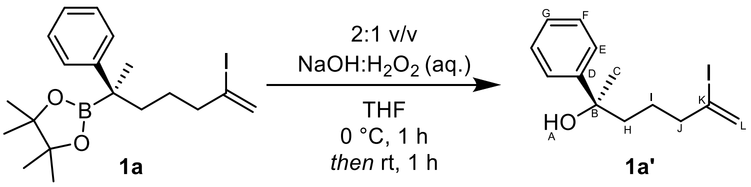

According to General Procedure **D**, vinyl iodide **1a** (21 mg, 0.05 mmol, 1.0 eq.) was reacted with a 2 м aqueous solution of NaOH (0.33 mL) and aqueous H_2O_2 (30% w/w, 0.17 mL) at 0 °C in THF (0.33 mL). The product (13 mg, 82%, 99:1 e.r.) was isolated as a colorless oil after purification by flash column chromatography (pentane/EtOAc 9:1).

R*f* = 0.3 (pentane/EtOAc 9:1).

$[\alpha]_D^{25}$**:** +8 (*c* = 2.8).

NMR Spectroscopy (*see spectra*):

^{1}H NMR (400 MHz, $CDCl_3$): δ_H 7.47–7.40 (m, 2H, H_E), 7.39–7.31 (m, 2H, H_F), 7.28–7.21 (m, 1H, H_G), 5.97 (apparent q, *J* = 1.4 Hz, 1H, H_L), 5.67 (apparent d, *J* = 1.5 Hz, 1H, $H_{L'}$), 2.37–2.29 (m, 2H, H_J), 1.85–1.73 (m, 2H, H_H), 1.58 (s, 3H, H_C), 1.55–1.45 (m, 1H, H_I), 1.45–1.31 (m, 1H, $H_{I'}$) ppm;

^{13}C NMR (101 MHz, $CDCl_3$): δ_C 147.7 (C_D), 128.3 (C_F), 126.7 (C_G), 125.7 (C_L), 124.8 (C_E), 112.2 (C_K), 74.5 (C_B), 45.2 (C_J), 42.4 (C_H), 30.2 (C_C), 23.5 (C_I) ppm.

IR (film): ν_{max} 3414 (br), 2924, 1616, 1494, 1446, 1373, 1143, 1055 cm^{-1}.

HRMS (ESI): m/z calc'd for $C_{13}H_{17}INaO$ $[M+Na]^+$ 339.0216 found 399.0212.

Chiral HPLC (IC column, 99:1 hexane/IPA, 1 mL/min, rt, 325 nm): 9.1 min (major), 10.0 min (minor), 99:1 e.r.

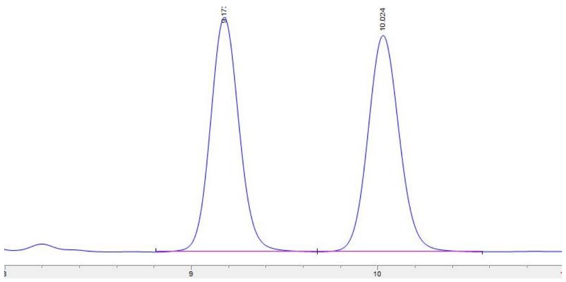

#	Time	Type	Area	Height	Width	Area%	Symmetry
1	9.173	BB	2270.3	212.3	0.1658	49.833	0.898
2	10.024	BB	2285.4	195.7	0.1818	50.167	0.898

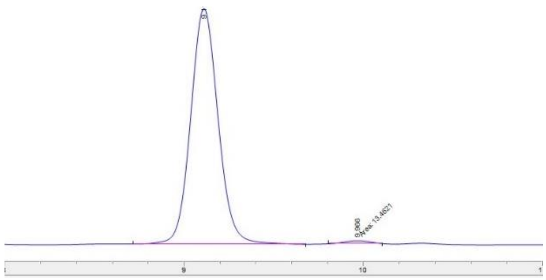

#	Time	Type	Area	Height	Width	Area%	Symmetry
1	9.11	BB	1126.1	105.8	0.1647	98.819	0.895
2	9.966	MM	13.5	1.4	0.1625	1.181	1.015

2-(3-Bromobut-3-en-1-yl)-4,4,5,5-tetramethyl-1,3,2-dioxaborolane (16)

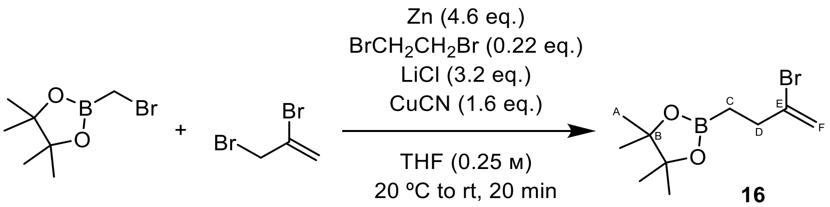

16

According to a literature procedure,[8] zinc powder (3.01 g, 46 mmol, 4.6 eq.) was suspended in THF (5 mL) at rt and 1,2-dibromoethane (0.19 mL, 2.2 mmol, 0.22 eq.) was added dropwise. To this suspension (bromomethyl)boronic acid pinacol ester (2.7 mL, 15 mmol, 1.5 eq.) in THF (14 mL) was added dropwise *via* syringe pump (1.0 mL/min). Following this addition, the suspension was stirred for 30 minutes and then stirring was stopped and the zinc allowed to settle. In the meantime, dry lithium chloride (1.36 g, 32 mmol, 3.2 eq.; dried under vacuum at 200 °C) and copper(I) cyanide (1.43 g, 16 mmol, 1.6 eq.) were added to a flask which was then set under a nitrogen atmosphere, solved in THF (20 mL) and cooled to –20 °C (cryostat). To this solution the organozinc solution was added *via* syringe pump (1.00 mL/min). Following the addition, the reaction mixture was stirred at 5 °C for 5 minutes and then cooled back to –20 °C whereupon 3-bromo-2-bromoprop-1-ene (0.98 g, 10 mmol, 1.0 eq.) was added *via* syringe pump (0.1 mL/min). After the addition was completed, the reaction mixture was warmed to rt and stirred at rt for 20 minutes and then quenched with water (80 mL) and Et_2O (40 mL). The aqueous phase was washed with Et_2O (2 × 30 mL). The combined organics were washed with brine, dried over $MgSO_4$ and concentrated *in vacuo*. The crude product was purified by flash column chromatography (pentane then pentane/Et_2O 9:1) to give boronic ester **16** as a pale-yellow oil (1.23 g, 4.7 mmol) in 47% yield.

NMR Spectroscopy:

^{1}H NMR (400 MHz, $CDCl_3$): δ_H 5.57 (apparent q, *J* = 1.4 Hz, 1H, H_F), 5.35 (apparent d, *J* = 1.5 Hz, 1H, H_F), 2.59–2.50 (m, 2H, H_D), 1.24 (s, 12H, H_A), 1.09–1.01 (m, 2H, H_C) ppm;

^{13}C NMR (101 MHz, $CDCl_3$): δ_C 136.7 (C_F), 115.1 (C_E), 83.3 (C_B), 36.1 (C_C), 24.8 (C_A) ppm. C_C not observed due to quadrupolar relaxation.

All recorded spectroscopic data matched those previously reported in the literature.[8]

(*S*)-2-(6-Bromo-2-phenylhept-6-en-2-yl)-4,4,5,5-tetramethyl-1,3,2-dioxaborolane (1h)

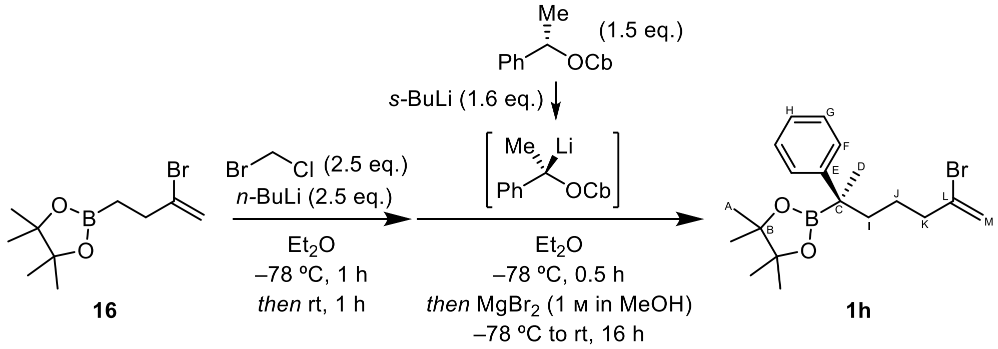

According to General Procedure A, boronic ester **16** (0.600 g, 2.3 mmol, 1.0 eq.) was reacted with bromochloromethane (0.37 mL, 5.7 mmol, 2.5 eq.) and *n*-BuLi (1.6 M in hexanes; 3.6 mL, 5.7 mmol, 2.5 eq.). The crude product was then reacted with (*S*)-1-phenylethyl diisopropylcarbamate[6] (0.631 g, 2.5 mmol, 1.1 eq.) and *s*-BuLi (1.3 M in cyclohexane/hexane 92:8; 2.8 mL, 3.7 mmol, 1.6 eq.). The crude product was purified by flash column chromatography (pentane/Et_2O 50:1) to give the product as a colorless oil (0.634 g, 1.7 mmol, 99:1 e.r.) in 73% yield over 2 steps.

The e.r. of boronic ester **1h** could not be determined by chiral HPLC/SFC. Instead, the boronic ester was oxidised and the e.r. of alcohol **1h'** was determined.

R_f = 0.6 (pentane/Et_2O 95:5).

$[\alpha]_D^{25}$: –3 (*c* = 5.4).

NMR Spectroscopy (*see spectra*):

^{1}H NMR (400 MHz, $CDCl_3$): δ_H 7.33–7.24 (m, 5H, $H_{F,G}$), 7.18–7.09 (m, 1H, H_H), 5.53 (apparent q, *J* = 1.4 Hz, 1H, H_M), 5.36 (apparent d, *J* = 1.6 Hz, 1H, $H_{M'}$), 2.44–2.33 (m, 2H, H_K), 1.86–1.74 (m, 1H, H_I), 1.73–1.61 (m, 1H, $H_{I'}$), 1.52–1.40 (m, 2H, H_J), 1.35 (s, 3H, H_D), 1.21 (s, 6H, H_A), 1.20 (s, 6H, $H_{A'}$) ppm;

^{13}C NMR (101 MHz, $CDCl_3$): δ_C 147.1 (C_E), 134.8 (C_M), 128.1 (C_F *or* C_G), 126.8 (C_F *or* C_G), 125.1 (C_H), 116.3 (C_L), 83.4 (C_B), 42.1 (C_K), 38.2 (C_I), 24.6 (C_J), 24.2 (C_A), 21.6 (C_D) ppm; C_C not observed due to quadrupolar relaxation;

^{11}B NMR (128 MHz, $CDCl_3$): δ_B 33.1 ppm.

IR (film): v_{max} 2977, 2945, 1629, 1467, 1379, 1349, 1312, 1141 cm^{-1}.

HRMS (ESI): m/z calc'd for $C_{19}H_{28}{}^{11}BBrNaO_2$ $[M+Na]^+$ 401.1261 found 401.1265.

(*R*)-6-Bromo-2-phenylhept-6-en-2-ol (1h')

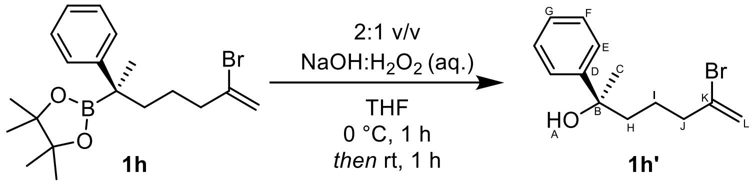

According to General Procedure **D**, vinyl bromide **1h** (19 mg, 0.05 mmol, 1.0 eq.) was reacted with a 2 м aqueous solution of NaOH (0.33 mL) and aqueous H_2O_2 (30% w/w, 0.17 mL) at 0 °C in THF (0.33 mL). The product (11 mg, 78%, 99:1 e.r.) was isolated as a colorless oil after purification by flash column chromatography (pentane/EtOAc 9:1).

R_f = 0.4 (pentane/EtOAc 9:1).

$[\alpha]_D^{25}$**:** +5 (*c* = 2.5).

NMR Spectroscopy (*see spectra*):

^{1}H NMR (400 MHz, $CDCl_3$): δ_H 7.46–7.39 (m, 2H, H_E), 7.37–7.31 (m, 2H, H_F), 7.26–7.21 (m, 1H, H_G), 5.50 (apparent q, *J* = 1.3 Hz, 1H, H_L), 5.35 (apparent d, *J* = 1.7 Hz, 1H, $H_{L'}$), 2.35 (apparent td, *J* = 7.2, 1.1 Hz, 2H, H_J), 1.82–1.74 (m, 2H, H_H), 1.66 (br s, 1H, H_A), 1.62–1.48 (m, 1H, H_I), 1.57 (s, 3H, H_C), 1.48–1.35 (m, 1H, $H_{I'}$) ppm;

^{13}C NMR (101 MHz, $CDCl_3$): δ_C 147.7 (C_D), 134.4 (C_K), 128.3 (C_F), 126.8 (C_G), 124.8 (C_E), 116.9 (C_L), 74.6 (C_B), 42.7 (C_J), 41.4 (C_H), 30.3 (C_C), 22.4 (C_I) ppm.

IR (film): ν_{max} 3421 (br), 2949, 1629, 1494, 1446, 1374, 1151 cm^{-1}.

HRMS (ESI): m/z calc'd for $C_{13}H_{17}BrNaO$ [M+Na]$^+$ 291.0355 found 291.0356.

Chiral HPLC (IC column, 98:2 hexane/IPA, 0.5 mL/min, rt, 325 nm): 12.9 min (major), 13.7 min (minor), 99:1 e.r.

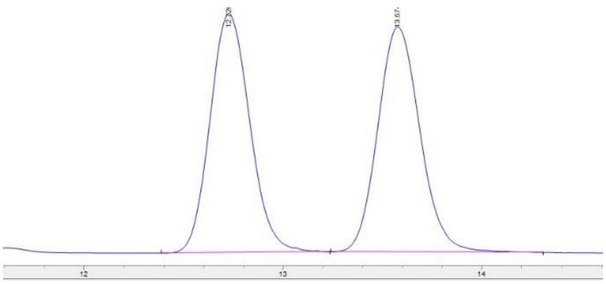

#	Time	Type	Area	Height	Width	Area%	Symmetry
1	12.726	BV R	3114.3	224	0.2142	50.035	0.87
2	13.574	BV R	3109.9	210.9	0.2265	49.965	0.849

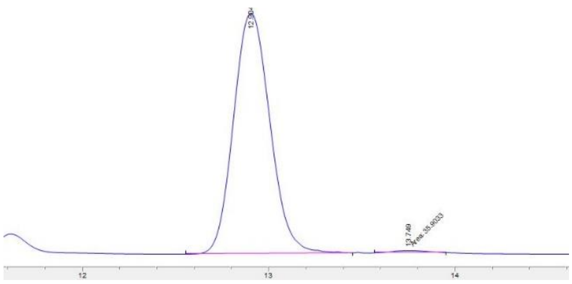

#	Time	Type	Area	Height	Width	Area%	Symmetry
1	12.904	BV R	4013.3	295.4	0.2075	99.113	0.883
2	13.749	MM	35.9	2.8	0.2132	0.887	0.799

(*S*)-2-(6-Iodo-2-(4-methoxyphenyl)hept-6-en-2-yl)-4,4,5,5-tetramethyl-1,3,2-dioxaborolane (1b)

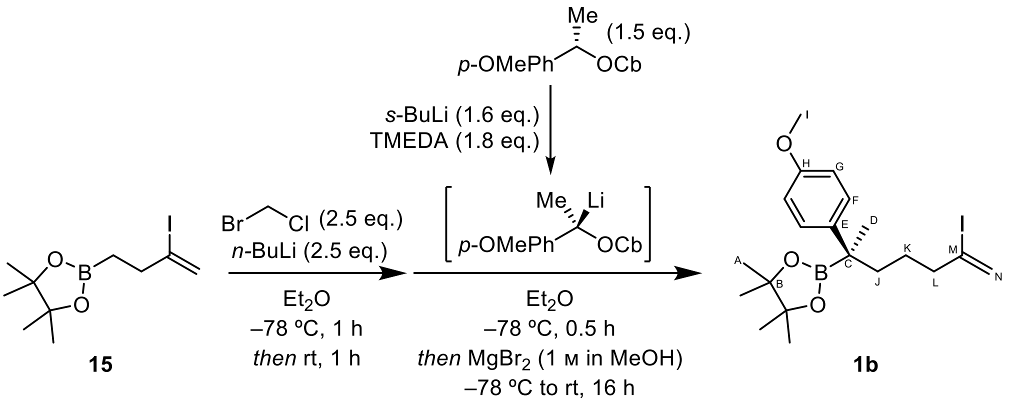

According to General Procedure **B**, boronic ester **15** (0.462 g, 1.5 mmol, 1.0 eq.) was reacted with bromochloromethane (0.24 mL, 3.8 mmol, 2.5 eq.) and *n*-BuLi (1.6 м in hexanes; 2.3 mL, 3.8 mmol, 2.5 eq.). The crude product was then reacted with (*S*)-1-(4-methoxyphenyl)ethyl diisopropylcarbamate[6] (0.629 g, 2.3 mmol, 1.5 eq.), TMEDA (0.40 mL, 2.7 mmol, 1.8 eq.) and *s*-BuLi (1.3 м in cyclohexane/hexane 92:8; 1.9 mL, 2.4 mmol, 1.6 eq.). The crude product was purified by flash column chromatography (pentane/Et_2O 9:1) to give the product as a colorless oil (0.498 g, 1.1 mmol, 95:5 e.r.) in 73% yield over 2 steps.

The e.r. of boronic ester **1b** could not be determined by chiral HPLC/SFC. Instead, the boronic ester was oxidised and the e.r. of alcohol **1b'** was determined.

R*f* = 0.3 (pentane/Et_2O 95:5).

$[\alpha]_D^{25}$**:** –3 (*c* = 5.0).

NMR Spectroscopy (*see spectra*):

^{1}H NMR (400 MHz, $CDCl_3$): δ_H 7.25–7.19 (m, 2H, H_F), 6.87–6.79 (m, 2H, H_G), 5.98 (apparent q, *J* = 1.4 Hz, 1H, H_N), 5.68–5.63 (m, 1H, $H_{N'}$), 3.78 (s, 3H, H_I), 2.42–2.30 (m, 2H, H_L), 1.83–1.69 (m, 1H, H_J), 1.61 (ddd, *J* = 13.1, 10.8, 5.7 Hz, 1H, $H_{J'}$), 1.43 (dddd, *J* = 14.5, 11.0, 7.1, 5.4 Hz, 2H, H_K), 1.33 (s, 3H, H_D), 1.21 (s, 6H, H_A), 1.21 (s, 6H, $H_{A'}$) ppm;

^{13}C NMR (101 MHz, $CDCl_3$): δ_C 157.2 (C_H), 139.1 (C_E), 127.7 (C_F), 125.2 (C_N), 113.5 (C_G), 112.7 (C_M), 83.3 (C_B), 55.2 (C_I), 46.1 (C_L), 38.3 (C_J), 25.4 (C_K), 24.7 (C_A), 24.6 ($C_{A'}$), 21.8 (C_D) ppm; C_C not observed due to quadrupolar relaxation;

^{11}B NMR (128 MHz, $CDCl_3$): δ_B 33.8 ppm.

IR (film): ν_{max} 2976, 2936, 1613, 1509, 1462, 1379, 1307, 1246, 1183, 1138 cm^{-1}.

HRMS (ESI): m/z calc'd for $C_{20}H_{34}{}^{11}BINO_3$ $[M+NH_4]^+$ 474.1675 found 474.1697.

(*R*)-6-Iodo-2-(4-methoxyphenyl)hept-6-en-2-ol (1b')

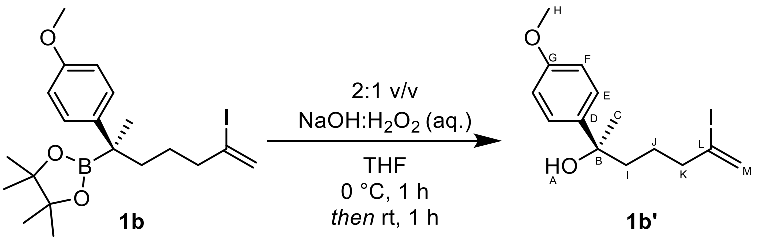

According to General Procedure **D**, vinyl iodide **1b** (23 mg, 0.05 mmol, 1.0 eq.) was reacted with a 2 м aqueous solution of NaOH (0.33 mL) and aqueous H_2O_2 (30% w/w, 0.17 mL) at 0 °C in THF (0.33 mL). The product (14 mg, 83%, 95:5 e.r.) was isolated as a colorless oil after purification by flash column chromatography (pentane/EtOAc 9:1).

R*f* = 0.1 (pentane/EtOAc 9:1).

$[\alpha]_D^{25}$**:** +2 (*c* = 3.6).

NMR Spectroscopy (<u>*see spectra*</u>):

^{1}H NMR (400 MHz, $CDCl_3$): δ_H 7.39–7.30 (m, 2H, H_E), 6.92–6.84 (m, 2H, H_F), 5.97 (apparent q, *J* = 1.4 Hz, 1H, H_M), 5.67 (dt, *J* = 1.4, 0.6 Hz, 1H, $H_{M'}$), 3.81 (s, 3H, H_H), 2.36–2.29 (m, 2H, H_K), 1.81–1.67 (m, 2H, H_I), 1.56 (s, 3H, H_C), 1.53–1.37 (m, 2H, H_J) ppm;

^{13}C NMR (101 MHz, $CDCl_3$): δ_C 158.3 (C_G), 139.9 (C_D), 126.0 (C_E), 125.6 (C_M), 113.5 (C_F), 112.2 (C_L), 74.2 (C_B), 55.3 (C_H), 45.2 (C_K), 42.5 (C_I), 30.2 (C_C), 23.6 (C_J) ppm.

IR (film): ν_{max} 2933, 1608, 1511, 1289, 1246, 1180, 1034 cm^{-1}.

HRMS (ESI): m/z calc'd for $C_{14}H_{19}INaO_2$ $[M+Na]^+$ 369.0322 found 369.0330.

Chiral HPLC (IC column, 95:5 hexane/IPA, 1 mL/min, rt, 230 nm): 10.7 min (major), 13.2 min (minor), 95:5 e.r.

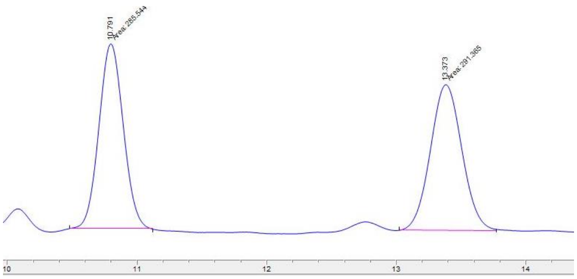

#	Time	Type	Area	Height	Width	Area%	Symmetry
1	10.791	MM	285.5	21.8	0.2179	49.495	0.94
2	13.373	MM	291.4	17.2	0.2821	50.505	0.94

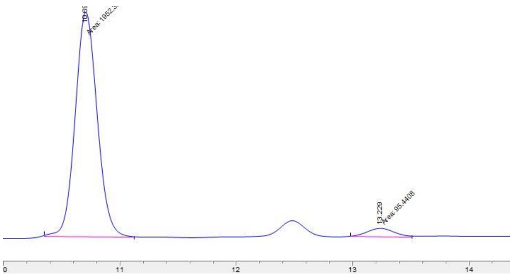

#	Time	Type	Area	Height	Width	Area%	Symmetry
1	10.699	MM	1952.3	148.2	0.2196	95.339	0.904
2	13.229	MM	95.4	5.7	0.2796	4.661	0.888

(*S*)-2-(2-(4-Fluorophenyl)-6-iodohept-6-en-2-yl)-4,4,5,5-tetramethyl-1,3,2-dioxaborolane (1c)

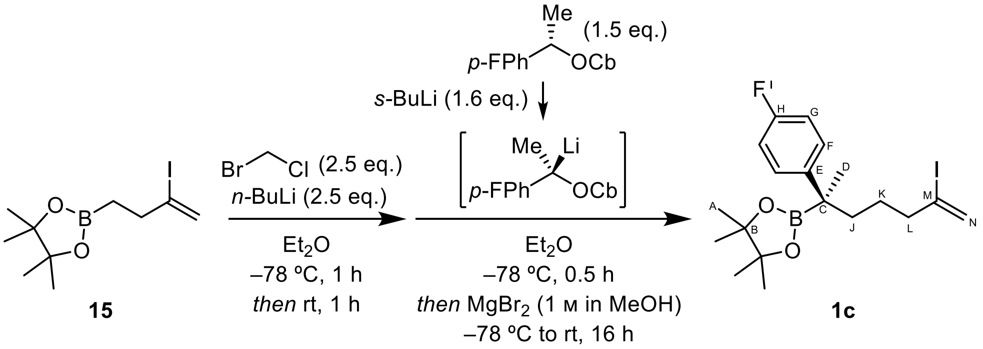

According to <u>General Procedure A</u>, boronic ester **15** (0.462 g, 1.5 mmol, 1.0 eq.) was reacted with bromochloromethane (0.24 mL, 3.8 mmol, 2.5 eq.) and *n*-BuLi (1.6 M in hexanes; 2.3 mL, 3.8 mmol, 2.5 eq.). The crude product was then reacted with (*S*)-1-(4-fluorophenyl)ethyl diisopropylcarbamate[5] (0.602 g, 2.3 mmol, 1.5 eq.) and *s*-BuLi (1.3 M in cyclohexane/hexane 92:8; 1.9 mL, 2.4 mmol, 1.6 eq.). The crude product was purified by flash column chromatography (pentane/Et_2O 50:1) to give the product as a colorless oil (0.403 g, 0.91 mmol, e.r. n.d.) in 61% yield over 2 steps.

The e.r. of boronic ester **1c** could not be determined by chiral HPLC/SFC or by using Pirkle's chiral solvating agent.

R*f* = 0.5 (pentane/Et_2O 95:5).

$[\alpha]_D^{25}$: –3 (c = 5.0).

NMR Spectroscopy (*see spectra*):

^{1}H NMR (400 MHz, $CDCl_3$): δ_H 7.31–7.21 (m, 2H, H_F), 7.01–6.91 (m, 2H, H_G), 5.98 (apparent q, J = 1.4 Hz, 1H, H_N), 5.66 (apparent d, J = 1.5 Hz, 1H, H_N), 2.36 (apparent tt, J = 7.2, 1.6 Hz, 2H, H_L), 1.80-1.69 (m, 1H, H_J), 1.67–1.58 (m, 1H, $H_{J'}$), 1.48–1.36 (m, 2H, H_K), 1.33 (s, 3H, H_D), 1.21 (s, 6H, H_A), 1.21 (s, 6H, $H_{A'}$) ppm;

^{13}C NMR (101 MHz, $CDCl_3$): δ_C 161.2 (d, J = 242.9 Hz, C_H), 142.6 (d, J = 3.0 Hz, C_E), 128.2 (d, J = 7.6 Hz, C_F), 125.3 (C_N), 114.7 (d, J = 20.7 Hz, C_G), 112.5 (C_M), 83.5 (C_B), 45.9 (C_L), 38.2 (C_J), 25.2 (C_K), 24.6 (C_A), 24.6 ($C_{A'}$), 21.8 (C_D) ppm; C_C not observed due to quadrupolar relaxation;

^{11}B NMR (128 MHz, $CDCl_3$): δ_B 33.7 ppm;

^{19}F NMR (377 MHz, $CDCl_3$): δ_F –118.9 ppm.

IR (film): ν_{max} 2977. 2931, 1616, 1508, 1466, 1380, 1350, 1316, 1231, 1141 cm^{-1}.

HRMS (ESI): m/z calc'd for $C_{19}H_{30}{}^{11}BFINO_2$ $[M+NH_4]^+$ 426.1475 found 462.1471.

(*R*)-2-(4-Fluorophenyl)-6-iodohept-6-en-2-ol (1c')

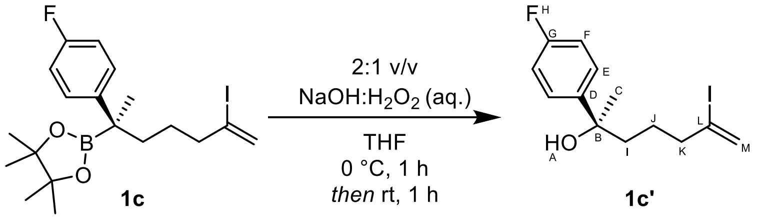

According to General Procedure **D**, vinyl iodide **1c** (22 mg, 0.05 mmol, 1.0 eq.) was reacted with a 2 M aqueous solution of NaOH (0.33 mL) and aqueous H_2O_2 (30% w/w, 0.17 mL) at 0 °C in THF (0.33 mL). The product (15 mg, 87%, e.r. n.d.) was isolated as a colorless oil after purification by flash column chromatography (pentane/EtOAc 9:1).

The e.r. of alcohol **1c'** could not be determined by chiral HPLC/SFC or by using Pirkle's chiral solvating agent.

R*f* = 0.1 (pentane/EtOAc 9:1).

$[\alpha]_D^{25}$: +9 (c = 2.7).

NMR Spectroscopy (*see spectra*):

^{1}H NMR (400 MHz, $CDCl_3$): δ_H 7.46–7.34 (m, 2H, H_E), 7.08–6.96 (m, 2H, H_F), 5.98 (apparent q, J = 1.4 Hz, 1H, H_M), 5.67 (apparent dd, J = 1.4, 0.7 Hz, 1H, $H_{M'}$), 2.35–2.30 (m, 2H, H_K), 1.83–1.66 (m, 2H, H_I), 1.62 (br s, 1H, H_A), 1.57 (s, 3H, H_C), 1.54–1.44 (m, 1H, H_J), 1.42–1.30 (m, 1H, $H_{J'}$) ppm;

^{13}C NMR (101 MHz, $CDCl_3$): δ_C 161.7 (d, J = 244.6 Hz, C_G), 143.4 (d, J = 3.1 Hz, C_D), 126.5 (d, J = 7.9 Hz, C_E), 125.8 (C_M), 114.9 (d, J = 21.1 Hz, C_F), 112.1 (C_L), 74.2 (C_B), 45.1 (C_K), 42.5 (C_I), 30.4 (C_C), 23.4 (C_J) ppm;

^{19}F NMR (377 MHz, $CDCl_3$): δ_F –116.7 ppm.

IR (film): ν_{max} 3415 (br), 2943, 1602, 1510, 1226, 1160 cm^{-1}.

HRMS (ESI): m/z calc'd for $C_{13}H_{16}FINaO$ $[M+Na]^+$ 357.0122 found 357.0105.

(*R*)-2-(1-(4-Iodopent-4-en-1-yl)-2,3-dihydro-1H-inden-1-yl)-4,4,5,5-tetramethyl-1,3,2-dioxaborolane (1d)

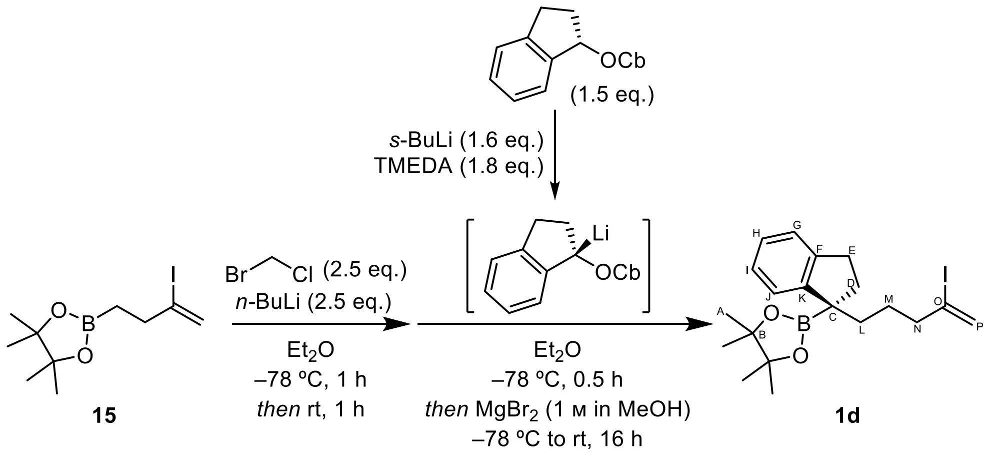

According to General Procedure **B**, boronic ester **15** (0.308 g, 1.0 mmol, 1.0 eq.) was reacted with bromochloromethane (0.16 mL, 2.5 mmol, 2.5 eq.) and *n*-BuLi (1.6 M in hexanes; 1.6 mL, 2.5 mmol, 2.5 eq.). The crude product was then reacted with (*S*)-2,3-dihydro-1H-inden-1-yl diisopropylcarbamate[6] (0.392 g, 1.5 mmol, 1.5 eq.), TMEDA (0.27 mL, 1.8 mmol, 1.8 eq.) and *s*-BuLi (1.3 M in cyclohexane/hexane 92:8; 1.2 mL, 1.6 mmol, 1.6 eq.). The crude product was purified by flash column chromatography (pentane/Et_2O 50:1) to give the product as a colorless solid (0.268 g, 0.61 mmol, 96:4 e.r.) in 61% yield over 2 steps.

The e.r. of boronic ester **1d** could not be determined by chiral HPLC/SFC. Instead, the boronic ester was oxidised and the e.r. of alcohol **1d'** was determined.

R*f* = 0.6 (pentane/Et_2O 95:5).

$[\alpha]_D^{25}$**:** +8 (*c* = 4.8).

NMR Spectroscopy (*see spectra*):

^{1}H NMR (400 MHz, $CDCl_3$): δ_H 7.25–7.20 (m, 1H, H_{Ar}), 7.20-7.16 (m, 1H, H_{Ar}), 7.14–7.05 (m, 2H, H_{Ar}), 6.00 (apparent q, *J* = 1.4 Hz, 1H, H_P), 5.69–5.63 (m, 1H, $H_{P'}$), 2.92 (apparent t, *J* = 7.4 Hz, 2H, H_E), 2.47–2.30 (m, 3H, $H_{D, N}$), 1.94–1.75 (m, 2H, $H_{D', L}$) 1.62–1.46 (m, 2H, H_M), 1.47–1.36 (m, 1H, $H_{L'}$), 1.19 (s, 6H, H_A), 1.17 (s, 6H, $H_{A'}$) ppm;

^{13}C NMR (101 MHz, $CDCl_3$): δ_C 148.8 (C_F), 143.7 (C_K), 125.9 (C_{Ar}), 125.7 (C_{Ar}), 125.3 (C_P), 124.3 (C_{Ar}), 124.1 (C_{Ar}), 112.6 (C_O), 83.3 (C_B), 45.9 (C_N), 36.8 (C_L), 34.0 (C_D), 31.6 (C_E), 26.7 (C_M), 24.7 (C_A) ppm; C_C not observed due to quadrupolar relaxation;

^{11}B NMR (128 MHz, $CDCl_3$): δ_B 33.3 ppm.

IR (film): ν_{max} 2976, 2934, 1616, 1370, 1313, 1263, 1213, 1165, 1141 cm^{-1}.

HRMS (ESI): m/z calc'd for $C_{20}H_{28}{}^{11}BINaO_2$ $[M+Na]^+$ 456.1569 found 456.1584.

(*S*)-1-(4-Iodopent-4-en-1-yl)-2,3-dihydro-*1H*-inden-1-ol (1d')

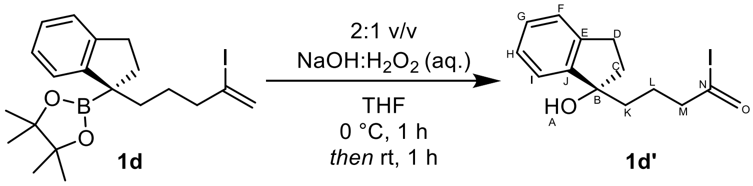

According to General Procedure **D**, vinyl iodide **1d** (22 mg, 0.05 mmol, 1.0 eq.) was reacted with a 2 м aqueous solution of NaOH (0.33 mL) and aqueous H_2O_2 (30% w/w, 0.17 mL) at 0 °C in THF (0.33 mL). The product (11 mg, 70%, 96:4 e.r.) was isolated as a colorless oil after purification by flash column chromatography (pentane/EtOAc 9:1).

R*f* = 0.2 (pentane/EtOAc 9:1).

$[\boldsymbol{\alpha}]_{\boldsymbol{D}}^{\mathbf{25}}$**:** –3 (*c* = 2.9).

NMR Spectroscopy (*see spectra*):

^{1}H NMR (400 MHz, CD_2Cl_2): δ_H 7.35–7.29 (m, 1H, H_{Ar}), 7.27–7.18 (m, 3H, H_{Ar}), 6.03 (apparent q, *J* = 1.4 Hz, 1H, H_O), 5.72–5.66 (m, 1H, $H_{O'}$), 3.00 (ddd, *J* = 16.1, 8.6, 4.9 Hz, 1H, H_D), 2.83 (ddd, *J* = 16.1, 8.2, 6.5 Hz, 1H, $H_{D'}$), 2.50-2.36 (m, 2H, H_M), 2.31 (ddd, *J* = 13.2, 8.2, 4.9 Hz, 1H, H_C), 2.08 (ddd, *J* = 13.2, 8.6, 6.5 Hz, 1H, $H_{C'}$), 1.90-1.79 (m, 1H, H_K), 1.77 (s, 1H, H_A), 1.72–1.61 (m, 2H, $H_{K', L}$), 1.61–1.55 (m, 1H, $H_{L'}$) ppm;

^{13}C NMR (101 MHz, CD_2Cl_2): δ_C 147.6 (C_E *or* C_J), 143.1 (C_E *or* C_J), 128.1 (C_{Ar}), 126.5 (C_{Ar}), 125.5 (C_O), 124.9 (C_{Ar}), 122.7 (C_{Ar}), 112.4 (C_N), 83.3 (C_B), 45.4 (C_M), 40.0 (C_C *or* C_D), 38.7 (C_K), 29.4 (C_C *or* C_D), 23.9 (C_L) ppm.

IR (film): ν_{max} 3372 (br), 2940, 2848, 1620, 1461, 1166, 1034 cm^{-1}.

HRMS (ESI): m/z calc'd for $C_{14}H_{17}INaO$ $[M+Na]^+$ 351.0216 found 351.0219.

Chiral HPLC (IC column, 95:5 hexane/IPA, 1 mL/min, rt, 254 nm): 7.0 min (minor), 7.3 min (major), 96:4 e.r.

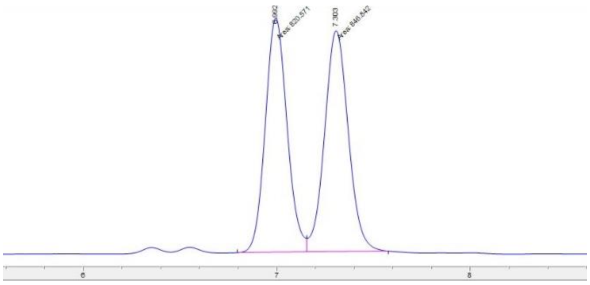

#	Time	Type	Area	Height	Width	Area%	Symmetry
1	6.992	MF	820.6	104.8	0.1305	49.212	0.897
2	7.303	FM	846.8	99	0.1426	50.788	0.914

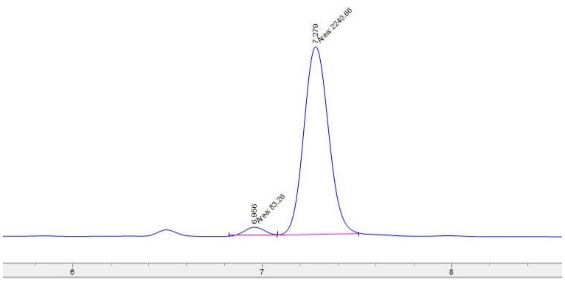

#	Time	Type	Area	Height	Width	Area%	Symmetry
1	6.956	MM	83.3	11.2	0.1237	3.583	0.888
2	7.279	MM	2240.7	246.6	0.1515	96.417	0.896

(*S*)-2-(6-Iodohept-6-en-2-yl)-4,4,5,5-tetramethyl-1,3,2-dioxaborolane (1e)

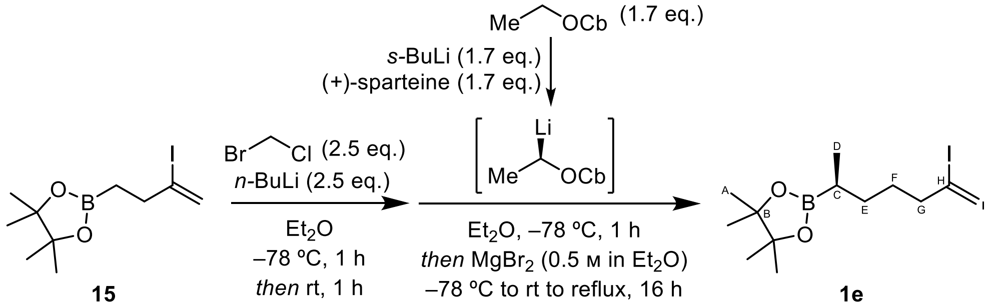

According to General Procedure C, boronic ester **15** (0.308 g, 1.0 mmol, 1.0 eq.) was reacted with bromochloromethane (0.16 mL, 2.5 mmol, 2.5 eq.) and *n*-BuLi (1.6 M in hexanes; 1.6 mL, 2.5 mmol, 2.5 eq.). The crude product was then reacted with ethyl diisopropylcarbamate[9] (0.289 g, 1.7 mmol, 1.7 eq.), (+)-sparteine (380 µL, 1.7 mmol, 1.7 eq.) and *s*-BuLi (1.3 M in cyclohexane/hexane 92:8; 1.3 mL, 1.7 mmol, 1.7 eq.). The crude product was purified by flash column chromatography (pentane/Et_2O 100:1 to 50:1) to give the product as a colorless oil (0.193 g, 0.55 mmol, 98:2 e.r.) in 55% yield over 2 steps.

The e.r. of boronic ester **1e** could not be determined by chiral HPLC/SFC. Instead, the boronic ester was oxidised and the e.r. of alcohol **1e'** was determined.

R*f* = 0.7 (pentane/Et_2O 98:2).

$[\alpha]_D^{25}$**:** +5 (*c* = 5.0).

NMR Spectroscopy (*see spectra*):

¹H NMR (400 MHz, $CDCl_3$): δ_H 6.00 (apparent q, *J* = 1.4 Hz, 1H, H_I), 5.66 (dt, *J* = 1.3, 0.6 Hz, 1H, $H_{I'}$), 2.38 (apparent td, *J* = 7.3, 1.3 Hz, 2H, H_G), 1.58–1.48 (m, 2H, H_F), 1.48–1.38 (m, 1H, H_E), 1.35–1.27 (m, 1H, $H_{E'}$), 1.24 (s, 12H, H_A), 1.06–0.94 (m, 4H, $H_{C,\ D}$) ppm;

¹³C NMR (101 MHz, $CDCl_3$): δ_C 125.1 (C_I), 112.8 (C_H), 82.9 (C_B), 45.6 (C_G), 31.8 (C_E), 28.5 (C_F), 24.8 (C_A), 24.8 ($C_{A'}$), 15.5 (C_D) ppm; C_C not observed due to quadrupolar relaxation;

¹¹B NMR (128 MHz, $CDCl_3$): δ_B 35.5 ppm.

IR (film): ν_{max} 2976, 2929, 2870, 1617, 1460, 1370, 1315, 1144 cm^{-1}.

HRMS (ESI): m/z calc'd for $C_{13}H_{24}{}^{11}BINaO_2$ $[M+Na]^+$ 373.0809 found 373.0806.

(*S*)-6-Iodohept-6-en-2-ol (1e')

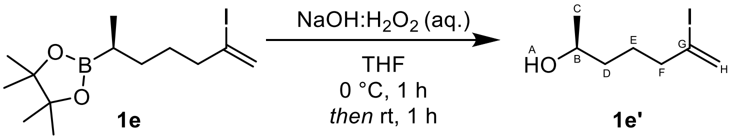

According to General Procedure D, vinyl iodide **1e** (18 mg, 0.05 mmol, 1.0 eq.) was reacted with a 2 M aqueous solution of NaOH (0.33 mL) and aqueous H_2O_2 (30% w/w, 0.17 mL) at 0 °C in THF (0.33 mL). The product (8 mg, 70%, 98:2 e.r.) was isolated as a colorless oil after purification by flash column chromatography

(pentane/EtOAc 9:1).

R*f* = 0.1 (pentane/EtOAc 9:1).

$[\alpha]_D^{25}$**:** +3 (*c* = 2.7).

NMR Spectroscopy (*see spectra*):

^{1}H NMR (400 MHz, $CDCl_3$): δ_H 6.03 (apparent q, *J* = 1.4 Hz, 1H, H_H), 5.70 (apparent dd, *J* = 1.4, 0.7 Hz, 1H, $H_{H'}$), 3.89–3.76 (m, 1H, H_B), 2.41 (apparent td, *J* = 7.2, 1.3 Hz, 2H, H_F), 1.71–1.37 (m, 4H, $H_{D, E}$), 1.21 (d, *J* = 6.2 Hz, 3H, H_C) ppm;

^{13}C NMR (101 MHz, $CDCl_3$): δ_C 125.6 (C_H), 112.1 (C_G), 67.8 (C_B), 45.1 (C_F), 37.6 (C_D), 25.3 (C_E), 23.6 (C_C) ppm.

IR (film): ν_{max} 3362 (br), 2964, 2925, 1616, 1458, 1401, 1373, 1136 cm^{-1}.

HRMS (ESI): m/z calc'd for $C_7H_{13}INaO$ [M+Na]$^+$ 262.9909 found 262.9903.

Chiral HPLC (IB column, 99.5:0.5 hexane/IPA, 1 mL/min, rt, 254 nm): 26.0 min (minor), 26.8 min (major), 98:2 e.r.

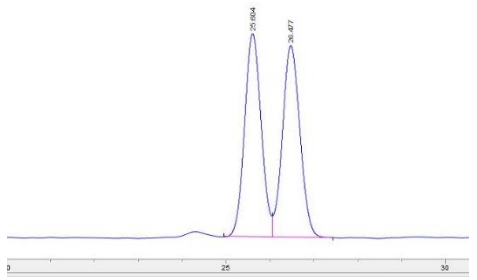

#	Time	Type	Area	Height	Width	Area%	Symmetry
1	25.604	BV	1551	59	0.3903	50.132	0.911
2	26.477	VB	1542.9	55.6	0.4221	49.868	0.894

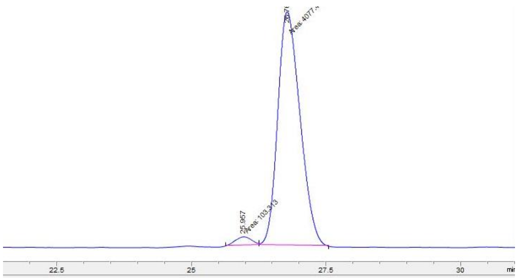

#	Time	Type	Area	Height	Width	Area%	Symmetry
1	25.957	MM	103.3	5	0.3454	2.471	0.997
2	26.764	MM	4077.4	141.8	0.4794	97.529	0.748

(*R*)-2-(7-Iodo-2-methyloct-7-en-3-yl)-4,4,5,5-tetramethyl-1,3,2-dioxaborolane (1f)

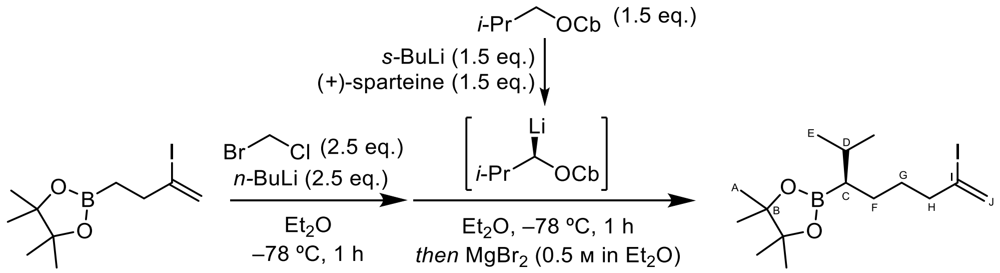

15 **1f**

According to General Procedure C, boronic ester **15** (0.308 g, 1.0 mmol, 1.0 eq.) was reacted with bromochloromethane (0.16 mL, 2.5 mmol, 2.5 eq.) and *n*-BuLi (1.6 M in hexanes; 1.6 mL, 2.5 mmol, 2.5 eq.). The crude product was then reacted with *iso*-butyl diisopropylcarbamate[9] (0.302 g, 1.5 mmol, 1.5 eq.), (+)-sparteine (350 μL, 1.5 mmol, 1.5 eq.) and *s*-BuLi (1.3 M in cyclohexane/hexane 92:8; 1.2 mL, 1.5 mmol, 1.5 eq.). The crude product was purified by flash column chromatography (pentane/Et_2O 100:1) to give the product as a colorless oil (0.147 g, 0.39 mmol, 95:5 e.r.) in 39% yield over 2 steps.

The e.r. of boronic ester **1f** could not be determined by chiral HPLC/SFC. Instead, the boronic ester was oxidised and the e.r. of alcohol **1f’** was determined.

R*f* = 0.6 (pentane/Et_2O 98:2).

$[\alpha]_D^{25}$: +5 (*c* = 5.0).

NMR Spectroscopy (*see spectra*):

^{1}H NMR (400 MHz, $CDCl_3$): δ_H 6.00 (apparent q, *J* = 1.4 Hz, 1H, H_J), 5.66 (apparent dd, *J* = 1.3, 0.6 Hz, 1H, $H_{J'}$), 2.45–2.33 (m, 2H, H_H), 1.70 (hept, *J* = 6.7 Hz, 1H, H_D), 1.57–1.49 (m, 1H, H_G), 1.48–1.31 (m, 3H, $H_{F, F', G'}$), 1.26 (s, 12H, H_A), 0.92 (d, *J* = 6.7 Hz, 3H, H_E), 0.91 (d, *J* = 6.7 Hz, 3H, $H_{E'}$) ppm;

^{13}C NMR (101 MHz, $CDCl_3$): δ_C 125.1 (C_J), 112.8 (C_I), 82.9 (C_B), 45.7 (C_H), 29.6 (C_D), 29.1 (C_G), 27.8 (C_F), 25.0 (C_A), 24.9 ($C_{A'}$), 22.3 (C_E), 21.8 ($C_{E'}$) ppm; C_C not observed due to quadrupolar relaxation;

^{11}B NMR (128 MHz, $CDCl_3$): δ_B 33.6 ppm.

IR (film): ν_{max} 3423, 2960, 2925, 2858, 1617, 1459, 1380, 1315, 1260, 1216, 1144 cm^{-1}.

HRMS (ESI): m/z calc’d for $C_{15}H_{29}O_2{}^{11}BI$ $[M+H]^+$ 379.1300 found 379.1308.

(*R*)-7-Iodo-2-methyloct-7-en-3-ol (1f’)

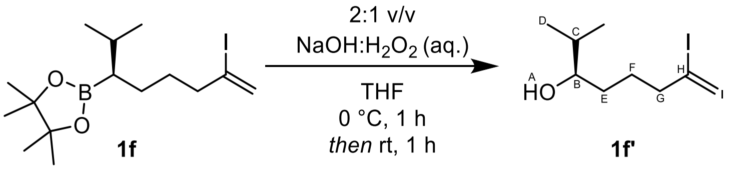

According to General Procedure **D**, vinyl iodide **1f** (19 mg, 0.05 mmol, 1.0 eq.) was reacted with a 2 м aqueous solution of NaOH (0.33 mL) and aqueous H_2O_2 (30% w/w, 0.17 mL) at 0 °C in THF (0.33 mL). The product (12 mg, 90%, 95:5 e.r.) was isolated as a colorless oil after purification by flash column chromatography (pentane/EtOAc 9:1).

R*f* = 0.2 (pentane/EtOAc 9:1).

$[\alpha]_D^{25}$: +9 (*c* = 2.7).

NMR Spectroscopy (*see spectra*):

^{1}H NMR (400 MHz, $CDCl_3$): δ_H 6.03 (apparent q, *J* = 1.4 Hz, 1H, H_I), 5.70 (apparent dd, *J* = 1.4, 0.7 Hz, 1H, $H_{I'}$), 3.38 (ddd, *J* = 8.6, 5.2, 3.5 Hz, 1H, H_B), 2.46–2.38 (m, 2H, H_G), 1.78–1.60 (m, 2H, $H_{C, F}$), 1.60-1.43 (m, 2H, $H_{E, F'}$), 1.43–1.31 (m, 1H, $H_{E'}$), 0.92 (d, *J* = 6.8 Hz, 3H, H_D), 0.91 (d, *J* = 6.8 Hz, 3H, $H_{D'}$) ppm;

^{13}C NMR (101 MHz, $CDCl_3$): δ_C 125.5 (C_I), 112.4 (C_H), 76.4 (C_B), 45.2 (C_G), 33.6 (C_C), 32.6 (C_E), 25.5 (C_F), 18.9 (C_D), 17.1 ($C_{D'}$) ppm.

IR (film): ν_{max} 3374 (br), 2957, 2872, 1616, 1459, 1385, 1261, 1173, 1139 cm^{-1}.

HRMS (ESI): m/z calc’d for $C_9H_{17}INaO$ $[M+Na]^+$ 291.0216 found 291.0208.

Chiral HPLC (IA column, 99.9:0.1 hexane/IPA, 1 mL/min, rt, 254 nm): 27.5 min (minor), 29.2 (major), 95:5 e.r.

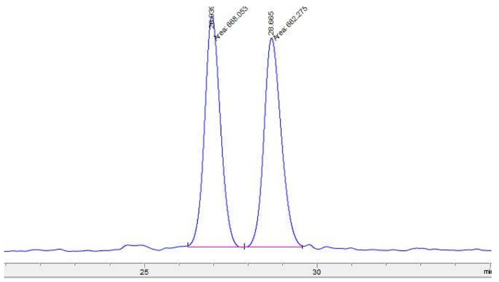

#	Time	Type	Area	Height	Width	Area%	Symmetry
1	26.939	BB	1200.5	37.8	0.4419	50.323	0.847
2	28.664	BB	1185.1	34.2	0.4595	49.677	0.757

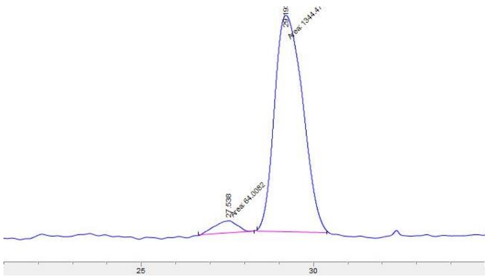

#	Time	Type	Area	Height	Width	Area%	Symmetry
1	27.526	MM	115.4	2.4	0.8099	4.547	1.515
2	29.191	MM	2422.7	42.4	0.9524	95.453	0.676

2-(5-Iodohex-5-en-1-yl)-4,4,5,5-tetramethyl-1,3,2-dioxaborolane (1g)

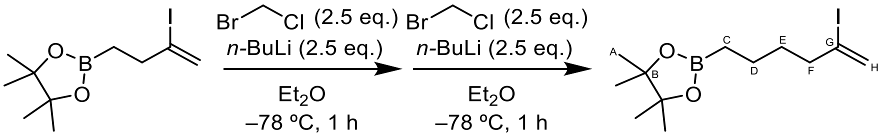

15 **1g**

According to a modified literature procedure,[2] boronic ester **15** (0.616 g, 2.0 mmol, 1.0 eq.) was solved in Et_2O (20 mL) and cooled to –78 °C (dry ice/acetone). Bromochloromethane (0.32 mL, 5.0 mmol, 2.5 eq.) was added dropwise. To the resultant mixture *n*-BuLi (1.6 ᴍ in hexanes; 3.1 mL, 5.0 mmol, 2.5 eq.) was added *via* syringe pump (0.03 mL/min). Following the addition, the reaction mixture was stirred at –78 °C for 1 h and then warmed to rt and stirred at this temperature for 1 h. The reaction mixture was filtered through a silica plug (eluting Et_2O) and concentrated *in vacuo*. The crude product was used without further purification. The above procedure was then repeated to yield the crude twice homologated boronic ester which was purified by flash column chromatography (pentane/CH_2Cl_2 9:1) to give the product as a colorless oil (0.376 g, 1.1 mmol) in 56% yield over 2 steps.

R*f* = 0.4 (pentane/Et_2O 98:2).

NMR Spectroscopy (*see spectra*):

> **^{1}H NMR** (400 MHz, $CDCl_3$): δ_H 6.00 (apparent q, *J* = 1.4 Hz, 1H, H_H), 5.67 (dt, *J* = 1.4, 0.6 Hz, 1H, $H_{H'}$), 2.41–2.33 (m, 2H, H_F), 1.56–1.47 (m, 2H, H_E), 1.47–1.37 (m, 2H, H_D), 1.25 (s, 12H, H_A), 0.79 (t, *J* = 7.7 Hz, 2H, H_C) ppm;
>
> **^{13}C NMR** (101 MHz, $CDCl_3$): δ_C 125.1 (C_H), 112.7 (C_G), 83.0 (C_B), 45.2 (C_F), 31.7 (C_E), 24.9 (C_A), 24.8 ($C_{A'}$), 22.7 (C_D) ppm; C_C not observed due to quadrupolar relaxation;
>
> **^{11}B NMR** (128 MHz, $CDCl_3$): δ_B 33.7 ppm.

IR (film): ν_{max} 2987, 2931, 2862, 1617, 1378, 1320, 1145 cm^{-1}.

HRMS (ESI): m/z calc'd for $C_{12}H_{22}{}^{11}BIO_2Na$ $[M+Na]^+$ 359.0652 found 359.0655.

2.3. Synthesis of Cyclopentyl Boronic Esters

2.3.1. General Procedures

2.3.1.1. General Procedure E: the electrophile-induced ring contraction of a 6-membered cyclic alkenyl boronate complex in THF

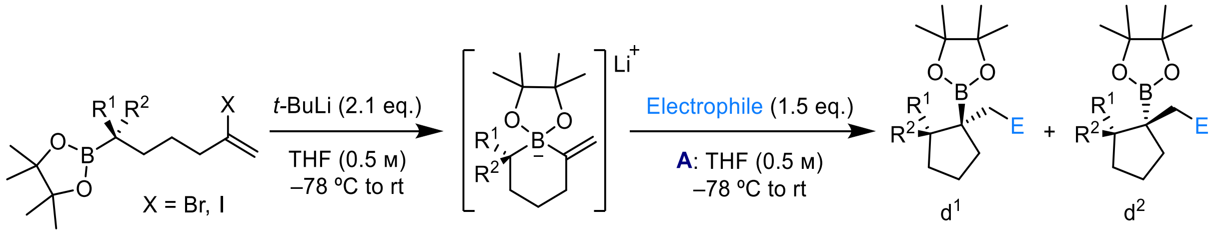

tert-Butyl lithium[a] (in pentane, 0.42 mmol, 2.1 eq.) was added dropwise to a solution of the starting vinyl halide (0.2 mmol, 1.0 eq.) in anhydrous THF (0.4 mL, 0.5 M) at –78 °C (dry ice/acetone) and allowed to stir at this temperature for 1 h. The cooling bath was removed, and the reaction was stirred at rt for a further 1 h before being cooled again to –78 °C (dry ice/acetone) whereupon the electrophile[b] (0.3 mmol, 1.5 eq.) was added. The reaction mixture was stirred at –78 °C for 2 h and slowly warmed to rt overnight (the replenishment of dry ice in the cooling bath was halted). The reaction mixture was diluted with Et_2O (5 mL) and quenched with water[c] (5 mL). The aqueous layer was extracted with Et_2O (3 × 5 mL) and the combined organics washed with brine, dried over $MgSO_4$ and concentrated *in vacuo*. The crude residue was analysed by ^{1}H NMR to determine in what diastereomeric ratio the product was formed. The crude product was then purified by flash column chromatography.

[a] *tert*-Butyl lithium should be titrated before use.[1] [b] Electrophiles sufficiently soluble in THF were added as 1 M solutions in THF, those with partial solubility were quickly added directly into the flask. [c] To be replaced with 0.5 M aqueous NaOH when using Eschenmoser's salt as the electrophile.

2.3.1.2. General Procedure F: the electrophile-induced ring contraction of a 6-membered cyclic alkenyl boronate complex in 1:1 THF/MeCN

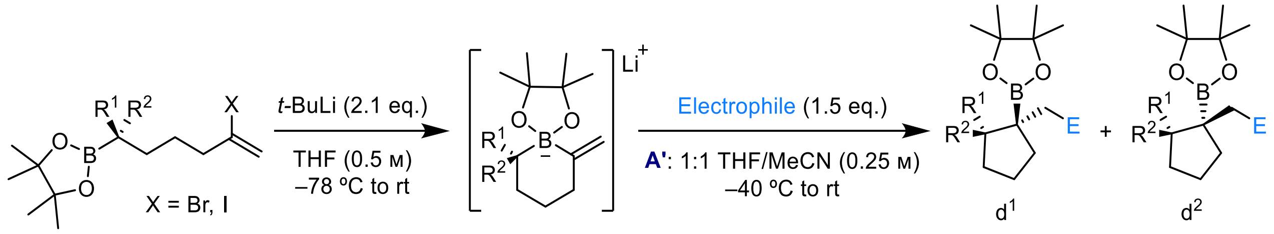

As with General Procedure **E**, but with the addition of MeCN (0.4 mL) to the boronate complex at –40 °C (cryostat) followed by electrophile addition. Stirring at –40 °C for 2 h preceded warming to rt overnight.

2.3.1.3. General Procedure G: the electrophile-induced ring contraction of a 6-membered cyclic alkenyl boronate complex in 1:1 THF/TFE

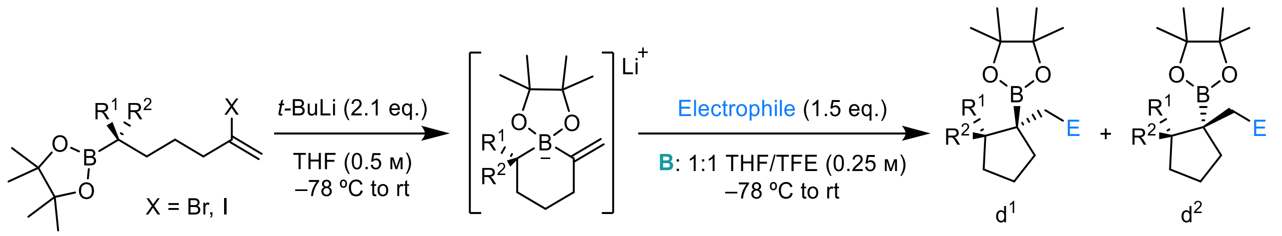

As with General Procedure **E**, but with the addition of TFE (0.4 mL) to the boronate complex at –78 °C (dry ice/acetone) followed by electrophile addition. Stirring at –78 °C for 2 h preceded warming to rt overnight.

2.3.1.4. General Procedure H: the oxidation of cyclopentyl boronic esters

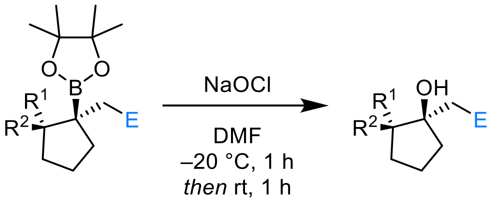

According to a literature procedure,[10] the boronic ester (0.05 mmol, 1.0 eq.) was solved in DMF (0.65 mL) and cooled to –20 °C (cryostat). Aqueous NaOCl (8% available chlorine, 0.14 mL) was added dropwise. The reaction mixture was allowed to stir at this temperature for 1 h followed by at rt for 1 h whereupon it was then diluted with Et_2O (2 mL) and quenched with saturated aqueous $Na_2S_2O_3$ (2 mL). The aqueous layer was extracted with Et_2O (3 × 2 mL) and the combined organics washed with brine, dried over $MgSO_4$ and concentrated *in vacuo*. The crude product was then purified by flash column chromatography.

2.3.2. Substrate Scope

***N,N*-Dimethyl-2-((1*S*,2*S*)-2-methyl-2-phenyl-1-(4,4,5,5-tetramethyl-1,3,2-dioxaborolan-2-yl)cyclopentyl)ethan-1-amine hydrochloride salt (3a.d[1])**

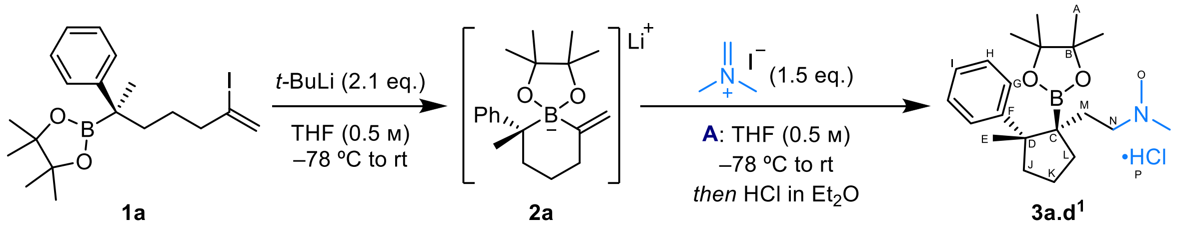

According to General Procedure E, vinyl iodide **1a** (85 mg, 0.2 mmol, 1.0 eq.) was allowed to react with *tert*-butyl lithium (1.6 M in pentane, 0.27 mL, 0.42 mmol, 2.1 eq.) and *N,N*-dimethylmethyleneiminium iodide (56 mg, 0.3 mmol, 1.5 eq.) to give a crude residue (89:11 d.r. by crude ^{1}H NMR) which was dissolved in Et_2O (ca. 0.5 mL) and then 2 N HCl (110 µL, 0.22 mmol, 1.1 eq.) in Et_2O was added. The white precipitate formed was collected *via* suction filtration and dried under high vacuum to afford the boronic ester (57 mg, 73%, 89:11 d^1/d^2, >98:2 e.r., 100% e.s.) as a white foam.

The same procedure was repeated on a 2.35 mmol scale (1.00 g of boronic ester **1a**) to give **3a** (0.721 g, 78%, 90:10 d^1/d^2, >98:2 e.r., 100% e.s.) as a white foam.

R_f = 0.2 for both diastereomers (CH_2Cl_2/MeOH 9:1).

$[\alpha]_D^{25}$: –3 (*c* = 5.7).

NMR Spectroscopy (*see spectra*):

^{1}H NMR (400 MHz, $CDCl_3$): δ_H 12.02 (s, 1H, H_P), 7.37–7.32 (m, 2H, H_G), 7.32–7.25 (m, 2H, H_H), 7.21–7.14 (m, 1H, H_I), 2.90–2.70 (m, 2H, H_N), 2.59 (d, *J* = 4.8 Hz, 3H, H_O), 2.47 (d, *J* = 4.8 Hz, 3H, $H_{O'}$), 2.42–2.30 (m, 1H, H_J), 2.19–2.05 (m, 1H, H_L), 2.04–1.87 (m, 2H, $H_{K, K'}$), 1.87–1.78 (m, 1H, $H_{J'}$), 1.59–1.44 (m, 2H, $H_{L', M}$), 1.36 (s, 3H, H_E), 1.30 (s, 6H, H_A), 1.30 (s, 6H, $H_{A'}$), 1.17–1.06 (m, 1H, $H_{M'}$) ppm;

^{13}C NMR (101 MHz, $CDCl_3$): δ_C 147.2 (C_F), 128.0 (C_H), 127.3 (C_G), 125.9 (C_I), 83.8 (C_B), 55.5 (C_N), 53.5 (C_D), 42.5 (C_O), 41.1 ($C_{O'}$), 37.8 (C_J), 32.5 (C_L), 28.6 (C_E), 27.4 (C_M), 25.2 (C_A), 25.0 ($C_{A'}$), 21.9 (C_K) ppm; C_C not observed due to quadrupolar relaxation;

^{11}B NMR (128 MHz, $CDCl_3$): δ_B 33.6 ppm;

Assignments made for the major diastereoisomer.

IR (film): ν_{max} 3398 (br), 2973, 2596, 1471, 1372, 1313, 1142 cm^{-1}.

HRMS (ESI): m/z calc'd for $C_{22}H_{37}{}^{11}BNO_2$ $[M+H]^+$ 358.2916 found 358.2912.

Chiral solvating agent: The enantiopurity of boronic ester **3a.d[1]** was determined using Pirkle's alcohol ((*R*)-(–)-1-(9-anthryl)-2,2,2-trifluoroethanol) NMR shift reagent (1.0 eq, rt, $CDCl_3$, 0.05 M), >98:2 e.r. A portion of the HCl salt was converted to free amine **17a.d[1]** by washing with 0.5 M NaOH and extracting with Et_2O for e.r. determination.

^{1}H NMR ($CDCl_3$, 500 MHz) expansion of **(±)-17a.d[1]** only:

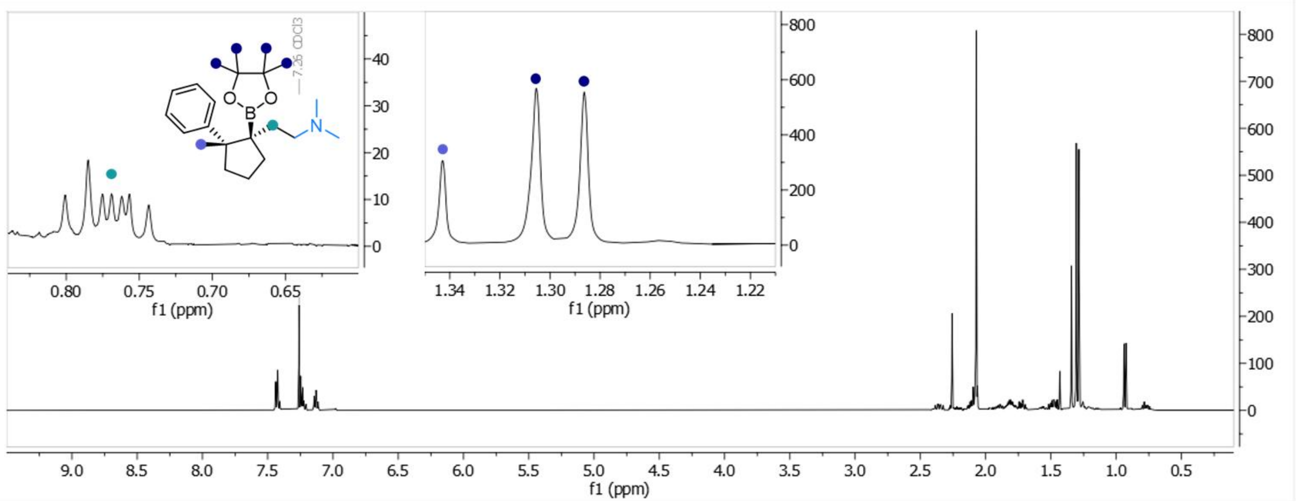

^{1}H NMR ($CDCl_3$, 500 MHz) expansion of **(±)-17a.d[1]** with Pirkle's alcohol:

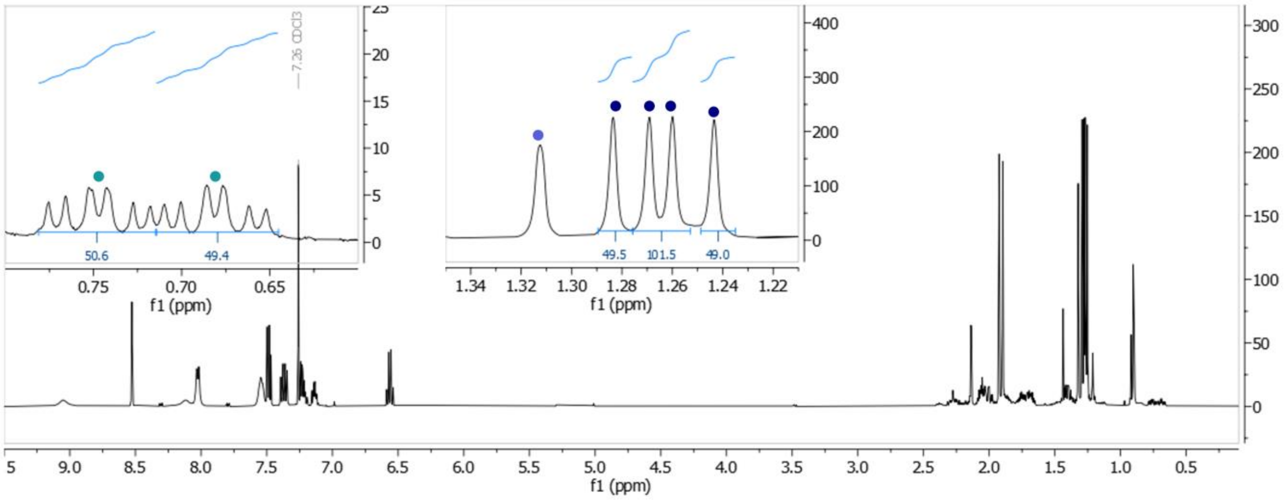

^{1}H NMR ($CDCl_3$, 500 MHz) expansion of **17a.d[1]** with Pirkle's alcohol:

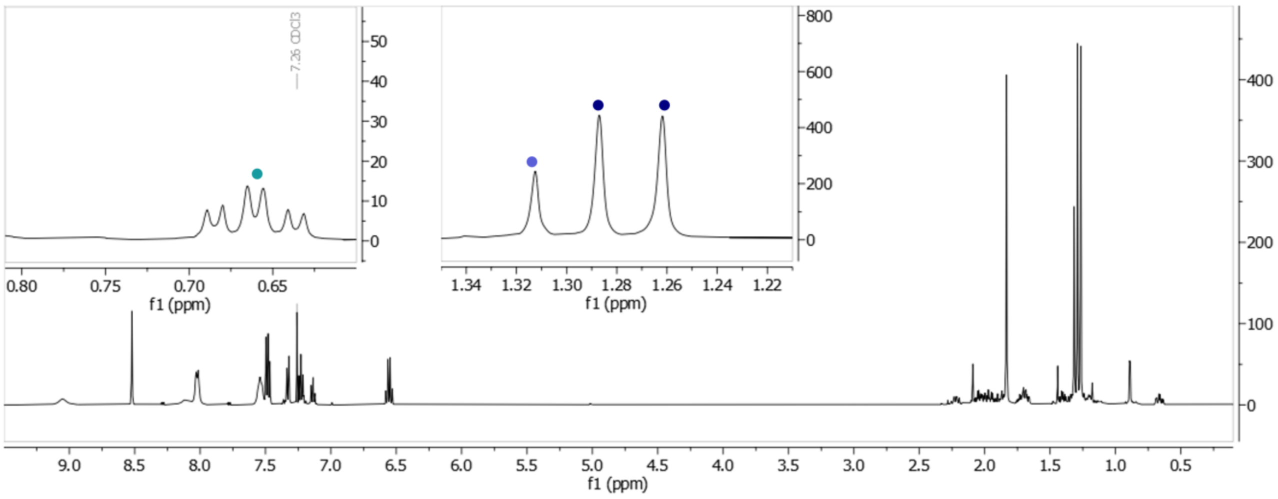

N,N-Dimethyl-2-((1R,2S)-2-methyl-2-phenyl-1-(4,4,5,5-tetramethyl-1,3,2-dioxaborolan-2-yl)cyclopentyl)ethan-1-amine hydrochloride salt (3a.d^2)

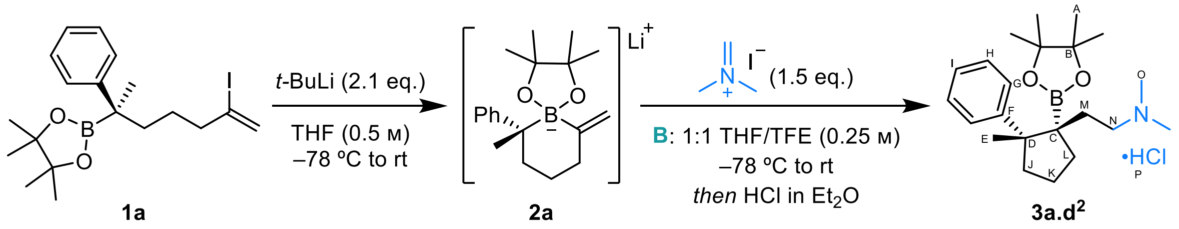

According to General Procedure **G**, vinyl iodide **1a** (85 mg, 0.2 mmol, 1.0 eq.) was allowed to react with *tert*-butyl lithium (1.6 M in pentane, 0.27 mL, 0.42 mmol, 2.1 eq.) and *N,N*-dimethylmethyleneiminium iodide (56 mg, 0.3 mmol, 1.5 eq.) to give a crude residue (86:14 d.r. by crude ^{1}H NMR) which was dissolved in Et_2O (ca. 0.5 mL) and then 2 N HCl (110 µL, 0.22 mmol, 1.1 eq.) in Et_2O was added. The white precipitate formed was collected *via* suction filtration and dried under high vacuum to afford the boronic ester (71 mg, 99%, 14:86 d^1/d^2, >98:2 e.r., 100% e.s.) as a white foam.

R*f* = 0.2 for both diastereomers (CH_2Cl_2/MeOH 9:1).

$[\alpha]_D^{25}$: –2 (*c* = 6.8).

NMR Spectroscopy (*see spectra*):

^{1}H NMR (400 MHz, $CDCl_3$): δ_H 12.05 (s, 1H, H_P), 7.37–7.32 (m, 2H, H_G), 7.23–7.17 (m, 2H, H_H), 7.14–7.07 (m, 1H, H_I), 3.08–2.87 (m, 1H, H_N), 2.77 (s, 3H, H_O), 2.76 (s, 3H, $H_{O'}$), 2.66–2.57 (m, 1H, H_J), 2.27 (td, *J* = 11.7, 4.7 Hz, 1H, H_M), 2.01–1.73 (m, 4H, $H_{K, K', L, M'}$), 1.73–1.60 (m, 1H, $H_{J'}$), 1.60-1.39 (m, 1H, $H_{L'}$), 1.21 (s, 3H, H_E), 0.90 (s, 6H, H_A), 0.82 (s, 6H, $H_{A'}$) ppm;

^{13}C NMR (101 MHz, $CDCl_3$): δ_C 147.8 (C_F), 127.9 (C_H), 126.7 (C_G), 126.0 (C_I), 83.2 (C_B), 56.9 (C_N), 51.1 (C_D), 43.6 (C_O), 41.6 ($C_{O'}$), 38.3 (C_J), 32.8 (C_L), 28.0 (C_M), 24.8 (C_A), 24.7 ($C_{A'}$), 24.5 (C_E), 21.0 (C_K) ppm; C_C not observed due to quadrupolar relaxation;

^{11}B NMR (128 MHz, $CDCl_3$): δ_B 33.6 ppm;

Assignments made for the major diastereoisomer.

IR (film): ν_{max} 3405 (br), 2968, 2660, 1474, 1372, 1316, 1142 cm^{-1}.

HRMS (ESI): m/z calc'd for $C_{22}H_{37}{}^{11}BNO_2$ [M+H]$^+$ 358.2912 found 358.2908.

Chiral solvating agent: The enantiopurity of boronic ester **3a.d^2** was determined using Pirkle's alcohol ((*R*)-(–)-1-(9-anthryl)-2,2,2-trifluoroethanol) NMR shift reagent (1.0 eq, rt, $CDCl_3$, 0.05 M), >98:2 e.r. A portion of the HCl salt was converted to free amine **17a.d^2** by washing with 0.5 M NaOH and extracting with Et_2O for e.r. determination.

^{1}H NMR ($CDCl_3$, 500 MHz) expansion of **(±)-17a.d[2]** only:

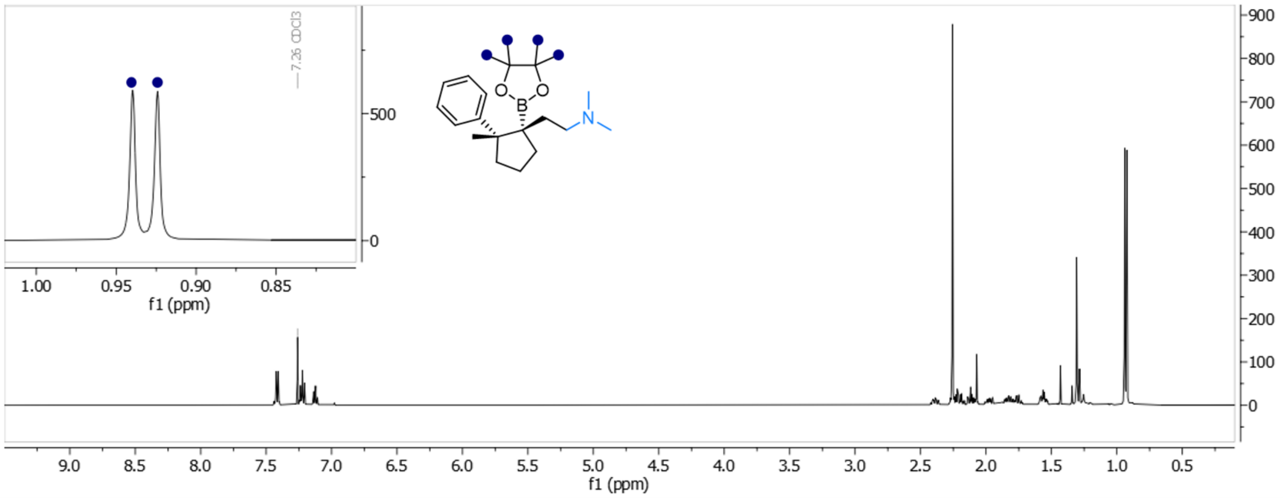

^{1}H NMR ($CDCl_3$, 500 MHz) expansion of **(±)-17a.d[2]** with Pirkle's alcohol:

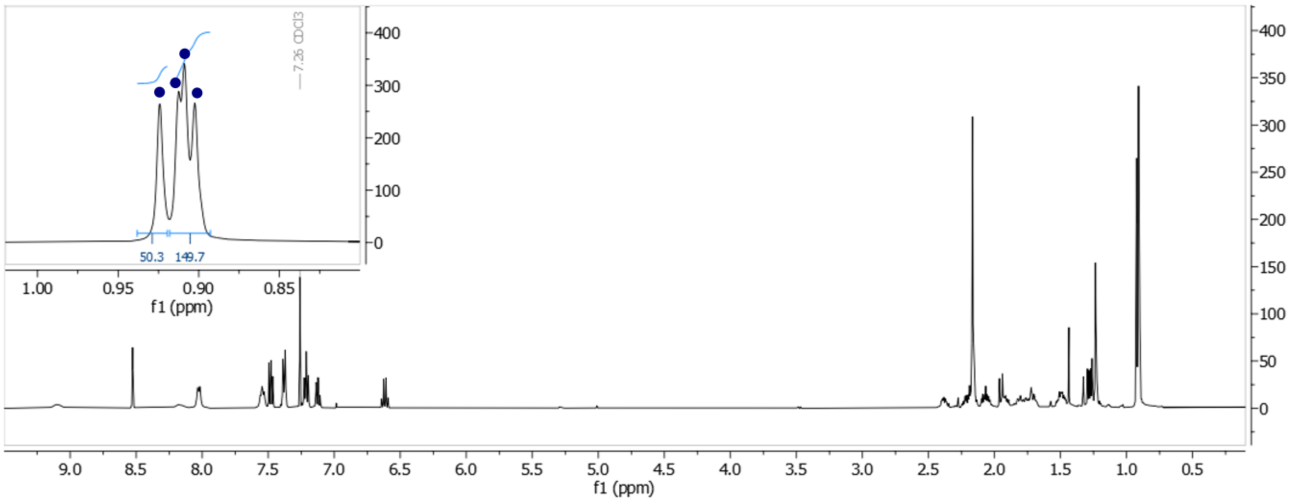

^{1}H NMR ($CDCl_3$, 500 MHz) expansion of **17a.d[2]** with Pirkle's alcohol:

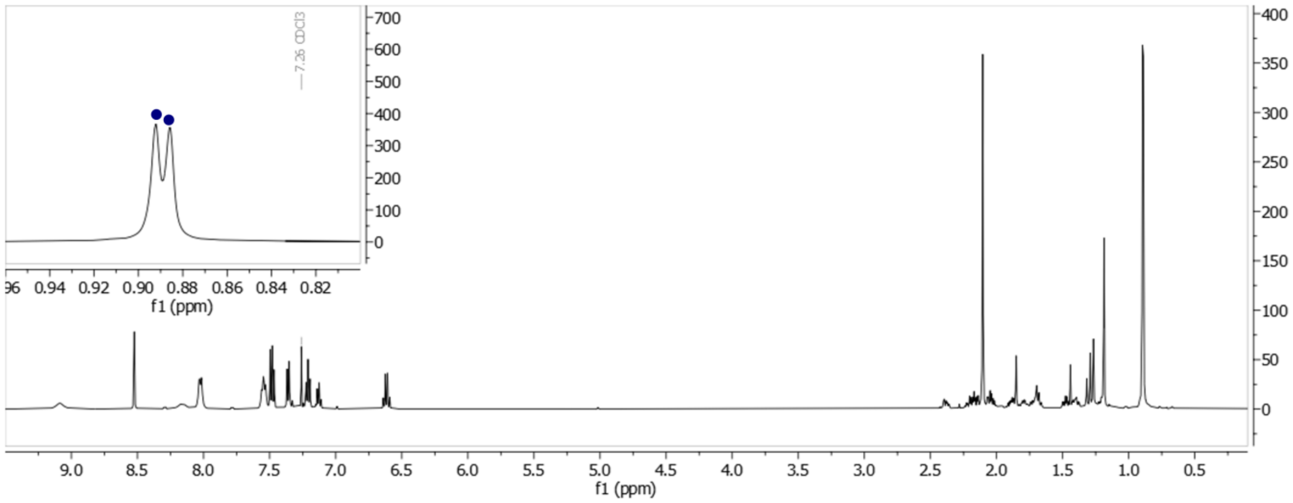

2-((1*S*,2*S*)-2-(4-Methoxyphenyl)-2-methyl-1-(4,4,5,5-tetramethyl-1,3,2-dioxaborolan-2-yl)cyclopentyl)-*N,N*-dimethylethan-1-amine hydrochloride salt (3b.d[1])

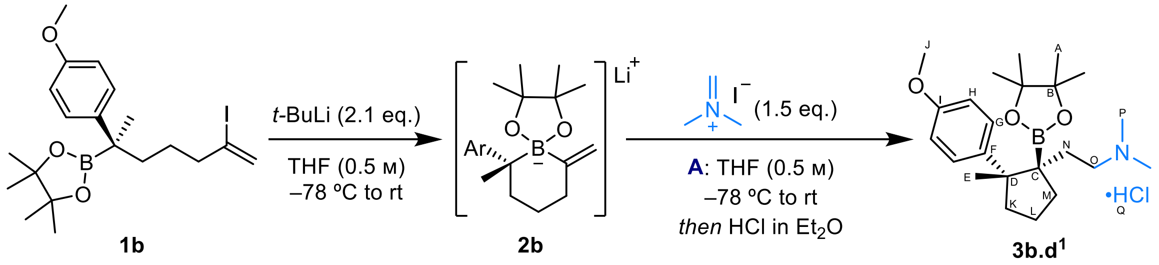

According to General Procedure **E**, vinyl iodide **1b** (91 mg, 0.2 mmol, 1.0 eq.) was allowed to react with *tert*-butyl lithium (1.7 M in pentane, 0.25 mL, 0.42 mmol, 2.1 eq.) and *N,N*-dimethylmethyleneiminium iodide (56 mg, 0.3 mmol, 1.5 eq.) to give a crude residue which was dissolved in Et_2O (ca. 0.5 mL) and then 2 N HCl (110 µL, 0.22 mmol, 1.1 eq.) in Et_2O was added. The white precipitate formed was collected *via* suction filtration and dried under high vacuum to afford the boronic ester (83 mg, 98%, 94:6 d^1/d^2, 95:5 e.r., 100% e.s.) as a white foam.

R*f* = 0.6 for both diastereomers (CH_2Cl_2/MeOH 9:1).

$[\alpha]_D^{25}$: –1 (*c* = 6.1).

NMR Spectroscopy (*see spectra*):

^{1}H NMR (400 MHz, $CDCl_3$): δ_H 11.98 (s, 1H, H_Q), 7.24–7.19 (m, 2H, H_G), 6.80–6.74 (m, 2H, H_H), 3.75 (s, 3H, H_J), 2.87–2.67 (m, 2H, H_O) 2.56 (d, *J* = 4.9 Hz, 3H, H_P), 2.45 (d, *J* = 4.9 Hz, 3H, $H_{P'}$), 2.33–2.20 (m, 1H, H_K), 2.05 (ddd, *J* = 13.1, 9.5, 5.3 Hz, 1H, H_M), 1.98–1.81 (m, 2H, $H_{L, L'}$), 1.75 (ddd, *J* = 12.6, 9.2, 3.8 Hz, 1H, $H_{K'}$), 1.50 (td, *J* = 12.7, 5.4 Hz, 1H, H_N), 1.46–1.39 (m, 1H, $H_{M'}$), 1.29 (s, 3H, H_E), 1.25 (s, 6H, H_A), 1.24 (s, 6H, $H_{A'}$), 1.07 (td, *J* = 12.6, 4.8 Hz, 1H, $H_{N'}$) ppm;

^{13}C NMR (101 MHz, $CDCl_3$): δ_C 157.5 (C_I), 139.1 (C_F), 128.3 (C_G), 113.2 (C_H), 83.8 (C_B), 55.6 (C_O), 55.2 (C_J), 52.8 (C_D), 42.5 (C_P), 41.1 ($C_{P'}$), 38.0 (C_K), 32.5 (C_M), 28.6 (C_E), 27.5 (C_N), 25.2 (C_A), 25.0 ($C_{A'}$), 22.0 (C_L) ppm; C_C not observed due to quadrupolar relaxation;

^{11}B NMR (128 MHz, $CDCl_3$): δ_B 33.2 ppm;

Assignments made for the major diastereoisomer.

IR (film): ν_{max} 3406 (br), 2967, 2645, 1610, 1513, 1468, 1371, 1312, 1253, 1141 cm^{-1}.

HRMS (ESI): m/z calc'd for $C_{23}H_{39}{}^{11}BNO_3$ $[M+H]^+$ 388.3022 found 388.3032.

Chiral solvating agent: The enantiopurity of boronic ester **3b.d[1]** was determined using Pirkle's alcohol ((*R*)-(–)-1-(9-anthryl)-2,2,2-trifluoroethanol) NMR shift reagent (2.0 eq, rt, $CDCl_3$, 0.03 M), 95:5 e.r. A portion of the HCl salt was converted to free amine **17b.d[1]** by washing with 0.5 M NaOH and extracting with Et_2O for e.r. determination.

^{1}H NMR ($CDCl_3$, 500 MHz) expansion of **(±)-17b.d^1** only:

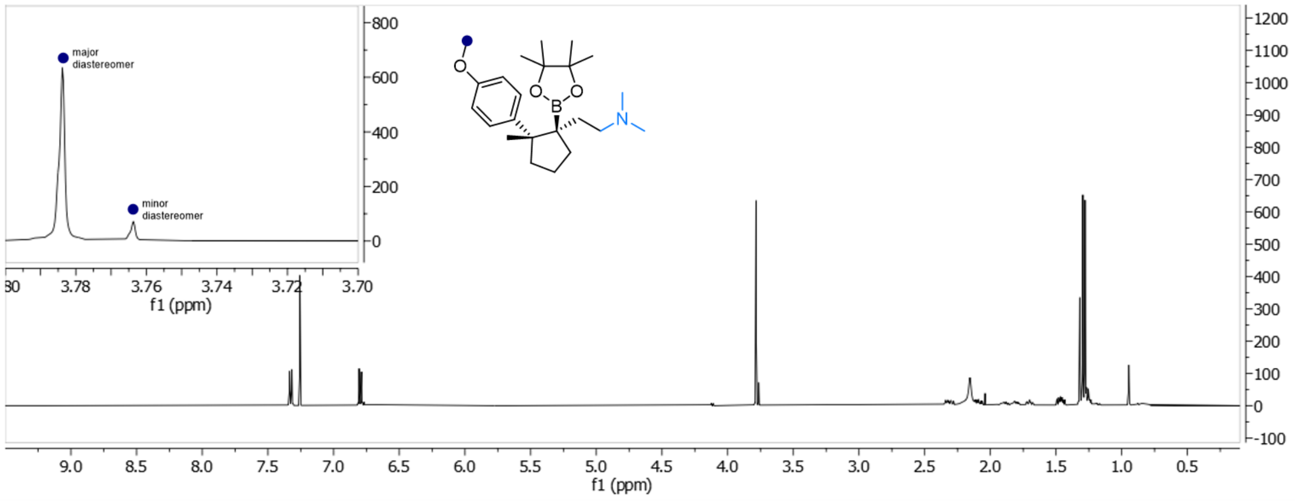

^{1}H NMR ($CDCl_3$, 500 MHz) expansion of **(±)-17b.d^1** with Pirkle's alcohol:

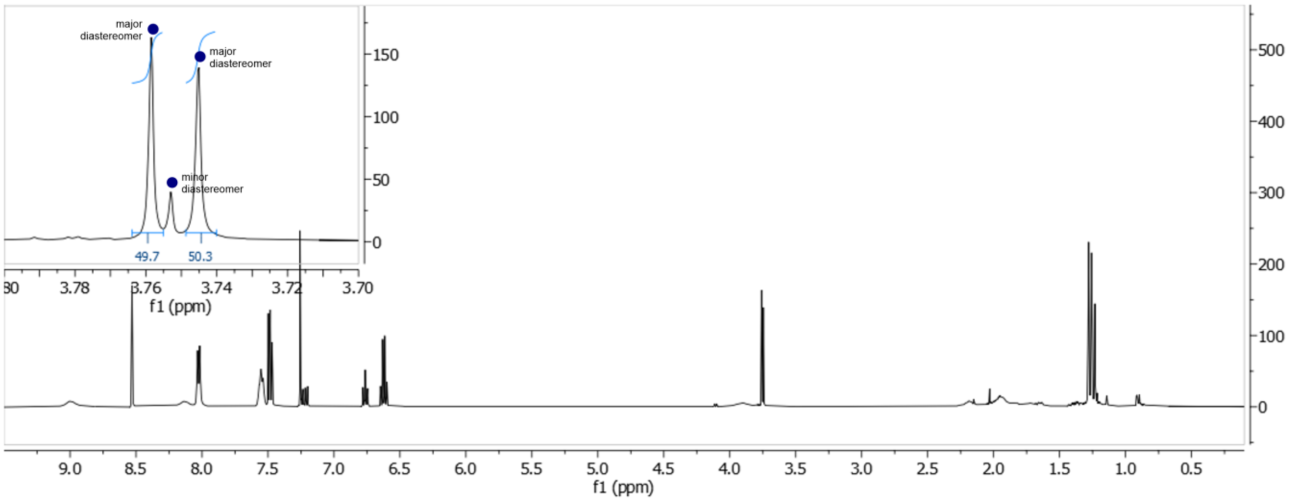

^{1}H NMR ($CDCl_3$, 500 MHz) expansion of **17b.d^1** with Pirkle's alcohol:

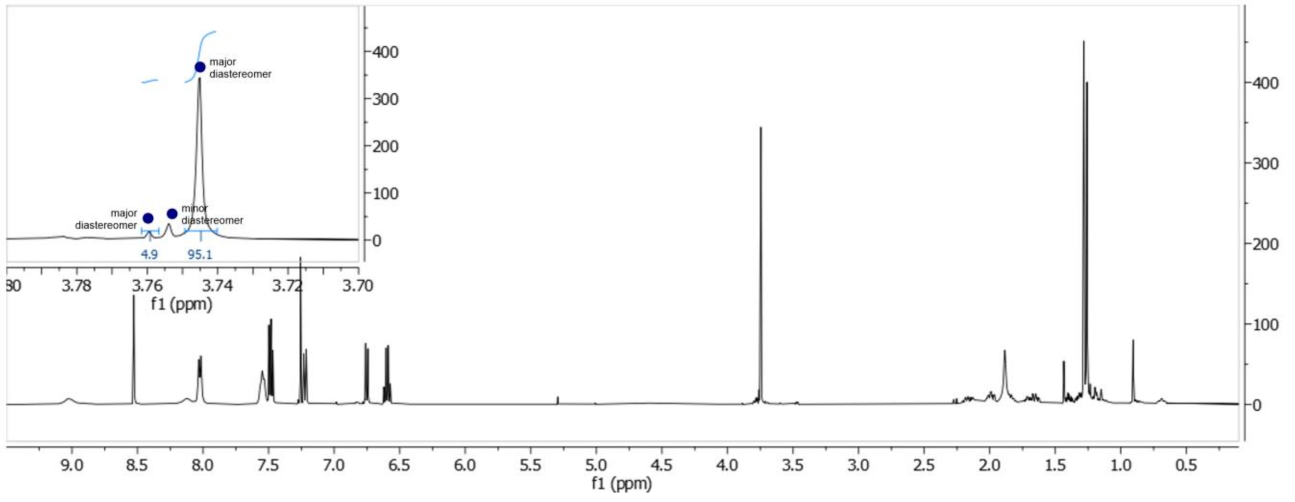

2-((1*R*,2*S*)-2-(4-Methoxyphenyl)-2-methyl-1-(4,4,5,5-tetramethyl-1,3,2-dioxaborolan-2-yl)cyclopentyl)-*N,N*-dimethylethan-1-amine hydrochloride salt (3b.d^2)

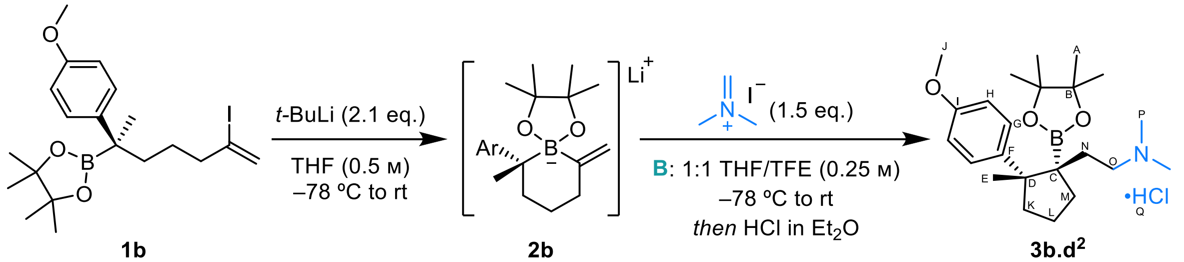

According to General Procedure **G**, vinyl iodide **1b** (91 mg, 0.2 mmol, 1.0 eq.) was allowed to react with *tert*-butyl lithium (1.7 M in pentane, 0.25 mL, 0.42 mmol, 2.1 eq.) and *N,N*-dimethylmethyleneiminium iodide (56 mg, 0.3 mmol, 1.5 eq.) to give a crude residue which was dissolved in Et_2O (ca. 0.5 mL) and then 2 N HCl (110 µL, 0.22 mmol, 1.1 eq.) in Et_2O was added. The white precipitate formed was collected *via* suction filtration and dried under high vacuum to afford the boronic ester (82 mg, 96%, 11:89 d^1/d^2, 95:5 e.r., 100% e.s.) as a white foam.

R*f* = 0.6 for both diastereomers (CH_2Cl_2/MeOH 9:1).

$[\alpha]_D^{25}$: –2 (*c* = 6.0).

NMR Spectroscopy (*see spectra*):

^{1}H NMR (400 MHz, $CDCl_3$): δ_H 12.15 (s, 1H, H_Q), 7.29–7.23 (m, 2H, H_G), 6.76–6.71 (m, 2H, H_H), 3.71 (s, 3H, H_J), 3.05–2.87 (m, 2H, H_O), 2.76 (d, *J* = 4.8 Hz, 6H, H_P), 2.62–2.51 (m, 1H, H_K), 2.28–2.19 (m, 1H, H_N), 1.98–1.69 (m, 4H, $H_{L, L', M, N'}$), 1.64 (ddd, *J* = 12.0, 7.4, 2.6 Hz, 1H, $H_{K'}$), 1.51 (ddd, *J* = 11.1, 9.2, 5.0 Hz, 1H, $H_{M'}$), 1.18 (s, 3H, H_E), 0.92 (s, 6H, H_A), 0.85 (s, 6H, $H_{A'}$) ppm;

^{13}C NMR (101 MHz, $CDCl_3$): δ_C 157.8 (C_I), 140.0 (C_F), 127.8 (C_G), 113.2 (C_H), 83.1 (C_B), 56.9 (C_O), 55.3 (C_J), 50.4 (C_D), 43.5 (C_P), 41.7 ($C_{P'}$), 38.5 (C_K), 32.8 (C_M), 28.0 (C_N), 24.8 (C_A), 24.8 ($C_{A'}$), 24.5 (C_E), 21.0 (C_L) ppm; C_C not observed due to quadrupolar relaxation;

^{11}B NMR (128 MHz, $CDCl_3$): δ_B 33.2 ppm;

Assignments made for the major diastereoisomer.

IR (film): ν_{max} 3406 (br), 2967, 2645, 1610, 1513, 1468, 1371, 1312, 1253, 1187, 1141 cm^{-1}.

HRMS (ESI): m/z calc'd for $C_{23}H_{39}{}^{11}BNO_3$ $[M+H]^+$ 388.3018 found 388.3013.

Chiral solvating agent: The enantiopurity of boronic ester **3b.d^2** was determined using Pirkle's alcohol ((*R*)-(–)-1-(9-anthryl)-2,2,2-trifluoroethanol) NMR shift reagent (3.0 eq, rt, $CDCl_3$, 0.04 M), 95:5 e.r. A portion of the HCl salt was converted to free amine **17b.d^2** by washing with 0.5 M NaOH and extracting with Et_2O for e.r. determination.

^{1}H NMR ($CDCl_3$, 500 MHz) expansion of **(±)-17b.d²** only:

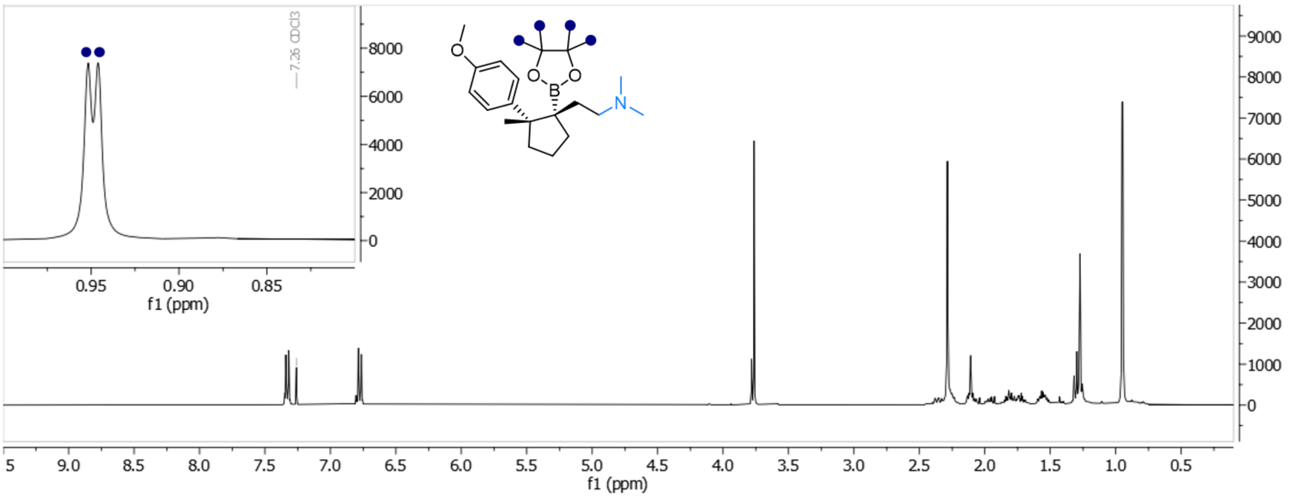

^{1}H NMR ($CDCl_3$, 500 MHz) expansion of **(±)-17b.d²** with Pirkle's alcohol:

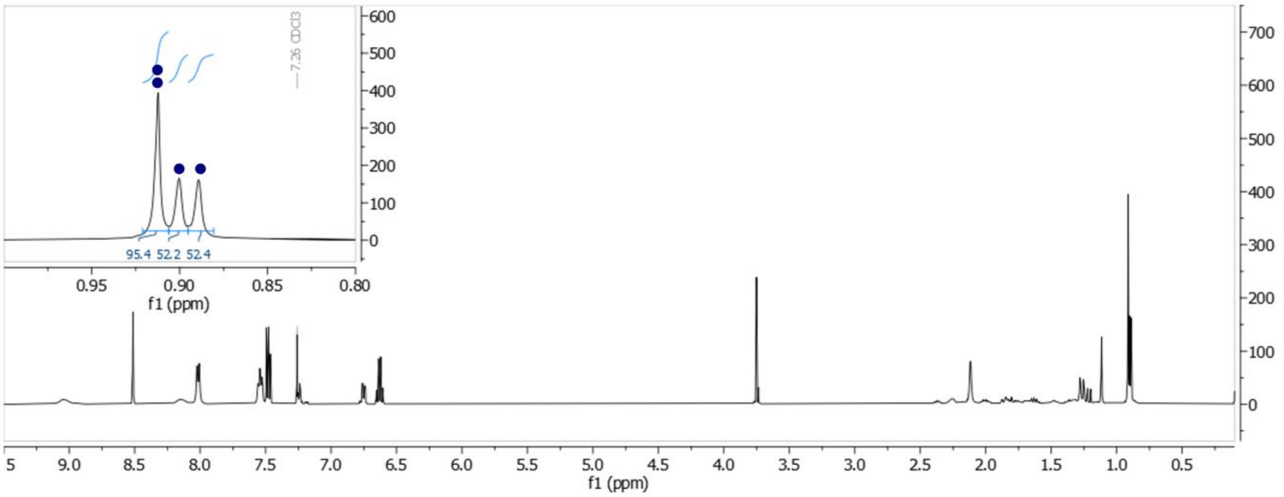

^{1}H NMR ($CDCl_3$, 500 MHz) expansion of **17b.d²** with Pirkle's alcohol:

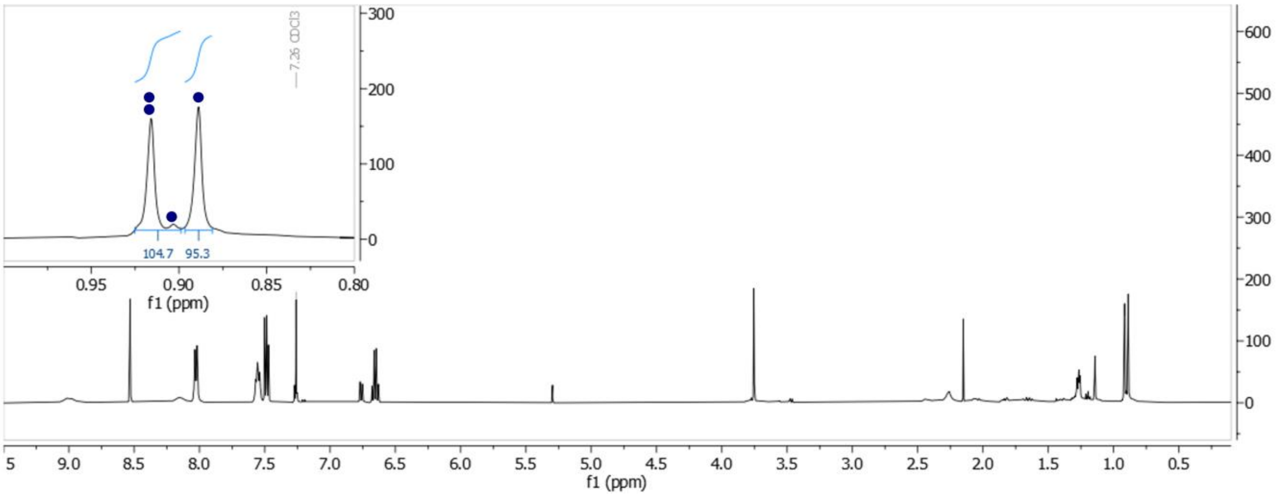

2-((1*S*,2*S*)-2-(4-Fluorophenyl)-2-methyl-1-(4,4,5,5-tetramethyl-1,3,2-dioxaborolan-2-yl)cyclopentyl)-*N,N*-dimethylethan-1-amine hydrochloride salt (3c.d[1])

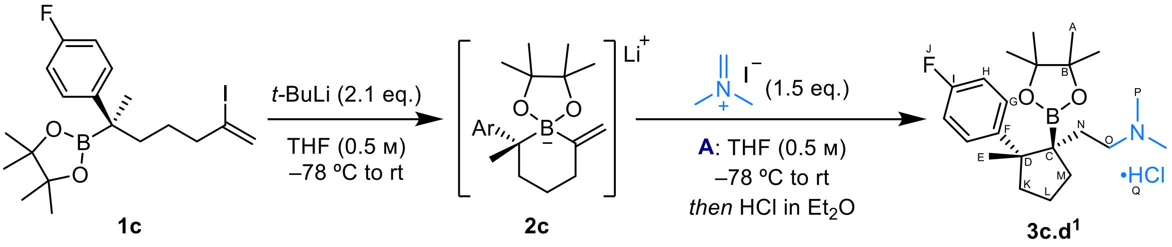

According to General Procedure **E**, vinyl iodide **1c** (89 mg, 0.2 mmol, 1.0 eq.) was allowed to react with *tert*-butyl lithium (1.7 M in pentane, 0.25 mL, 0.42 mmol, 2.1 eq.) and *N,N*-dimethylmethyleneiminium iodide (56 mg, 0.3 mmol, 1.5 eq.) to give a crude residue which was dissolved in Et_2O (ca. 0.5 mL) and then 2 N HCl (110 µL, 0.22 mmol, 1.1 eq.) in Et_2O was added. The white precipitate formed was collected *via* suction filtration and dried under high vacuum to afford the boronic ester (74 mg, 90%, 80:20 d^1/d^2, e.r. n.d.) as a white foam.

The e.r. of boronic ester **3c.d[1]** could not be determined by chiral HPLC/SFC or by using Pirkle's chiral solvating agent.

R*f* = 0.6 for both diastereomers (CH_2Cl_2/MeOH 9:1).

$[\alpha]_D^{25}$**:** –3 (*c* = 6.1).

NMR Spectroscopy (*see spectra*):

^{1}H NMR (400 MHz, $CDCl_3$): δ_H 12.05 (s, 1H, H_Q), 7.37–7.20 (m, 2H, H_G), 6.98–6.85 (m, 2H, H_H), 2.85–2.68 (m, 2H, H_O), 2.58 (d, *J* = 5.0 Hz, 3H, H_P), 2.47 (d, *J* = 5.0 Hz, 3H, $H_{P'}$), 2.34–2.18 (m, 1H, H_K), 2.07 (ddd, *J* = 13.3, 9.3, 5.7 Hz, 1H, H_M), 1.98–1.82 (m, 2H, $H_{L, L'}$), 1.76 (ddd, *J* = 12.7, 8.9, 3.9 Hz, 1H, $H_{K'}$), 1.54–1.40 (m, 2H, $H_{M', N}$), 1.28 (s, 3H, H_E), 1.25 (s, 6H, H_A), 1.25 (s, 6H, $H_{A'}$), 1.13–1.02 (m, 1H, $H_{N'}$) ppm;

^{13}C NMR (101 MHz, $CDCl_3$): δ_C 161.0 (d, *J* = 244.7 Hz, H_I), 142.9 (d, *J* = 3.3 Hz, H_F), 128.9 (d, *J* = 7.5 Hz, H_G), 114.6 (d, *J* = 20.7 Hz, H_H), 83.9 (C_B), 55.7 (C_O), 53.1 (C_D), 42.7 (C_P), 41.2 ($C_{P'}$), 37.9 (C_K), 32.2 (C_M), 28.7 (C_E), 27.6 (C_N), 25.2 (C_A), 24.9 ($C_{A'}$), 21.8 (C_L) ppm; C_C not observed due to quadrupolar relaxation;

^{11}B NMR (128 MHz, $CDCl_3$): δ_B 33.6 ppm;

^{19}F NMR (377 MHz, $CDCl_3$): δ_F –117.8 ppm;

Assignments made for the major diastereoisomer.

IR (film): ν_{max} 3402 (br), 2968, 2633, 1510, 1472, 1368, 1314, 1166, 1229, 1166, 1141 cm^{-1}.

HRMS (ESI): m/z calc'd for $C_{22}H_{36}{}^{11}BFNO_2$ $[M+H]^+$ 376.2822 found 376.2821.

2-((1*R*,2*S*)-2-(4-Fluorophenyl)-2-methyl-1-(4,4,5,5-tetramethyl-1,3,2-dioxaborolan-2-yl)cyclopentyl)-*N*,*N*-dimethylethan-1-amine hydrochloride salt (3c.d^2)

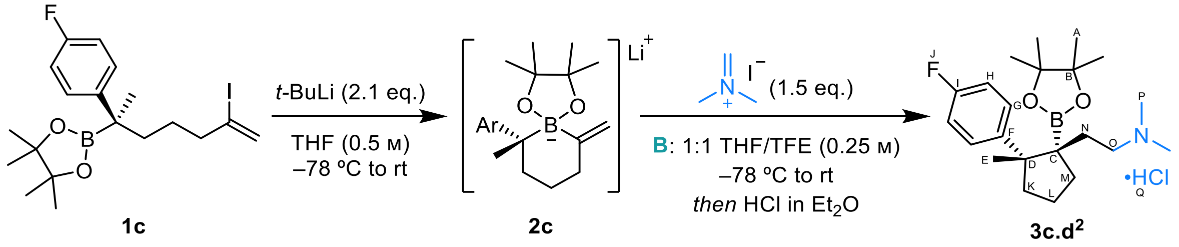

According to General Procedure G, vinyl iodide **1c** (89 mg, 0.2 mmol, 1.0 eq.) was allowed to react with *tert*-butyl lithium (1.7 M in pentane, 0.25 mL, 0.42 mmol, 2.1 eq.) and *N*,*N*-dimethylmethyleneiminium iodide (56 mg, 0.3 mmol, 1.5 eq.) to give a crude residue which was dissolved in Et_2O (ca. 0.5 mL) and then 2 N HCl (110 µL, 0.22 mmol, 1.1 eq.) in Et_2O was added. The white precipitate formed was collected *via* suction filtration and dried under high vacuum to afford the boronic ester (79 mg, 96%, 7:93 d^1/d^2, e.r. n.d.) as a white foam.

The e.r. of boronic ester **3c.d^2** could not be determined by chiral HPLC/SFC or by using Pirkle's chiral solvating agent.

R*f* = 0.6 for both diastereomers (CH_2Cl_2/MeOH 9:1).

$[\alpha]_D^{25}$: –4 (*c* = 5.9).

NMR Spectroscopy (*see spectra*):

^{1}H NMR (400 MHz, $CDCl_3$): δ_H 7.34–7.27 (m, 2H, H_G), 6.92–6.84 (m, 2H, H_H), 2.96–2.79 (m, 2H, H_O), 2.71 (s, 6H, H_P), 2.59–2.47 (m, 1H, H_K), 2.21 (td, *J* = 11.8, 5.0 Hz, 1H, H_N), 1.98–1.83 (m, 2H, $H_{M, N'}$), 1.83–1.70 (m, 2H, $H_{L, L'}$), 1.66 (ddd, *J* = 12.0, 7.4, 2.8 Hz, 1H, $H_{K'}$), 1.53 (ddd, *J* = 11.6, 9.5, 5.5 Hz, 1H, $H_{M'}$), 1.19 (s, 3H, H_E), 0.91 (s, 6H, H_A), 0.85 (s, 6H, $H_{A'}$) ppm;

^{13}C NMR (101 MHz, $CDCl_3$): δ_C 161.2 (d, *J* = 244.4 Hz, C_I), 143.8 (d, *J* = 3.3 Hz, C_F), 128.3 (d, *J* = 7.5 Hz, C_G) 114.4 (d, *J* = 20.5 Hz, C_H), 83.2 (C_B), 57.0 (C_O), 50.6 (C_D), 42.9 (C_P), 38.6 (C_K), 32.7 (C_M), 28.4 (C_N), 24.8 (C_A), 24.7 ($C_{A'}$), 24.7 (C_E), 20.9 (C_L) ppm; C_C not observed due to quadrupolar relaxation;

^{11}B NMR (128 MHz, $CDCl_3$): δ_B 33.6 ppm;

^{19}F NMR (377 MHz, $CDCl_3$): δ_F –117.6 ppm;

Assignments made for the major diastereoisomer.

IR (film): ν_{max} 3401 (br), 2968, 2657, 1602, 1510, 1475, 1372, 1317, 1229, 1165, 1141 cm^{-1}.

HRMS (ESI): m/z calc'd for $C_{22}H_{36}{}^{11}BFNO_2$ $[M+H]^+$ 376.2818 found 376.2814.

N,N-dimethyl-2-((1R,2R)-2-(4,4,5,5-tetramethyl-1,3,2-dioxaborolan-2-yl)-2',3'-dihydrospiro[cyclopentane-1,1'-inden]-2-yl)ethan-1-amine hydrochloride salt (3d.d²)

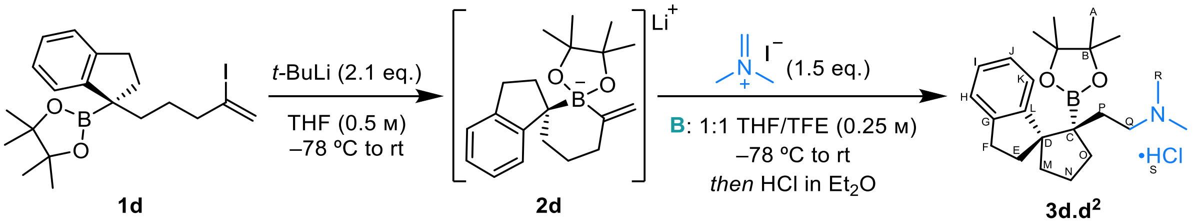

According to General Procedure **G**, vinyl iodide **1d** (88 mg, 0.2 mmol, 1.0 eq.) was allowed to react with *tert*-butyl lithium (1.7 M in pentane, 0.25 mL, 0.42 mmol, 2.1 eq.) and *N,N*-dimethylmethyleneiminium iodide (56 mg, 0.3 mmol, 1.5 eq.) to give a crude residue (>95:5 d.r. by crude ^{1}H NMR) which was dissolved in Et_2O (ca. 0.5 mL) and then 2 N HCl (110 µL, 0.22 mmol, 1.1 eq.) in Et_2O was added. The white precipitate formed was collected *via* suction filtration and dried under high vacuum to afford the boronic ester (77 mg, 94%, 5:95< d^1/d^2, 96:4 e.r., 100% e.s.) as a white foam.

R*f* = 0.4 for both diastereomers (CH_2Cl_2/MeOH 9:1).

$[\alpha]_D^{25}$: +22 (*c* = 6.5)

NMR Spectroscopy (*see spectra*):

^{1}H NMR (400 MHz, $CDCl_3$): δ_H 12.17 (s, 1H, H_S), 7.22–7.15 (m, 1H, H_{Ar}), 7.11–6.96 (m, 3H, H_{Ar}), 3.01–2.83 (m, 2H, $H_{F, Q}$), 2.80–2.58 (m, 2H, $H_{F', Q'}$), 2.72 (d, *J* = 4.9 Hz, 3H, H_R) 2.68 (d, *J* = 4.9 Hz, 3H, $H_{R'}$), 2.37 (ddd, *J* = 12.8, 8.3, 6.2 Hz, 1H, H_E), 2.21–2.10 (m, 1H, H_O), 2.04–1.88 (m, 2H, $H_{M, P}$), 1.85–1.69 (m, 5H, $H_{E', M', N, N', P'}$), 1.60 (ddd, *J* = 13.8, 8.6, 5.1 Hz, 1H, $H_{O'}$), 0.94 (s, 6H, H_A), 0.89 (s, 6H, $H_{A'}$) ppm;

^{13}C NMR (101 MHz, $CDCl_3$): δ_C 149.3 (C_G *or* C_L), 143.6 (C_G *or* C_L), 126.6 (C_{Ar}), 125.8 (C_{Ar}), 124.5 (C_{Ar}), 124.4 (C_{Ar}), 83.4 (C_B), 62.0 (C_D), 56.4 (C_Q), 43.2 (C_R), 41.9 ($C_{R'}$), 38.7 (C_M), 35.2 (C_E), 31.9 (C_O), 31.0 (C_F), 27.7 (C_P), 25.0 (C_A), 24.7 ($C_{A'}$), 21.6 (C_N) ppm; C_C not observed due to quadrupolar relaxation;

^{11}B NMR (128 MHz, $CDCl_3$): δ_B 32.8 ppm;

Assignments made for the major diastereoisomer.

IR (film): ν_{max} 3410 (br), 2957, 2463, 1474, 1372, 1311, 1261, 1141 cm^{-1}.

HRMS (ESI): m/z calc'd for $C_{23}H_{37}{}^{11}BNO_2$ $[M+H]^+$ 370.2916 found 370.2929.

Chiral solvating agent: The enantiopurity of boronic ester **3d.d²** was determined using Pirkle's alcohol ((*R*)-(–)-1-(9-anthryl)-2,2,2-trifluoroethanol) NMR shift reagent (2.0 eq, rt, $CDCl_3$, 0.04 M), 96:4 e.r. A portion of the HCl salt was converted to free amine **17d.d²** by washing with 0.5 M NaOH and extracting with Et_2O for e.r. determination.

^{1}H NMR ($CDCl_3$, 500 MHz) expansion of **(±)-17d.d²** only:

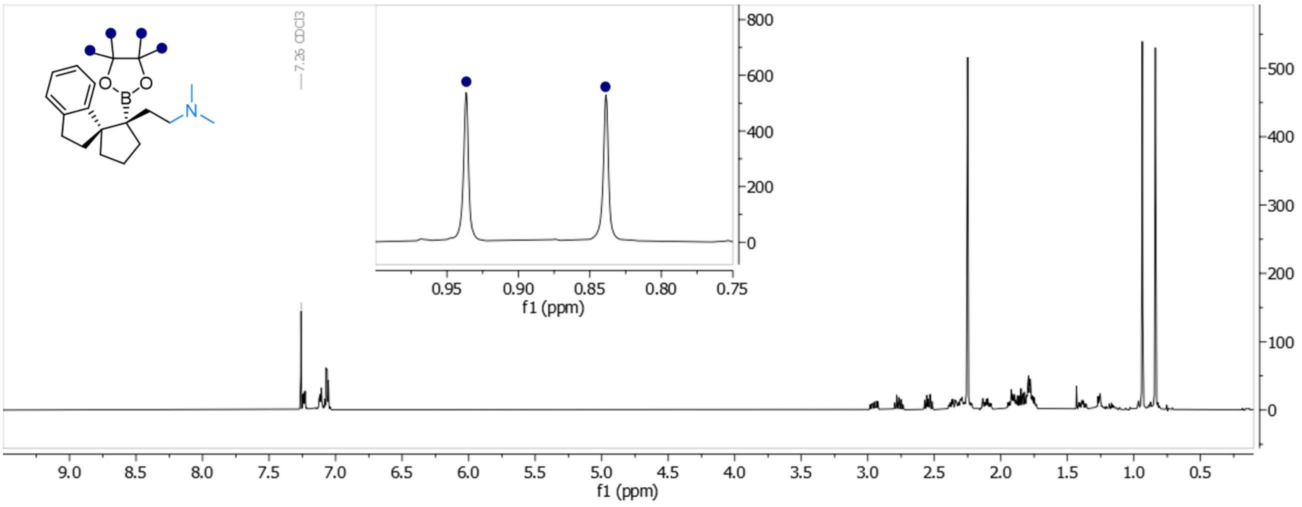

^{1}H NMR ($CDCl_3$, 500 MHz) expansion of **(±)-17d.d²** with Pirkle's alcohol:

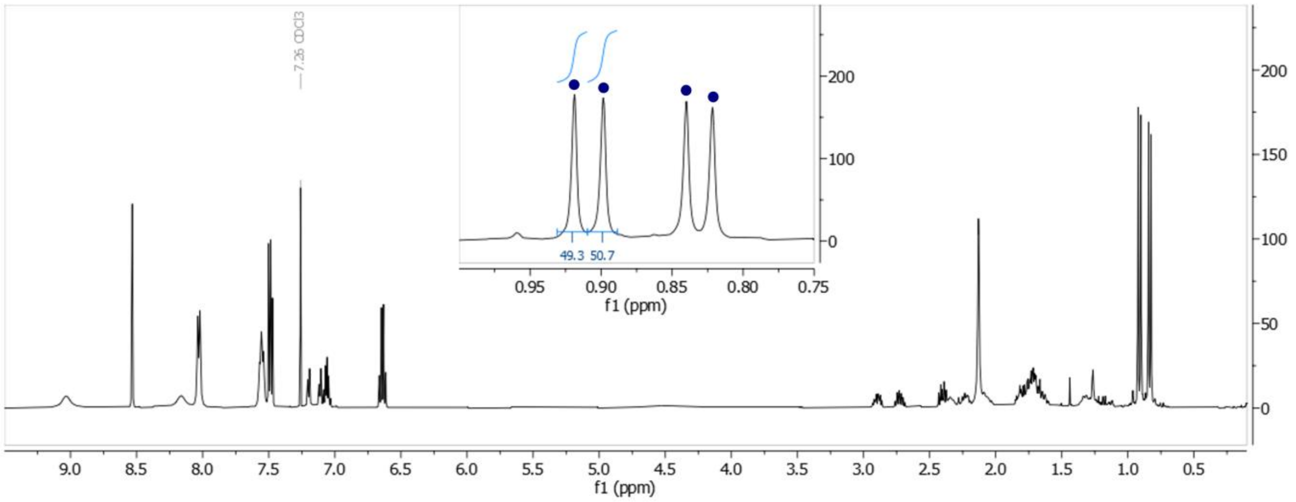

^{1}H NMR ($CDCl_3$, 500 MHz) expansion of **17d.d²** with Pirkle's alcohol:

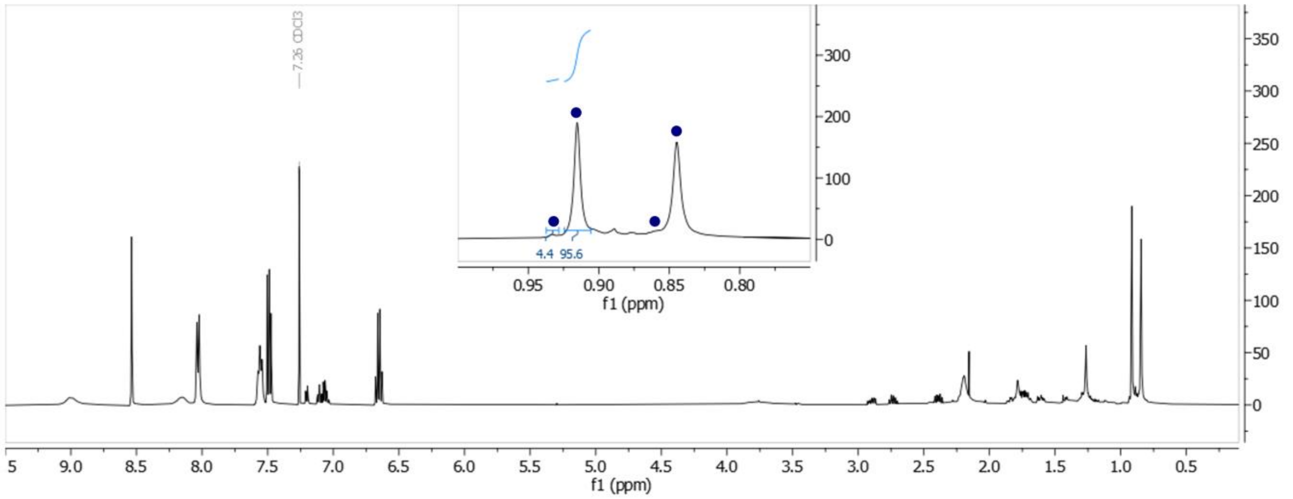

Racemic test reaction:

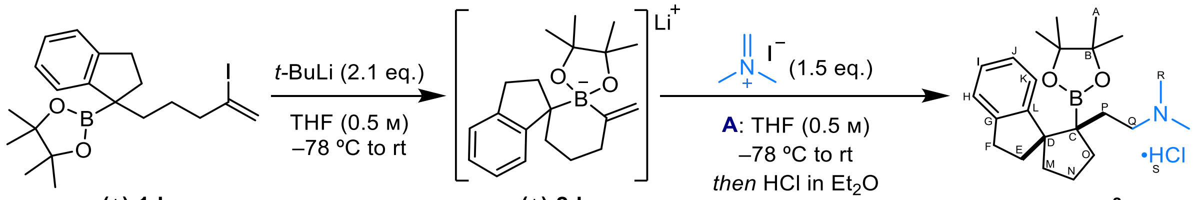

(±)-1d (±)-2d (±)-3d.d2

According to General Procedure E, vinyl iodide **(±)-1d** (44 mg, 0.1 mmol, 1.0 eq.) was allowed to react with *tert*-butyl lithium (1.7 м in pentane, 0.12 mL, 0.21 mmol, 2.1 eq.) and *N,N*-dimethylmethyleneiminium iodide (28 mg, 0.15 mmol, 1.5 eq.) to give a crude residue (>95:5 d.r. by crude ^{1}H NMR) which was dissolved in Et_2O (ca. 0.3 mL) and then 2 N HCl (55 µL, 0.11 mmol, 1.1 eq.) in Et_2O was added. The white precipitate formed was collected *via* suction filtration and dried under high vacuum to afford the racemic boronic ester (38 mg, 94%, 5:95< d^1/d^2) as a white foam.

_N,N_-Dimethyl-2-((1_S_,2_S_)-2-methyl-1-(4,4,5,5-tetramethyl-1,3,2-dioxaborolan-2-yl)cyclopentyl)ethan-1-amine hydrochloride salt (3e)

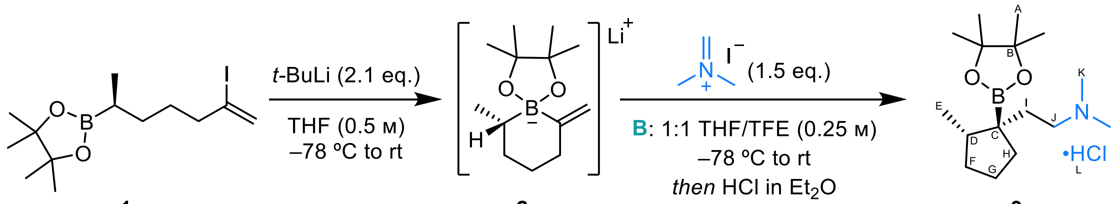

1e 2e 3e

According to General Procedure G, vinyl iodide **1e** (70 mg, 0.2 mmol, 1.0 eq.) was allowed to react with *tert*-butyl lithium (1.7 м in pentane, 0.25 mL, 0.42 mmol, 2.1 eq.) and *N,N*-dimethylmethyleneiminium iodide (56 mg, 0.3 mmol, 1.5 eq.) to give a crude residue (94:6 d.r. by crude GCMS) which was dissolved in Et_2O (ca. 0.5 mL) and then 2 N HCl (110 µL, 0.22 mmol, 1.1 eq.) in Et_2O was added. The white precipitate formed was collected *via* suction filtration and dried under high vacuum to afford the boronic ester (58 mg, 91%, 94:6 d.r., 98:2 e.r., 100% e.s.) as a white foam.

R*f* = 0.2 for both diastereomers (CH_2Cl_2/MeOH 9:1).

$[\alpha]_D^{25}$: +3 (*c* = 6.4).

NMR Spectroscopy (*see spectra*):

^{1}H NMR (400 MHz, $CDCl_3$): δ_H 12.09 (s, 1H, H_L), 3.06–2.92 (m, 2H, H_J), 2.74 (apparent t, *J* = 4.9 Hz, 6H, H_K), 2.02 (td, *J* = 11.4, 10.8, 5.8 Hz, 1H, H_I), 1.87–1.62 (m, 3H, $H_{F, G, H}$), 1.60–1.42 (m, 3H, $H_{D, G', I'}$), 1.36–1.25 (m, 1H, $H_{F'}$), 1.24–1.17 (m, 1H, $H_{H'}$), 1.17 (s, 12H, H_A), 0.93 (d, *J* = 6.9 Hz, 3H, H_E) ppm;

^{13}C NMR (101 MHz, $CDCl_3$): δ_C 82.7 (C_B), 56.3 (C_J), 45.4 (C_D), 43.0 (C_K), 41.8 ($C_{K'}$), 34.7 (C_H), 34.0 (C_F), 31.8 (C_I), 25.1 (C_A), 24.9 ($C_{A'}$), 22.9 (C_G), 17.3 (C_E) ppm; C_C not observed due to quadrupolar relaxation;

^{11}B NMR (128 MHz, $CDCl_3$): δ_B 33.4 ppm;

Assignments made for the major diastereoisomer.

IR (film): v_{max} 3402 (br), 2952, 2868, 1644, 1470, 1372, 1315, 1141 cm^{-1}.

HRMS (ESI): m/z calc'd for $C_{16}H_{33}{}^{11}BNO_2$ [M+H]$^+$ 282.2602 found 282.2611.

Chiral solvating agent: The enantiopurity of boronic ester **3e** was determined using Pirkle's alcohol ((*R*)-(–)-1-(9-anthryl)-2,2,2-trifluoroethanol) NMR shift reagent (2.0 eq, rt, $CDCl_3$, 0.05 м), 98:2 e.r. A portion of the HCl salt was converted to free amine **17e** by washing with 0.5 м NaOH and extracting with Et_2O for e.r. determination.

^1H NMR ($CDCl_3$, 500 MHz) expansion of **(±)-17e** only:

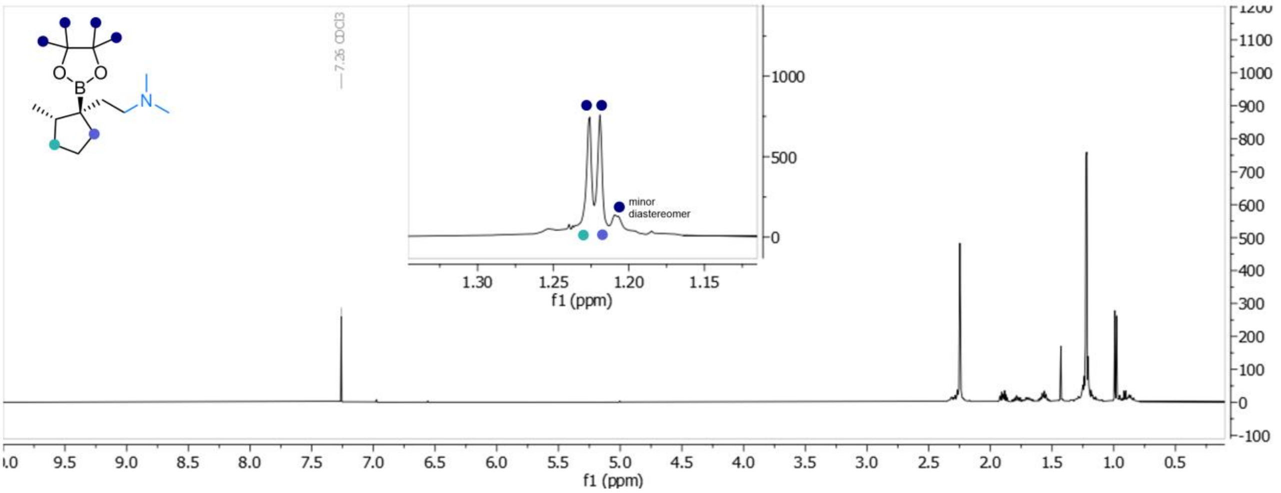

^1H NMR ($CDCl_3$, 500 MHz) expansion of **(±)-17e** with Pirkle's alcohol:

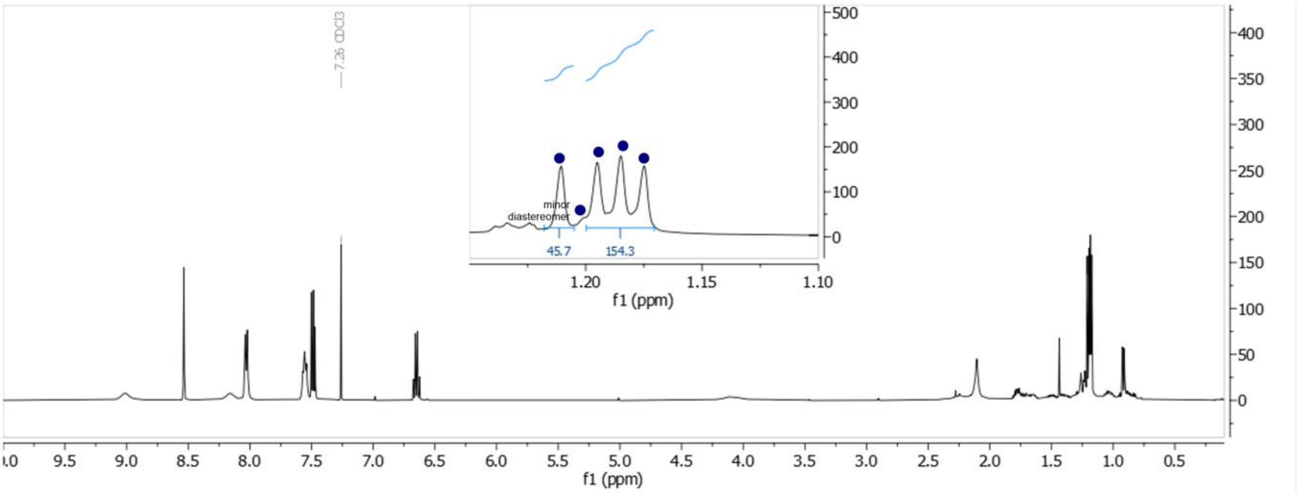

^{1}H NMR ($CDCl_3$, 500 MHz) expansion of **17e** with Pirkle's alcohol:

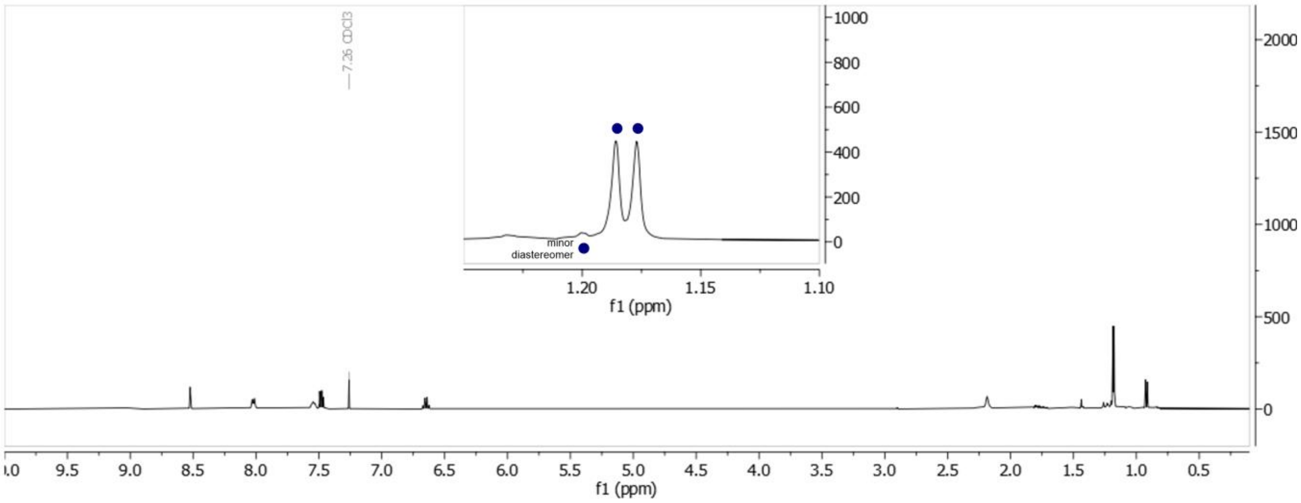

Racemic test reaction:

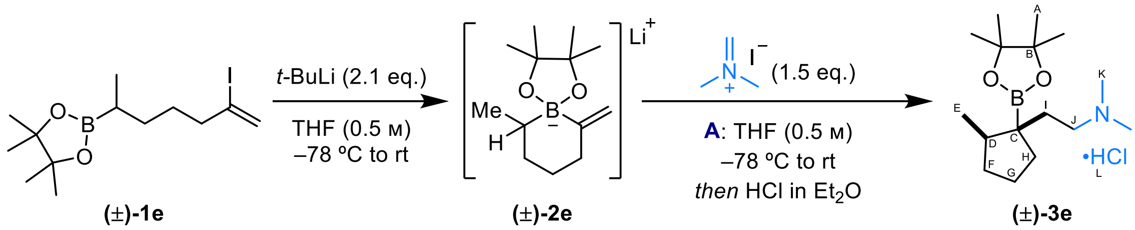

According to General Procedure E, vinyl iodide **(±)-1e** (70 mg, 0.2 mmol, 1.0 eq.) was allowed to react with *tert*-butyl lithium (1.7 M in pentane, 0.25 mL, 0.42 mmol, 2.1 eq.) and *N,N*-dimethylmethyleneiminium iodide (56 mg, 0.3 mmol, 1.5 eq.) to give a crude residue (93:7 d.r. by crude GCMS) which was dissolved in Et_2O (ca. 0.5 mL) and then 2 N HCl (110 µL, 0.22 mmol, 1.1 eq.) in Et_2O was added. The white precipitate formed was collected *via* suction filtration and dried under high vacuum to afford the racemic boronic ester (54 mg, 84%, 93:7 d.r.) as a white foam.

2-((1*S*,2*R*)-2-Isopropyl-1-(4,4,5,5-tetramethyl-1,3,2-dioxaborolan-2-yl)cyclopentyl)-*N,N*-dimethylethan-1-amine hydrochloride salt (3f)

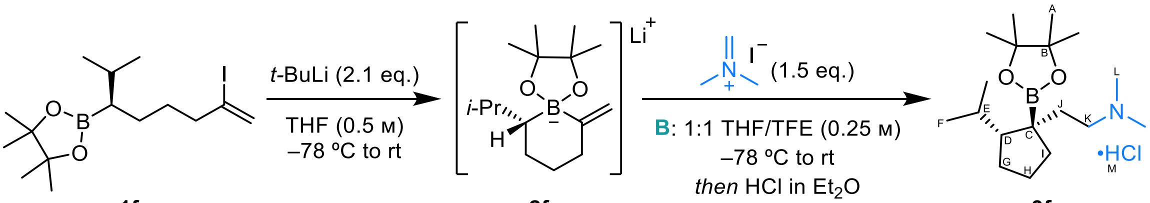

According to General Procedure G, vinyl iodide **1f** (76 mg, 0.2 mmol, 1.0 eq.) was allowed to react with *tert*-butyl lithium (1.7 M in pentane, 0.25 mL, 0.42 mmol, 2.1 eq.) and *N,N*-dimethylmethyleneiminium iodide (56 mg, 0.3 mmol, 1.5 eq.) to give a crude residue (>95:5 d.r. by crude GCMS) which was dissolved in Et_2O (ca. 0.5 mL) and then 2 N HCl (110 µL, 0.22 mmol, 1.1 eq.) in Et_2O was added. The white precipitate formed was collected *via* suction filtration and dried under high vacuum to afford the boronic ester (54 mg, 78%, >95:5 d.r.,

95:5 e.r., 100% e.s.) as a white foam.

R_f = 0.2 for both diastereomers (CH_2Cl_2/MeOH 9:1).

$[\alpha]_D^{25}$**:** +7 (*c* = 5.4).

NMR Spectroscopy (*see spectra*):

^{1}H NMR (400 MHz, $CDCl_3$): δ_H 12.03 (s, 1H, H_M), 3.14–3.04 (m, 1H, H_K), 3.04–2.92 (m, 1H, $H_{K'}$), 2.74 (apparent dd, *J* = 7.9, 4.8 Hz, 6H, H_L), 2.13 (td, *J* = 12.3, 4.3 Hz, 1H, H_J), 1.87–1.71 (m, 2H, $H_{G, I}$), 1.70–1.44 (m, 4H, $H_{E, H, H', J'}$), 1.40–1.19 (m, 3H, $H_{D, G', I'}$), 1.18 (s, 6H, H_A), 1.17 (s, 6H, $H_{A'}$), 0.91 (d, *J* = 6.6 Hz, 3H, H_F), 0.82 (d, *J* = 6.6 Hz, 3H, $H_{F'}$) ppm;

^{13}C NMR (101 MHz, $CDCl_3$): δ_C 83.3 (C_B), 58.6 (C_D), 56.2 (C_K), 43.1 (C_L), 41.7 ($C_{L'}$), 35.9 (C_I), 33.1 (C_J), 32.0 (C_E), 31.5 (C_G), 24.9 (C_A), 24.9 ($C_{A'}$), 23.6 (C_H), 23.3 (C_F), 22.6 ($C_{F'}$) ppm; C_C not observed due to quadrupolar relaxation;

^{11}B NMR (128 MHz, $CDCl_3$): δ_B 33.7 ppm;

Assignments made for the major diastereoisomer.

IR (film): ν_{max} 3340 (br), 2955, 2870, 1634, 1473, 1372, 1311, 1258, 1166 cm^{-1}.

HRMS (ESI): m/z calc'd for $C_{18}H_{37}{}^{11}BNO_2$ $[M+H]^+$ 310.2915 found 310.2919.

Chiral solvating agent: The enantiopurity of boronic ester **3f** was determined using Pirkle's alcohol ((*R*)-(–)-1-(9-anthryl)-2,2,2-trifluoroethanol) NMR shift reagent (2.0 eq, rt, $CDCl_3$, 0.05 M), 95:5 e.r. A portion of the HCl salt was converted to free amine **17f** by washing with 0.5 M NaOH and extracting with Et_2O for e.r. determination.

^{1}H NMR ($CDCl_3$, 500 MHz) expansion of **(±)-17f** only:

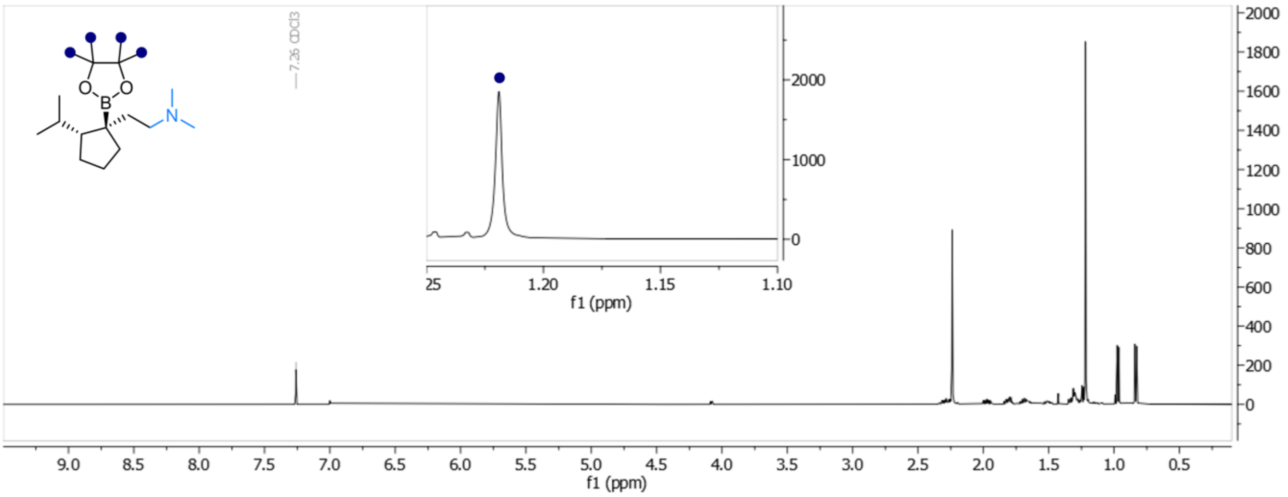

^{1}H NMR ($CDCl_3$, 500 MHz) expansion of **(±)-17f** with Pirkle's alcohol:

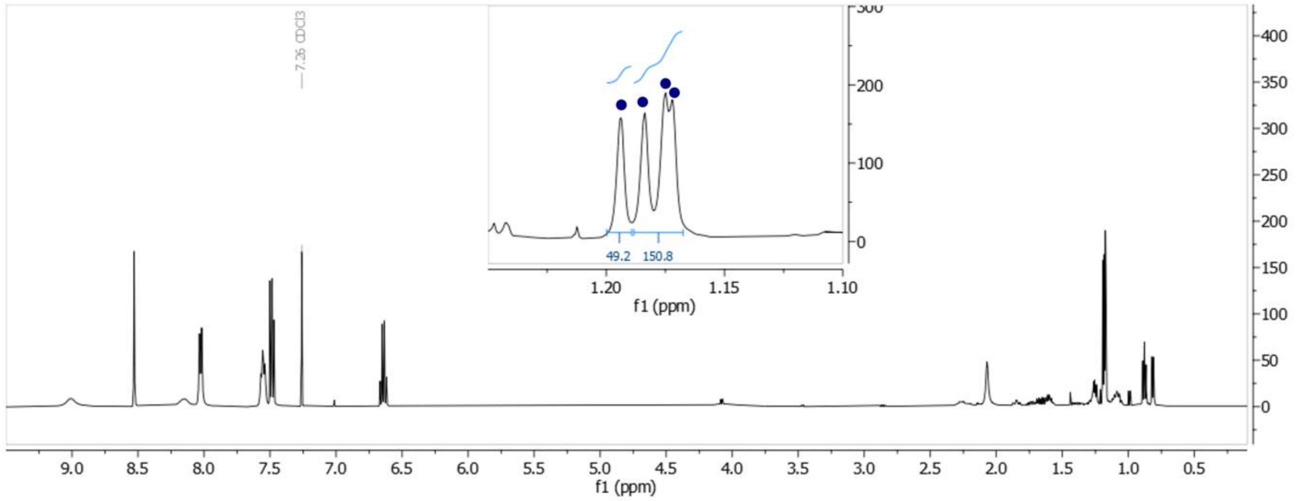

^{1}H NMR ($CDCl_3$, 500 MHz) expansion of **17f** with Pirkle's alcohol:

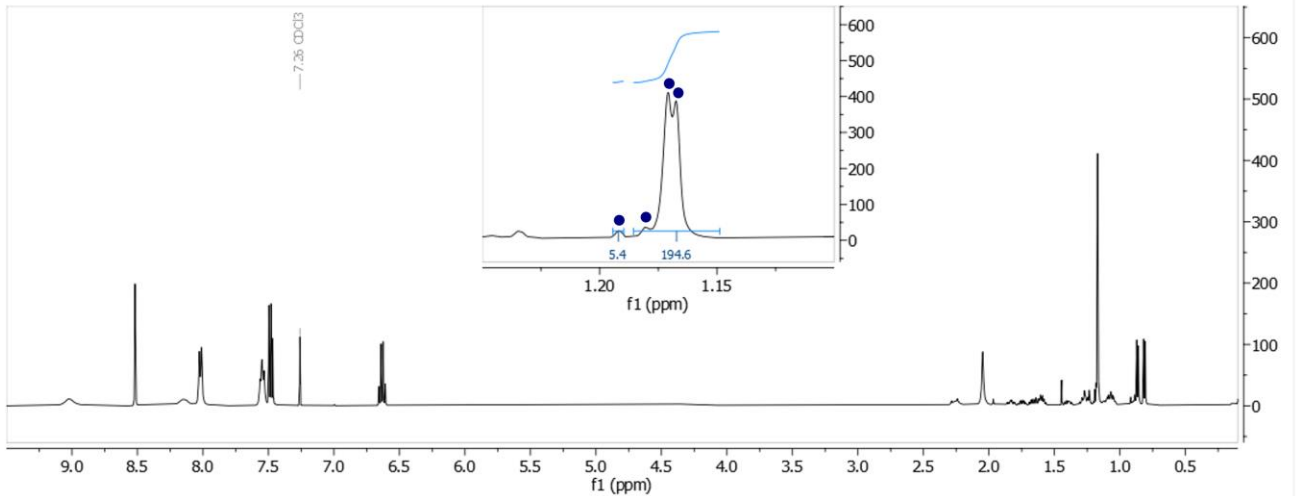

Racemic test reaction:

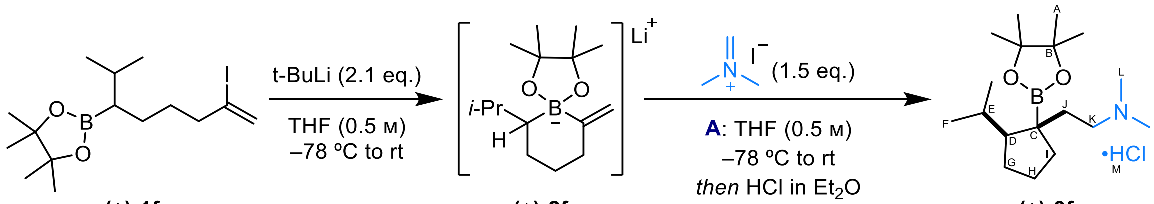

(±)-1f (±)-2f (±)-3f

According to General Procedure **E**, vinyl iodide **(±)-1f** (38 mg, 0.1 mmol, 1.0 eq.) was allowed to react with *tert*-butyl lithium (1.5 м in pentane, 0.14 mL, 0.21 mmol, 2.1 eq.) and *N,N*-dimethylmethyleneiminium iodide (28 mg, 0.15 mmol, 1.5 eq.) to give a crude residue (91:9 d.r. by crude ^{1}H NMR) which was dissolved in Et_2O (ca. 0.3 mL) and then 2 N HCl (55 µL, 0.11 mmol, 1.1 eq.) in Et_2O was added. The white precipitate formed was collected *via* suction filtration and dried under high vacuum to afford the racemic boronic ester (24 mg, 68%, 91:9 d.r.) as

a white foam.

_N,N_-Dimethyl-2-(1-(4,4,5,5-tetramethyl-1,3,2-dioxaborolan-2-yl)cyclopentyl)ethan-1-amine hydrochloride salt (3g)

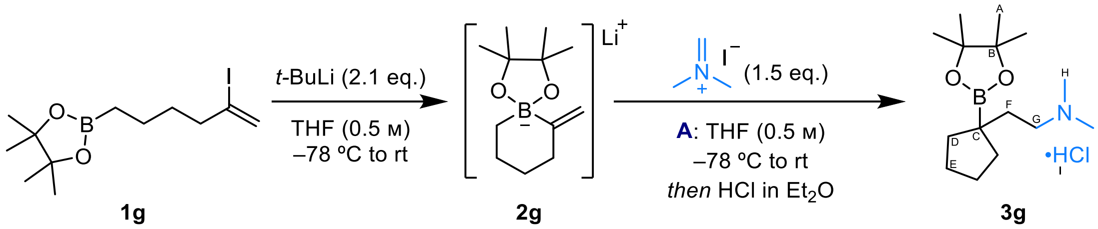

According to General Procedure E, vinyl iodide **1g** (67 mg, 0.2 mmol, 1.0 eq.) was allowed to react with *tert*-butyl lithium (1.8 M in pentane, 0.24 mL, 0.42 mmol, 2.1 eq.) and *N,N*-dimethylmethyleneiminium iodide (56 mg, 0.3 mmol, 1.5 eq.) to give a crude residue which was dissolved in Et_2O (ca. 0.5 mL) and then 2 N HCl (110 µL, 0.22 mmol, 1.1 eq.) in Et_2O was added. The white precipitate formed was collected *via* suction filtration and dried under high vacuum to afford the boronic ester (33 mg, 55%) as a white foam.

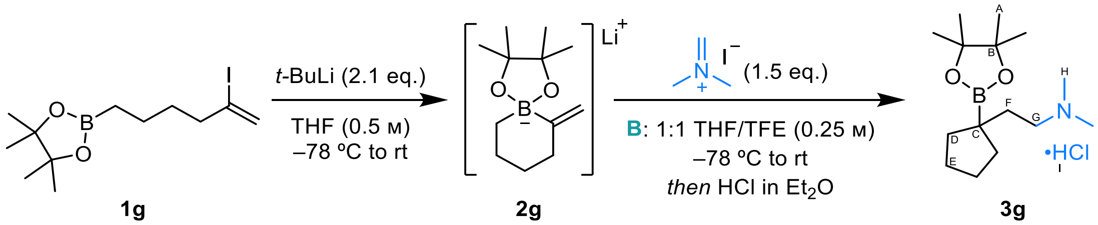

According to General Procedure G, vinyl iodide **1g** (67 mg, 0.2 mmol, 1.0 eq.) was allowed to react with *tert*-butyl lithium (1.8 M in pentane, 0.24 mL, 0.42 mmol, 2.1 eq.) and *N,N*-dimethylmethyleneiminium iodide (56 mg, 0.3 mmol, 1.5 eq.) to give a crude residue which was dissolved in Et_2O (ca. 0.5 mL) and then 2 N HCl (110 µL, 0.22 mmol, 1.1 eq.) in Et_2O was added. The white precipitate formed was collected *via* suction filtration and dried under high vacuum to afford the boronic ester (32 mg, 52%) as a white foam.

R_f = 0.2 (CH_2Cl_2/MeOH 9:1).

NMR Spectroscopy (*see spectra*):

> **^{1}H NMR** (400 MHz, $CDCl_3$): δ_H 12.26 (s, 1H, H_I), 3.07–2.88 (m, 2H, H_G), 2.75 (s, 3H, H_H), 2.74 (s, 3H, $H_{H'}$), 1.83–1.72 (m, 4H, $H_{D, F, F'}$), 1.63–1.55 (m, 4H, $H_{E, E'}$), 1.35–1.20 (m, 2H, $H_{D'}$), 1.19 (s, 12H, H_A) ppm;

> **^{13}C NMR** (101 MHz, $CDCl_3$): δ_C 83.5 (C_B), 56.3 (C_G), 42.5 (C_H), 35.2 (C_D), 31.3 (C_F), 25.1 (C_E), 24.7 (C_A) ppm; C_C not observed due to quadrupolar relaxation;

> **^{11}B NMR** (128 MHz, $CDCl_3$): δ_B 33.7 ppm.

IR (film): ν_{max} 3371, 2929 (br), 2859, 2604, 1737, 1379, 1318, 1260, 1144 cm^{-1}.

HRMS (ESI): m/z calc'd for $C_{15}H_{31}{}^{11}BNO_2$ $[M+H]^+$ 268.2445 found 268.2455.

2.3.3. Electrophile Scope

2.3.3.1 Successful electrophiles

1-(2-((1*S*,2*S*)-2-Methyl-2-phenyl-1-(4,4,5,5-tetramethyl-1,3,2-dioxaborolan-2-yl)cyclopentyl)ethyl)pyrrolidine (3h.d[1])

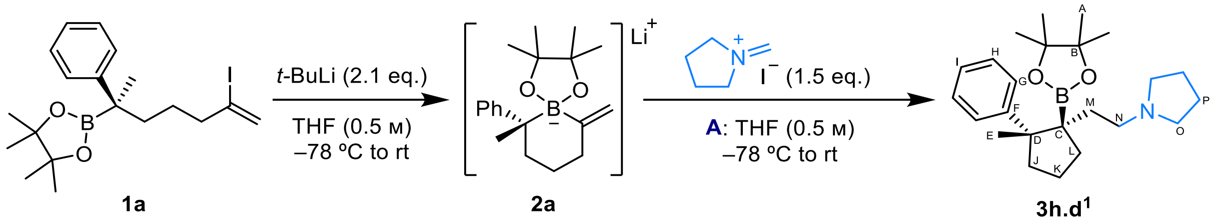

According to General Procedure E, vinyl iodide **1a** (85 mg, 0.2 mmol, 1.0 eq.) was allowed to react with *tert*-butyl lithium (1.6 M in pentane, 0.26 mL, 0.42 mmol, 2.1 eq.) and the pyrrolidine-derived iminium salt[11] (84 mg, 0.4 mmol, 2.0 eq.) to give a crude residue (82:18 d.r. by crude ^{1}H NMR) which was purified by flash column chromatography (9:1 pentane/CH_2Cl_2) to afford the boronic ester (21 mg, 27%, 82:18 d^1/d^2, >98:2 e.r., 100% e.s.) as a colorless oil.

R*f* = 0.2 for both diastereomers (CH_2Cl_2/MeOH 9:1).

$[\alpha]_D^{25}$**:** –2 (*c* = 3.0).

NMR Spectroscopy (*see spectra*):

^{1}H NMR (400 MHz, $CDCl_3$): δ_H 7.45–7.39 (m, 2H, H_G), 7.28–7.20 (m, 2H, H_H), 7.17–7.08 (m, 1H, H_I), 2.42–2.29 (m, 6H, $H_{J, N, O}$), 2.21 (apparent td, *J* = 11.6, 4.6 Hz, 1H, $H_{N'}$), 2.12 (ddd, *J* = 13.1, 9.7, 4.8 Hz, 1H, H_L), 1.95–1.77 (m, 2H, $H_{K, K'}$), 1.75–1.70 (m, 1H, $H_{J'}$), 1.70–1.65 (m, 4H, H_P), 1.56–1.46 (m, 2H, $H_{L', M}$), 1.34 (s, 3H, H_E), 1.30 (s, 6H, H_A), 1.28 (s, 6H, $H_{A'}$), 0.86 (td, *J* = 12.2, 4.6 Hz, 1H, $H_{M'}$) ppm;

^{13}C NMR (101 MHz, $CDCl_3$): δ_C 147.9 (C_F), 127.9 (C_G), 127.5 (C_H), 125.2 (C_I), 83.2 (C_B), 54.8 (C_N), 54.2 (C_O), 53.0 (C_D), 38.3 (C_J), 34.0 (C_M), 32.2 (C_L), 28.7 (C_E), 25.3 (C_A), 25.0 ($C_{A'}$), 24.7 (C_P), 23.3 (C_K) ppm; C_C not observed due to quadrupolar relaxation;

^{11}B NMR (128 MHz, $CDCl_3$): δ_B 33.2 ppm;

Assignments made for the major diastereoisomer.

IR (film): ν_{max} 2952, 2874, 2787, 1601, 1444, 1370, 1308, 1204, 1143 cm^{-1}.

HRMS (ESI): m/z calc'd for $C_{24}H_{39}{}^{11}BNO_2$ $[M+H]^+$ 384.3073 found 384.3086.

Chiral solvating agent: The enantiopurity of boronic ester **3h.d[1]** was determined using Pirkle's alcohol ((*R*)-(–)-1-(9-anthryl)-2,2,2-trifluoroethanol) NMR shift reagent (2.0 eq, rt, $CDCl_3$, 0.05 M), >98:2 e.r.

^{1}H NMR ($CDCl_3$, 500 MHz) expansion of **(±)-3h.d[1]** only:

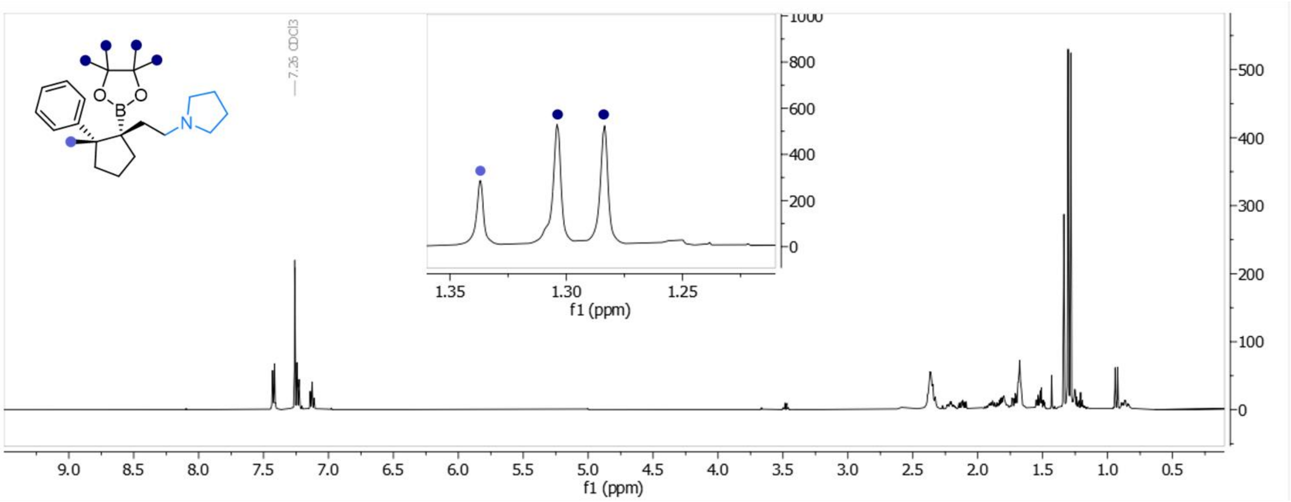

^{1}H NMR ($CDCl_3$, 500 MHz) expansion of **(±)-3h.d[1]** with Pirkle's alcohol:

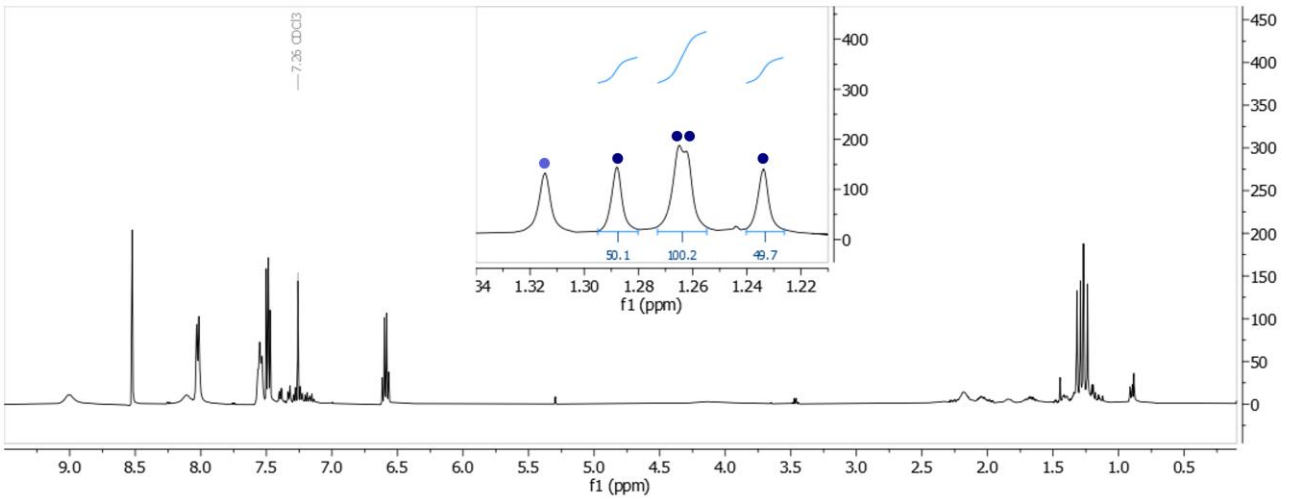

^{1}H NMR ($CDCl_3$, 500 MHz) expansion of **3h.d[1]** with Pirkle's alcohol:

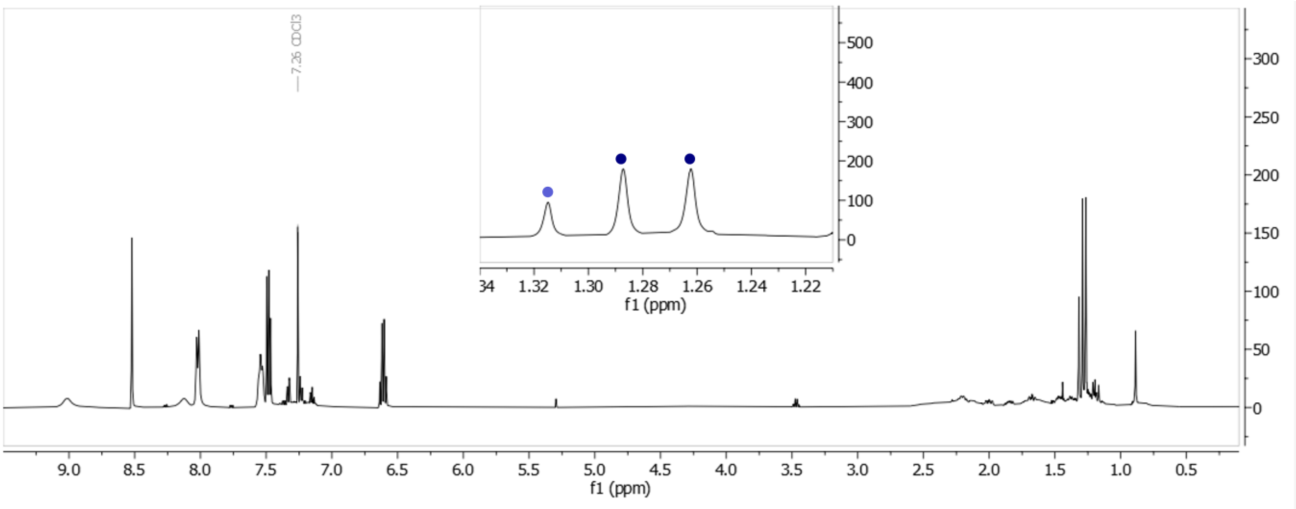

1-(2-((1*R*,2*S*)-2-Methyl-2-phenyl-1-(4,4,5,5-tetramethyl-1,3,2-dioxaborolan-2-yl)cyclopentyl)ethyl)pyrrolidine (3h.d²)

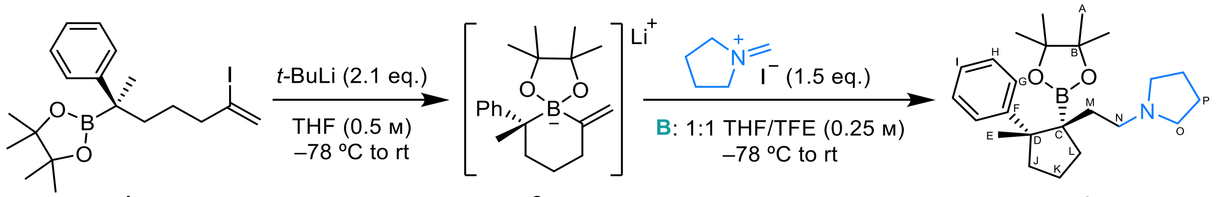

According to General Procedure G, vinyl iodide **1a** (85 mg, 0.2 mmol, 1.0 eq.) was allowed to react with *tert*-butyl lithium (1.6 M in pentane, 0.26 mL, 0.42 mmol, 2.1 eq.) and the pyrrolidine-derived iminium salt[11] (84 mg, 0.4 mmol, 2.0 eq.) to give a crude residue (82:18 d.r. by crude ^{1}H NMR) which was purified by flash column chromatography (9:1 pentane/CH_2Cl_2) to afford the boronic ester (46 mg, 60%, 18:82 d^1/d^2, >98:2 e.r., 100% e.s.) as a colorless oil.

R*f* = 0.2 for both diastereomers (CH_2Cl_2/MeOH 9:1).

$[\alpha]_D^{25}$: –1 (*c* = 8.0).

NMR Spectroscopy (*see spectra*):

^{1}H NMR (400 MHz, $CDCl_3$): δ_H 7.44–7.38 (m, 2H, H_G), 7.25–7.19 (m, 2H, H_H), 7.14–7.09 (m, 1H, H_I), 2.61–2.44 (m, 5H, $H_{N, O}$), 2.42–2.26 (m, 2H, $H_{J, N'}$), 2.18 (apparent td, *J* = 12.1, 4.2 Hz, 1H, H_M), 1.98 (ddd, *J* = 12.8, 9.0, 7.3 Hz, 1H, H_L), 1.87–1.70 (m, 7H, $H_{J', K, P}$), 1.68–1.55 (m, 2H, $H_{L', M'}$), 1.31 (s, 3H, H_E), 0.94 (s, 6H, H_A), 0.92 (s, 6H, $H_{A'}$) ppm;

^{13}C NMR (101 MHz, $CDCl_3$): δ_C 149.5 (C_F), 127.5 (C_H), 127.2 (C_G), 125.5 (C_I), 82.6 (C_B), 55.1 (C_N), 54.4 (C_O), 51.3 (C_D), 38.7 (C_J), 33.1 (C_M), 31.9 (C_L), 25.2 (C_A), 24.7 ($C_{A'}$), 23.4 (C_P), 21.6 (C_K) ppm; C_C not observed due to quadrupolar relaxation;

^{11}B NMR (128 MHz, $CDCl_3$): δ_B 33.2 ppm;

Assignments made for the major diastereoisomer.

IR (film): ν_{max} 2960, 2788, 1443, 1371, 1310, 1210, 1139, 1032 cm^{-1}.

HRMS (ESI): m/z calc'd for $C_{24}H_{39}{}^{11}BNO_2$ $[M+H]^+$ 384.3067 found 384.3068.

Chiral solvating agent: The enantiopurity of boronic ester **3h.d²** was determined using Pirkle's alcohol ((*R*)-(–)-1-(9-anthryl)-2,2,2-trifluoroethanol) NMR shift reagent (2.0 eq, rt, $CDCl_3$, 0.04 M), >98:2 e.r.

^{1}H NMR (CDCl3, 500 MHz) expansion of **(±)-3h.d²** only:

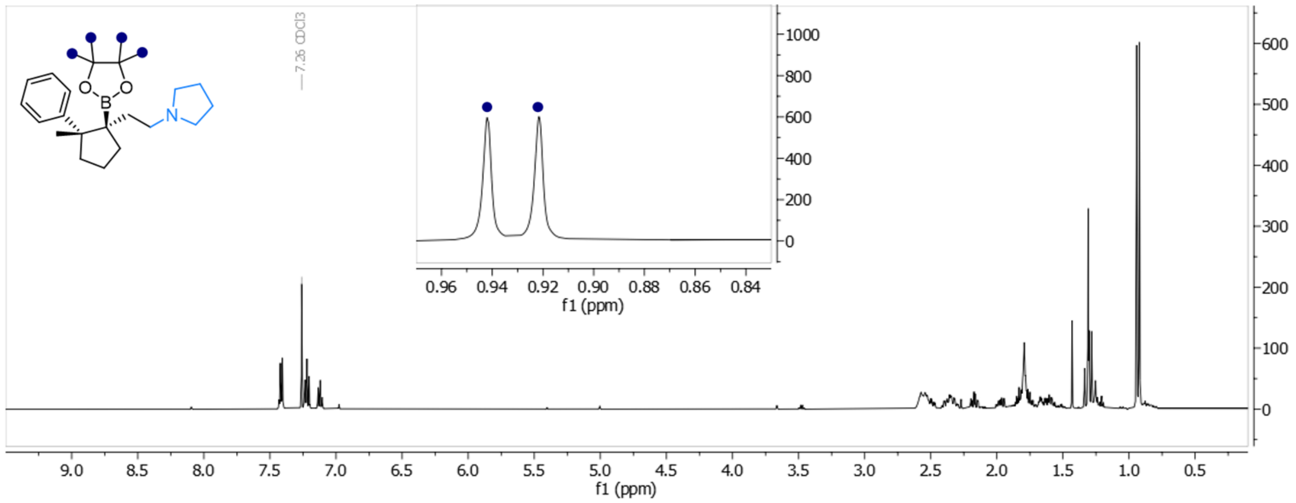

^{1}H NMR (CDCl3, 500 MHz) expansion of **(±)-3h.d²** with Pirkle's alcohol:

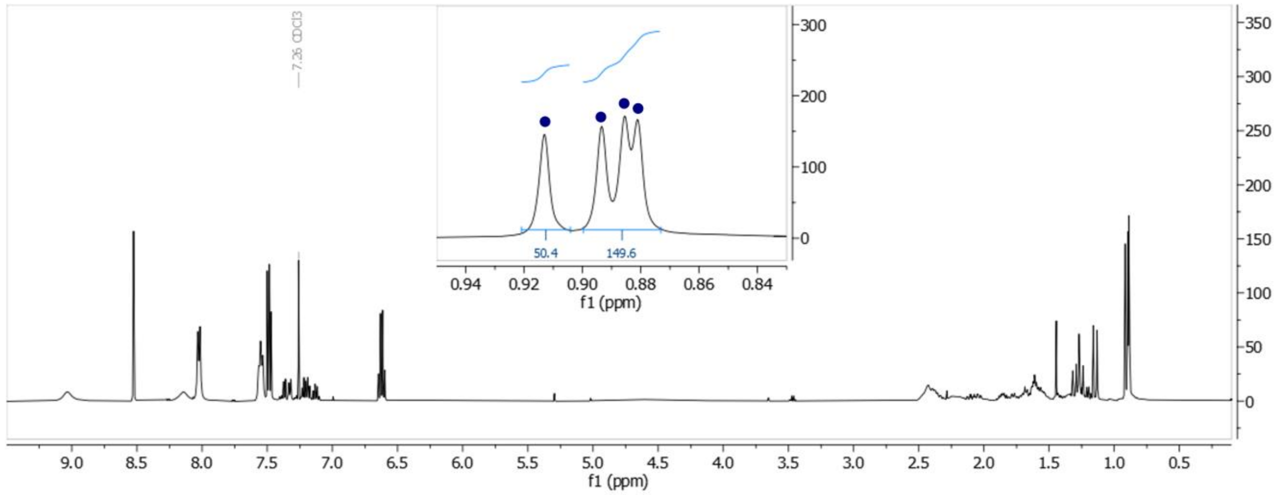

^{1}H NMR (CDCl3, 500 MHz) expansion of **3h.d²** with Pirkle's alcohol:

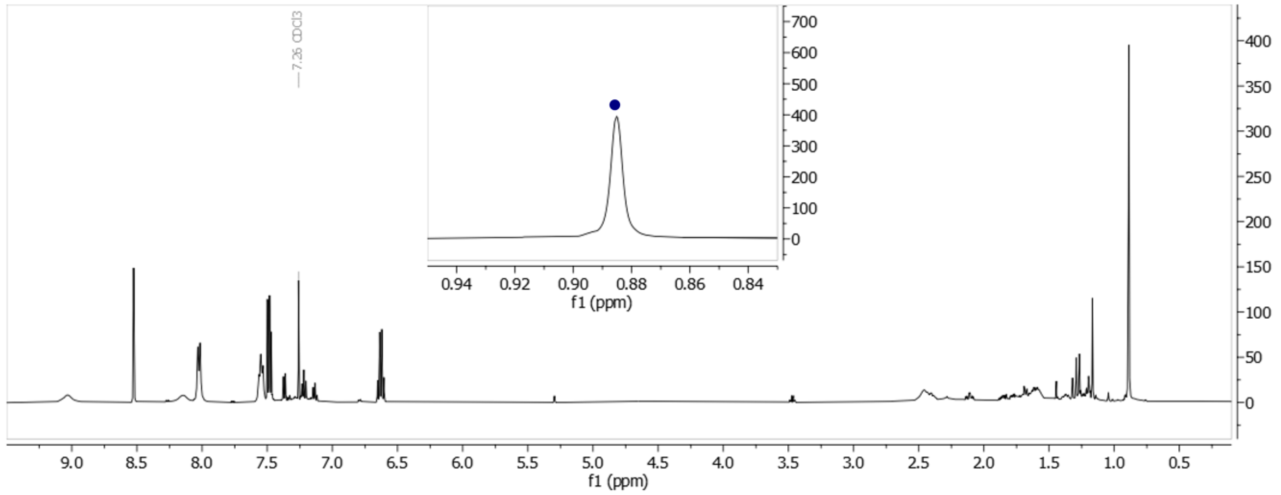

2-((1*S*,2*S*)-1-(Cyclohepta-2,4,6-trien-1-ylmethyl)-2-methyl-2-phenylcyclopentyl)-4,4,5,5-tetramethyl-1,3,2-dioxaborolane (3i.d¹)

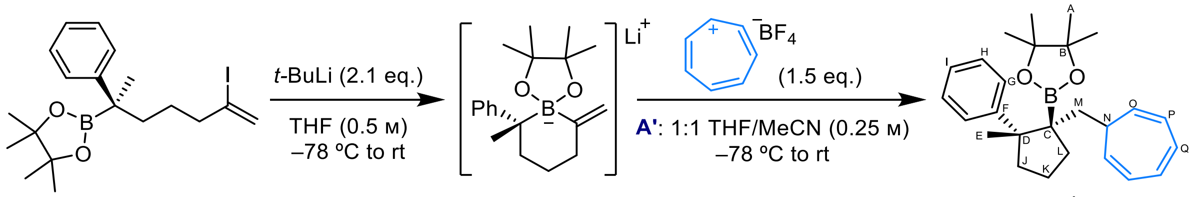

1a **2a** **3i.d¹**

According to General Procedure F, vinyl iodide **1a** (85 mg, 0.2 mmol, 1.0 eq.) was allowed to react with *tert*-butyl lithium (1.6 м in pentane, 0.27 mL, 0.21 mmol, 2.1 eq.) and tropylium tetrafluoroborate (53 mg, 0.3 mmol, 1.5 eq.) to give a crude residue (94:6 d.r. by crude ^{1}H NMR) which was purified by flash column chromatography (100:1 pentane/Et_2O) to afford the boronic ester (34 mg, 44%, 94:6 d^1/d^2, 99:1 e.r., 100% e.s.) as a colorless oil. Boronic ester **5** was isolated as a side product.

The e.r. of boronic ester **3i.d¹** could not be determined by chiral HPLC/SFC. Instead, the boronic ester was oxidised and the e.r. of alcohol **3i'.d¹** was determined.

R*f* = 0.4 for both diastereomers (pentane/CH_2Cl_2 9:1).

$[\alpha]_D^{25}$: +15 (*c* = 6.0).

NMR Spectroscopy (*see spectra*):

^{1}H NMR (400 MHz, $CDCl_3$): δ_H 7.49–7.45 (m, 2H, H_G), 7.35–7.27 (m, 2H, H_H), 7.22–7.16 (m, 1H, H_I), 6.65–6.54 (m, 2H, H_Q), 6.08–6.01 (m, 2H, H_P), 5.10 (dd, *J* = 9.2, 5.7 Hz, 1H, H_O), 5.24 (dd, *J* = 9.2, 5.7 Hz, 1H, $H_{O'}$), 2.48–2.34 (m, 1H, H_J), 2.19–2.07 (m, 1H, H_L), 2.03–1.79 (m, 3H, $H_{K, K', M}$), 1.79–1.71 (m, 1H, $H_{J'}$), 1.71–1.59 (m, 1H $H_{L'}$), 1.36 (s, 3H, H_E), 1.28–1.14 (m, 2H, $H_{M', N}$), 1.24 (s, 6H, H_A), 1.20 (s, 6H, $H_{A'}$) ppm;

^{13}C NMR (101 MHz, $CDCl_3$): δ_C 148.1 (C_F), 130.8 (C_Q), 130.5 ($C_{Q'}$), 127.9 (C_O), 127.9 (C_G), 127.5 (C_H), 127.42 ($C_{O'}$), 125.3 (C_I), 123.1 (C_P), 123.1 ($C_{P'}$), 83.3 (C_B), 53.4 (C_D), 39.4 (C_M), 38.9 (C_N), 38.4 (C_J), 32.5 (C_L), 28.9 (C_E), 25.0 (C_A), 25.0 ($C_{A'}$), 22.1 (C_K) ppm; C_C not observed due to quadrupolar relaxation;

^{11}B NMR (128 MHz, $CDCl_3$): δ_B 34.6 ppm;

Assignments made for the major diastereoisomer.

IR (film): ν_{max} 2975, 1444, 1370, 1308, 1143 cm^{-1}.

HRMS (ESI): m/z calc'd for $C_{26}H_{36}{}^{11}BO_2$ $[M+H]^+$ 391.2808 found 391.2793.

(1*R*,2*R*)-1-(Cyclohepta-2,4,6-trien-1-ylmethyl)-2-methyl-2-phenylcyclopentan-1-ol (3i'.d¹)

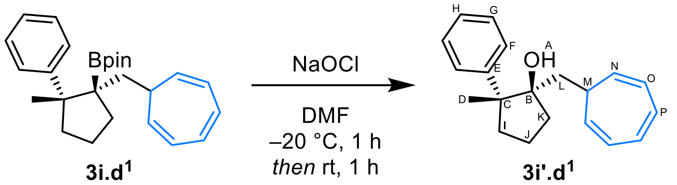

3i.d¹ **3i'.d¹**

According to General Procedure H, boronic ester **3i.d¹** (23 mg, 0.06 mmol, 94:6 d^1/d^2, 1.0 eq.) was allowed to

react with aqueous NaOCl (0.16 mL) in DMF (0.77 mL) to give a crude residue which was purified by flash column chromatography (100:3 pentane/EtOAc) to give the alcohol (8 mg, 49%, 94:6 d[1]/d[2], 99:1 e.r.) as a colorless oil.

R*f* = 0.5 for both diastereomers (pentane/EtOAc 9:1).

$[\alpha]_D^{25}$**:** +2 (*c* = 3.5).

NMR Spectroscopy (*see spectra*):

^{1}H NMR (400 MHz, $CDCl_3$): δ_H 7.46–7.40 (m, 2H, H_F), 7.35–7.28 (m, 2H, H_G), 7.24–7.17 (m, 1H, H_H), 6.72–6.56 (m 2H, H_P), 6.18–6.01 (m 2H, H_O), 5.17 (dd, *J* = 9.3, 5.6 Hz, 1H, H_N), 4.96 (dd, *J* = 9.3, 5.6 Hz, H_N), 2.36–2.22 (m, 1H, H_I), 2.03–1.87 (m, 3H, $H_{I', J, K}$), 1.86–1.68 (m, 3H, $H_{J', K', L}$), 1.68–1.58 (m, 1H, H_M), 1.51–1.43 (m, 1H, $H_{L'}$), 1.41 (s, 3H, H_D) ppm;

^{13}C NMR (101 MHz, $CDCl_3$): δ_C 147.3 (C_E), 131.0 (C_P), 130.8 ($C_{P'}$), 127.9 (C_H), 127.1 (C_G), 126.7 (C_N), 126.1 ($C_{N'}$), 125.9 (C_I), 123.8 (C_O), 123.7 ($C_{O'}$), 84.9 (C_B), 52.4 (C_C), 40.2 (C_L), 35.9 (C_I), 35.8 (C_K), 35.6 (C_M), 23.8 (C_D), 19.1 (C_J) ppm;

Assignments made for the major diastereoisomer.

IR (film): ν_{max} 3582 (br), 2959, 2924, 1723, 1600, 1496, 1464, 1375, 1261, 1092, 1031 cm^{-1}.

HRMS (ESI): m/z calc'd for $C_{20}H_{24}ONa$ $[M+Na]^+$ 303.1719 found 303.1730.

Chiral HPLC (IB column, 97:3 hexane/IPA, 0.5 mL/min, rt, 230 nm): 11.0 min (major), 11.4 min (minor), 99:1 e.r.

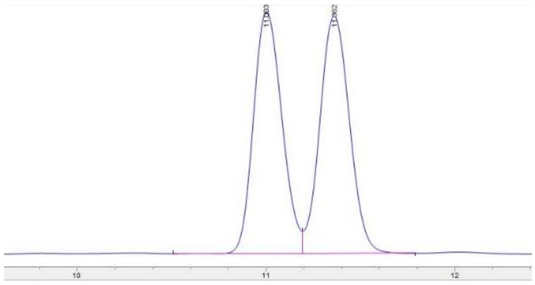

#	Time	Type	Area	Height	Width	Area%	Symmetry
1	11.003	VV R	4124.9	394	0.1651	49.453	0.811
2	11.362	VB	4216.1	386.3	0.1698	50.547	0.896

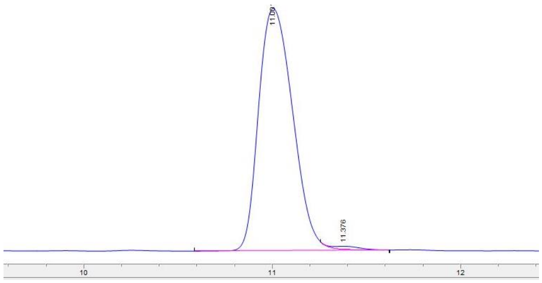

#	Time	Type	Area	Height	Width	Area%	Symmetry
1	11.001	BV R	8684.1	689.6	0.2004	98.915	0.71
2	11.376	VB E	95.3	9.5	0.144	1.085	0.552

(*S*)-2-(6-(cyclohepta-2,4,6-trien-1-yl)-6-phenylhept-1-en-2-yl)-4,4,5,5-tetramethyl-1,3,2-dioxaborolane (5)

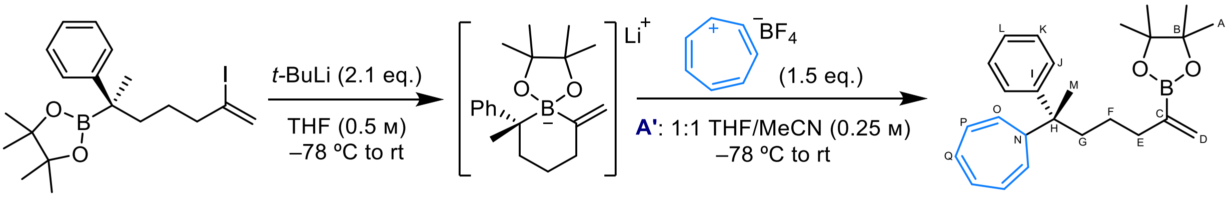

1a **2a** **5**

According to General Procedure F, vinyl iodide **1a** (85 mg, 0.2 mmol, 1.0 eq.) was allowed to react with *tert*-butyl lithium (1.6 M in pentane, 0.27 mL, 0.21 mmol, 2.1 eq.) and tropylium tetrafluoroborate (53 mg, 0.3 mmol,

1.5 eq.) to give a crude residue which was purified by flash column chromatography (100:1 pentane/Et_2O) to afford boronic ester **5** (16 mg, 21%, 93:7 e.r., 88% e.s.) alongside boronic ester **3i.d**[1].

The formation of boronic ester **5** is not enantiospecific. It is believed that this reaction occurs with predominant inversion of configuration. This is an observation consistent with our group's previous work into the reactivity of boronate complexes as nucleophiles, where the reaction with an electrophile occurs through two competing pathways: 1) polar pathway (2 electron) giving products with inversion at the reacting centre, 2) SET pathway (1 electron) with racemisation.[12]

The e.r. of boronic ester **5** could not be determined by chiral HPLC/SFC. Instead, the boronic ester was oxidised and the e.r. of ketone **5'** was determined.

R*f* = 0.2 (pentane/CH_2Cl_2 9:1).

$[\alpha]_D^{25}$: +17 (*c* = 2.4).

NMR Spectroscopy (*see spectra*):

^{1}**H NMR** (400 MHz, $CDCl_3$): δ_H 7.31-7.23 (m, 4H, $H_{J, K}$), 7.20-7.11 (m, 1H, H_L), 6.68-6.58 (m, 2H, H_Q), 6.27-6.17 (m, 1H, H_P), 6.10-6.02 (m, 1H, $H_{P'}$), 5.68 (apparent s, 1H, H_D), 5.48 (dt, J = 3.2, 1.4 Hz, 1H, $H_{D'}$), 5.45 (dd, *J* = 9.5, 5.8 Hz, 1H, H_O), 5.11 (dd, *J* = 9.5, 5.8 Hz, 1H, $H_{O'}$), 2.08-1.98 (m, 2H, H_E), 1.88-1.69 (m, 3H, $H_{G, G', N}$), 1.47 (s, 3H, H_M), 1.29-1.23 (m, 1H, H_F), 1.19 (s, 12H, H_A), 1.11-0.98 (m, 1H, $H_{F'}$) ppm;

^{13}**C NMR** (101 MHz, $CDCl_3$): δ_C 146.5 (C_I), 130.8 (C_Q), 130.5 ($C_{Q'}$), 128.8 (C_D), 128.0 (C_J *or* C_K), 127.0 (C_J *or* C_K), 125.6 (C_L), 124.8 (C_P), 124.4 (C_O), 124.2 ($C_{P'}$), 123.4 ($C_{O'}$), 83.3 (C_B), 48.4 (C_N), 41.9 (C_H), 40.7 (C_G), 36.0 (C_E), 24.7 (C_A), 24.7 ($C_{A'}$), 23.4 (C_F), 20.7 (C_M) ppm; C_C not observed due to quadrupolar relaxation;

^{11}**B NMR** (128 MHz, $CDCl_3$): δ_B 29.4 ppm.

IR (film): ν_{max} 2976, 2933, 1616, 1444, 1370, 1308, 1142 cm^{-1}.

HRMS (ESI): m/z calc'd for $C_{26}H_{35}{}^{11}BNaO_2$ $[M+Na]^+$ 413.2627 found 413.2624.

(*S*)-6-(cyclohepta-2,4,6-trien-1-yl)-6-phenylheptan-2-one (5')

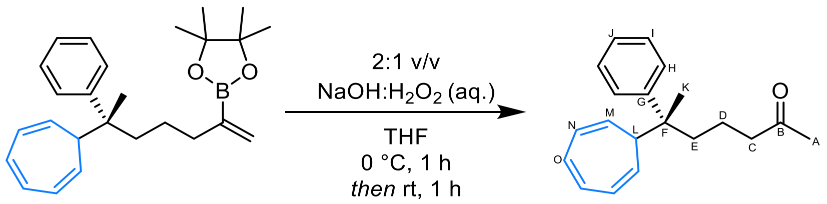

According to General Procedure **D**, boronic ester **5** (10 mg, 0.03 mmol, 1.0 eq.) was reacted with a 2 M aqueous solution of NaOH (0.17 mL) and aqueous H_2O_2 (30% w/w, 0.08 mL) at 0 °C in THF (0.15 mL). The product (5 mg, 70%, 93:7 e.r.) was isolated as a colorless oil after purification by flash column chromatography (pentane/EtOAc 9:1).

R*f* = 0.3 (pentane/EtOAc 9:1).

$[\alpha]_D^{25}$: +46 (c = 2.0).

NMR Spectroscopy (*see spectra*):

^{1}H NMR (400 MHz, $CDCl_3$): δ_H 7.27–7.20 (m, 4H, $H_{H,\ I}$), 7.15–7.09 (m, 1H, H_J), 6.64–6.54 (m, 2H, H_O), 6.20–6.14 (m, 1H, H_N), 6.04–5.95 (m, 1H, $H_{N'}$), 5.36 (dd, J = 9.5, 5.8 Hz, 1H, H_M), 5.03 (dd, J = 9.5, 5.8 Hz, 1H, $H_{M'}$), 2.22 (t, J = 7.3 Hz, 2H, H_C), 1.95 (s, 3H, H_A), 1.79–1.63 (m, 3H, $H_{E,\ E',\ L}$), 1.43 (s, 3H, H_K), 1.38–1.26 (m, 1H, H_D), 1.20–1.05 (m, 1H, $H_{D'}$) ppm;

^{13}C NMR (101 MHz, $CDCl_3$): δ_C 208.9 (C_B), 146.0 (C_G), 130.9 (C_O), 130.5 ($C_{O'}$), 128.2 (C_H *or* C_I), 126.9 (C_H *or* C_I), 125.9 (C_J), 125.0 (C_N), 124.3 ($C_{N'}$), 124.1 (C_M), 122.9 ($C_{M'}$), 48.3 (C_L), 44.2 (C_C), 41.9 (C_F), 40.7 (C_E), 29.7 (C_A), 20.4 (C_K), 18.4 (C_D) ppm.

IR (film): ν_{max} 3002, 2949, 1714 (C=O), 1498, 1445, 1359, 1161 cm^{-1}.

HRMS (ESI): m/z calc'd for $C_{20}H_{24}O$ $[M+H]^+$ 281.1900 found 281.1894.

Chiral HPLC (IB column, 90:10 hexane/IPA, 1 mL/min, rt, 254 nm): 5.4 min (major), 5.8 (minor), 93:7 e.r.

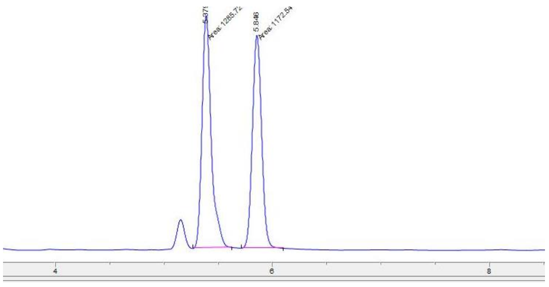

#	Time	Type	Area	Height	Width	Area%	Symmetry
1	5.379	MM	1285.7	218.4	0.0981	52.302	0.652
2	5.846	MM	1172.5	200.1	0.0977	47.698	0.867

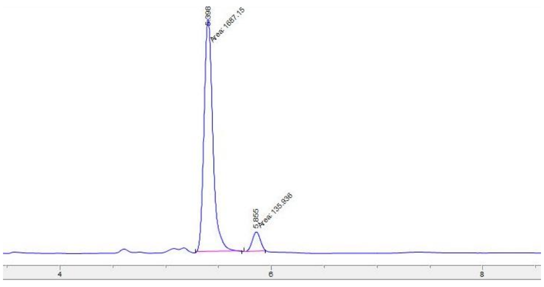

#	Time	Type	Area	Height	Width	Area%	Symmetry
1	5.398	MM	1687.2	288.9	0.0973	92.544	0.811
2	5.855	MM	135.9	24.6	0.0921	7.456	1.028

2-((1*R*,2*S*)-1-(Cyclohepta-2,4,6-trien-1-ylmethyl)-2-methyl-2-phenylcyclopentyl)-4,4,5,5-tetramethyl-1,3,2-dioxaborolane (3i.d^2)

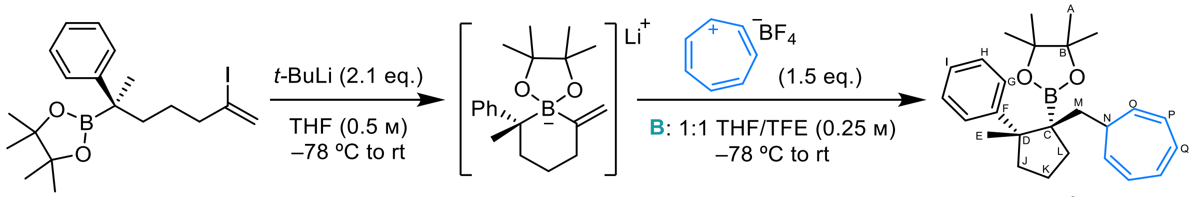

1a **2a** **3i.d^2**

According to General Procedure **G**, vinyl iodide **1a** (85 mg, 0.2 mmol, 1.0 eq.) was allowed to react with *tert*-butyl lithium (1.6 M in pentane, 0.27 mL, 0.42 mmol, 2.1 eq.) and tropylium tetrafluoroborate (53 mg, 0.3 mmol, 1.5 eq.) to give a crude residue (83:17 d.r. by crude ^{1}H NMR) which was purified by flash column chromatography (100:1 pentane/Et_2O) to afford the boronic ester (54 mg, 69%, 17:83 d^1/d^2, 99:1 e.r., 100% e.s.) as a colorless oil.

The e.r. of boronic ester **3i.d^2** could not be determined by chiral HPLC/SFC. Instead, the boronic ester was oxidised and the e.r. of alcohol **3i'.d^2** was determined.

R_f = 0.2 for both diastereomers (pentane/CH_2Cl_2 9:1).

$[\alpha]_D^{25}$: –17 (*c* = 9.4).

NMR Spectroscopy (*see spectra*):

^{1}H NMR (400 MHz, $CDCl_3$): δ_H 7.46–7.41 (m, 2H, H_G), 7.29–7.23 (m, 2H, H_H), 7.19–7.10 (m, 1H, H_I), 6.71–6.57 (m, 2H, H_Q), 6.19–6.08 (m, 2H, H_P), 5.31 (dd, J = 9.2, 5.7 Hz, 1H, H_O), 5.25 (dd, J = 9.2, 5.7 Hz, 1H, $H_{O'}$), 2.44 (dd, J = 13.0, 4.3 Hz, 1H, H_M), 2.41–2.31 (m, 1H, H_J), 2.01–1.77 (m, 5H, $H_{J, K, K', L, M'}$), 1.78–1.70 (m, 1H, $H_{L'}$), 1.41 (s, 3H, H_E), 1.34–1.27 (m, 1H, H_N), 0.93 (s, 6H, H_A), 0.88 (s, 6H, $H_{A'}$) ppm;

^{13}C NMR (101 MHz, $CDCl_3$): δ_C 150.3 (C_F), 130.9 (C_Q), 130.6 (C_Q), 127.9 (C_O), 127.6 (C_H), 127.3 (C_G), 127.2 (C_O), 125.5 (C_I), 123.4 (C_P), 123.3 ($C_{P'}$), 82.3 (C_B), 51.6 (C_D), 39.0 (C_N), 38.7 (C_J), 38.1 (C_M), 32.0 (C_L), 25.1 (C_A), 24.9 ($C_{A'}$), 24.3 (C_E), 21.9 (C_K) ppm; C_C not observed due to quadrupolar relaxation;

^{11}B NMR (128 MHz, $CDCl_3$): δ_B 34.6 ppm;

Assignments made for the major diastereoisomer.

IR (film): ν_{max} 2974, 2872, 1443, 1372, 1311, 1142 cm^{-1}.

HRMS (ESI): m/z calc'd for $C_{26}H_{36}{}^{11}BO_2$ [M+H]$^+$ 391.2803 found 391.2802.

(1*S*,2*R*)-1-(Cyclohepta-2,4,6-trien-1-ylmethyl)-2-methyl-2-phenylcyclopentan-1-ol (3i'.d^2)

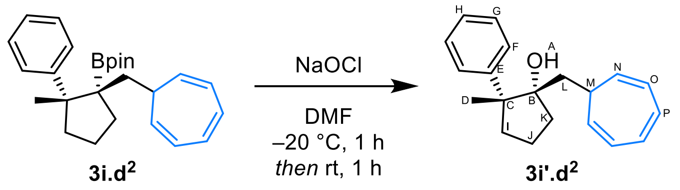

According to General Procedure H, boronic ester **3i.d^2** (19 mg, 0.05 mmol, 17:83 d^1/d^2, 1.0 eq.) was allowed to react with aqueous NaOCl (0.14 mL) in DMF (0.65 mL) to give a crude residue which was purified by flash column chromatography (100:3 pentane/EtOAc) to give the alcohol (6 mg, 41%, 17:83 d^1/d^2, 99:1 e.r.) as a colorless oil.

R_f = 0.5 for both diastereomers (pentane/EtOAc 9:1).

$[\alpha]_D^{25}$: –32 (*c* = 2.3).

NMR Spectroscopy (*see spectra*):

^{1}H NMR (400 MHz, $CDCl_3$): δ_H 7.57–7.48 (m, 2H, H_F), 7.41–7.34 (m, 2H, H_G), 7.32–7.24 (m, 1H, H_H), 6.68–6.56 (m, 2H, H_P), 6.18–6.03 (m, 2H, H_O), 5.23–5.10 (m, 2H, H_N), 2.81–2.66 (m, 1H, H_I), 2.18–2.10 (m, 1H, H_L), 2.01–1.70 (m, 6H, $H_{J, J', K, K', L', M}$), 1.64–1.55 (m, 1H, $H_{I'}$), 1.36 (s, 3H, H_D) ppm;

^{13}C NMR (101 MHz, $CDCl_3$): δ_C 142.9 (C_E), 130.9 (C_P), 130.8 (C_P), 128.2 (C_G), 128.1 (C_F), 126.7 (C_N), 126.6 (C_H), 126.3 ($C_{N'}$), 123.9 (C_O), 123.7 ($C_{O'}$), 84.4 (C_B), 53.9 (C_C), 39.2 (C_L), 36.0 (C_K), 35.6 ($C_{I, M}$), 23.6 (C_D), 19.5 (C_J) ppm;

Assignments made for the major diastereoisomer.

IR (film): ν_{max} 3457 (br), 2964, 2935, 1600, 1497, 1443, 1379, 1034 cm^{-1}.

HRMS (ESI): m/z calc'd for $C_{20}H_{23}$ $[M+H–H_2O]^+$ 263.1794 found 263.1794.

Chiral SFC (Whelk-O1 column, iso 5% hexane/IPA 10%, 4 mL/min, 125 bar): 1.7 min (d^2, minor enantiomer), 2.0 min (d^2, major enantiomer), 2.4 min (d^1, both enantiomers) 99:1 e.r.

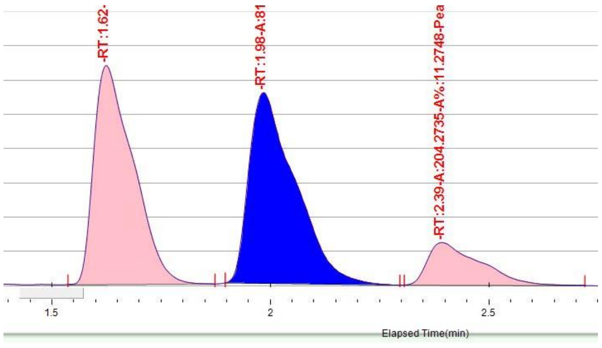

File	Signal	Peak Name	Area Percent	Area	Retention Time	Height
MEF-D-32rac_03-12-2020 14-54-49.tta	MaxAbsorbance	Peak1	43.8426	794.3291	1.62 min	127.8296
		Peak2	44.8826	813.1703	1.98 min	111.3337
		Peak3	11.2748	204.2735	2.39 min	24.8371

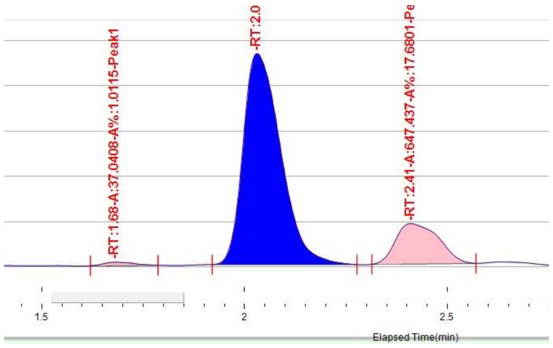

File	Signal	Peak Name	Area Percent	Area	Retention Time	Height
MEF-D-38ee_03-12-2020 17-26-48.tta	MaxAbsorbance	Peak1	1.0115	37.0408	1.68 min	7.4534
		Peak2	81.3084	2977.4771	2.03 min	466.7121
		Peak3	17.6801	647.437	2.41 min	89.1287

2-((1*S*,2*S*)-1-(Benzo[*d*][1,3]dithiol-2-ylmethyl)-2-methyl-2-phenylcyclopentyl)-4,4,5,5-tetramethyl-1,3,2-dioxaborolane (3j.d¹)

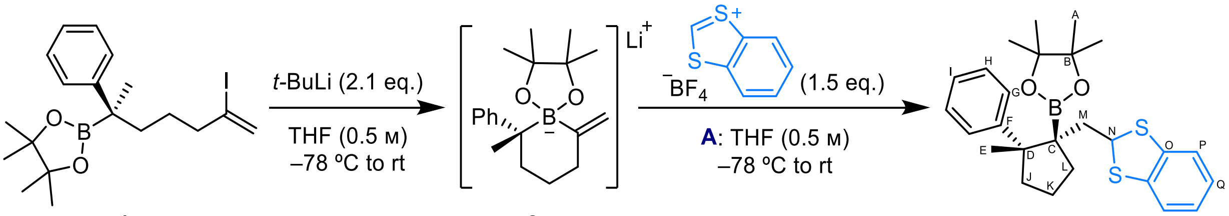

1a **2a** **3j.d¹**

According to General Procedure E, vinyl iodide **1a** (85 mg, 0.2 mmol, 1.0 eq.) was allowed to react with *tert*-butyl lithium (1.7 M in pentane, 0.25 mL, 0.42 mmol, 2.1 eq.) and 1,3-benzodithiolium tetrafluoroborate (72 mg, 0.3 mmol, 1.5 eq.) to give a crude residue (86:14 d.r. by crude ^{1}H NMR) which was purified by flash column chromatography (100:1 pentane/Et_2O) to afford the boronic ester (24 mg, 26%, 86:14 d^1/d^2, 99:1 e.r., 100% e.s.) as a colorless oil.

The e.r. of boronic ester **3j.d¹** could not be determined by chiral HPLC/SFC. Instead, the boronic ester was oxidised and the e.r. of alcohol **3j'.d¹** was determined.

$\mathbf{R_f}$ = 0.3 for both diastereomers (pentane/CH_2Cl_2 9:1).

$[\alpha]_D^{25}$: –8 (*c* = 1.2).

NMR Spectroscopy (*see spectra*):

^{1}H NMR (400 MHz, $CDCl_3$): δ_H 7.40–7.34 (m, 2H, H_G), 7.29–7.23 (m, 2H, H_H), 7.18–7.12 (m, 1H, H_I), 7.11–7.05 (m, 2H, H_P *or* H_Q), 6.95–6.89 (m, 2H, H_P *or* H_Q), 4.98 (dd, *J* = 8.4, 4.6 Hz, 1H, H_N), 2.35–2.18 (m, 2H, $H_{J,\,L}$), 2.11 (dd, *J* = 14.6, 4.6 Hz, 1H, H_M), 2.01–1.82 (m, 2H, $H_{K,\,K'}$), 1.81–1.67 (m, 2H, $H_{J',\,L'}$), 1.53 (dd, *J* =

14.6, 8.4 Hz, 1H, $H_{M'}$), 1.34 (s, 6H, H_A), 1.32 (s, 9H, $H_{A', E}$) ppm;

^{13}C NMR (101 MHz, $CDCl_3$): δ_C 147.7 (C_F), 138.4 (C_O), 138.1 ($C_{O'}$), 127.8 (C_H), 127.7 (C_G), 125.6 (C_I), 125.2 (C_P *or* C_Q), 125.1 (C_P *or* C_Q), 122.1 (C_P *or* C_Q), 121.9 (C_P *or* C_Q), 83.6 (C_B), 54.1 (C_D *or* C_N), 54.0 (C_D *or* C_N), 43.4 (C_M), 38.1 (C_J), 32.6 (C_L), 29.0 (C_E), 25.3 (C_A), 25.1 ($C_{A'}$), 21.8 (C_K) ppm; C_C not observed due to quadrupolar relaxation;

^{11}B NMR (128 MHz, $CDCl_3$): δ_B 33.3 ppm;

Assignments made for the major diastereoisomer.

IR (film): ν_{max} 2973, 2874, 1444, 1361, 1309, 1208, 1140 cm^{-1}.

HRMS (ESI): m/z calc'd for $C_{26}H_{33}{}^{11}BNaO_2S_2$ [M+Na]$^+$ 475.1912 found 475.1912.

Racemic test reaction:

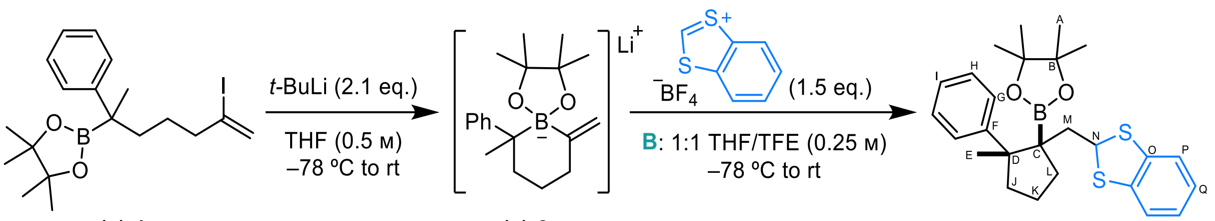

According to General Procedure H, vinyl iodide **(±)-1a** (43 mg, 0.1 mmol, 1.0 eq.) was allowed to react with *tert*-butyl lithium (1.7 M in pentane, 0.12 mL, 0.24 mmol, 2.1 eq.) and 1,3-benzodithiolium tetrafluoroborate (36 mg, 0.15 mmol, 1.5 eq.) to give a crude residue (54:46 d.r. by crude ^{1}H NMR) which was purified by flash column chromatography (100:1 pentane/Et_2O) to afford the racemic boronic ester (20 mg, 44%, 54:46 d^1/d^2) as a colorless oil.

(1*R*,2*R*)-1-(Benzo[*d*][1,3]dithiol-2-ylmethyl)-2-methyl-2-phenylcyclopentan-1-ol (3j'.d^1)

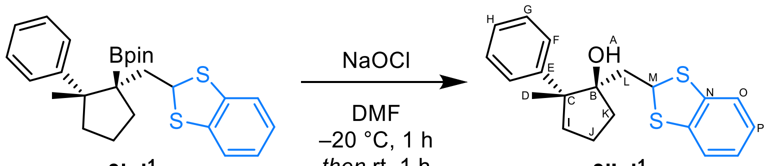

According to General Procedure H, boronic ester **3j.d^1** (12 mg, 0.03 mmol, 86:14 d^1/d^2, 1.0 eq.) was allowed to react with aqueous NaOCl (0.07 mL) in DMF (0.34 mL) to give a crude residue which was purified by flash column chromatography (95:5 pentane/EtOAc) to give the alcohol (5 mg, 59%, 86:14 d^1/d^2, 99:1 e.r.) as a colorless oil.

R*f* = 0.4 (d^2), 0.1 (d^1) (pentane/EtOAc 9:1).

$[\alpha]_D^{25}$: +3 (c = 1.6).

NMR Spectroscopy (*see spectra*):

^{1}H NMR (500 MHz, $CDCl_3$): δ_H 7.37–7.33 (m, 2H, H_F *or* H_G), 7.32–7.24 (m, 2H, H_F *or* H_G), 7.22–7.17 (m,

1H, H_H), 7.15–7.09 (m, 2H, H_Q *or* H_P), 7.01–6.92 (m, 2H, H_Q *or* H_P), 5.13 (dd, J = 7.8, 5.6 Hz, 1H, H_M), 2.29–2.20 (m, 1H, H_I *or* H_K), 2.12 (dd, J = 14.9, 7.8 Hz, 1H, H_L), 2.02–1.90 (m, 2H, H_I *or* H_K), 1.89–1.81 (m, 1H, H_I *or* H_K), 1.77 (dd, J = 14.9, 5.6 Hz, 1H, $H_{L'}$), 1.40 (s, 3H, H_D), 1.27–1.23 (m, 2H, $H_{J, J'}$) ppm;

^{13}C NMR (125 MHz, $CDCl_3$): δ_C 146.4 (C_E), 138.0 (C_N), 137.4 ($C_{N'}$), 128.1 (C_{Ar}), 126.9 (C_{Ar}), 126.2 (C_{Ar}), 125.4 (C_{Ar}), 122.2 (C_{Ar}), 122.1 (C_{Ar}), 84.4 (C_B), 52.6 (C_C *or* C_M), 51.3 (C_C *or* C_M), 44.2 (C_L), 36.0 (C_I *or* C_K), 35.6 (C_I *or* C_K), 23.5 (C_D), 19.2 (C_J) ppm;

Assignments made for the major diastereoisomer.

IR (film): ν_{max} 3498 (br), 3056, 2961, 2877, 1496, 1444, 1374, 1118, 1031 cm^{-1}.

HRMS (ESI): m/z calc'd for $C_{20}H_{22}NaOS_2$ [M+Na]$^+$ 365.1004 found 365.1011.

Chiral HPLC (IA column, 97:3 hexane/IPA, 1 mL/min, rt, 230 nm): 10.4 min (major), 12.3 min (minor), 99:1 e.r.

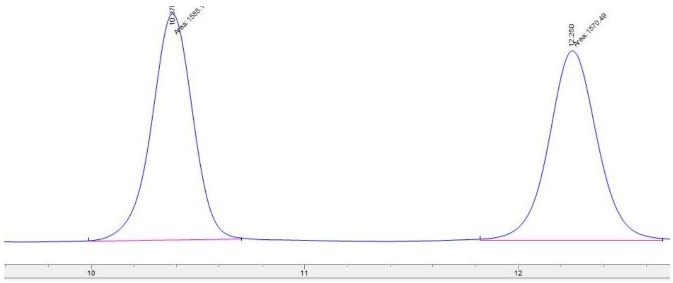

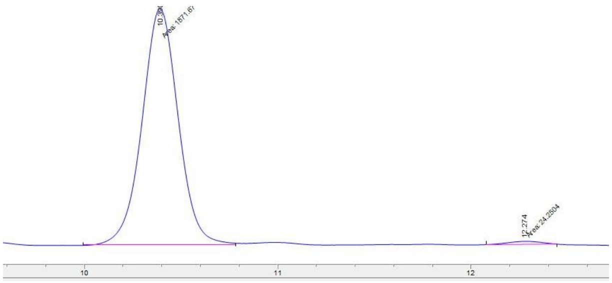

#	Time	Type	Area	Height	Width	Area%	Symmetry
1	10.378	MM	1585.1	117.5	0.2248	50.232	1.041
2	12.25	MM	1570.5	98.5	0.2657	49.768	0.947

#	Time	Type	Area	Height	Width	Area%	Symmetry
1	10.39	MM	1871.7	144	0.2166	98.721	0.961
2	12.274	MM	24.3	2	0.2066	1.279	1.045

2-((1*S*,2*S*)-1-(Bromomethyl)-2-methyl-2-phenylcyclopentyl)-4,4,5,5-tetramethyl-1,3,2-dioxaborolane (3k.d²)

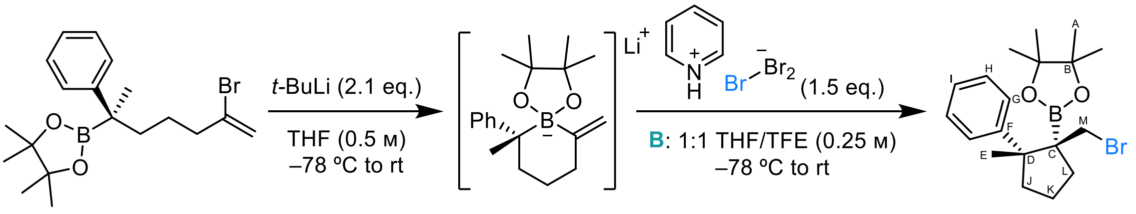

1h **2a** **3k.d²**

According to General Procedure G, vinyl bromide **1h** (76 mg, 0.2 mmol, 1.0 eq.) was allowed to react with *tert*-butyl lithium (1.6 M in pentane, 0.26 mL, 0.42 mmol, 2.1 eq.) and pyridinium tribromide (96 mg, 0.3 mmol, 1.5 eq.) to give a crude residue (>95:5 d.r. by crude ^{1}H NMR) which was purified by flash column chromatography (100:1 pentane/Et_2O) to afford the boronic ester (43 mg, 57%, 5:95< d^1/d^2, 99:1 e.r., 100% e.s.) as a white solid.

R*f* = 0.3 for both diastereomers (pentane/CH_2Cl_2 9:1).

$[\alpha]_D^{25}$: –7 (*c* = 8.6).

NMR Spectroscopy (*see spectra*):

^{1}H NMR (400 MHz, $CDCl_3$): δ_H 7.39–7.33 (m, 2H, H_G), 7.29–7.22 (m, 2H, H_H), 7.20–7.13 (m, 1H, H_I), 4.08

(d, J = 8.6 Hz, 1H, H_M), 3.54 (d, J = 8.6 Hz, 1H, $H_{M'}$), 2.52–2.36 (m, 1H, H_J), 2.28–2.16 (m, 1H, H_L), 2.01–1.79 (m, 4H, $H_{J', K, K', L'}$), 1.37 (s, 3H, H_E), 1.00 (s, 6H, H_A), 0.97 (s, 6H, $H_{A'}$) ppm;

^{13}C NMR (101 MHz, $CDCl_3$): δ_C 148.4 (C_F), 127.8 (C_H), 127.0 (C_G), 126.0 (C_I), 83.4 (C_B), 52.1 (C_D), 41.6 (C_M), 39.7 (C_J), 33.0 (C_L), 25.2 (C_A), 24.7 ($C_{A'}$), 24.0 (C_E), 21.3 (C_K) ppm; C_C not observed due to quadrupolar relaxation;

^{11}B NMR (128 MHz, $CDCl_3$): δ_B 31.5 ppm;

Assignments made for the major diastereoisomer.

IR (film): ν_{max} 2974, 2875, 1444, 1371, 1324, 1237, 1210, 1142, 1032 cm^{-1}.

HRMS (ESI): m/z calc'd for $C_{19}H_{28}O_2{}^{11}BBrNa$ [M+Na]$^+$ 401.1261 found 401.1272.

Chiral HPLC (IB column, 100:0 hexane/IPA, 0.7 mL/min, rt, 254 nm): 7.9 min (minor), 8.2 min (major), 99:1 e.r.

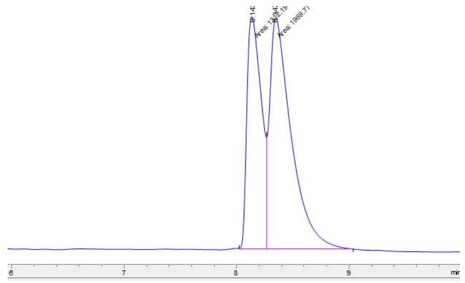

#	Time	Type	Area	Height	Width	Area%	Symmetry
1	8.143	MF	1312.2	147.7	0.148	39.982	0.436
2	8.347	FM	1969.8	146.6	0.224	60.018	0.336

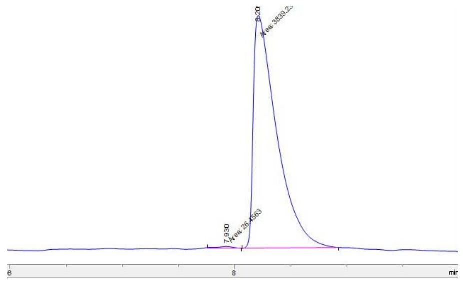

#	Time	Type	Area	Height	Width	Area%	Symmetry
1	7.93	MM	26.5	2.3	0.19	0.684	1.05
2	8.209	MM	3839.2	265.7	0.2408	99.316	0.25

The identity of the minor enantiomeric peak was confirmed by spiking a small amount of the racemic compound into the enantioenriched sample so that the minor enantiomeric peak became more pronounced.

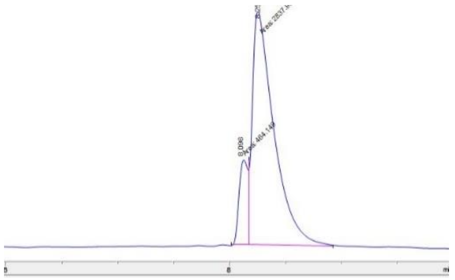

#	Time	Type	Area	Height	Width	Area%	Symmetry
1	8.096	MF	464.1	77.7	0.0996	14.056	0.872
2	8.25	FM	2838	213.8	0.2213	85.944	0.357

Racemic test reaction:

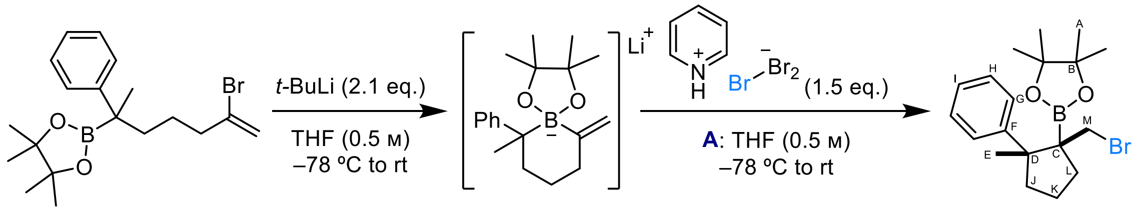

(±)-1h (±)-2a (±)-3k.d[2]

According to General Procedure **E**, vinyl bromide **(±)-1h** (38 mg, 0.1 mmol, 1.0 eq.) was allowed to react with *tert*-butyl lithium (1.8 M in pentane, 0.12 mL, 0.21 mmol, 2.1 eq.) and pyridinium tribromide (48 mg, 0.15 mmol, 1.5 eq.) to give a crude residue (93:7 d.r. by crude ^{1}H NMR) which was purified by flash column chromatography (100:1 pentane/Et_2O) to afford the racemic boronic ester (11 mg, 30%, 7:93 d^1/d^2) as a white solid.

2-((1*S*,2*S*)-1-(Iodomethyl)-2-methyl-2-phenylcyclopentyl)-4,4,5,5-tetramethyl-1,3,2-dioxaborolane (3l.d^2)

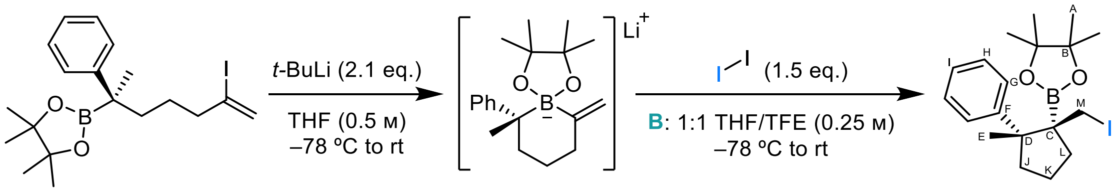

1a **2a** **3l.d^2**

According to General Procedure **G**, vinyl iodide **1a** (85 mg, 0.2 mmol, 1.0 eq.) was allowed to react with *tert*-butyl lithium (1.7 M in pentane, 0.25 mL, 0.42 mmol, 2.1 eq.) and iodine (76 mg in 0.3 mL THF, 0.3 mmol, 1.5 eq.) to give a crude residue (>95:5 d.r. by crude ^{1}H NMR) which was purified by flash column chromatography (4:1 pentane/CH_2Cl_2) to afford the boronic ester (58 mg, 68%, 5:95< d^1/d^2, 99:1 e.r., 100% e.s.) as a white solid. Note: trace elimination product was observed.

R*f* = 0.3 (d^2), 0.1 (d^1) (pentane/CH_2Cl_2 9:1).

$[\alpha]_D^{25}$: –7 (*c* = 9.8).

NMR Spectroscopy (*see spectra*):

> **^{1}H NMR** (400 MHz, $CDCl_3$): δ_H 7.32–7.24 (m, 2H, H_G), 7.23–7.13 (m, 2H, H_H), 7.13–7.03 (m, 1H, H_I), 3.82 (d, *J* = 8.2 Hz, 1H, H_M), 3.31 (d, *J* = 8.2 Hz, 1H, $H_{M'}$), 2.45–2.34 (m, 1H, H_J), 2.23–2.10 (m, 1H, H_L), 1.91–1.73 (m, 4H, $H_{J', K, K', L'}$), 1.27 (s, 3H, H_E), 0.92 (s, 12H, H_A) ppm;
>
> **^{13}C NMR** (101 MHz, $CDCl_3$): δ_C 148.3 (C_F), 127.8 (C_G), 126.8 (C_H), 126.0 (C_I), 83.4 (C_B), 51.6 (C_D), 39.7 (C_J), 36.0 (C_L), 25.3 (C_A), 24.9 ($C_{A'}$), 23.9 (C_E), 20.9 (C_K), 16.9 (C_M) ppm; C_C not observed due to quadrupolar relaxation;
>
> **^{11}B NMR** (128 MHz, $CDCl_3$): δ_B 31.6 ppm;
>
> *Assignments made for the major diastereoisomer.*

IR (film): ν_{max} 2922, 2853, 1463, 1372, 1319, 1140 cm^{-1}.

HRMS (ESI): m/z calc'd for $C_{19}H_{28}O_2{}^{11}BNaI$ $[M+Na]^+$ 449.1123 found 449.1136.

Chiral SFC (Whelk-O1 column, iso 2% hexane, 2 mL/min, 100 bar): 17.5 min (minor), 18.4 min (major), 99:1 e.r.

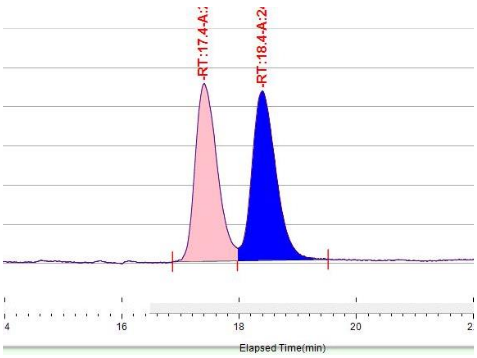

File	Signal	Peak Name	Area Percent	Area	Retention Time	Height
MEF-D-28-2rac_12-01-2021 17-21-21.tta	MaxAbsorbance	Peak1	48.9494	2353.8686	17.4 min	90.7954
		Peak2	51.0506	2454.9097	18.4 min	86.5248

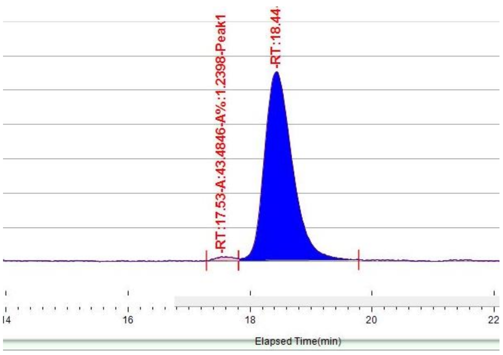

File	Signal	Peak Name	Area Percent	Area	Retention Time	Height
MEF-D-50ee_12-01-2021 18-03-11.tta	MaxAbsorbance	Peak1	1.2398	43.4846	17.53 min	2.305
		Peak2	98.7602	3464.0186	18.44 min	109.8198

Racemic test reaction:

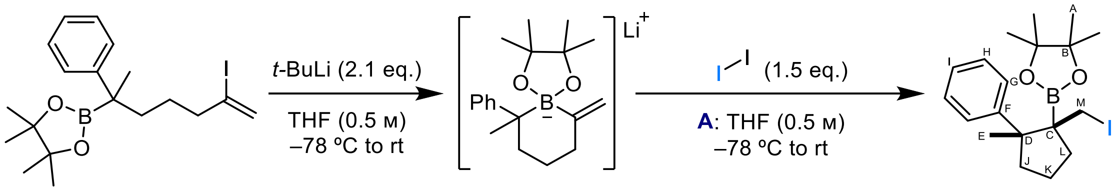

(±)-1a (±)-2a (±)-3l.d²

According to General Procedure E, vinyl iodide **(±)-1a** (43 mg, 0.1 mmol, 1.0 eq.) was allowed to react with *tert*-butyl lithium (1.7 M in pentane, 0.13 mL, 0.21 mmol, 2.1 eq.) and iodine (38 mg in 0.15 mL THF, 0.15 mmol, 1.5 eq.) to give a crude residue (93:7 d.r. by crude ^{1}H NMR) which was purified by flash column chromatography (4:1 pentane/CH_2Cl_2) to afford the racemic boronic ester (28 mg, 66%, 7:93 d^1/d^2) as a white solid. Note: trace elimination product was observed.

4,4,5,5-Tetramethyl-2-((1*S*,2*S*)-2-methyl-2-phenyl-1-((phenylselanyl)methyl)cyclopentyl)-1,3,2-dioxaborolane (3m.d²)

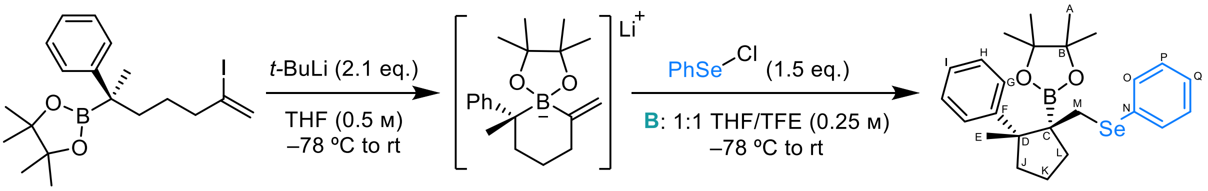

1a **2a** **3m.d²**

According to a modified General Procedure G, vinyl iodide **1a** (85 mg, 0.2 mmol, 1.0 eq.) was allowed to react with *tert*-butyl lithium (1.7 M in pentane, 0.25 mL, 0.42 mmol, 2.1 eq.) and phenylselenyl chloride (58 mg in 0.15 mL THF, 0.3 mmol, 1.5 eq.) with the addition of *degassed* TFE to give a crude residue (85:15 d.r. by crude ^{1}H NMR) which was purified by flash column chromatography (1000:1 to 100:1 pentane/Et_2O) to afford the boronic ester (35 mg, 38%, 15:85 d^1/d^2, 99:1 e.r., 100% e.s.) as a colorless oil.

R*f* = 0.3 (d²), 0.1 (d¹) (pentane/CH_2Cl_2 9:1).

$[\alpha]_D^{25}$**:** –7 (*c* = 9.8).

NMR Spectroscopy (*see spectra*):

^{1}H NMR (400 MHz, $CDCl_3$): δ_H 7.61–7.56 (m, 2H, H_O), 7.43–7.38 (m, 2H, H_G), 7.30–7.21 (m, 5H, $H_{H, P, Q}$), 7.19–7.12 (m, 1H, H_I), 3.60 (d, *J* = 10.1 Hz, 1H, H_M), 3.16 (d, *J* = 10.1 Hz, 1H, $H_{M'}$), 2.50–2.37 (m, 1H, H_J), 2.28–2.15 (m, 1H, H_L), 1.93–1.67 (m, 4H, $H_{J', K, K', L'}$), 1.36 (s, 3H, H_E), 1.02 (s, 6H, H_A), 0.97 (s, 6H, $H_{A'}$) ppm;

^{13}C NMR (101 MHz, $CDCl_3$): δ_C 148.9 (C_F), 132.8 (C_O), 128.9 (C_H *or* C_P), 127.7 (C_H *or* C_P), 127.0 (C_G), 126.5 (C_Q), 125.8 (C_I), 83.2 (C_B), 52.1 (C_D), 39.2 (C_J), 36.0 (C_M), 33.5 (C_L), 25.1 (C_A), 24.9 ($C_{A'}$), 24.2 (C_E), 21.6 (C_K) ppm; C_C not observed due to quadrupolar relaxation;

^{11}B NMR (128 MHz, $CDCl_3$): δ_B 32.8 ppm;

Assignments made for the major diastereoisomer.

IR (film): ν_{max} 2958, 2926, 1579, 1477, 1371, 1315, 1142 cm^{-1}.

HRMS (APCI): m/z calc'd for $C_{25}H_{33}{}^{11}BO_2Se$ $[M]^+$ 457.1812 found 457.1799.

Chiral SFC (Whelk-O1 column, iso 2% hexane, 4 mL/min, 125 bar): 13.8 min (d^2, minor enantiomer), 14.6 min (d^1, major enantiomer), 17.8 min (d^2, major enantiomer), 19.1 min (d^1, minor enantiomer), 99:1 e.r.

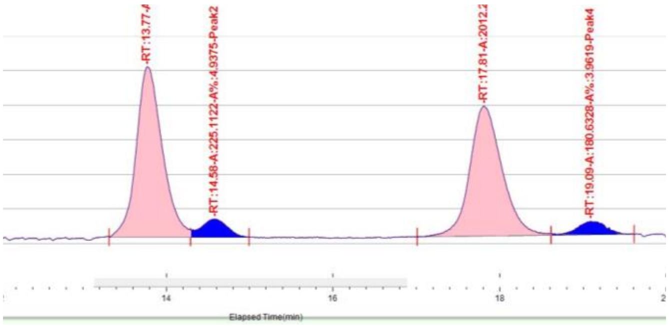

File	Signal	Peak Name	Area Percent	Area	Retention Time	Height
MEF-D-42rac_12-01-2021 16-39-36.tta	MaxAbsorbance	Peak1	46.9652	2141.2438	13.77 min	98.7685
		Peak2	4.9375	225.1122	14.58 min	10.4978
		Peak3	44.1354	2012.2253	17.81 min	75.3307
		Peak4	3.9619	180.6328	19.09 min	7.7193

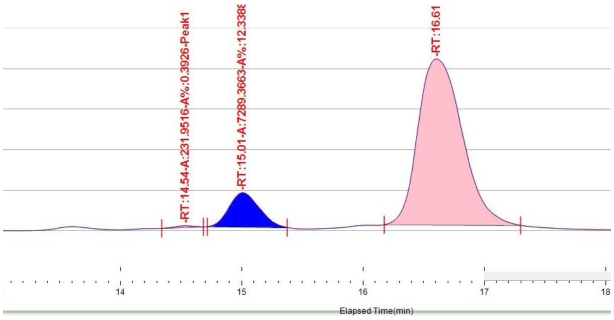

File	Signal	Peak Name	Area Percent	Area	Retention Time	Height
MEF-E-14-2ee_23-06-2021 09-42-17.tta	MaxAbsorbance	Peak1	0.3926	231.9516	14.54 min	21.4512
		Peak2	12.3388	7289.3663	15.01 min	423.939
		Peak3	87.2686	51555.3807	16.61 min	2048.6532

Racemic test reaction:

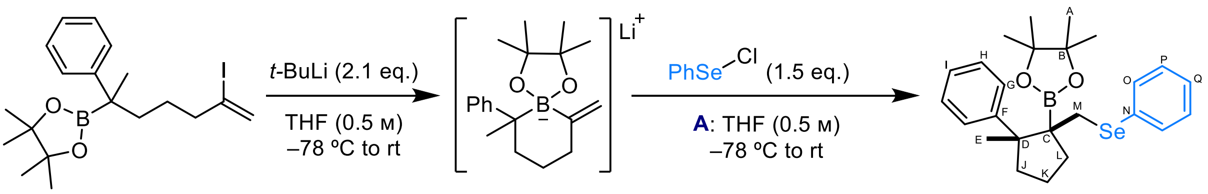

According to General Procedure **E**, vinyl iodide **(±)-1a** (49 mg, 0.12 mmol, 1.0 eq.) was allowed to react with *tert*-butyl lithium (1.7 M in pentane, 0.14 mL, 0.24 mmol, 2.1 eq.) and phenylselenyl chloride (33 mg in 0.17 mL THF, 0.17 mmol, 1.5 eq.) to give a crude residue (56:44 d.r. by crude ^{1}H NMR) which was purified by flash column chromatography (20:1 pentane/Et_2O) to afford the racemic boronic ester (10 mg, 20%, 44:56 d^1/d^2) as a colorless oil.

4,4,5,5-Tetramethyl-2-((1*S*,2*S*)-2-methyl-2-phenyl-1-((phenylthio)methyl)cyclopentyl)-1,3,2-dioxaborolane (3n.d^2)

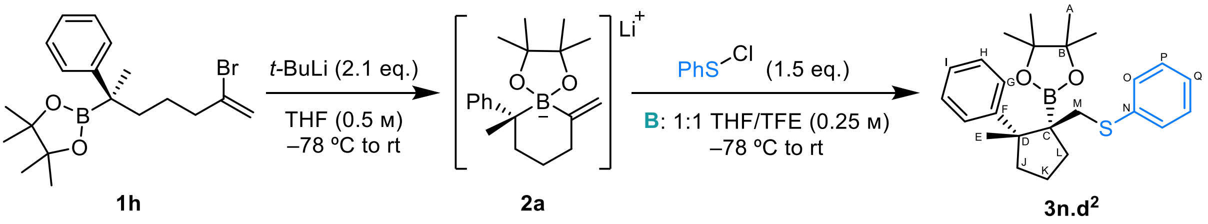

According to General Procedure **G**, vinyl bromide **1h** (76 mg, 0.2 mmol, 1.0 eq.) was allowed to react with *tert*-butyl lithium (1.6 M in pentane, 0.27 mL, 0.42 mmol, 2.1 eq.) and freshly prepared phenylsulfenyl chloride[13] (43 mg in 0.3 mL THF, 0.3 mmol, 1.5 eq.) to give a crude residue (83:17 d.r. by crude ^{1}H NMR) which was purified by flash column chromatography (200:1 to 100:1 pentane/Et_2O) to afford the boronic ester (62 mg, 76%, 17:83 d^1/d^2, 99:1 e.r., 100% e.s.) as a white solid.

The e.r. of boronic ester **3n.d^2** could not be determined by chiral HPLC/SFC. Instead, the boronic ester was oxidised and the e.r. of alcohol **3n'.d^2** was determined.

R*f* = 0.6 for both diastereomers (pentane/Et_2O 95:5).

$[\alpha]_D^{25}$: +37 (*c* = 5.2).

NMR Spectroscopy (*see spectra*):

^{1}H NMR (400 MHz, $CDCl_3$): δ_H 7.45–7.34 (m, 4H, $H_{G,O}$), 7.31–7.18 (m, 4H, $H_{H,P}$), 7.20–7.10 (m, 2H, $H_{I,Q}$), 3.50 (d, J = 10.4 Hz, 1H, H_M), 3.15 (d, J = 10.4 Hz, 1H, $H_{M'}$), 2.46–2.32 (m, 1H, H_J), 2.21–2.09 (m, 1H, H_L), 1.95–1.70 (m, 4H, $H_{J',K,K',L'}$), 1.41 (s, 3H, H_E), 1.02 (s, 6H, H_A), 0.94 (s, 6H, $H_{A'}$) ppm;

^{13}C NMR (101 MHz, $CDCl_3$): δ_C 149.1 (C_F), 139.0 (C_N), 129.5 (C_G *or* C_O), 128.8 (C_H *or* C_P), 127.8 (C_H *or* C_P), 127.2 (C_G *or* C_O), 125.9 (C_I *or* C_Q), 125.7 (C_I *or* C_Q), 83.3 (C_B), 52.0 (C_D), 40.7 (C_M), 39.3 (C_J), 32.1 (C_L), 25.3 (C_A), 24.9 ($C_{A'}$), 24.3 (C_E), 22.0 (C_K) ppm; C_C not observed due to quadrupolar relaxation;

^{11}B NMR (128 MHz, $CDCl_3$): δ_B 32.2 ppm;

Assignments made for the major diastereoisomer.

IR (film): ν_{max} 2974, 2875, 1583, 1480, 1370, 1317,1210, 1141, 1026 cm^{-1}.

HRMS (ESI): m/z calc'd for $C_{25}H_{33}{}^{11}BNaO_2S$ $[M+Na]^+$ 431.2191 found 431.2192.

Racemic test reaction:

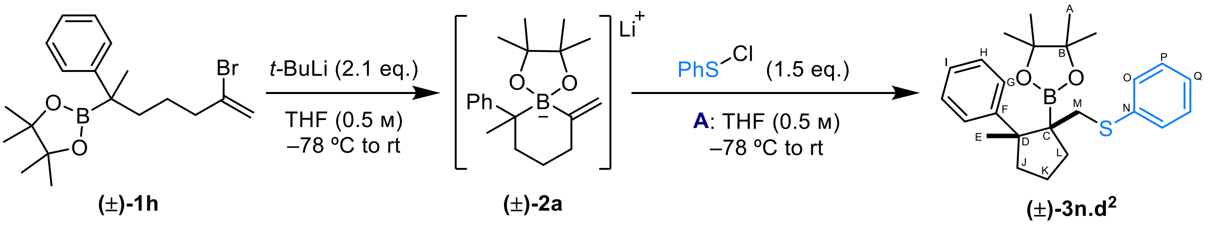

According to General Procedure E, vinyl bromide **(±)-1h** (38 mg, 0.1 mmol, 1.0 eq.) was allowed to react with

tert-butyl lithium (1.6 м in pentane, 0.13 mL, 0.21 mmol, 2.1 eq.) and phenylsulfenyl chloride[13] (22 mg in 0.15 mL THF, 0.15 mmol, 1.5 eq.) to give a crude residue (82:18 d.r. by crude ^{1}H NMR) which was purified by flash column chromatography (200:1 to 100:1 pentane/Et_2O) to afford the racemic boronic ester (18 mg, 44%, 18:82 d^1/d^2) as a white solid.

(1*R*,2*R*)-2-Methyl-2-phenyl-1-((phenylthio)methyl)cyclopentan-1-ol (3n'.d^2)

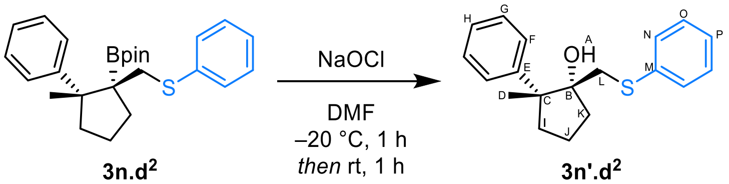

3n.d^2 **3n'.d^2**

According to General Procedure H, boronic ester **3n.d^2** (19 mg, 0.05 mmol, 17:83 d^1/d^2, 1.0 eq.) was allowed to react with aqueous NaOCl (0.14 mL) in DMF (0.65 mL) to give a crude residue which was purified by flash column chromatography (95:5 pentane/EtOAc) to give the alcohol (4 mg, 30%, 17:83 d^1/d^2, 99:1 e.r.) as a colorless oil, along with recovered starting material (8 mg, 41%). The alcohol was air-sensitive, and thus full characterisation was not obtained.

Chiral HPLC (IA column, 99:1 hexane/IPA, 1 mL/min, rt, 254 nm): 6.4 min (minor), 6.9 min (major), 99:1 e.r.

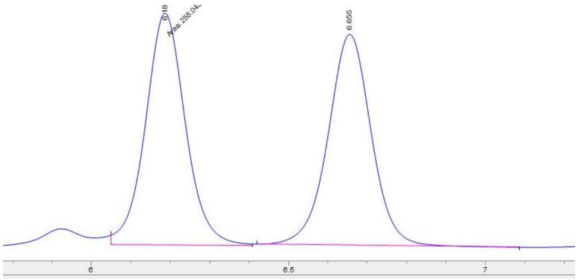

#	Time	Type	Area	Height	Width	Area%	Symmetry
1	6.186	MM	258	36.4	0.1182	50.661	0.936
2	6.655	BB	251.3	33	0.1154	49.339	0.985

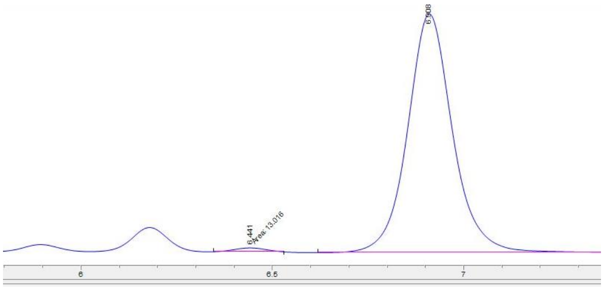

#	Time	Type	Area	Height	Width	Area%	Symmetry
1	6.441	MM	13	2.2	0.0989	1.233	1.052
2	6.908	BB	1042.9	128	0.1231	98.767	0.897

2-((1*S*,2*S*)-1,2-Dimethyl-2-phenylcyclopentyl)-4,4,5,5-tetramethyl-1,3,2-dioxaborolane (3o.d^2)

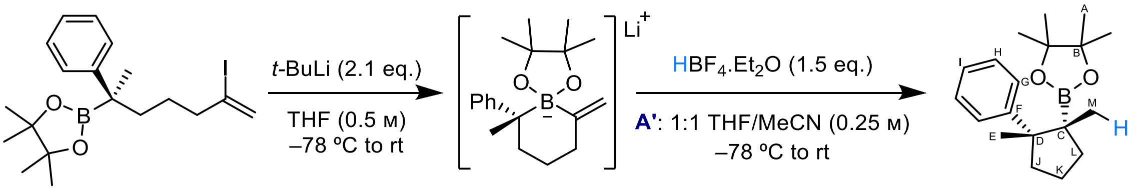

1a **2a** **3o.d^2**

According to General Procedure F, vinyl iodide **1a** (85 mg, 0.2 mmol, 1.0 eq.) was allowed to react with *tert*-butyl lithium (1.5 м in pentane, 0.28 mL, 0.42 mmol, 2.1 eq.) and tetrafluoroboric acid diethyl ether complex (41 µL, 0.3 mmol, 1.5 eq.) to give a crude residue (86:14 d.r. by GCMS) which was purified by flash column chromatography (9:1 pentane/CH_2Cl_2) to afford the boronic ester (32 mg, 53%, 14:86 d^1/d^2, 99:1 e.r., 100% e.s.) as a colorless oil.

The e.r. of boronic ester **3o.d²** could not be determined by chiral HPLC/SFC. Instead, the boronic ester was oxidised and the e.r. of alcohol **3o'.d²** was determined.

R*f* = 0.2 for both diastereomers (pentane/CH_2Cl_2 9:1).

$[\alpha]_D^{25}$**:** –10 (*c* = 6.0).

NMR Spectroscopy (*see spectra*):

¹H NMR (400 MHz, $CDCl_3$): δ_H 7.43–7.36 (m, 2H, H_G), 7.25–7.18 (m, 2H, H_H), 7.18–7.07 (m, 1H, H_I), 2.59–2.43 (m, 1H, H_J), 2.04–1.93 (m, 1H, H_L), 1.90–1.73 (m, 3H, $H_{J', K, K'}$), 1.54–1.42 (m, 1H, $H_{L'}$), 1.25 (s, 3H, H_E), 1.19 (s, 3H, H_M), 0.97 (s, 6H, H_A), 0.93 (s, 6H, $H_{A'}$) ppm;

¹³C NMR (101 MHz, $CDCl_3$): δ_C 150.5 (C_F), 127.6 (C_H), 126.7 (C_G), 125.3 (C_I), 82.4 (C_B), 50.2 (C_D), 39.2 (C_J), 36.7 (C_L), 24.9 (C_E), 24.6 (C_A), 24.5 ($C_{A'}$), 21.4 (C_K), 20.3 (C_M) ppm; C_C not observed due to quadrupolar relaxation;

¹¹B NMR (128 MHz, $CDCl_3$): δ_B 33.9 ppm;

Assignments made for the major diastereoisomer.

IR (film): ν_{max} 2974, 1444, 1355, 1306, 1146 cm⁻¹.

HRMS (ESI): m/z calc'd for $C_{19}H_{30}{}^{11}BO_2$ $[M+H]^+$ 301.2337 found 301.2330.

Racemic test reaction:

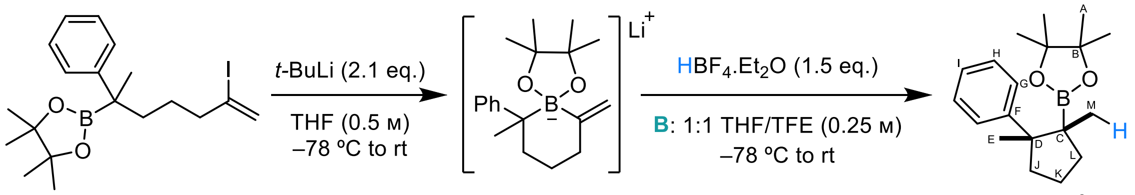

(±)-1a **(±)-2a** **(±)-3o.d²**

According to General Procedure **G**, vinyl iodide **(±)-1a** (80 mg, 0.19 mmol, 1.0 eq.) was allowed to react with *tert*-butyl lithium (1.6 M in pentane, 0.27 mL, 0.42 mmol, 2.1 eq.) and tetrafluoroboric acid diethyl ether complex (41 μL, 0.3 mmol, 1.5 eq.) to give a crude residue (73:27 d.r. by GCMS) which was purified by flash column chromatography (9:1 pentane/CH_2Cl_2) to afford the boronic ester (17 mg, 29%, 27:73 d¹/d²) as a colorless oil.

(1*R*,2*R*)-1,2-Dimethyl-2-phenylcyclopentan-1-ol (3o'.d²)

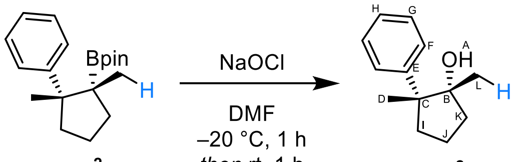

3o.d² **3o'.d²**

According to General Procedure **H**, boronic ester **3o.d²** (14 mg, 0.05 mmol, 14:86 d¹/d², 1.0 eq.) was allowed to react with aqueous NaOCl (0.14 mL) in DMF (0.65 mL) to give a crude residue which was purified by flash column chromatography (pentane then 3:1 pentane/Et_2O) to give alcohol **3o'.d²** (d²: 4 mg; d¹: <0.5 mg; 45%, 99:1 e.r.) as a colorless oil.

R_f = 0.4 (d^2), 0.3 (d^1) (pentane/EtOAc 9:1).

$[\alpha]_D^{25}$**:** –24 (*c* = 2.2).

NMR Spectroscopy (*see spectra*):

^{1}H NMR (400 MHz, $CDCl_3$): δ_H 7.53–7.47 (m, 2H, H_F), 7.41–7.31 (m, 2H, H_G), 7.30–7.21 (m, 1H, H_H), 2.79–2.67 (m, 1H, H_I), 1.99–1.86 (m, 3H, $H_{J, K, K'}$), 1.86–1.73 (m, 1H, $H_{J'}$), 1.69–1.59 (m, 1H, $H_{I'}$), 1.32 (s, 3H, H_D), 1.26 (s, 3H, H_L), 0.90 (s, 1H, H_A) ppm;

^{13}C NMR (101 MHz, $CDCl_3$): δ_C 143.5 (C_E), 128.2 (C_G), 127.8 (C_F), 126.5 (C_H), 82.5 (C_B), 52.9 (C_C), 38.4 (C_K), 35.8 (C_I), 24.2 (C_D), 22.9 (C_L), 19.3 (C_J) ppm;

Assignments made for the major diastereoisomer.

IR (film): ν_{max} 3446 (br), 2962, 2928, 1600, 1497, 1443, 1371, 1136 cm^{-1}.

HRMS (ESI): m/z calc'd for $C_{13}H_{18}ONa$ $[M+Na]^+$ 213.1250 found 213.1260.

Chiral HPLC (IA column, 97:3 hexane/IPA, 0.5 mL/min, rt, 230 nm): 13.1 min (minor), 13.7 min (major), 99:1 e.r.

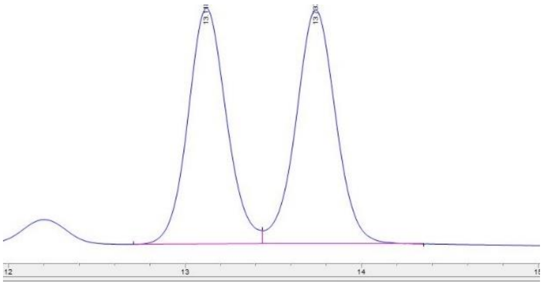

#	Time	Type	Area	Height	Width	Area%	Symmetry
1	13.118	BV	954.4	62.7	0.236	49.418	0.897
2	13.737	VB	976.9	62.2	0.2373	50.582	0.973

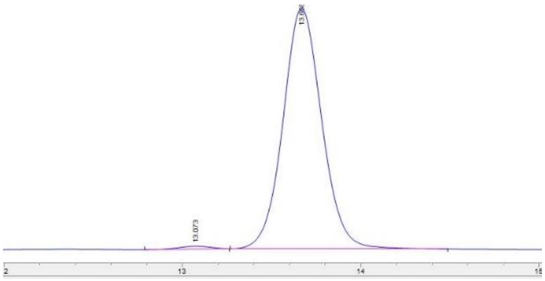

#	Time	Type	Area	Height	Width	Area%	Symmetry
1	13.073	BB	24.7	2	0.1471	0.958	1.151
2	13.668	BB	2559.3	169.2	0.231	99.042	0.907

2.3.3.2 Unsuccessful electrophiles

A range of electrophiles proved unsuccessful under reaction conditions A, yielding no or very small amounts of the desired product (Scheme S2).

The formation of boronic ester **3l.d**[2] in cases where the vinyl iodide starting material was used has been tentatively attributed to the formation of an electrophilic iodinating species under the reaction conditions, possibly from the oxidation of the iodide anion, generated in the lithium-halogen exchange step, by the electrophile.[14,15] In each case, the reaction mixture had a brown color following overnight stirring, which was lost on adding saturated aqueous $Na_2S_2O_3$, indicative of the presence of elemental iodide.[16]

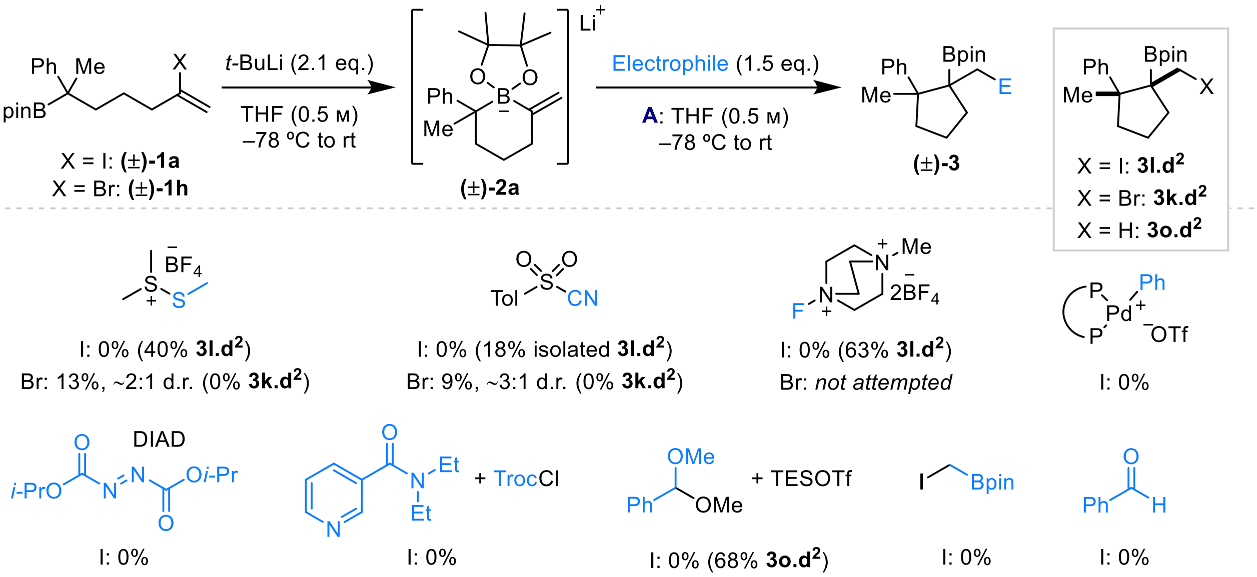

Scheme S2: Unsuccessful electrophiles under conditions A.

Yields determined by ^{1}H NMR analysis using CH_2Br_2 as an internal standard unless otherwise indicated.

Exchanging the vinyl iodide for a vinyl bromide increased the scope of competent electrophiles under the reaction conditions (conditions B), removing the competing iodination pathway (Scheme S3).

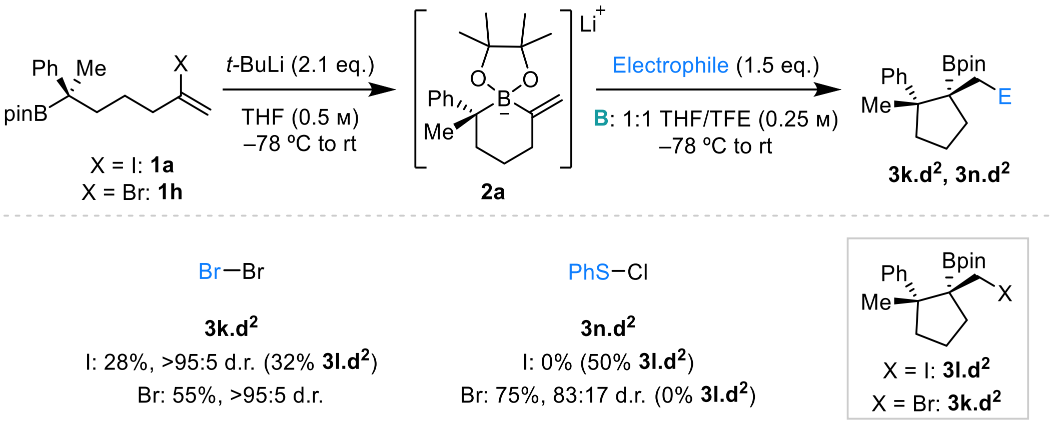

Scheme S3: The influence of the identity of the vinyl halide on product formation.

Yields determined by ^{1}H NMR analysis using CH_2Br_2 as an internal standard.

2.4. Application to the Total Synthesis of (+)-Herbertene-1,14-diol

1-(2-Methoxy-5-methylphenyl)ethan-1-one (7)

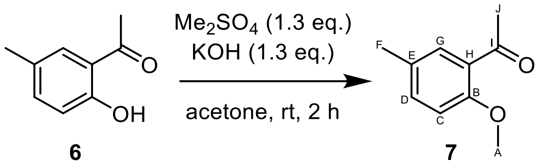

According to a modified literature procedure,[8] 1-(2-hydroxy-5-methylphenyl)ethan-1-one **6** (5.00 g, 33 mmol, 1 eq.) was solved in acetone (70 mL) at rt and potassium hydroxide (2.43 g, 43 mmol, 1.3 eq.) was added followed by dimethyl sulfate (4.1 mL, 43 mmol, 1.3 eq.) dropwise. The reaction mixture was stirred at rt for 2 h (and followed by TLC) whereupon a 1 M aqueous solution of NaOH (100 mL) was added. The aqueous phase was extracted with Et_2O (3 × 100 mL) and the combined organics were washed with brine, dried over $MgSO_4$ and concentrated *in vacuo*. The crude product was purified by flash column chromatography (pentane/EtOAc 20:1) to give ketone **7** (5.17 g, 32 mmol) as a colourless oil in 95% yield.

NMR Spectroscopy (*see spectra*):

^{1}H NMR (400 MHz, $CDCl_3$): δ_H 7.53 (d, *J* = 2.8 Hz, 1H, H_G), 7.26 (dd, *J* = 8.4, 2.1 Hz, 1H, H_D), 6.86 (d, *J* = 8.4 Hz, 1H, H_C), 3.88 (s, 3H, H_A), 2.60 (s, 3H, H_J), 2.30 (s, 3H, H_F) ppm;

^{13}C NMR (101 MHz, $CDCl_3$): δ_C 200.1 (C_I), 157.0 (C_B), 134.1 (C_D), 130.6 (C_G), 129.9 (C_E), 128.0 (C_H), 111.6 (C_C), 55.6 (C_A), 31.8 (C_J), 20.2 (C_F) ppm.

All recorded spectroscopic data matched those previously reported in the literature.[17]

(*S*)-1-(2-Methoxy-5-methylphenyl)ethan-1-ol (8)

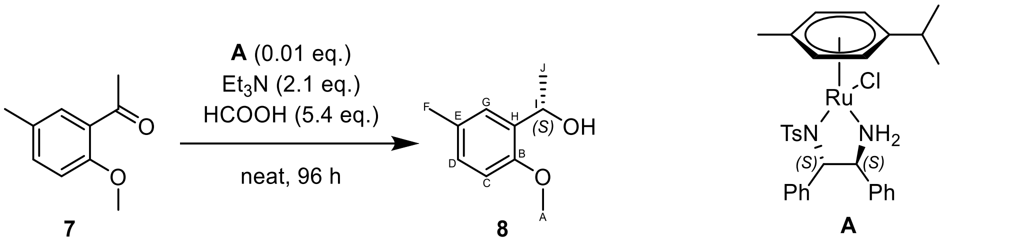

RuCl(*p*-cymene)[(*S*,*S*)-Ts-DPEN]

According to a modified literature procedure,[8] formic acid (6.1 mL, 162 mmol, 5.4 eq.) was added to triethylamine (8.8 mL, 63 mmol, 2.1 eq.) at 0 °C. The reaction mixture was warmed to room temperature whereupon ketone **7** (4.92 g, 30 mmol, 1 eq.) was added dropwise, followed by RuCl(*p*-cymene)[(*S*,*S*)-Ts-DPEN] **A** (0.190 g, 0.3 mmol, 0.01 eq.) as one portion. Nitrogen was bubbled through the reaction mixture over 96 h, at which point the reaction was deemed to be complete by TLC. The reaction mixture was quenched with water (100 mL) and EtOAc (100 mL) was added. The organic layer was separated, and the aqueous phase was extracted with Et_2O (3 × 100 mL). The combined organics were washed with brine, dried over $MgSO_4$ and concentrated *in vacuo*. The crude product was purified by flash column chromatography (pentane/EtOAc 20:1) to give alcohol **8** (4.72 g, 28 mmol, 96:4 e.r.) as an off-white solid in 95% yield.

R_f = 0.2 (pentane/EtOAc 9:1).

$[\alpha]_D^{25}$: –25 (c = 2.1).

NMR Spectroscopy (*see spectra*):

^{1}H NMR (400 MHz, $CDCl_3$): δ_H 7.08–7.05 (m, 1H, H_G), 6.96 (ddd, J = 8.4, 2.4, 0.8 Hz, 1H, H_D), 6.71 (d, J = 8.3 Hz, 1H, H_C), 4.98 (q, J = 6.5 Hz, 1H, H_I), 3.76 (s, 3H, H_A), 2.54 (br s, 1H, OH), 2.22 (s, 3H, H_F), 1.43 (d, J = 6.5 Hz, 3H, H_J) ppm;

^{13}C NMR (101 MHz, $CDCl_3$): δ_C 154.5 (C_B), 133.1 (C_H), 130.0 (C_E), 128.4 (C_D), 126.9 (C_G), 110.5 (C_C), 66.7 (C_I), 55.4 (C_A), 23.0 (C_J), 20.6 (C_F) ppm.

IR (film): ν_{max} 3380 (OH), 2969, 2927, 1612, 1501, 1464, 1287, 1244, 1181, 1157, 1140, 1077, 1028 cm^{-1}.

HRMS (APCI): m/z calc'd for $C_{10}H_{13}O$ $[M+H–H_2O]^+$ 149.0961 found 149.0960.

Chiral HPLC (IA column, 98:2 hexane/IPA, 1 mL/min, rt, 254 nm): 21.5 min (major), 22.7 (minor), 96:4 e.r.

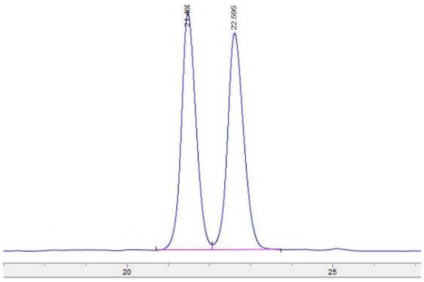

#	Time	Type	Area	Height	Width	Area%	Symmetry
1	21.46	BV	4878.6	196	0.3559	49.957	0.804
2	22.595	VV R	4886.9	179.6	0.4069	50.043	0.77

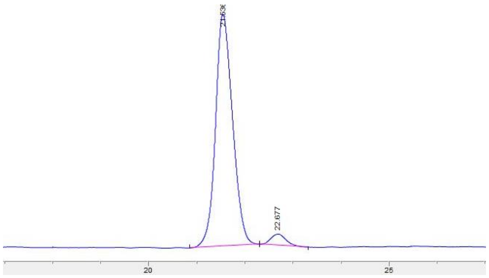

#	Time	Type	Area	Height	Width	Area%	Symmetry
1	21.536	BV R	5806.1	235	0.3542	95.814	0.756
2	22.677	VV R	253.7	11.4	0.261	4.186	0.818

(*S*)-1-(2-Methoxy-5-methylphenyl)ethyl diisopropylcarbamate (9)

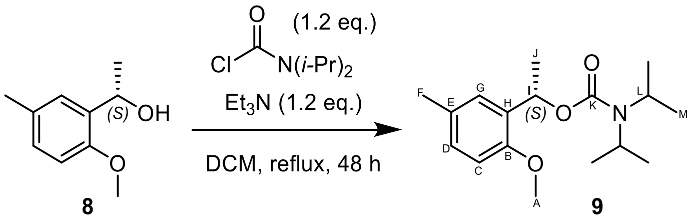

According to a modified literature procedure,[8] alcohol **8** (4.16 g, 25 mmol, 1.0 eq.) was added to a solution of *N*,*N*-diisopropylcarbamoyl chloride (4.91 g, 30 mmol, 1.2 eq.) and triethylamine (4.2 mL, 30 mmol, 1.2 eq.) in dichloromethane (52 mL) and the solution was heated to reflux for 48 h, after which time a further portion of *N*,*N*-diisopropylcarbamoyl chloride (4.91 g, 30 mmol, 1.2 eq.) and triethylamine (4.2 mL, 30 mmol, 1.2 eq.) were added. Following an additional 48 h at reflux the reaction was deemed complete by TLC and the reaction mixture was cooled to rt. Water (100 mL) was added and the organic layer separated. The aqueous phase was extracted with dichloromethane (3 x 100 mL). The organic phases were combined, dried over $MgSO_4$ and concentrated *in vacuo*. The crude product was purified by flash column chromatography (pentane/EtOAc 9:1) to give carbamate **9** (6.87 g, 23 mmol) as a colourless, amorphous solid in 94% yield

R_f = 0.4 (pentane/EtOAc 9:1).

$[\alpha]_D^{25}$: +2 (c = 2.1).

NMR Spectroscopy (<u>*see spectra*</u>):

^{1}H NMR (400 MHz, $CDCl_3$): δ_H 7.14 (d, J = 2.3 Hz, 1H, H_G), 7.01 (dd, J = 8.3, 2.3 Hz, 1H, H_D), 6.75 (d, J = 8.3 Hz, 1H, H_C), 6.14 (d, J = 6.4 Hz, 1H, H_I), 4.19–3.62 (br m, 2H, H_L), 3.80 (s, 3H, H_A), 2.28 (s, 3H, H_F), 1.49 (d, J = 6.4 Hz, 3H, H_J), 1.38–1.06 (br s, 12H, H_M) ppm;

^{13}C NMR (101 MHz, $CDCl_3$): δ_C 155.1 (C_K), 153.9 (C_B), 131.4 (C_H), 129.5 (C_E), 128.4 (C_G), 126.8 (C_D), 110.6 (C_C), 68.0 (C_I), 55.5 (C_A), 21.8 (C_J), 21.1 (br, C_M), 20.7 (C_F) ppm.

IR (film): ν_{max} 2975, 2934, 1616 (C=O), 1496, 1461, 1370, 1341, 1305, 1236, 1142, 1110 cm^{-1}.

HRMS (ESI): m/z calc'd for $C_{17}H_{27}NNaO_3$ $[M+Na]^+$ 316.1883 found 316.1886.

(*S*)-2-(6-Iodo-2-(2-methoxy-5-methylphenyl)hept-6-en-2-yl)-4,4,5,5-tetramethyl-1,3,2-dioxaborolane (1i)

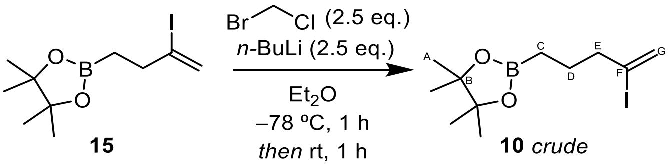

According to a combination of modified literature procedures,[2,8] boronic ester **15** (0.790 g, 2.6 mmol, 1.0 eq.) was solved in Et_2O (20 mL) and cooled to –78 °C (dry ice/acetone). Bromochloromethane (0.42 mL, 6.4 mmol, 2.5 eq.) was added dropwise to the solution, followed by *n*-BuLi (1.6 M in hexanes; 4.0 mL, 6.4 mmol, 2.5 eq.) *via* syringe pump (0.08 mL/min). Following the addition, the reaction mixture was stirred at –78 °C for 1 h and then warmed to rt and stirred at this temperature for 1 h. The reaction mixture was filtered through a silica plug (eluting Et_2O) and concentrated *in vacuo*. Crude boronic ester **10** was used without further purification.

NMR Spectroscopy:

^{1}H NMR (400 MHz, $CDCl_3$): δ_H 6.00 (apparent q, J = 1.4 Hz, 1H, H_G), 5.68 (apparent dd, J = 1.4, 0.7 Hz, 1H, $H_{G'}$), 2.40 (tdd, J = 7.3, 1.4, 0.5 Hz, 2H, H_E), 1.67–1.58 (m, 2H, H_D), 1.25 (s, 12H, H_A), 0.82–0.74 (m, 2H, H_C) ppm;

^{13}C NMR (101 MHz, $CDCl_3$): δ_C 125.4 (C_G), 112.4 (C_F), 83.0 (C_B), 47.7 (C_E), 24.8 (C_A), 23.5 (C_D) ppm. C_C not observed due to quadrupolar relaxation.

All recorded spectroscopic data matched those previously reported in the literature.[2]

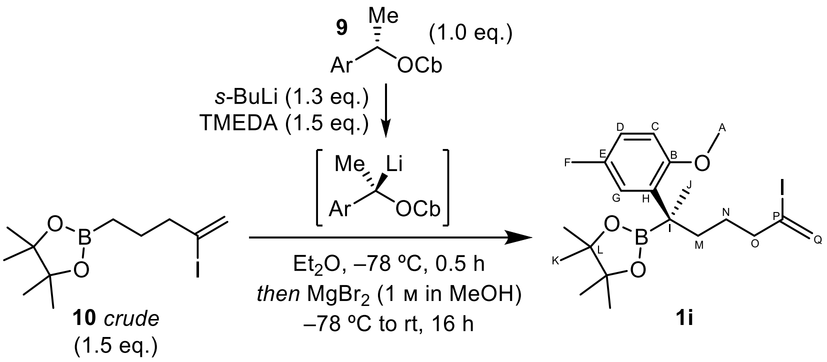

Diisopropylcarbamate **9** (0.509 g, 1.7 mmol, 1.0 eq.) was solved in anhydrous Et_2O (7 mL) and the solution

cooled to –78 °C (dry ice/acetone). TMEDA (0.38 mL, 2.6 mmol, 1.5 eq.) was added dropwise to the solution, followed by *s*-BuLi (1.3 M in cyclohexane/hexane 92:8; 1.7 mL, 2.3 mmol, 1.3 eq.) dropwise. The reaction mixture was stirred at –78 °C for 30 minutes whereupon a 1 M solution in Et_2O of the crude boronic ester (1.5 eq.) was added dropwise. Following subsequent stirring at –78 °C for 30 minutes, a 1 M solution of $MgBr_2$ in MeOH (2.8 mL, 1.7 eq.) was added at this temperature. The reaction mixture was then warmed to rt and stirred at this temperature for 1 h. The reaction mixture was quenched with water (35 mL) and the aqueous layer extracted with Et_2O (3 × 35 mL). The combined organics were washed with brine, dried over $MgSO_4$ and concentrated *in vacuo*. The crude product was purified by flash column chromatography (pentane/Et_2O 100:1 to 20:1) to give the product (0.722 g, 1.5 mmol, 96:4 e.r.) as a colourless oil in 88% yield.

The e.r. of boronic ester **1i** could not be determined by chiral HPLC/SFC. Instead, the boronic ester was oxidised and the e.r. of alcohol **1i'** was determined.

R*f* = 0.6 (pentane/Et_2O 95:5).

$[\alpha]_D^{25}$**:** –19 (*c* = 2.1).

NMR Spectroscopy (*see spectra*):

^{1}H NMR (400 MHz, $CDCl_3$): δ_H 6.98 (d, *J* = 2.2 Hz, 1H, H_G), 6.92 (ddd, *J* = 8.2, 2.2, 0.8 Hz, 1H, H_D), 6.69 (d, *J* = 8.2 Hz, 1H, H_C), 5.93 (apparent q, *J* = 1.4 Hz, 1H, H_Q), 5.62 (apparent dd, *J* = 1.5, 0.7 Hz, 1H, $H_{Q'}$), 3.76 (s, 3H, H_A), 2.32–2.25 (m, 5H, $H_{F,\ M}$), 1.78 (ddd, *J* = 13.4, 12.3, 4.7 Hz, 1H, H_M), 1.61 (ddd, *J* = 13.4, 12.2, 4.5 Hz, 1H, $H_{M'}$), 1.56–1.43 (m, 2H, H_N), 1.26 (s, 3H, H_J), 1.24 (s, 6H, H_K), 1.23 (s, 6H, $H_{K'}$) ppm;

^{13}C NMR (101 MHz, $CDCl_3$): δ_C 154.5 (C_B), 135.5 (C_H), 129.6 (C_E), 127.4 (C_G), 126.6 (C_D), 124.9 (C_Q), 113.0 (C_P), 109.6 (C_C), 82.8 (C_L), 54.9 (C_A), 46.1 (C_O), 35.0 (C_M), 24.9 (C_K), 24.9 ($C_{K'}$), 24.3 (C_N), 21.0 (C_F *or* C_J), 21.0 (C_F *or* C_J) ppm;

^{11}B NMR (128 MHz, $CDCl_3$): δ_B 33.5 ppm.

IR (film): ν_{max} 2969, 2932, 1690, 1503, 1436, 1368, 1288, 1249, 1217, 1135, 1068, 1048 cm^{-1}.

HRMS (EI): m/z calc'd for $C_{21}H_{32}O_3{}^{11}BI$ $[M]^+$ 470.1584 found 470.1486.

(*R*)-6-Iodo-2-(2-methoxy-5-methylphenyl)hept-6-en-2-ol (1i')

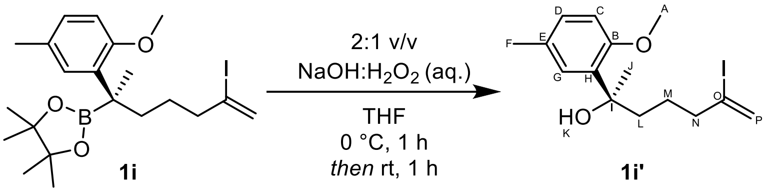

According to General Procedure **D**, vinyl iodide **1i** (24 mg, 0.05 mmol, 1.0 eq.) was reacted with a 2 M aqueous solution of NaOH (0.33 mL) and aqueous H_2O_2 (30% w/w, 0.17 mL) at 0 °C in THF (0.33 mL). The product (14 mg, 76%, 96:4 e.r.) was isolated as a colorless oil after purification by flash column chromatography (pentane/EtOAc 10:1).

R*f* = 0.2 (pentane/EtOAc 10:1).

$[\alpha]_D^{25}$: –3 (c = 2.1).

NMR Spectroscopy (*see spectra*):

^{1}H NMR (400 MHz, $CDCl_3$): δ_H 7.10–7.07 (m, 1H, H_G), 7.05–7.00 (m, 1H, H_D), 6.81 (d, J = 8.3 Hz, 1H, H_C), 5.98 (apparent q, J = 1.4 Hz, 1H, H_P), 5.67 (apparent dd, J = 1.4, 0.7 Hz, 1H, $H_{P'}$), 4.07 (s, 1H, H_K), 3.87 (s, 3H, H_A), 2.35 (t, J = 7.1, 2H, H_N), 2.30 (s, 3H, H_F), 1.92 (ddd, J = 13.5, 11.8, 4.8 Hz, 1H, H_L), 1.78 (ddd, J = 13.5, 11.5, 5.1 Hz, 1H, $H_{L'}$), 1.57 (s, 3H, H_J), 1.55–1.49 (m, 1H, H_M), 1.49–1.39 (m, 1H, $H_{M'}$) ppm;

^{13}C NMR (101 MHz, $CDCl_3$): δ_C 154.8 (C_B), 134.3 (C_H), 130.1 (C_E), 128.3 (C_D), 127.5 (C_G), 125.4 (C_P), 112.6 (C_O), 111.4 (C_C), 74.9 (C_I), 55.5 (C_A), 45.4 (C_N), 40.5 (C_L), 27.5 (C_J), 23.9 (C_M), 20.8 (C_F) ppm.

IR (film): ν_{max} 3438 (OH), 2933, 2863, 1619, 1499, 1458, 1364, 1236, 1142, 1033 cm^{-1}.

HRMS (EI): m/z calc'd for $C_{15}H_{21}IO_2$ $[M–H_2O]^+$ 342.0475 found 342.0473.

Chiral HPLC (IC column, 95:5 hexane/IPA, 0.7 mL/min, rt, 254 nm): 8.7 min (major), 9.6 min (minor), 96:4 e.r.

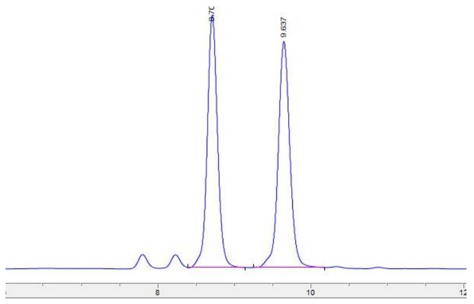

#	Time	Type	Area	Height	Width	Area%	Symmetry
1	8.702	VB	1643.2	176.5	0.1421	49.715	0.931
2	9.637	BB	1662	157.7	0.1645	50.285	0.927

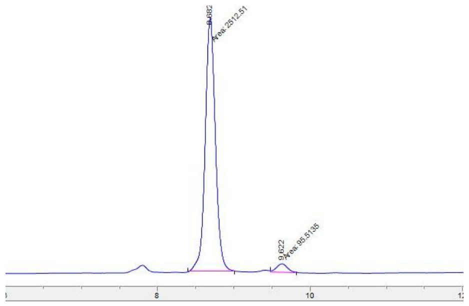

#	Time	Type	Area	Height	Width	Area%	Symmetry
1	8.682	MM	2512.5	267.9	0.1563	96.338	0.93
2	9.622	MM	95.5	9.3	0.1711	3.662	0.884

2-((1*S*,2*S*)-2-(2-Methoxy-5-methylphenyl)-1,2-dimethylcyclopentyl)-4,4,5,5-tetramethyl-1,3,2-dioxaborolane (3p)

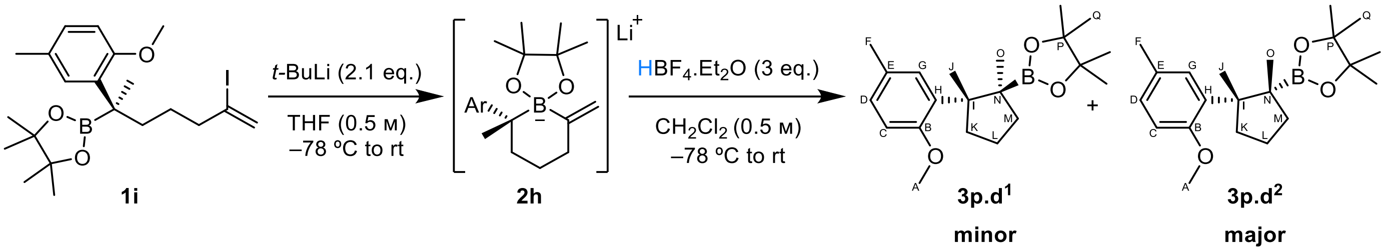

tert-Butyl lithium (1.7 M in pentane; 1.1 mL, 1.94 mmol, 2.1 eq.) was added dropwise to a solution of vinyl iodide **1i** (0.433 g, 0.92 mmol, 1.0 eq.) in anhydrous THF (1.8 mL, 0.5 M) at –78 °C (dry ice/acetone) and allowed to stir at this temperature for 1 h. The cooling bath was removed, and the reaction was stirred at rt for a further 1 h. After this time the solvent was removed *in vacuo* at rt and CH_2Cl_2 (1.8 mL, 0.5 M) was added. The reaction mixture was cooled again to –78 °C (dry ice/acetone) whereupon tetrafluoroboric acid diethyl ether complex (0.38 mL, 2.77 mmol, 3.0 eq.) was added. The reaction mixture was stirred at –78 °C for 2 h and slowly warmed to rt overnight (the replenishment of dry ice in the cooling bath was halted). The reaction mixture was diluted with Et_2O (5 mL) and quenched with sat. aq. $NaHCO_3$ (5 mL). The aqueous layer was extracted with Et_2O (3 × 5 mL), and the combined organics washed with brine, dried over $MgSO_4$ and concentrated *in vacuo*. The crude

residue was analysed by ^{1}H NMR to determine that the product was formed in 85:15 diastereomeric ratio. The crude product was then purified by flash column chromatography (pentane/CH_2Cl_2 4:1) to afford boronic ester **3p** (major d^2: 0.126 g, 0.37 mmol; minor d^1: 0.0212 g, 0.06 mmol) in 46% yield.

Major diastereomer (d^2):

R*f* = 0.3 (pentane/CH_2Cl_2 3:1).

$[\alpha]_D^{25}$**:** –29 (*c* = 2.1).

NMR Spectroscopy (*see spectra*):

^{1}H NMR (500 MHz, $CDCl_3$): δ_H 7.07 (d, *J* = 2.2 Hz, 1H, H_G), 6.93 (ddd, *J* = 8.2, 2.2, 0.8 Hz, 1H, H_D), 6.75 (d, *J* = 8.2 Hz, 1H, H_C), 3.78 (s, 3H, H_A), 2.32–2.24 (m, 5H, $H_{F, K, K'}$), 1.91–1.84 (m, 1H, H_M), 1.76–1.64 (m, 2H, $H_{L, L'}$), 1.61–1.51 (m, 1H, $H_{M'}$), 1.27 (s, 3H, H_O), 1.21 (s, 3H, H_J), 1.14 (s, 6H, H_Q), 1.02 (s, 6H, $H_{Q'}$) ppm;

^{13}C NMR (126 MHz, $CDCl_3$): δ_C 156.2 (C_B), 138.7 (C_H), 128.5 (C_E), 128.4 (C_G), 126.6 (C_D), 111.6 (C_C), 82.3 (C_P), 55.1 (C_A), 50.2 (C_I), 39.5 (C_K), 37.5 (C_M), 24.6 (C_Q), 24.5 ($C_{Q'}$), 21.6 (C_J), 21.0 (C_L), 20.8 (C_F), 20.5 (C_O) ppm;

^{11}B NMR (128 MHz, $CDCl_3$): δ_B 34.6 ppm.

IR (film): ν_{max} 2975, 2941, 2869, 2831, 1498, 1464, 1378, 1353, 1296, 1286, 1144, 1127 cm^{-1}.

HRMS (EI): m/z calc'd for $C_{21}H_{33}O_3{}^{11}B$ $[M]^+$ 344.2517 found 344.2514.

Minor diastereomer (d^1):

R*f* = 0.2 (pentane/CH_2Cl_2 3:1).

$[\alpha]_D^{25}$**:** –6 (*c* = 2.1).

NMR Spectroscopy (*see spectra*):

^{1}H NMR (500 MHz, $CDCl_3$): δ_H 7.10 (d, J = 2.2 Hz, 1H, H_G), 6.95 (ddd, *J* = 8.2, 2.2, 0.8 Hz, 1H, H_D), 6.76 (d, *J* = 8.2 Hz, 1H, H_C), 3.81 (s, 3H, H_A), 2.31–2.22 (m, 4H, $H_{F, K}$), 2.20–2.11 (m, 1H, H_M), 1.97–1.90 (m, 1H, $H_{K'}$), 1.82–1.73 (m, 2H, H_L), 1.45–1.37 (m, 1H, $H_{M'}$), 1.31 (s, 6H, H_Q), 1.30 (s, 3H, H_J), 1.27 (s, 6H, $H_{Q'}$), 0.71 (s, 3H, H_O) ppm;

^{13}C NMR (126 MHz, $CDCl_3$): δ_C 155.3 (C_B), 137.9 (C_H), 129.7 (C_G), 129.3 (C_E), 126.7 (C_D), 111.6 (C_C), 82.1 (C_P), 55.5 (C_A), 52.9 (C_I), 38.9 (C_K), 37.5 (C_M), 27.5 (C_J), 25.7 (C_Q), 24.5 ($C_{Q'}$), 22.3 (C_O), 21.2 (C_L), 20.8 (C_F) ppm;

^{11}B NMR (128 MHz, $CDCl_3$): δ_B 34.2 ppm.

IR (film): ν_{max} 2929, 2871, 1499, 1462, 1370, 1350, 1290, 1236, 1146 cm^{-1}.

HRMS (EI): m/z calc'd for $C_{21}H_{33}O_3{}^{11}B$ $[M]^+$ 344.2517 found 344.2511.

2-((1*S*,2*S*)-1,2-Dimethyl-2-vinylcyclopentyl)-1-methoxy-4-methylbenzene (11)

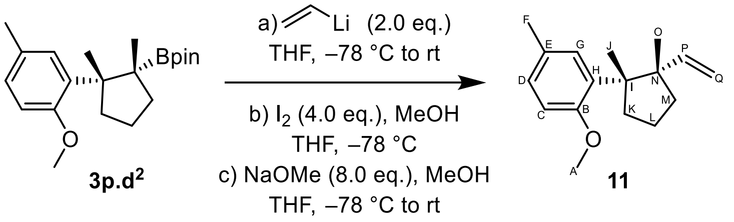

3p.d² **11**

According to a modified literature procedure,[18] boronic ester **3p.d²** (123 mg, 0.36 mmol, 1.0 eq.) was solved in THF (5.8 mL) and cooled to –78 °C (dry ice/acetone). To this solution, a solution of vinyl lithium (0.5 M in THF; 1.4 mL, 0.71 mmol, 2.0 eq.) was added dropwise. The reaction mixture was warmed to rt and stirred at this temperature for 30 minutes. It was then cooled to –78 °C and a solution of iodine (361 mg, 1.42 mmol, 4.0 eq.) in MeOH (1.4 mL) was added dropwise. The reaction mixture was stirred at –78 °C for 30 minutes whereupon NaOMe (0.5 M in MeOH; 5.7 mL, 2.85 mmol, 8.0 eq.) was added dropwise. The reaction mixture was warmed to rt and stirred at this temperature for an hour, after which time it was quenched with a saturated aqueous solution of $Na_2S_2O_3$ (5 mL). The aqueous layer was extracted Et_2O (3 × 5 mL), and the combined organic extracts were washed with brine, dried over $MgSO_4$ and concentrated *in vacuo*. The crude product was purified by flash column chromatography (pentane/CH_2Cl_2 50:1 to 33:1) to yield alkene **11** (67 mg, 0.27 mmol, 77%) as a colorless oil.

R_f = 0.2 (pentane/CH_2Cl_2 50:1).

$[\alpha]_D^{25}$**:** –34 (*c* = 2.1).

NMR Spectroscopy (*see spectra*):

¹H NMR (500 MHz, $CDCl_3$): δ_H 7.13 (d, *J* = 2.2 Hz, 1H, H_G), 7.00 (dd, *J* = 8.2, 2.2 Hz, 1H, H_D), 6.77 (d, *J* = 8.2 Hz, 1H, H_C), 5.73 (dd, *J* = 17.5 (*trans*), 10.8 (*cis*) Hz, 1H, H_P), 4.89 (dd, *J* = 17.5 (*trans*), 1.6 (*geminal*) Hz, 1H, H_{Qtrans}), 4.76 (dd, *J* = 10.8 (*cis*), 1.6 (*geminal*) Hz, 1H, H_{Qcis}), 3.76 (s, 3H, H_A), 2.50–2.40 (m, 1H, H_K), 2.31 (s, 3H, H_F), 1.98–1.90 (m, 1H, $H_{K'}$), 1.89–1.68 (m, 4H, $H_{L, L', M, M'}$), 1.42 (s, 3H, H_J), 1.33 (s, 3H, H_O) ppm;

¹³C NMR (126 MHz, $CDCl_3$): δ_C 156.6 (C_B), 147.6 (C_P), 135.5 (C_H), 129.7 (C_G), 128.6 (C_E), 127.1 (C_D), 111.5 (C_C), 108.8 (C_Q), 54.8 (C_A), 51.3 (C_I), 50.6 (C_N), 39.4 (C_K *or* C_M), 39.3 (C_K *or* C_M), 22.8 (C_J), 22.2 (C_O), 20.9 (C_F), 20.5 (C_L) ppm.

IR (film): ν_{max} 2952, 2875, 1638, 1498, 1465, 1243, 1176, 1063, 1034 cm^{-1}.

HRMS (EI): m/z calc'd for $C_{17}H_{24}O$ $[M]^+$ 244.1822 found 244.1821.

((1*R*,2*S*)-2-(2-Methoxy-5-methylphenyl)-1,2-dimethylcyclopentyl)methanol (12)

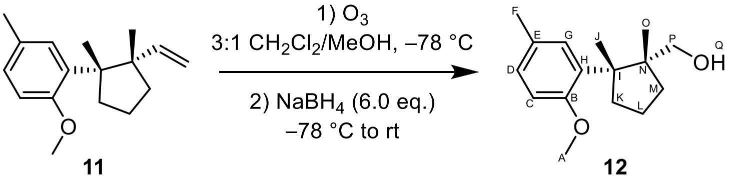

11 **12**

According to a modified literature procedure,[19] Sudan III indicator was added to alkene **11** (28 mg, 0.12 mmol, 1.0 eq.) solved in 3:1 CH_2Cl_2/MeOH (2.0 mL). The resultant pale red solution was cooled to –78 °C and ozone was bubbled (at half the maximum output level) through the solution at this temperature until the pale red colour disappeared. Dry nitrogen was then bubbled through the solution for 1 h at –78 °C, whereupon $NaBH_4$ (13 mg, 0.35 mmol, 3.0 eq.) was added in one portion and the reaction monitored by TLC. The solution was stirred at –78 °C for 1.5 h, at which time a further portion of $NaBH_4$ (13 mg, 0.35 mmol, 3.0 eq.) was added. After a further 0.5 h at –78 °C, the reaction mixture was warmed to 0 °C for 1 h and then to rt for 0.5 h. After this time, the reaction was diluted with EtOAc (5 mL) and quenched with water (5 mL). The aqueous layer was extracted EtOAc (3 × 5 mL), and the combined organic extracts were washed with brine, dried over $MgSO_4$ and concentrated in vacuo. The crude product was purified by flash column chromatography (pentane/EtOAc 10:1) to yield alcohol **12** (12 mg, 0.048 mmol, 41%, 96:4 e.r.) as a colorless oil.

R*f* = 0.1 (pentane/EtOAc 95:5).

$[\boldsymbol{\alpha}]_D^{25}$**:** –12 (*c* = 1.8).

NMR Spectroscopy (*see spectra*):

^{1}H NMR (400 MHz, $CDCl_3$): δ_H 7.09 (d, *J* = 2.2 Hz, 1H, H_G), 7.01 (ddd, *J* = 8.2, 2.2, 0.7 Hz, 1H, H_D), 6.80 (d, *J* = 8.2 Hz, 1H, H_C), 3.80 (s, 3H, H_A), 3.12 (s, 2H, H_P), 2.50–2.39 (m, 1H, H_K), 2.28 (s, 3H, H_F), 1.95–1.56 (m, 5H, $H_{K', L, L', M}$), 1.41 (s, 3H, H_J), 1.40–1.33 (m, 1H, $H_{M'}$), 1.20 (s, 3H, H_O) ppm;

^{13}C NMR (101 MHz, $CDCl_3$): δ_C 156.3 (C_B), 135.3 (C_H), 129.9 (C_E), 129.4 (C_G), 127.8 (C_D), 112.1 (C_C), 70.6 (C_P), 55.3 (C_A), 50.4 (C_I), 49.0 (C_N), 42.0 (C_K), 37.2 (C_M), 24.1 (C_J), 21.3 (C_L), 21.0 (C_F *or* C_O), 20.9 (C_F *or* C_O) ppm.

IR (film): ν_{max} 3439 (br, OH), 2945, 2877, 1498, 1465, 1245, 1175, 1068, 1030 cm^{-1}.

HRMS (ESI): m/z calc'd for $C_{16}H_{24}O_2$ $[M+H]^+$ 249.1849 found 249.1847.

Chiral HPLC (IC column, 97:3 hexane/IPA, 0.7 mL/min, rt, 254 nm): 11.4 min (major), 12.0 min (minor), 96:4 e.r.

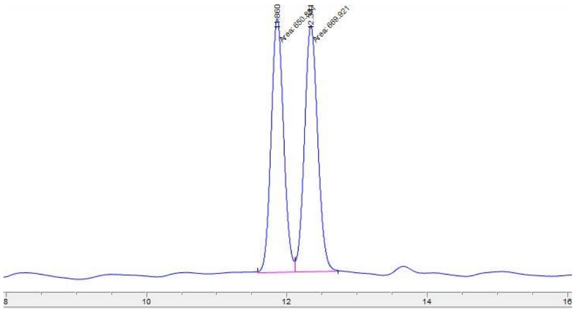

#	Time	Type	Area	Height	Width	Area%	Symmetry
1	11.86	MF	650.9	52.7	0.2058	49.279	0
2	12.341	FM	669.9	51.1	0.2183	50.721	0.909

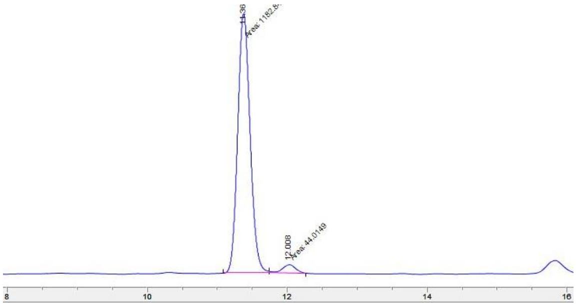

#	Time	Type	Area	Height	Width	Area%	Symmetry
1	11.36	MF	1182.8	96.2	0.2048	96.412	0.87
2	12.008	FM	44	3.2	0.2292	3.588	1.059

(+)-Herbertene-1,14-diol (13)

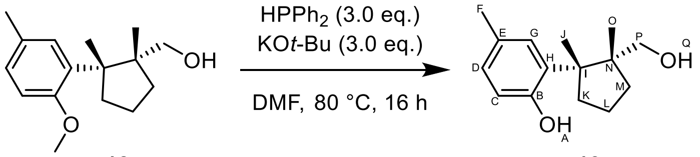

12 **13**

According to a modified literature procedure,[20] *t*-BuOK (16 mg, 0.143 mmol, 3.0 eq.) was solved in DMF (50 µL) at rt and $HPPh_2$ (25 µL, 0.143 mmol, 3.0 eq.) was added dropwise. Upon addition of $HPPh_2$ the colourless solution turned bright red. The mixture was stirred at rt for 10 minutes, after which time methyl ether **12** (12 mg, 0.048 mmol, 1.0 eq.) was added as a solution in DMF (50 µL) and heated to 80 ºC for 16 hours. The reaction mixture was then diluted with CH_2Cl_2 (1 mL) and brine (5 mL) was added. By adding aqueous 2 M HCl, the aqueous layer was adjusted to pH 1–2 and then extracted with CH_2Cl_2 (3 x 5 mL) and the combined organic extracts were washed with brine, dried over $MgSO_4$ and concentrated in vacuo. The crude product was purified by flash column chromatography (pentane/EtOAc 9:1 to 4:1) to yield **13** (6.5 mg, 0.028 mmol, 58%, 96:4 e.r.) as a white solid.

R*f* = 0.1 (pentane/EtOAc 9:1).

$[\alpha]_D^{25}$: +9.6 (*c* = 1.00, $CHCl_3$). Literature values: $[\alpha]_D^{21}$: +33.5 (*c* = 0.19, $CHCl_3$);[21] $[\alpha]_D^{27}$: +15.5 (*c* = 1.14, $CHCl_3$);[22] $[\alpha]_D^{25}$: +11.8 (*c* = 1.00, $CHCl_3$).[23]

NMR Spectroscopy (*see spectra*):

^{1}H NMR (500 MHz, $CDCl_3$): δ_H 6.96 (d, *J* = 2.1 Hz, 1H, H_G), 6.91 (ddd, *J* = 8.0, 2.1, 0.8 Hz, 1H, H_D), 6.73 (d, *J* = 8.0 Hz, 1H, H_C), 3.36 (d, *J* = 11.1 Hz, 1H, H_P), 3.28 (d, *J* = 11.0 Hz, 1H, $H_{P'}$), 2.52–2.38 (m, 1H, H_K), 2.27 (s, 3H, H_F), 1.98–1.77 (m, 3H, $H_{K', L, L'}$), 1.56 (s, 3H, H_J), 1.50–1.41 (m, 1H, H_M), 1.32–1.25 (m, 1H, $H_{M'}$), 1.23 (s, 3H, H_O) ppm; data matched that from the literature;[23]

^{13}C NMR (125 MHz, $CDCl_3$): δ_C 153.2 (C_B), 132.9 (C_H), 129.8 (C_G), 129.3 (C_E), 128.1 (C_D), 118.2 (C_C), 70.8 (C_P), 50.9 (C_I), 48.9 (C_N), 42.4 (C_K), 36.0 (C_M), 24.0 (C_J), 21.2 (C_L), 21.0 (C_F), 20.4 (C_O) ppm; data matched that from the literature.[23]

IR (film): ν_{max} 3205 (br, OH), 2956, 2927, 2878, 1463, 1411, 1276, 1260, 1023 cm^{-1}.

HRMS (Negative ion nanospray): m/z calc'd for $C_{15}H_{21}O_2$ $[M–H]^-$ 233.1542 found 233.1546.

2.4.1 Optimisation of Conditions for the Ring Contractive 1,2-Metallate Rearrangement

Full formation of boronate complex **2h** was achieved using our standard conditions and confirmed by ^{11}B NMR, with a characteristic peak observed at 6.6 ppm. A screen of electrophile addition conditions was then conducted, varying the reaction solvent, and the amount and identity of the proton source (Table S2).

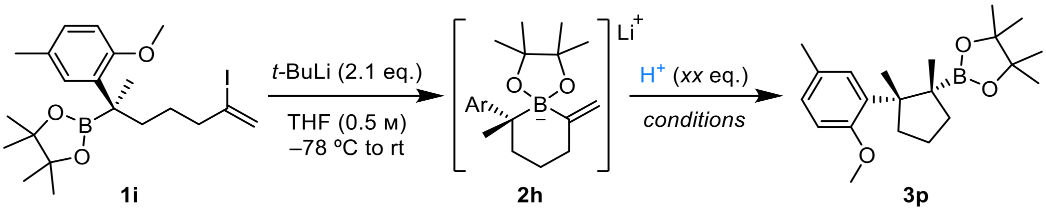

entry	proton source	conditions	^1H NMR yield (%)[a]	d.r. [b]
1	$HBF_4 \cdot Et_2O$ (1.5 eq.)	THF (0.5 м), –78 °C 2h, then rt[c]	trace	-
2	$HBF_4 \cdot Et_2O$ (1.5 eq.)	1:1 THF/MeCN (0.25 м), –40 °C 2h, then rt[c]	7	3:1
3	$HBF_4 \cdot Et_2O$ (1.5 eq.)	MeCN (0.5 м), –40 °C 2h, then rt[c]	14	4:1
4	$HBF_4 \cdot Et_2O$ (1.5 eq.)	Et_2O (0.5 м), –78 °C 2h, then rt[c]	9	6:1
5	$HBF_4 \cdot Et_2O$ (1.5 eq.)	$CHCl_3$ (0.5 м), –60 °C 2h, then rt[c]	8	7:1
6	$HBF_4 \cdot Et_2O$ (1.5 eq.)	CH_2Cl_2 (0.5 м), –78 °C 2h, then rt[c]	29	5:1
**7**	**$HBF_4 \cdot Et_2O$ (3 eq.)**	**CH_2Cl_2 (0.5 м), –78 °C 2h, then rt[c]**	**38**	**6:1**
8	$HBF_4 \cdot Et_2O$ (5 eq.)	CH_2Cl_2 (0.5 м), –78 °C 2h, then rt[c]	21	4:1
9	$HBF_4 \cdot Et_2O$ (2.5 eq.)	CH_2Cl_2 (0.5 м), –78 °C 2h, then rt[c]	33	6:1
10	HCl in Et_2O (3 eq.)	CH_2Cl_2 (0.5 м), –78 °C 2h, then rt[c]	24	4:1

Table S2: Total synthesis of (+)-herbertene-1,14-diol – Electrophile addition conditions screening. All reactions conducted on a 0.1 mmol scale. [a] Yield determined by ^1H NMR analysis using CH_2Br_2 as an internal standard. Isolated yields are given in parentheses. [b] Ratio determined by ^1H NMR analysis. [c] The reaction mixture was allowed to slowly warm to rt overnight in the cooling bath (either the cryostat cooling device was turned off or the replenishment of dry ice was halted).

Key observations:

- THF is a poor solvent for the electrophile addition step, DCM is superior since it yields the product in higher amounts and allows a much cleaner transformation of the boronate complex.
- Increasing the number of equivalents of proton source increases the yield, up until beyond 3 equivalents at which point adding more is detrimental to yield and d.r.
- HBF_4 diethyl ether complex is the best source of a proton for this transformation.

It was also found that the reaction yield (NMR yield) increased with increasing scale:

0.1 mmol – 38%; 0.3 mmol – 43%; 0.9 mmol – 47% (46% isolated).

2.4.2 An Alternative Boronic Ester Functionalisation: Homologation

A range of Matteson homologation protocols were attempted to introduce the carbinol moiety present in **12**, all proving unfruitful and returning only starting material (Table S3), likely due to the hindered nature of boronic ester **3p** resulting in decomposition of the carbenoid outcompeting the formation of boronate complex **18**.

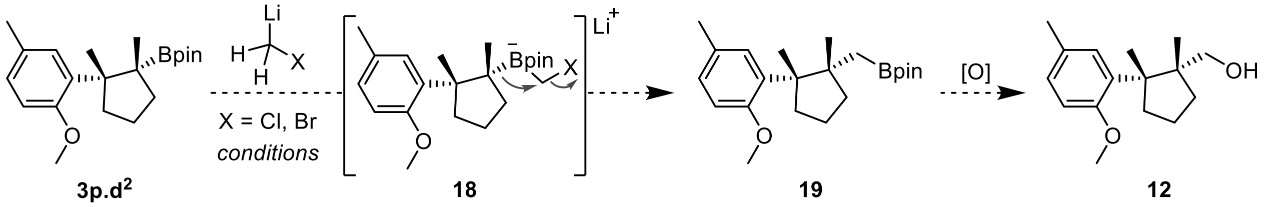

entry	conditions	result
1	CH_2BrCl (2.5 eq.), *n*-BuLi (2.5 eq.)<br>THF, –78 °C 10 mins, then rt o/n	85% RSM[a]
2	CH_2Br_2 (2.5 eq.), *n*-BuLi (2.2 eq.)<br>THF, –78 °C 10 mins, then rt 2 h[18]	90% RSM[a]
3	CH_2BrCl (5 eq.), *n*-BuLi (5 eq.)<br>THF, –78 °C 3 h, then rt o/n	85% RSM[a]

Table S3: Total synthesis of (+)-herbertene-1,14-diol – Boronic ester homologation attempts.
All reactions conducted on a 0.1 mmol scale. [a] Yield determined by ^1H NMR analysis using CH_2Br_2 as an internal standard.

2.4.3 Screening of Conditions for the Demethylation of the Aryl Methyl Ether

A range of demethylation conditions were trialed (Table S4). Standard demethylation conditions were not suitable for this substrate, with NaSEt unable to enact the desired demethylation (entry 1) and BBr_3 causing complete decomposition (entry 2). Pleasingly, the combination of $HPPh_2$ and KO*t*-Bu enabled the demethylation of **12** (entry 3), yielding the desired phenol in 58% isolated yield.

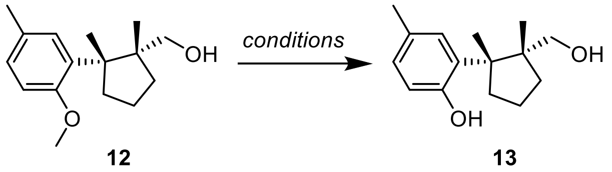

entry	conditions	result
1	NaSEt (20 eq.), DMF, 150 °C, 24 h	56% RSM[a]
2	BBr_3 (1.1 eq.), CH_2Cl_2, 0 °C to rt, 1 h	complex mixture, no RSM
3	$HPPh_2$ (3.0 eq.), KO*t*-Bu (3.0 eq.), 80 °C, 16 h	58% desired product[b]

Table S4: Total synthesis of (+)-herbertene-1,14-diol – Demethylation attempts.
[a] Yield determined by ^1H NMR analysis using CH_2Br_2 as an internal standard. [b] Isolated yield.

3. DETERMINATION OF RELATIVE STEREOCHEMISTRY

3.1. Ring Current Effects for the Determination of Relative Stereochemistry

3.1.1 Ring currents and their influences on the NMR chemical shifts of adjacent nuclei

An aromatic ring current is an electronic phenomenon observed in aromatic species. If an external magnetic field, B_0, is applied perpendicular to the plane of the aromatic system, a ring current is induced in the π-system (Figure S1). This ring current creates its own magnetic field, B_{ind}. For nuclei in the plane of the aromatic ring, for example protons on a phenyl ring, the external and induced magnetic fields are additive, giving a higher local magnetic field. Thus, a higher frequency or lower external magnetic field is required to achieve resonance. However, for nuclei above or below the plane of the aromatic ring the reverse is true since the local magnetic field is lower. The shielding influence of aromatic groups on adjacent groups is a phenomenon observed in a variety of contexts, such as in the use of chiral derivatising agents and chiral solvating agents.[24,25]

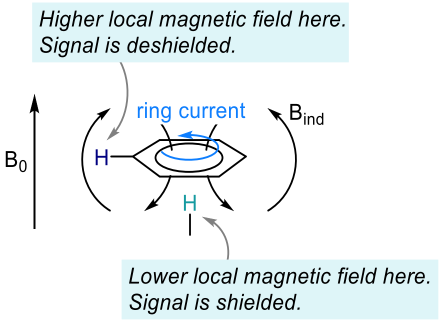

Figure S1: The ring current in an aromatic ring and its influence on adjacent nuclei.

Ring current effects on adjacent protons are often very pronounced in highly substituted 5-membered rings, where a phenyl substituent is forced to take a face-on conformation with vicinal substituents due to adjacent steric bulk.[26] In **20.d²**, where the phenyl group is face on to the adjacent methyl group, the chemical shift of the methyl group is reduced compared to in **20.d¹**, where it experiences no ring current influence (Figure S2).

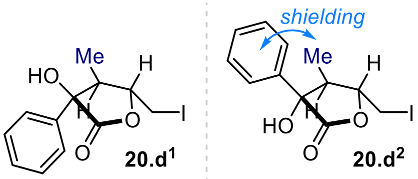

20.d¹ δ_{CH_3}= 1.1 ppm

20.d² δ_{CH_3}= 0.7 ppm

Figure S2: The influence of ring currents in a 5-membered lactone.

Silyl diether compounds of type **21** adopt a predominant conformation with a stabilising hydrogen bond, giving a 5-membered chelate-like structure (Figure S3).[27] Whichever group is adjacent to the shielding phenyl group displays a lower chemical shift compared to when it is distal to the phenyl group.

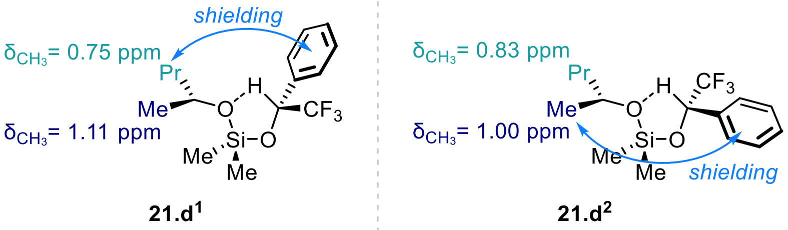

Figure S3: The influence of ring currents in a silyl diether compounds with a 5-membered chelate structure.

3.1.2 Ring currents in cyclopentyl boronic esters bearing aryl substituents

Such shielding influences are a characteristic feature of the ^{1}H NMR spectra of herbertane and cuparane sesquiterpene natural products, such as in herbertene-1,14-diol (**13**) and herbertene-1,15-diol (**22**) (Figure S4).[21]

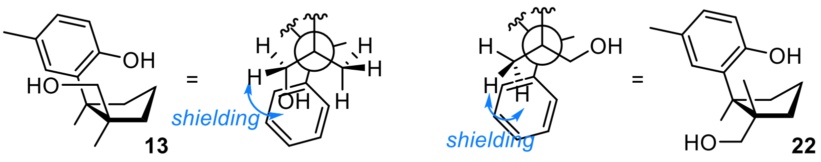

	δ (ppm)	
CH_2OH	3.28, 3.37	3.50, 4.02
CH_3	1.23	0.82

Figure S4: Comparison between the chemical shifts of the methylene and methyl protons in herbertene-1,14-diol and herbertene-1,15-diol.

The chemical shifts of the alcohol and methyl protons on the homobenzylic quaternary carbon in cyclopentyl alcohol **3o'** have been reported to vary significantly between each diastereomer and such variation has been attributed to the influence of the ring currents of the phenyl group.[28] We observed the same phenomenon in both cyclopentyl alcohol **3o'** and cyclopentyl boronic ester **3o** (Figure S5); whichever functionality was in close proximity (*cis* relationship) to the phenyl group had a lower chemical shift than when it was further away (*trans* relationship).

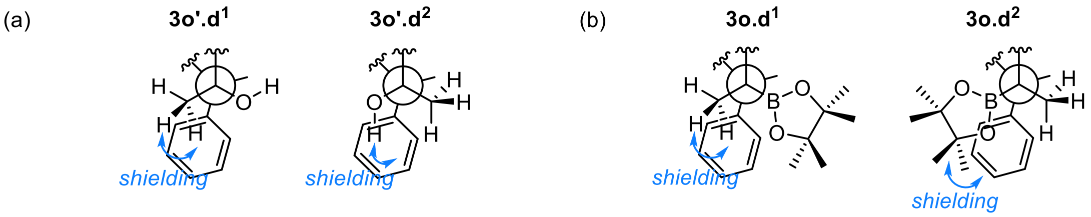

(a)	3o'.d1 δ (ppm)	3o'.d2 δ (ppm)
alcohol	1.48	0.90
methyl	0.94	1.26

(b)	3o.d1 δ (ppm)	3o.d2 δ (ppm)
pinacol	1.30, 1.29	0.97, 0.93
methyl	0.64	1.19

Figure S5: (a) Comparison between the chemical shifts of the alcohol and methyl protons in the diastereomers of 3o'; (b) Comparison between the chemical shifts of the pinacol and methyl protons in the diastereomers of 3o.

The chemical shifts of the pinacol methyl protons and methylene protons adjacent to the electrophile of all the product cyclopentyl boronic esters in the electrophile scope (Figure S6) and boronic ester scope (Figure S7) are presented. Following these patterns, it is easy to assign the relative configuration of the major diastereomer formed with each electrophile. The clearest trend is observed in the pinacol methyl proton shifts (*note:* the *gem*-dimethyl groups are diastereotopic and thus give two singlets or, in some cases, two coincident singlets which are seen as one peak). The trend is still evident when looking at the diastereotopic methylene protons, however the identity of the electrophile can result in large variations in chemical shift between compounds. We also encountered the same phenomenon during our total synthesis of (+)-herbertene-1,14-diol, allowing the identification of the major/minor diastereomer (Figure S8).

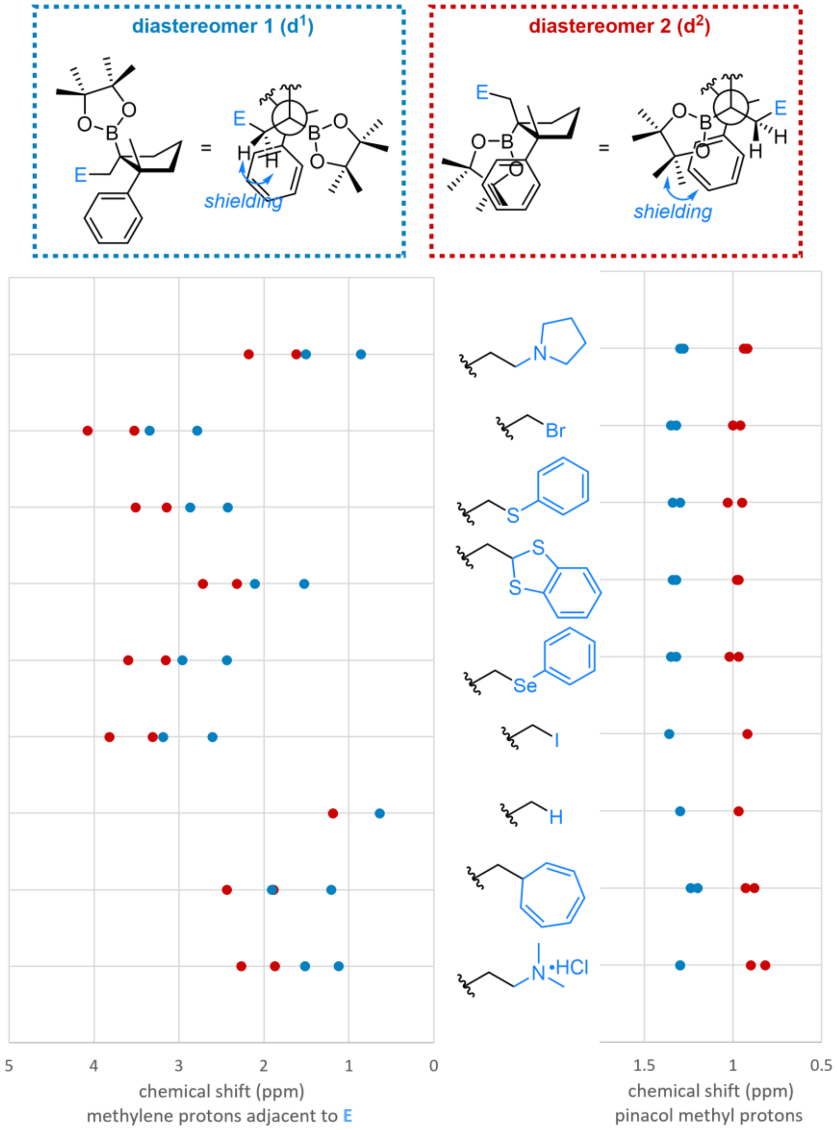

Figure S6: Electrophile scope – Comparison between the chemical shifts of the pinacol and methylene protons in cyclopentyl boronic esters.

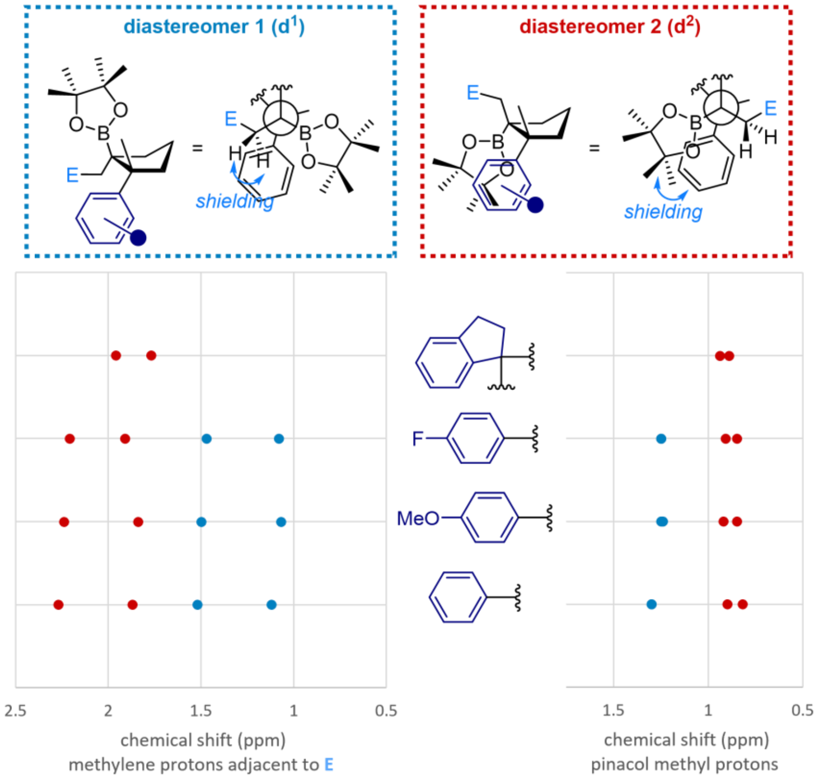

Figure S7: Substrate scope – Comparison between the chemical shifts of the pinacol and methylene protons in cyclopentyl boronic esters.

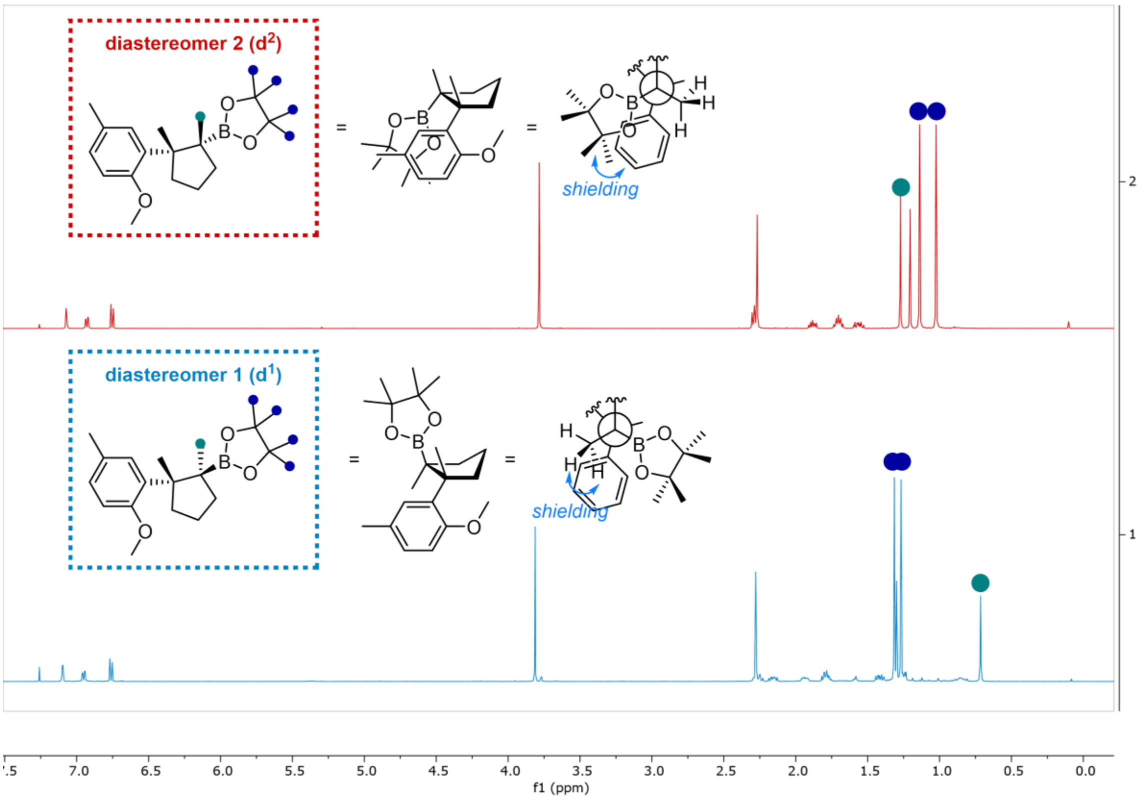

Figure S8: Total synthesis of (+)-herbertene-1,14-diol – Comparison between the chemical shifts of the pinacol and methyl protons in intermediate boronic ester 3p.

3.2. One Dimensional nOe NMR Experiments for the Determination of Relative Stereochemistry

3.2.1. 2-((1*S*,2*R*)-2-Isopropyl-1-(4,4,5,5-tetramethyl-1,3,2-dioxaborolan-2-yl)cyclopentyl)-*N*,*N*-dimethylethan-1-amine hydrochloride salt (3f)

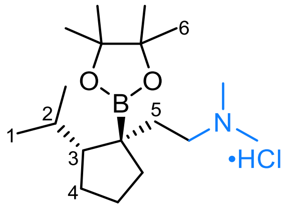

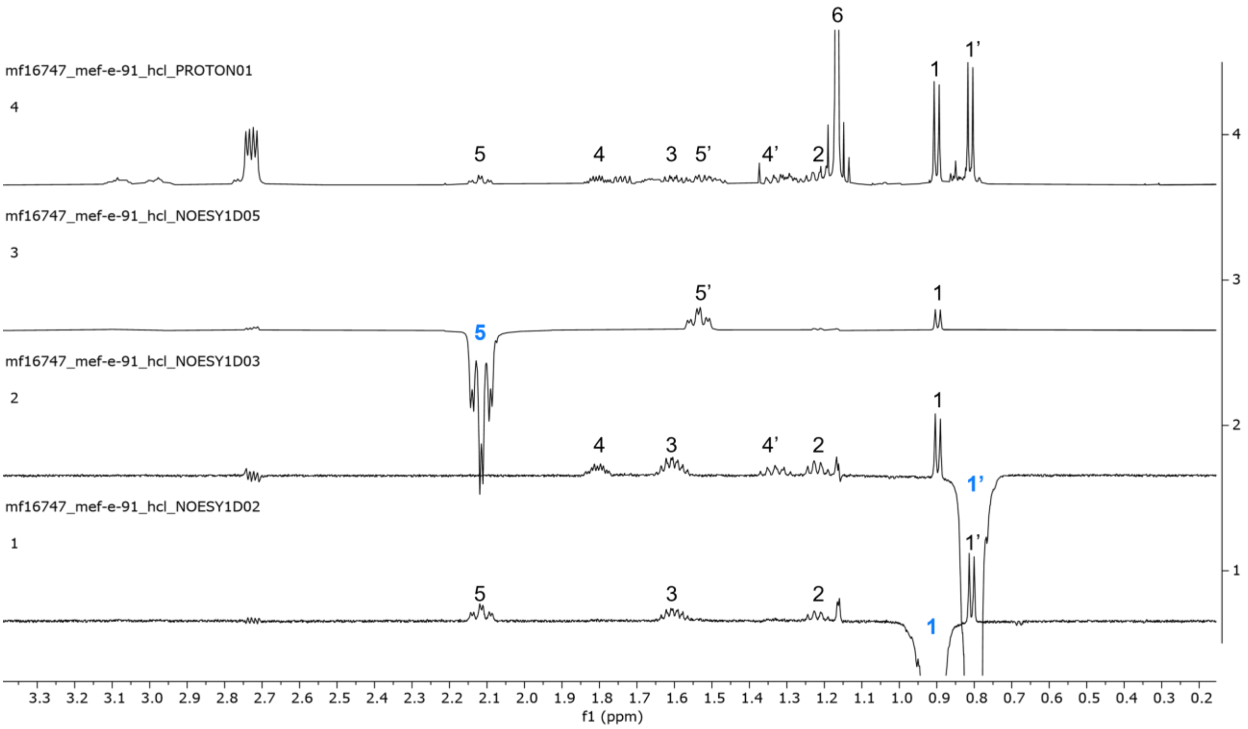

Key observations:

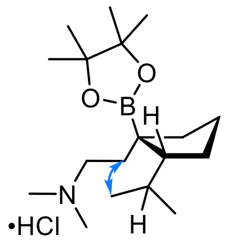

- Irradiation of C**H**$^5{}_2$: Observable nOe correlation with H^1 protons but no significant correlation with H^3.
- Irradiation of C**H**$^1{}_3$: Observable nOe correlation with H^5 but no significant correlation with H^6. nb. H$^{1'}$ no correlation with H^5 but correlation with H^4

3.2.2. *N*,*N*-Dimethyl-2-((1*S*,2*S*)-2-methyl-1-(4,4,5,5-tetramethyl-1,3,2-dioxaborolan-2-yl)cyclopentyl)ethan-1-amine hydrochloride salt (3e)

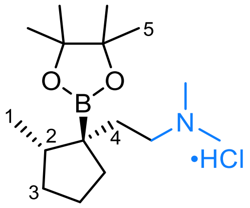

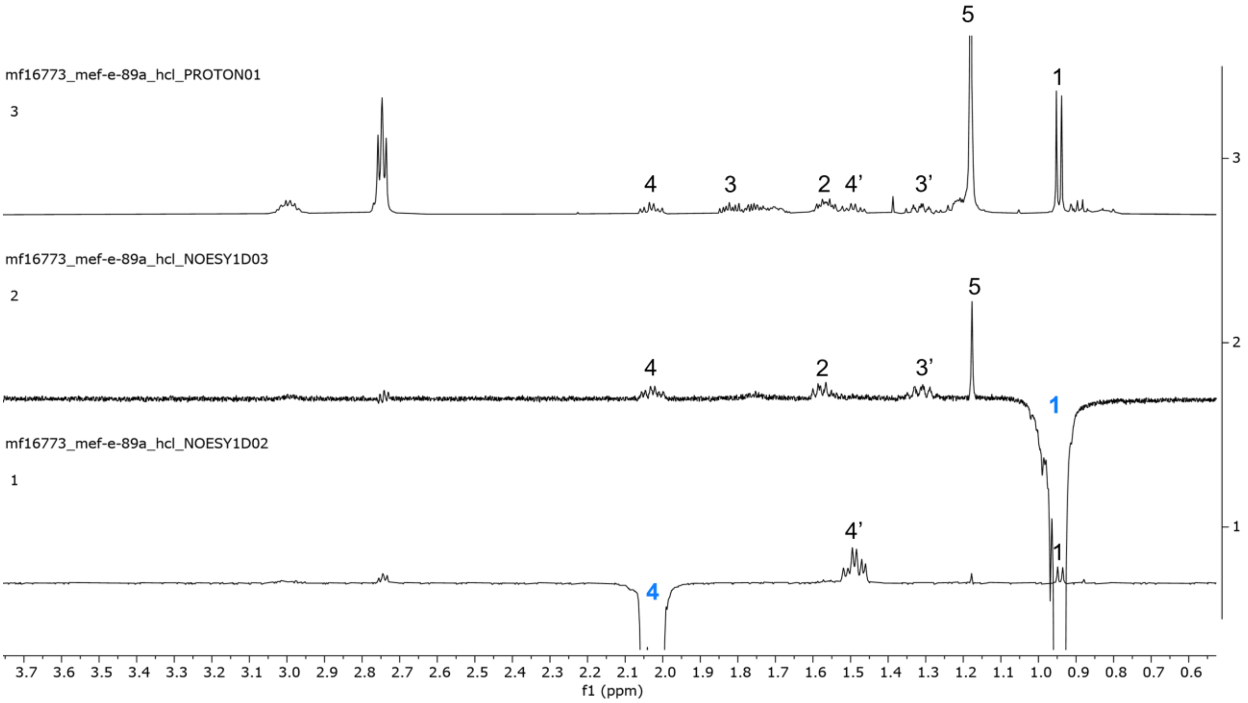

Key observations:

- Irradiation of C**H**[1]$_3$: Observable nOe correlation with H[4] proton and slight correlation with H[5].
- Irradiation of C**H**[4]$_2$: Observable nOe correlation with H[1] but no significant correlation with H[2].

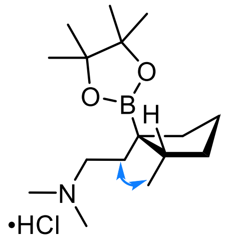

4. PROPOSED MODEL FOR DIASTEREOSELECTIVITY

4.1. α-Tertiary Stereocentres

In the addition of an electrophile to boronate complexes bearing an α-tertiary stereocentre, we propose that the diastereoselectivity observed is dictated by the preferred conformation of the boronate complex (Scheme S4). A destabilising gauche interaction between the pinacol and the alkyl substituent disfavours reacting conformation **II**, and so in the preferred reacting conformation (**I**) the large R group is placed axial to limit this destabilising interaction. Under both conditions A and B, we generate the same major diastereomer.

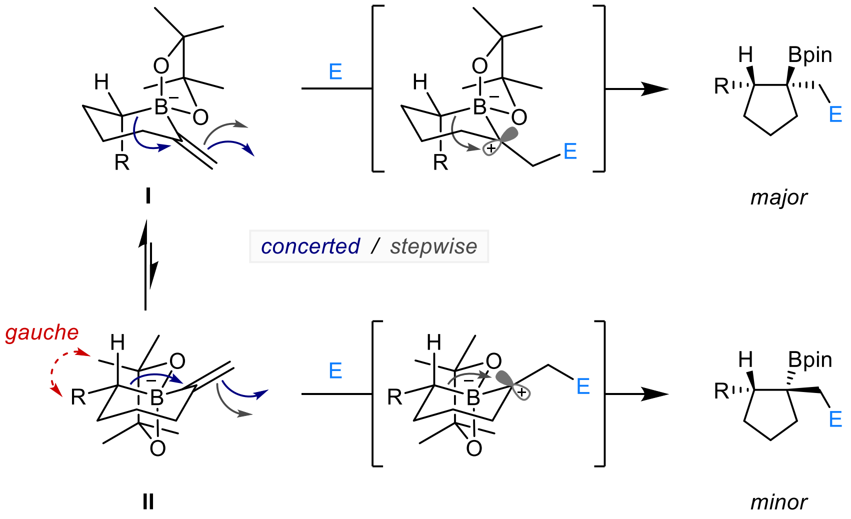

Scheme S4: Proposed stereochemical model for the addition of electrophiles to boronate complexes bearing an α-tertiary stereocentre.

4.2. α-Quaternary Stereocentres

In the addition of an electrophile to boronate complexes bearing an α-quaternary stereocentre, we propose that the difference in energy between conformations **III** and **IV** is very small (Scheme S5). Upon addition of the co-solvent, under conditions B, the switch in selectivity could result from changes in both the steric environment around the boronate complex and the electronics of the boronate complex compared to the system without the co-solvent (conditions A).

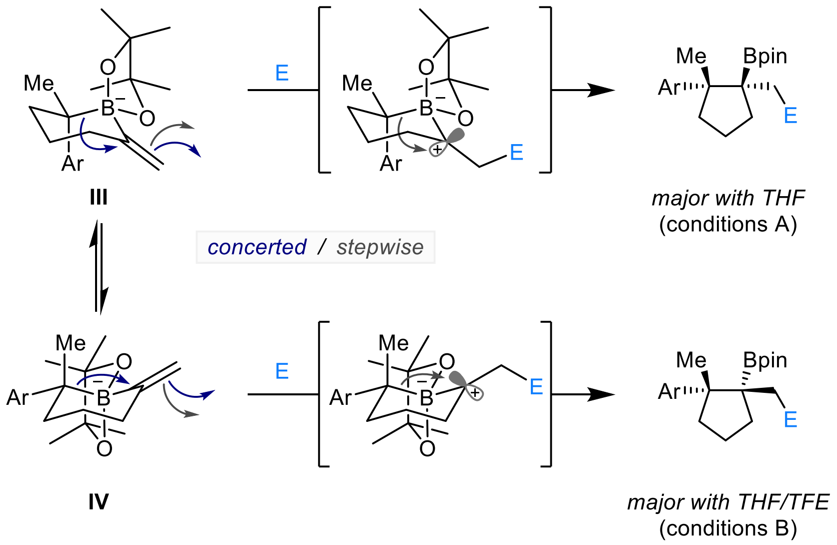

Scheme S5: Proposed stereochemical model for the addition of electrophiles to boronate complexes bearing an α-quaternary stereocentre.

We considered conducting a computational study to elucidate of the origins of this diastereodivergency. However, in our previously reported computational study of cyclic alkenyl boronate complexes, this proved highly challenging.[2] In one case, we calculated the energy difference between the reactive conformers in the stereodetermining step to be small, yet experimentally the diastereoselection was observed to be high. This indicated that other effects, not able to be accounted for in the calculations, may play a role in the preference for one conformer over another. These could include fluxional effects in the lithium coordination sphere, such as interactions between the lithium counterion and solvent molecules, the pinacol oxygen atoms, or aryl substituents. Therefore, we anticipate that it would be very difficult to draw conclusions from computational studies looking at the solvent-dependent diastereoselection of our system.

5. REFERENCES

[1] A. F. Burchat, J. M. Chong, N. Nielsen, *J. Organomet. Chem.* **1997**, *542*, 281–283.

[2] R. Davenport, M. Silvi, A. Noble, Z. Hosni, N. Fey, V. K. Aggarwal, *Angew. Chem. Int. Ed.* **2020**, *59*, 6525–6528; *Angew. Chem.* **2020**, *132*, 6587–6590.

[3] R. J. Armstrong, C. Sandford, C. García-Ruiz, V. K. Aggarwal, *Chem. Commun.* **2017**, *53*, 4922–4925.

[4] S. H. Bennett, A. Fawcett, E. H. Denton, T. Biberger, V. Fasano, N. Winter, V. K. Aggarwal, *J. Am. Chem. Soc*. **2020**, *142*, 16766–16775.

[5] V. Bagutski, R. M. French, V. K. Aggarwal, *Angew. Chem. Int. Ed.* **2010**, *49*, 5142–5145; *Angew. Chem.* **2010**, *122*, 5268–5271.

[6] J. L. Stymiest, V. Bagutski, R. M. French, V. K. Aggarwal, *Nature* **2008**, *456*, 778–783.

[7] G. Dutheuil, M. P. Webster, P. A. Worthington, V. K. Aggarwal, *Angew. Chem. Int. Ed.* **2009**, *48*, 6317–6319; *Angew. Chem.* **2009**, *121*, 6435 –6437.

[8] D. J. Blair, C. J. Fletcher, K. M. P. Wheelhouse, V. K. Aggarwal, *Angew. Chem. Int. Ed.* **2014**, *53*, 5552–5555; *Angew. Chem.* **2014**, *126*, 5658–5661.

[9] J. L. Stymiest, G. Dutheuil, A. Mahmood, V. K. Aggarwal, *Angew. Chem. Int. Ed.* **2007**, *46*, 7491–7494; *Angew. Chem.* **2007**, *119*, 7635 –7638.

[10] M. Silvi, R. Schrof, A. Noble, V. K. Aggarwal, *Chem. Eur. J.* **2018**, *24*, 4279–4282.

[11] J. M. Fabre, J. Garín, S. Uriel, *Tetrahedron* **1992**, *48*, 3983–3990.

[12] R. Larouche-Gauthier, T. G. Elford, V. K. Aggarwal, *J. Am. Chem. Soc.* **2011**, *133*, 16794–16797.

[13] A. G. M. Barrett, D. Dhanak, G. G. Graboski, S. J. Taylor, *Org. Synth.* **1990**, *68*, 8.

[14] P. T. Nyffeler, S. G. Durón, M. D. Burkart, S. P. Vincent, C. H. Wong, *Angew. Chem. Int. Ed.* **2005**, *44*, 192–212; *Angew. Chem.* **2005**, *117*, 196–217.

[15] E. Rivera-Chao, M. Fañanás-Mastral, *Angew. Chem. Int. Ed.* **2021**, *60*, 16922–16927; *Angew. Chem.* **2021**, *133*, 17059–17064.

[16] R. E. Banks, M. K. Besheesh, S. N. Mohialdin-Khaffaf, I. Sharif, *J. Chem. Soc., Perkin Trans. 1* **1996**, 2069–2076.

[17] R. Hayashi, A. Shimizu, J. I. Yoshida, *J. Am. Chem. Soc.* **2016**, *138*, 8400–8403.

[18] R. P. Sonawane, V. Jheengut, C. Rabalakos, R. Larouche-Gauthier, H. K. Scott, V. K. Aggarwal, *Angew. Chem. Int. Ed.* **2011**, *50*, 3760–3763; *Angew. Chem.* **2011**, *123*, 3844–3847.

[19] C. S. Swindell, P. P. Bomi, *J. Org. Chem.* **1990**, *55*, 3–5.

[20] W. Pan, C. Li, H. Zhu, F. Li, T. Li, W. Zhao, *Org. Biomol. Chem.* **2021**, *19*, 7633–7640

[21] H. Irita, T. Hashimoto, Y. Fukuyama, Y. Asakawa, *Phytochemistry* **2000**, *55*, 247–253.

[22] Y. Fukuyama, K. Matsumoto, Y. Tonoi, R. Yokoyama, H. Takahashi, H. Minami, H. Okazaki, Y. Mitsumoto, *Tetrahedron* **2001**, *57*, 7127–7135.

[23] S. Acherar, G. Audran, F. Fotiadu, H. Monti, *Eur. J. Org. Chem.* **2004**, 5092–5099.

[24] T. J. Wenzel, J. D. Wilcox, *Chirality* **2003**, *15*, 256–270.

[25] J. A. Dale, H. S. Mosher, *J. Am. Chem. Soc.* **1973**, *95*, 512–519.

[26] P. A. Bartlett, D. J. Tanzella, J. F. Barstow, *J. Org. Chem.* **1982**, *47*, 3941–3945.

[27] R. T. Williamson, A. C. B. Sosa, A. Mitra, P. J. Seaton, D. B. Weibel, F. C. Schroeder, J. Meinwald, F. E. Koehn, *Org. Lett.* **2003**, *5*, 1745–1748.

[28] T. D. Hoffman, D. J. Cram, *J. Am. Chem. Soc.* **1969**, *91*, 1000–1008.

6. SPECTROSCOPIC DATA

^{1}H NMR (400 MHz, $CDCl_3$) of **1a** *(<u>see procedure</u>)*

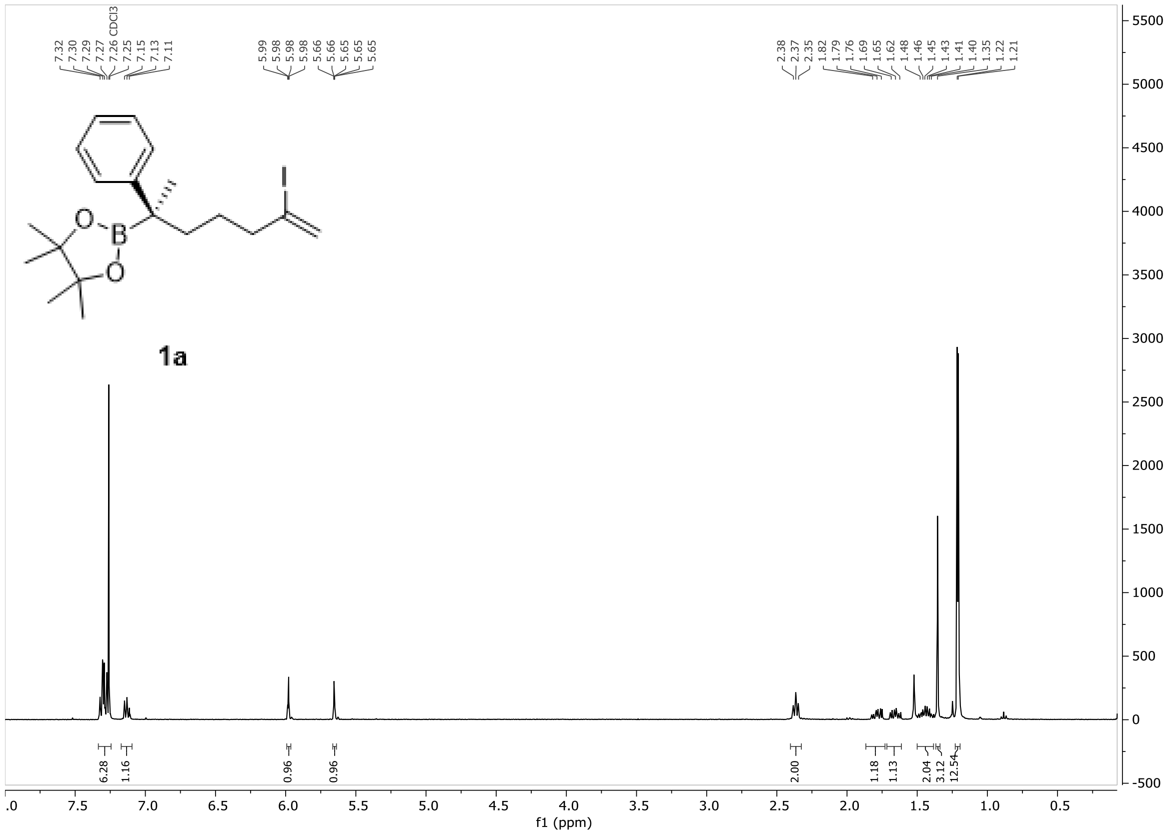

^{13}C NMR (101 MHz, $CDCl_3$) of **1a**

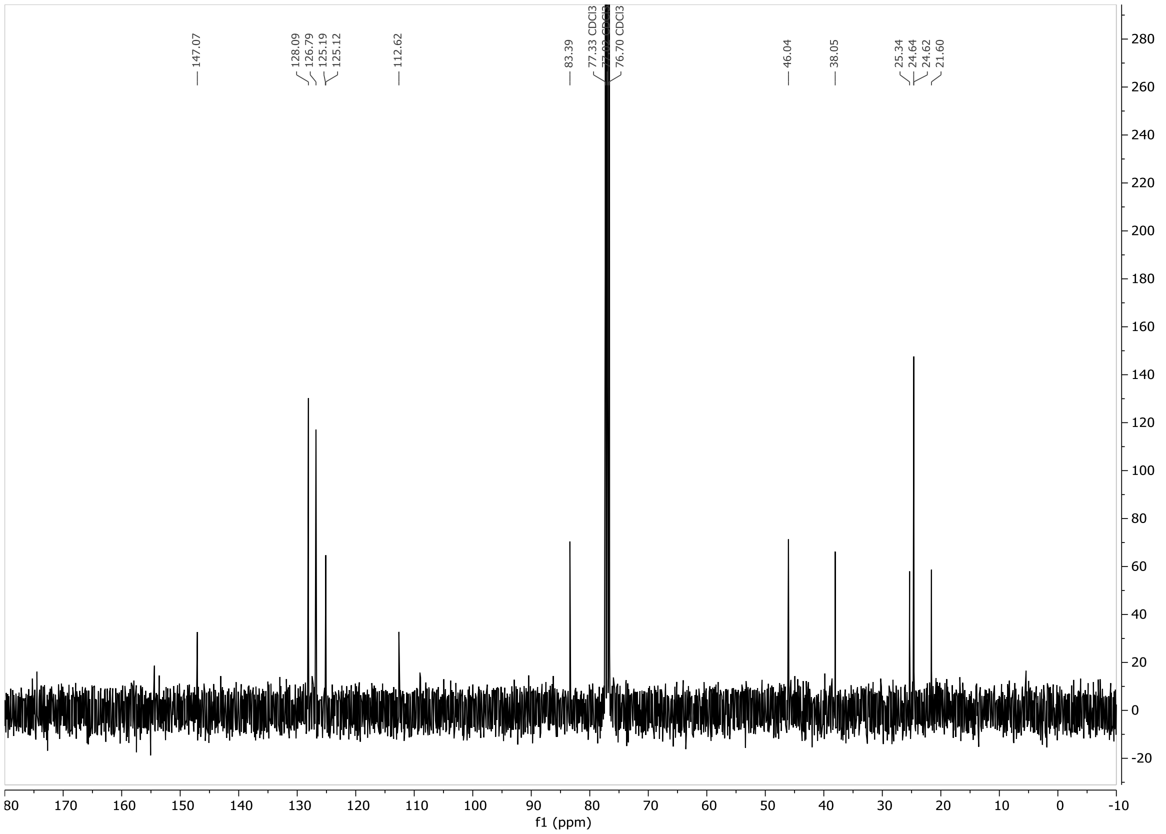

^{1}H NMR (400 MHz, $CDCl_3$) of **1a’** (*see procedure*)

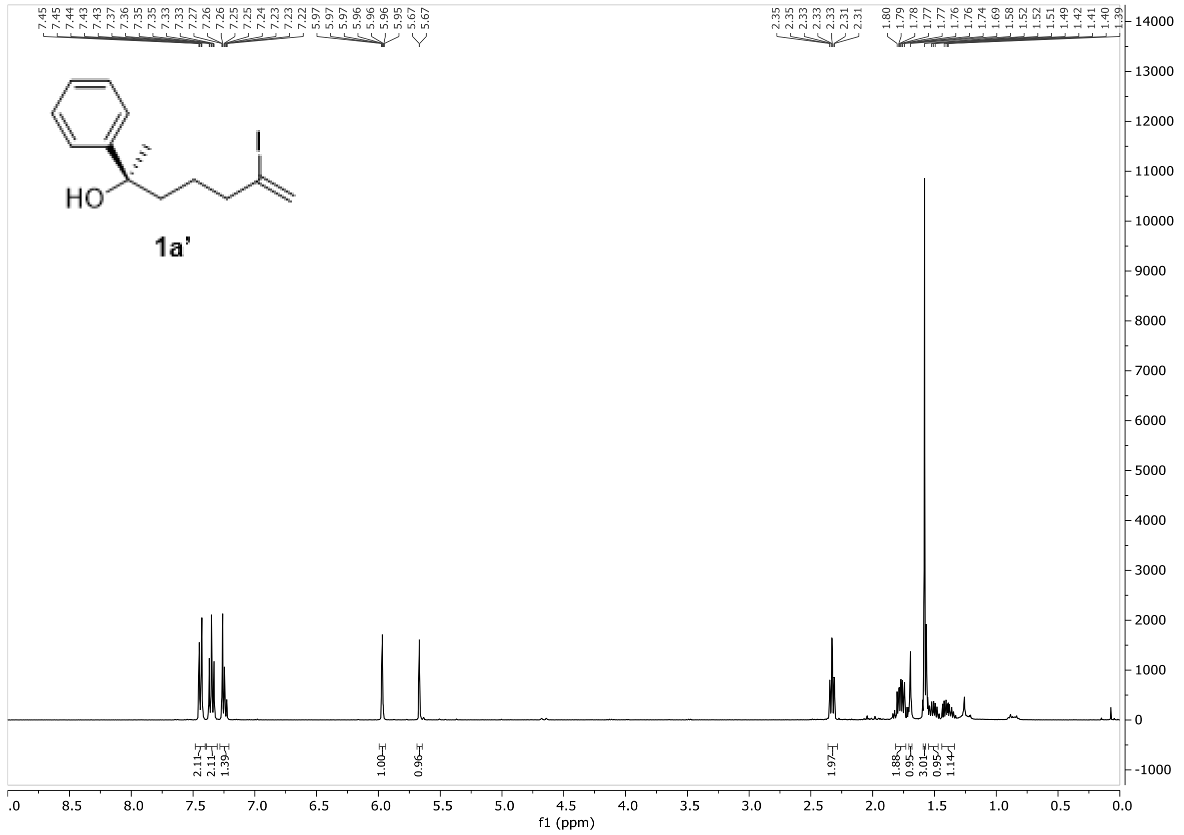

^{13}C NMR (101 MHz, $CDCl_3$) of **1a’**

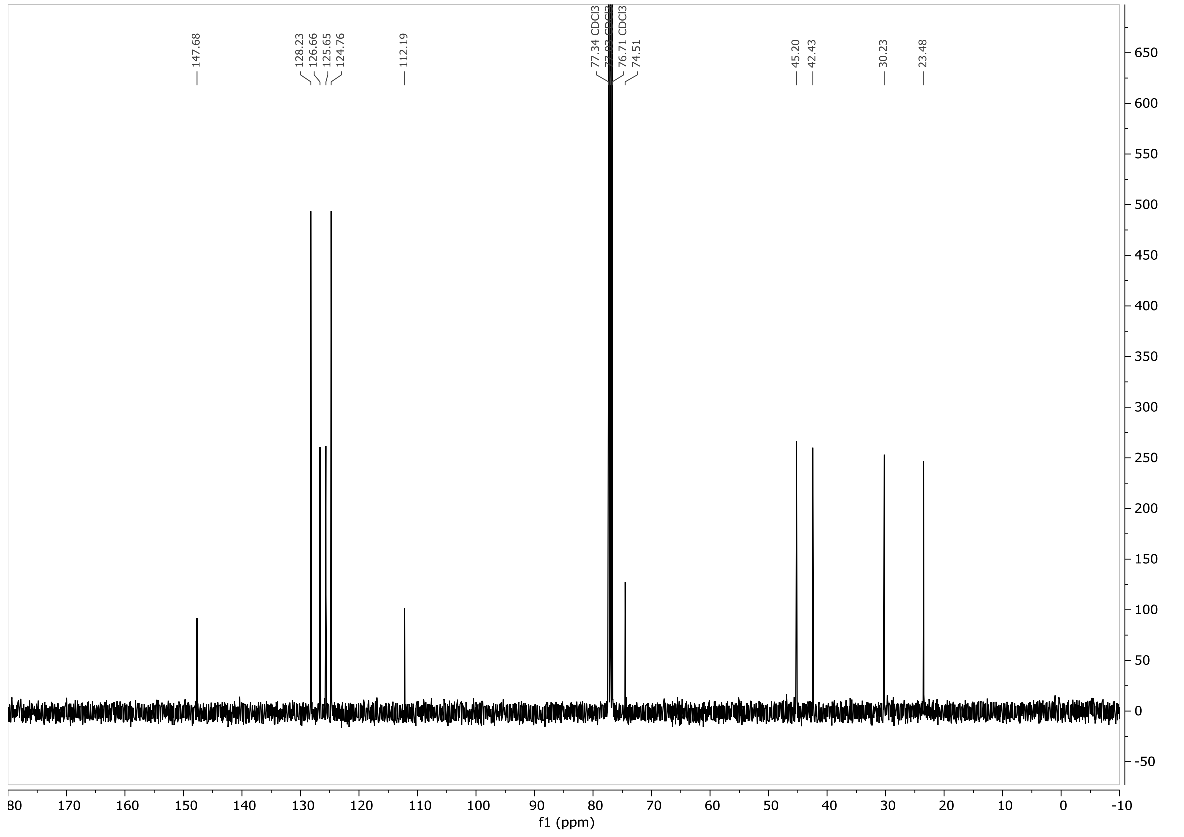

^{1}H NMR (400 MHz, $CDCl_3$) of **1h** *(see procedure)*

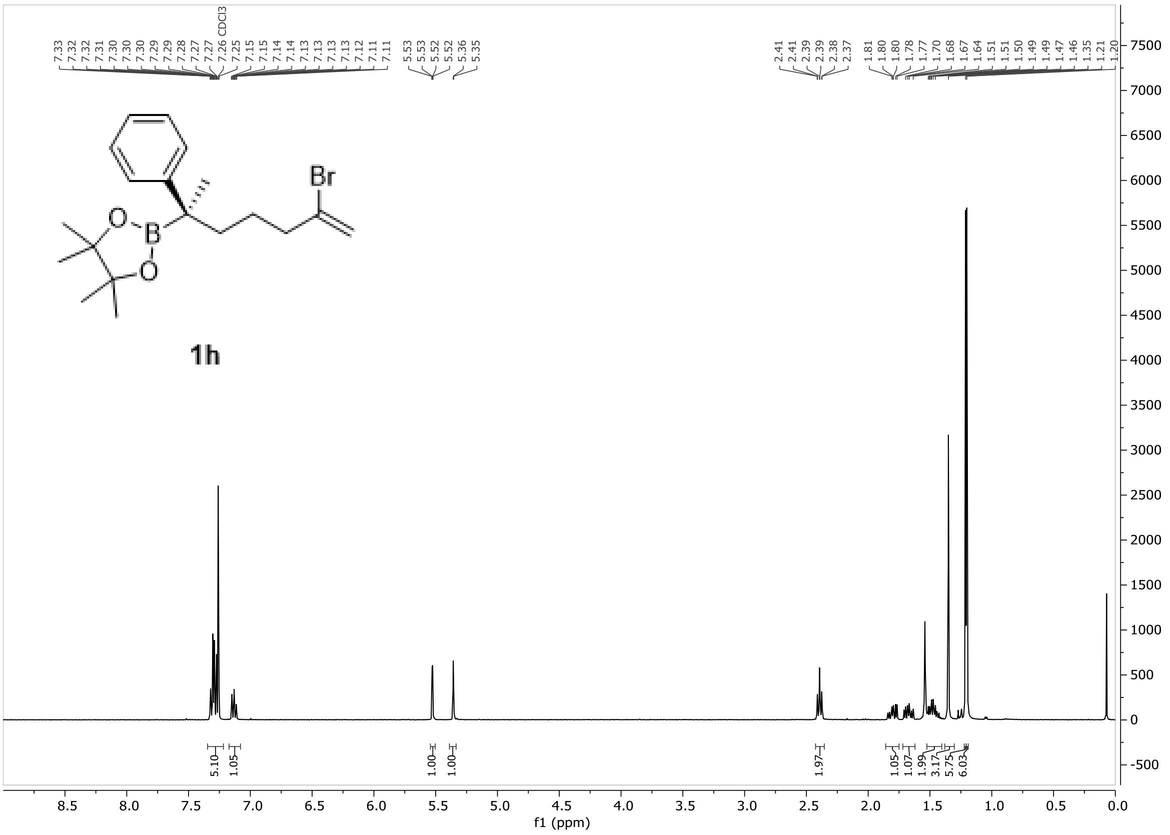

^{13}C NMR (101 MHz, $CDCl_3$) of **1h**

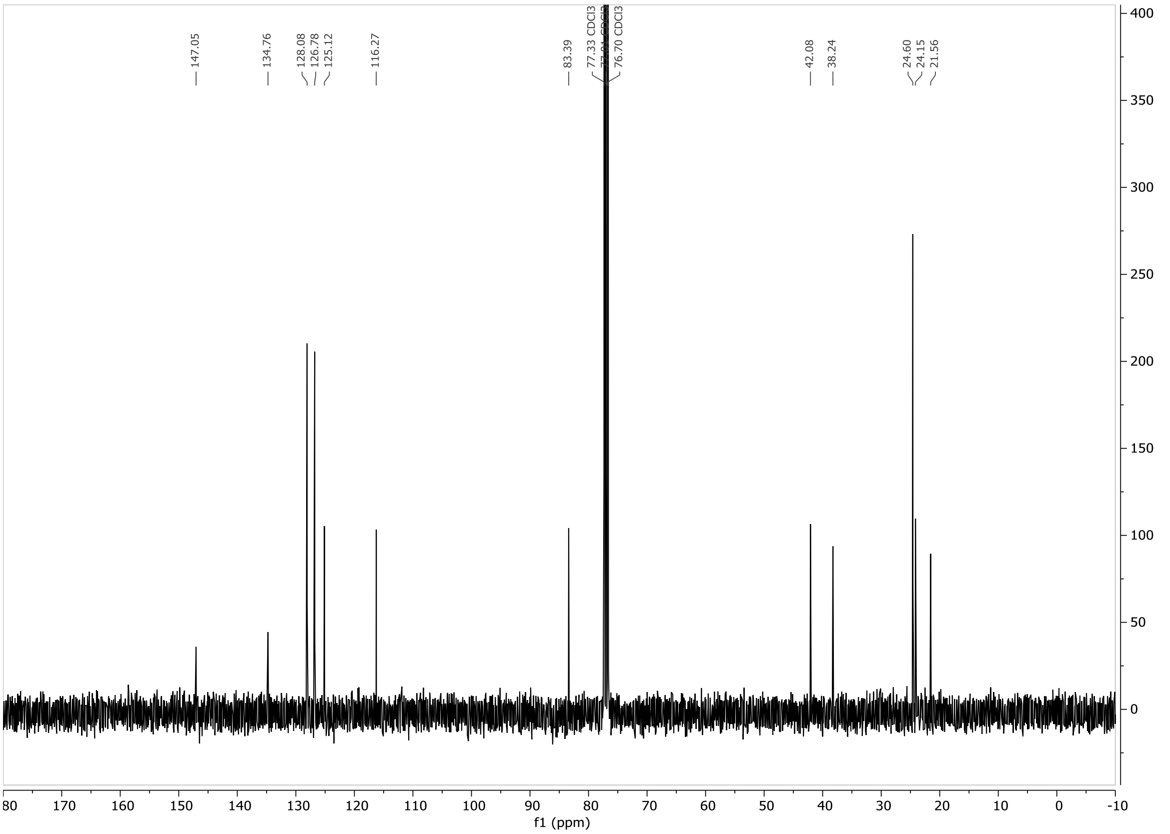

^{1}H NMR (400 MHz, $CDCl_3$) of **1h’** *(see procedure)*

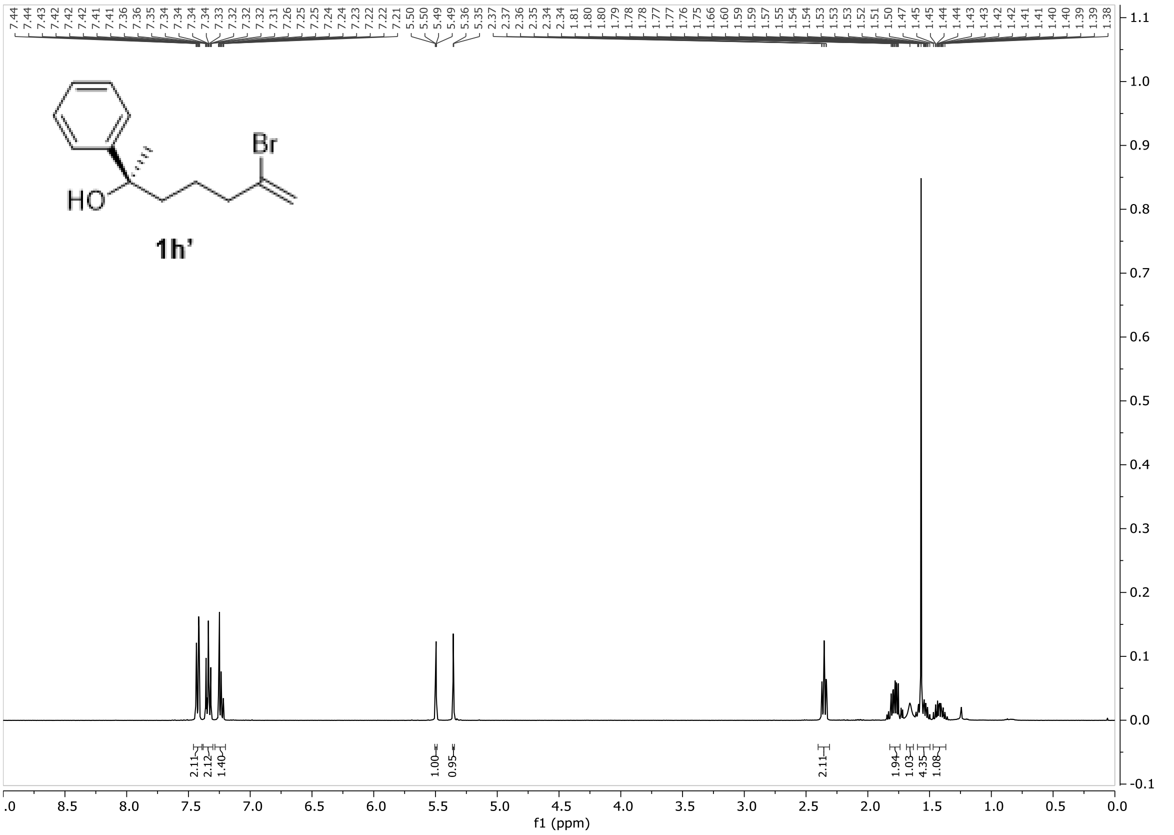

^{13}C NMR (101 MHz, $CDCl_3$) of **1h’**

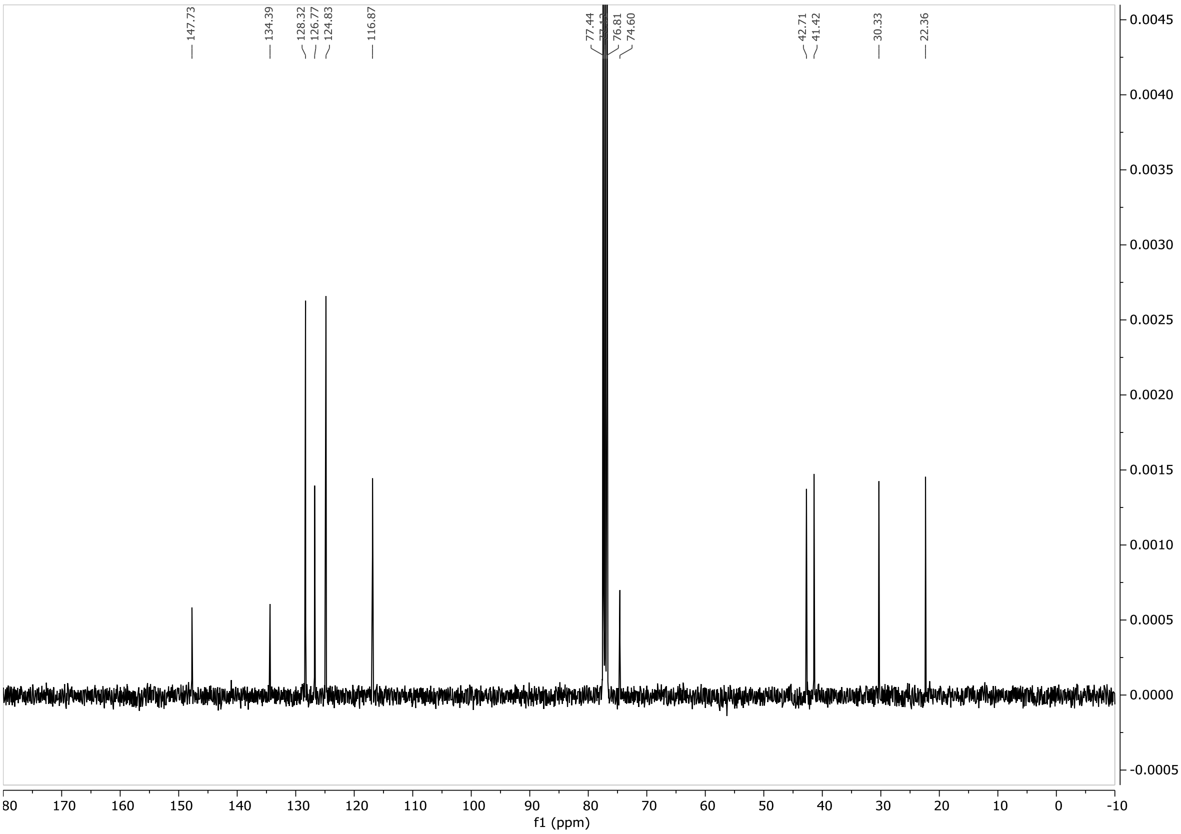

^{1}H NMR (400 MHz, $CDCl_3$) of **1b** *(see procedure)*

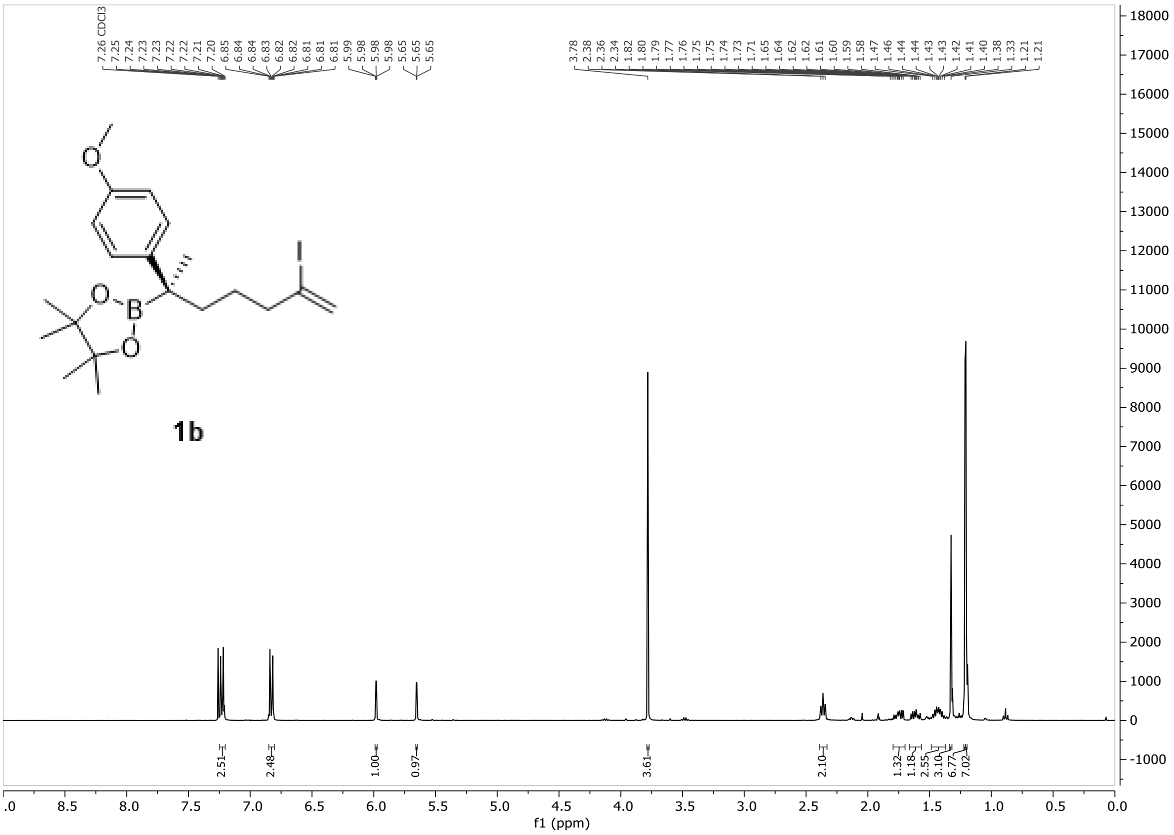

^{13}C NMR (101 MHz, $CDCl_3$) of **1b**

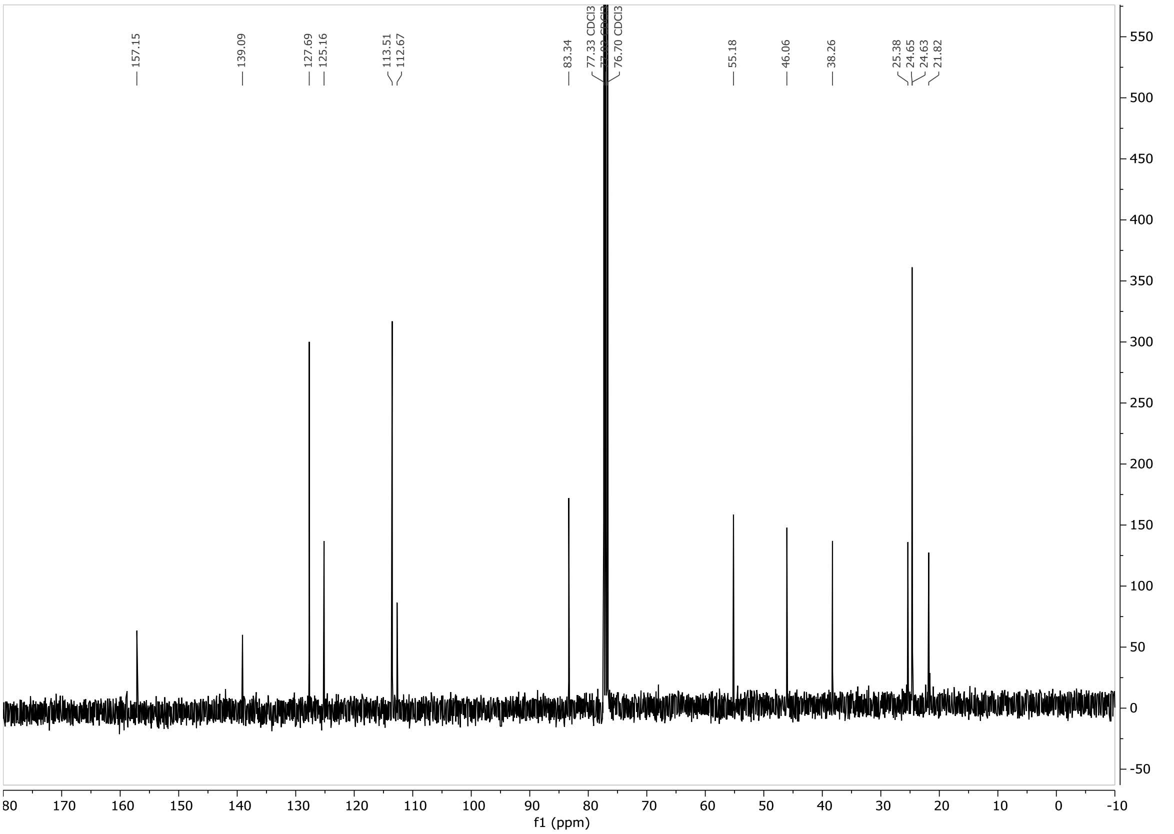

^{1}H NMR (400 MHz, $CDCl_3$) of **1b'** *(<u>see procedure</u>)*

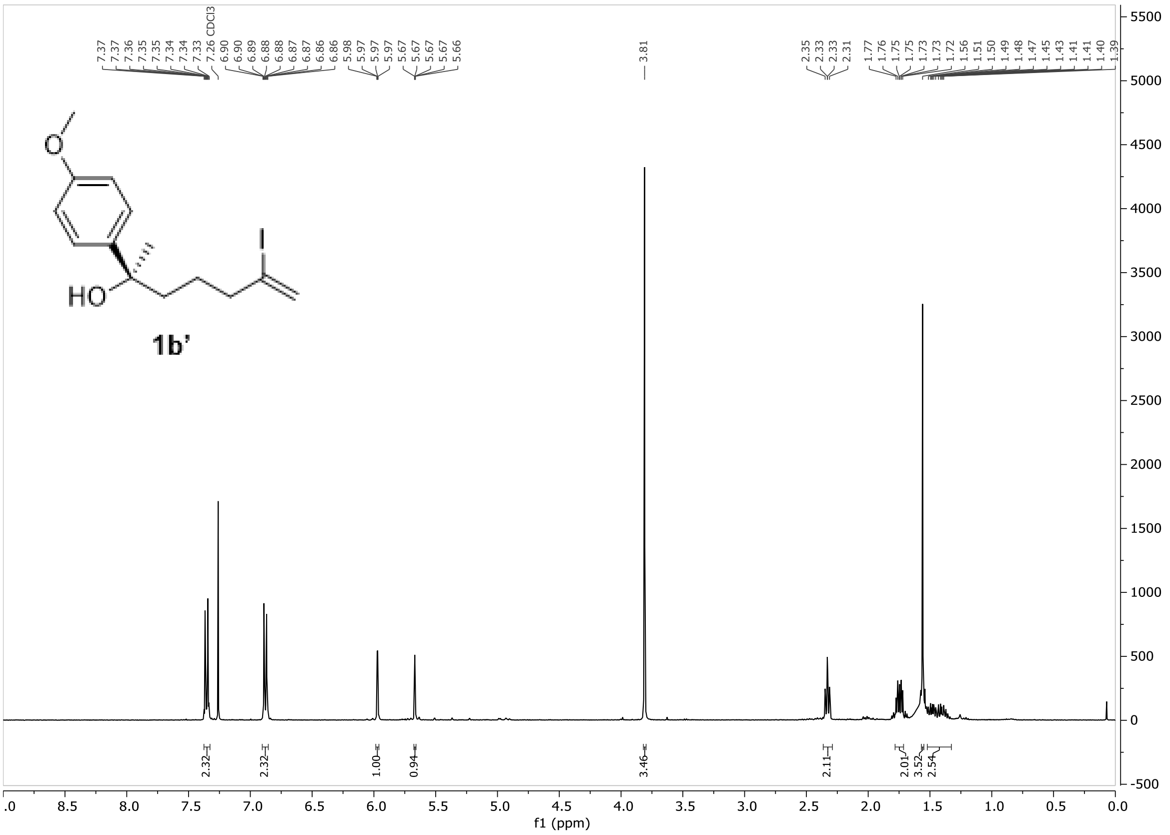

^{13}C NMR (101 MHz, $CDCl_3$) of **1b'**

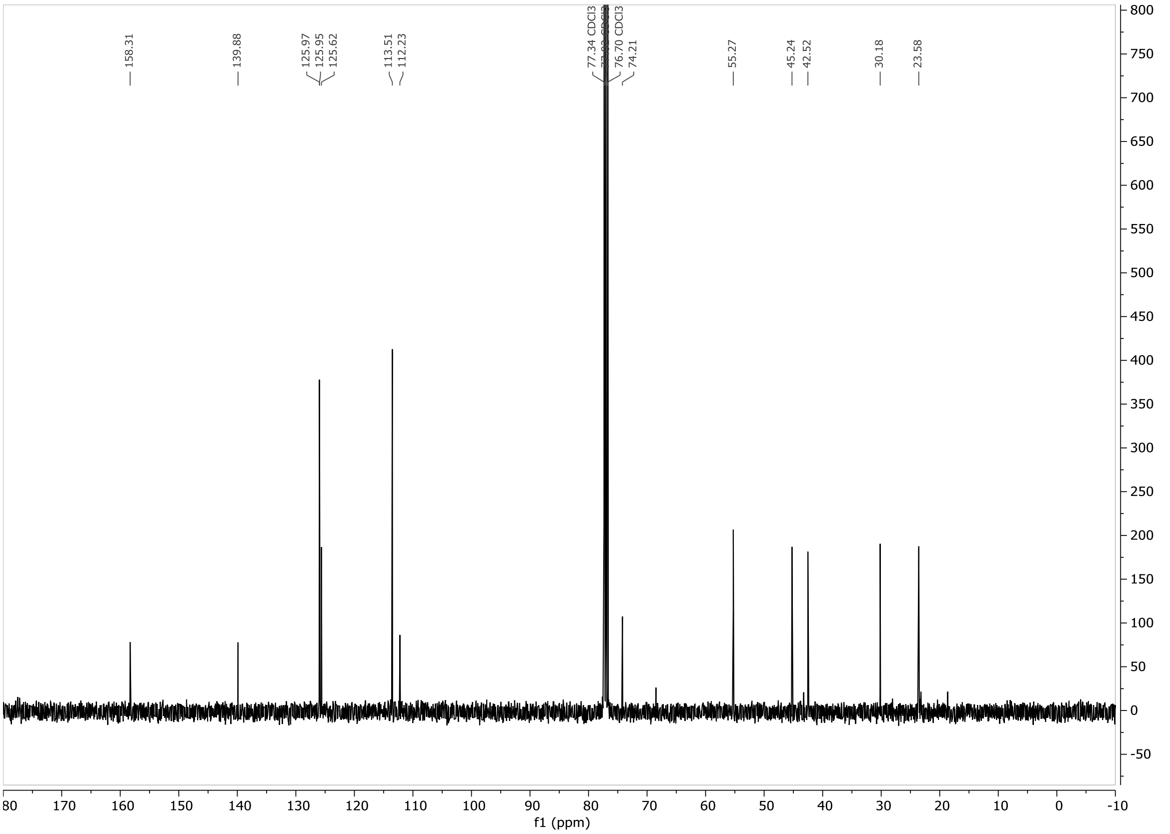

^{1}H NMR (400 MHz, $CDCl_3$) of **1c** *(see procedure)*

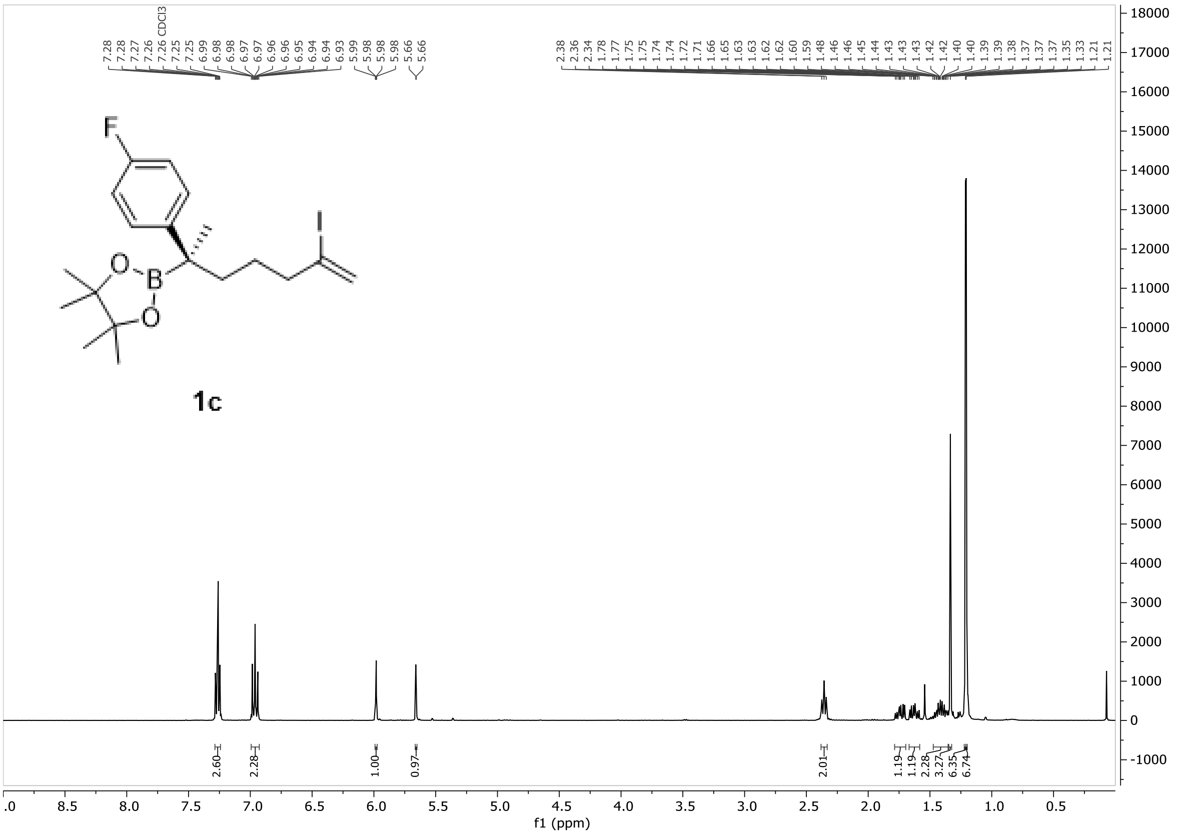

^{13}C NMR (101 MHz, $CDCl_3$) of **1c**

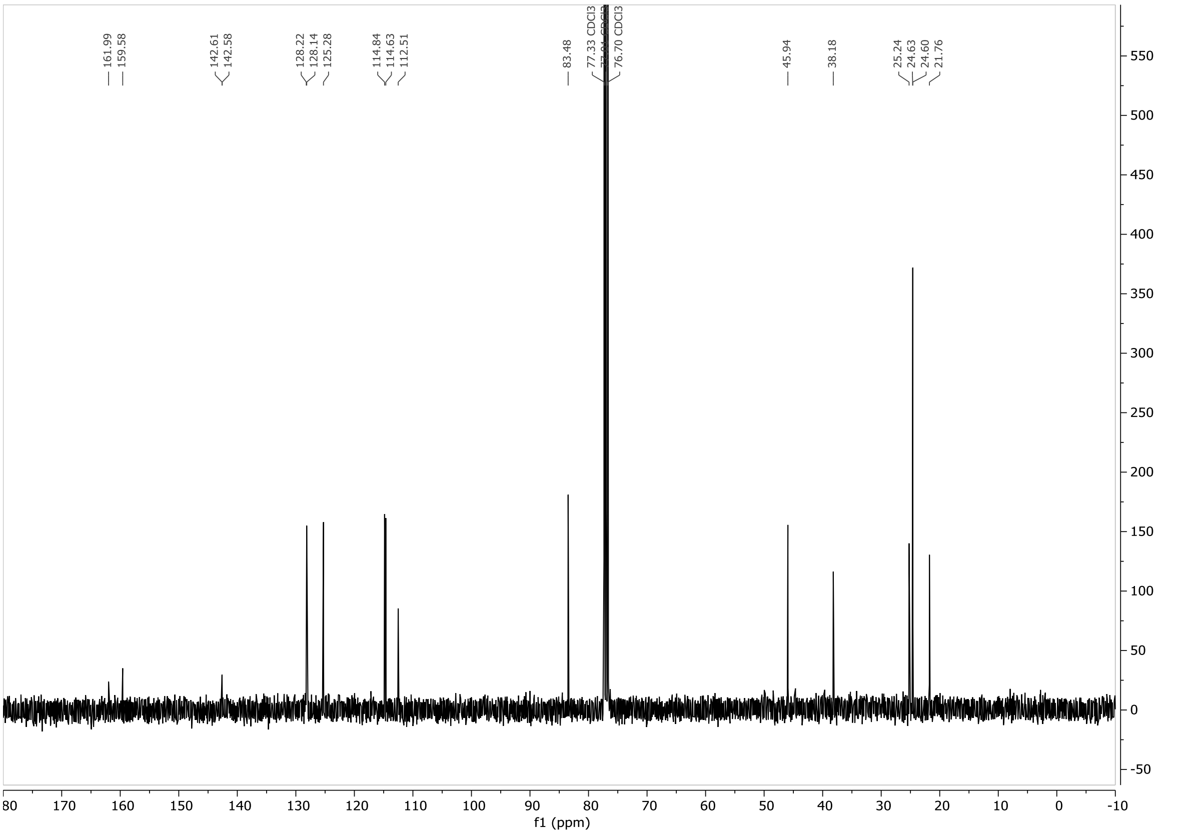

^{1}H NMR (400 MHz, $CDCl_3$) of **1c'** *(<u>see procedure</u>)*

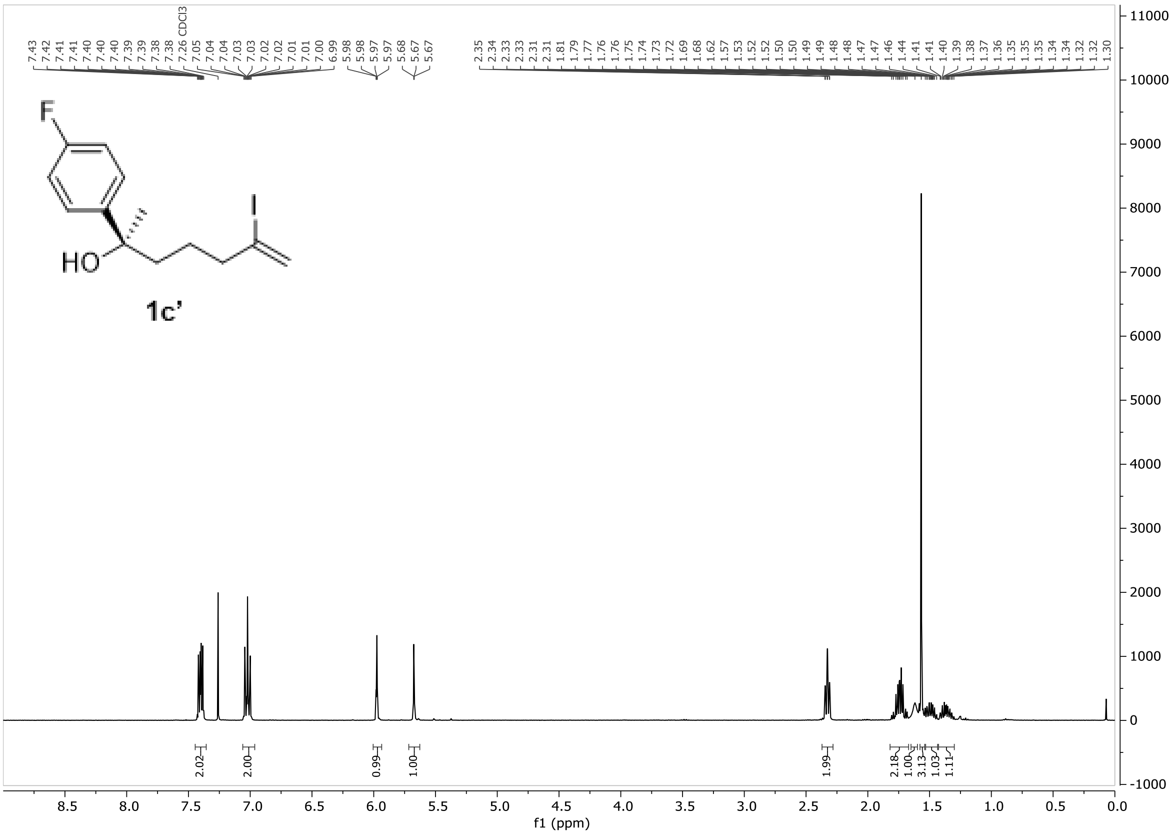

^{13}C NMR (101 MHz, $CDCl_3$) of **1c'**

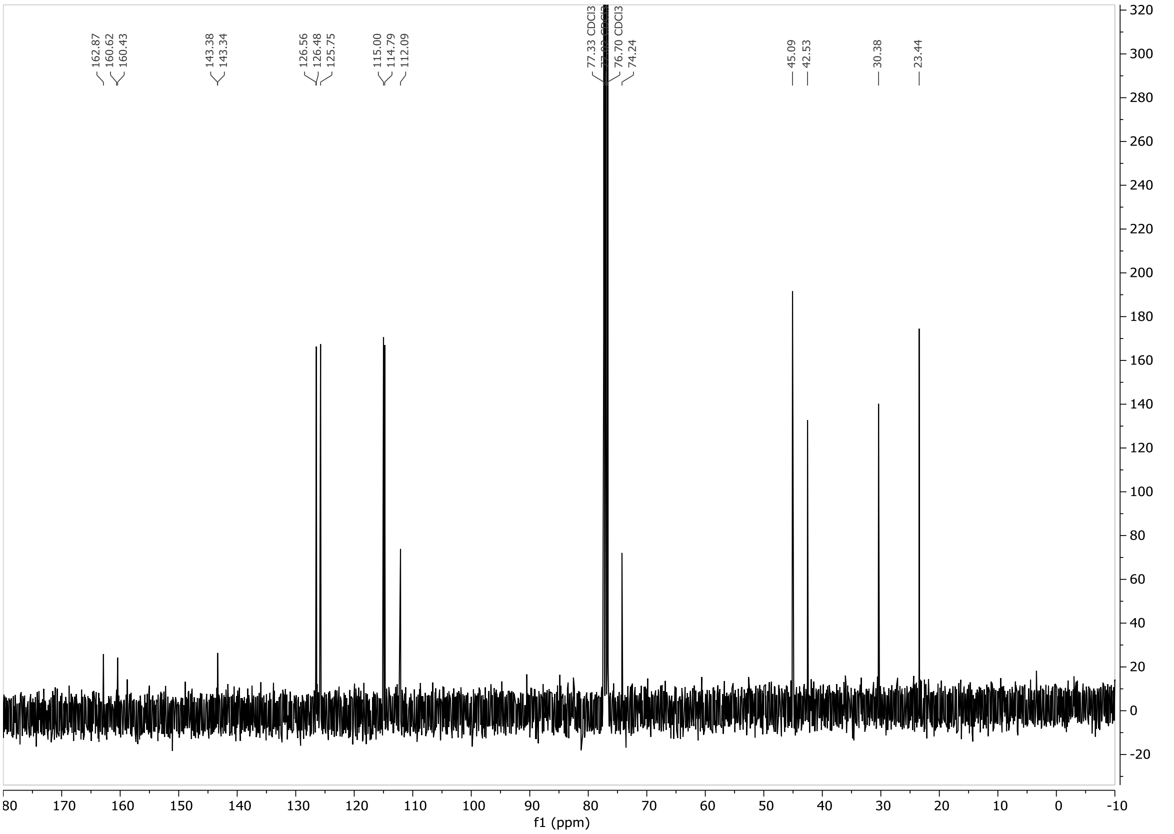

^{1}H NMR (400 MHz, $CDCl_3$) of **1d** *(see procedure)*

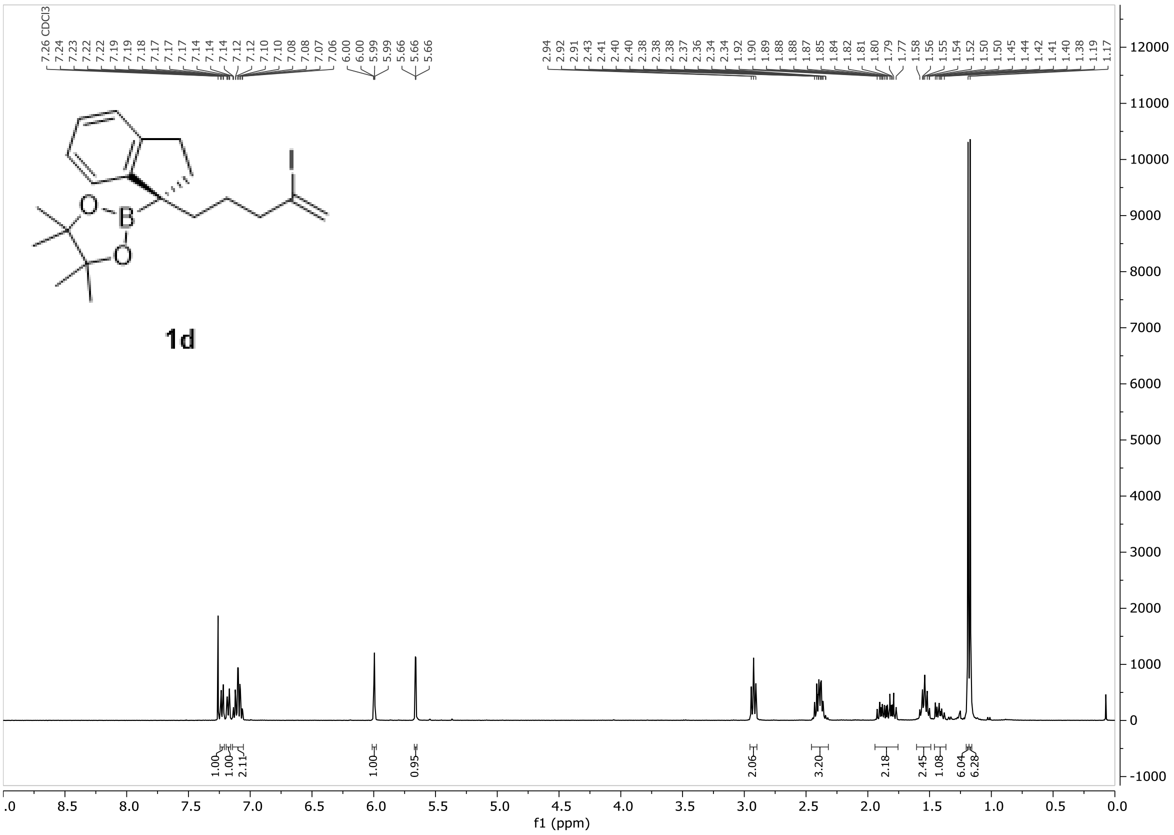

^{13}C NMR (101 MHz, $CDCl_3$) of **1d**

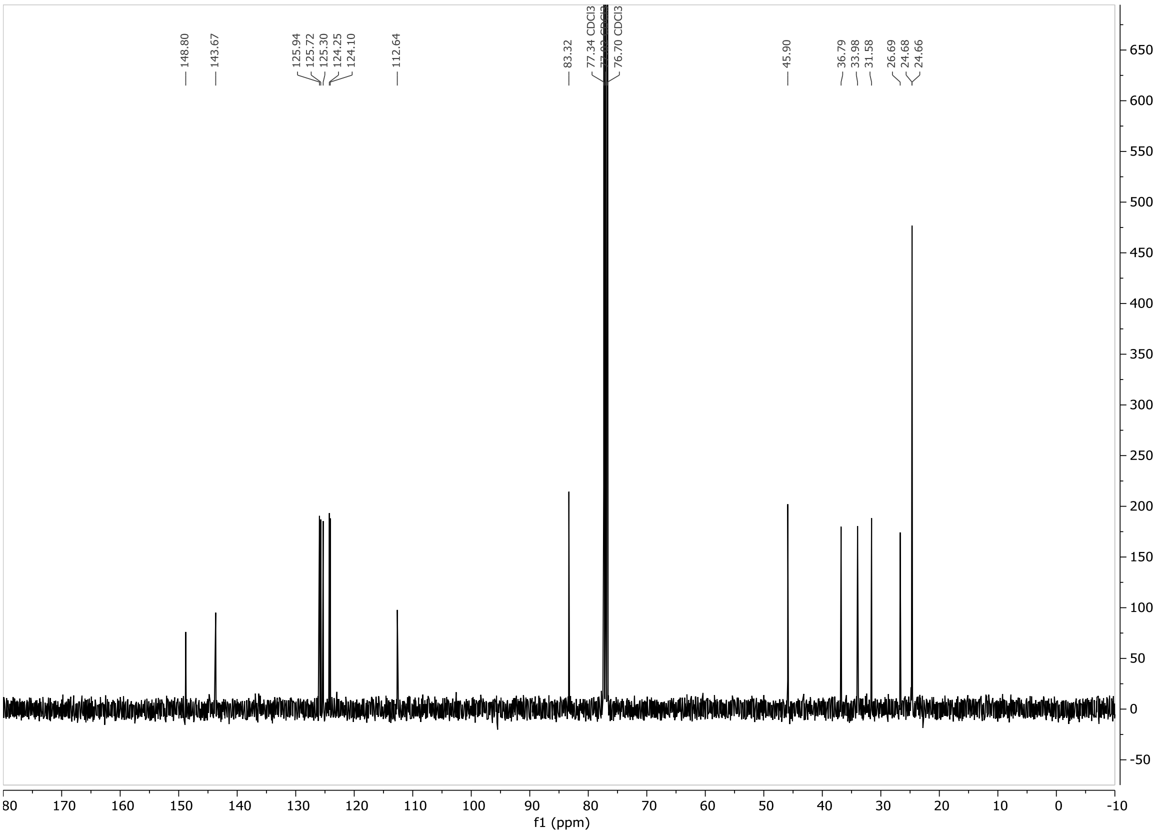

^{1}H NMR (400 MHz, CD_2Cl_2) of **1d’** *(<u>see procedure</u>)*

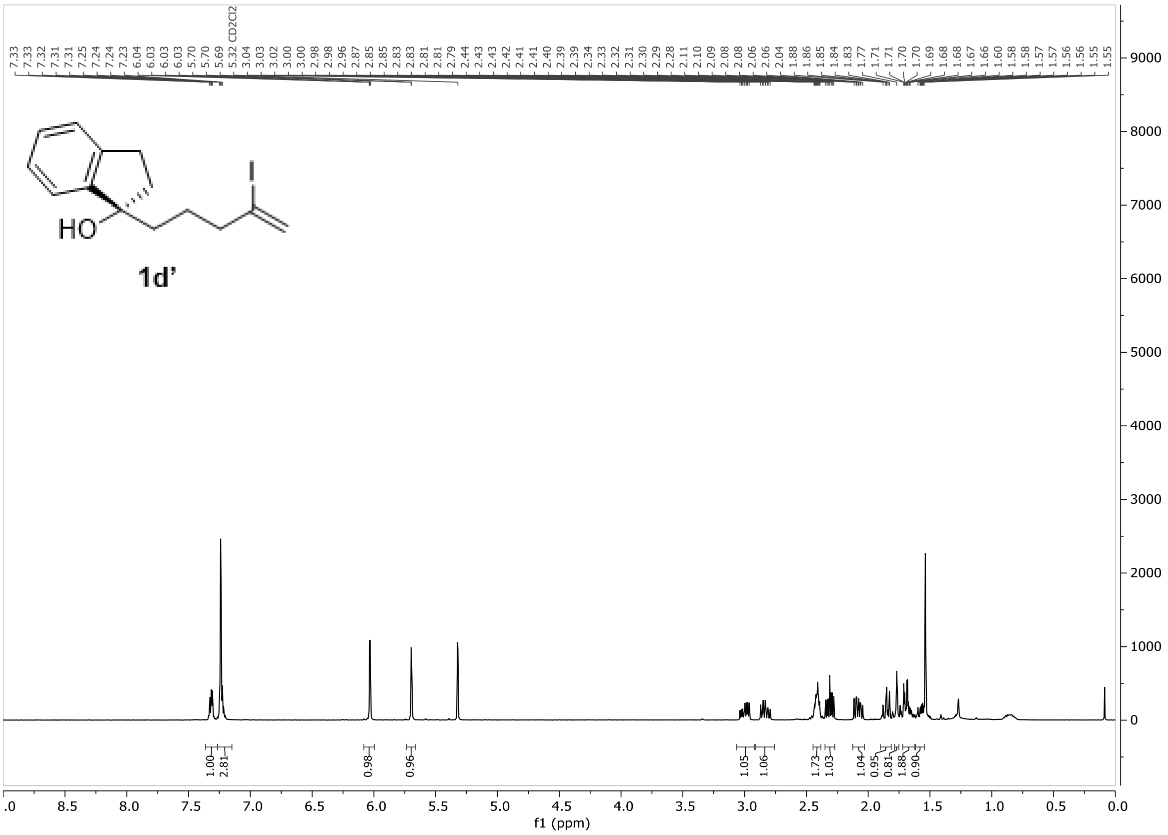

^{13}C NMR (101 MHz, CD_2Cl_2) of **1d’**

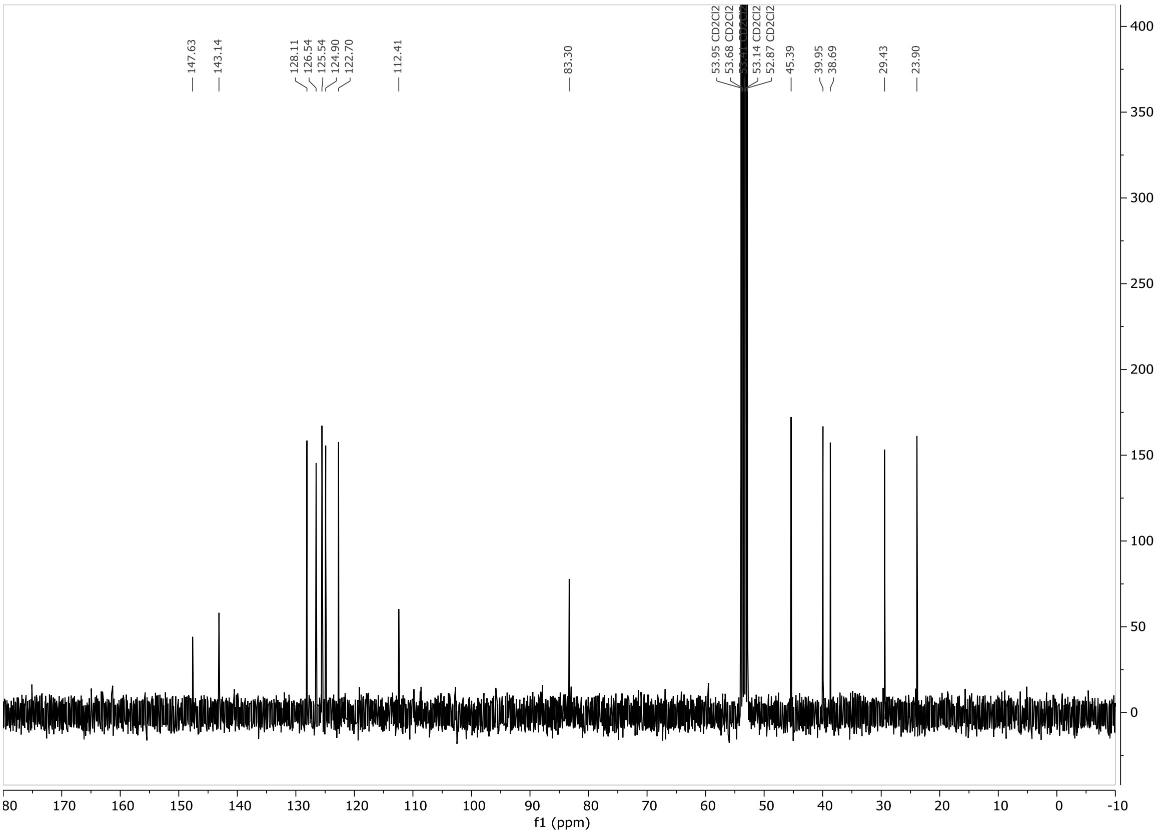

^{1}H NMR (400 MHz, $CDCl_3$) of **1e** *(see procedure)*

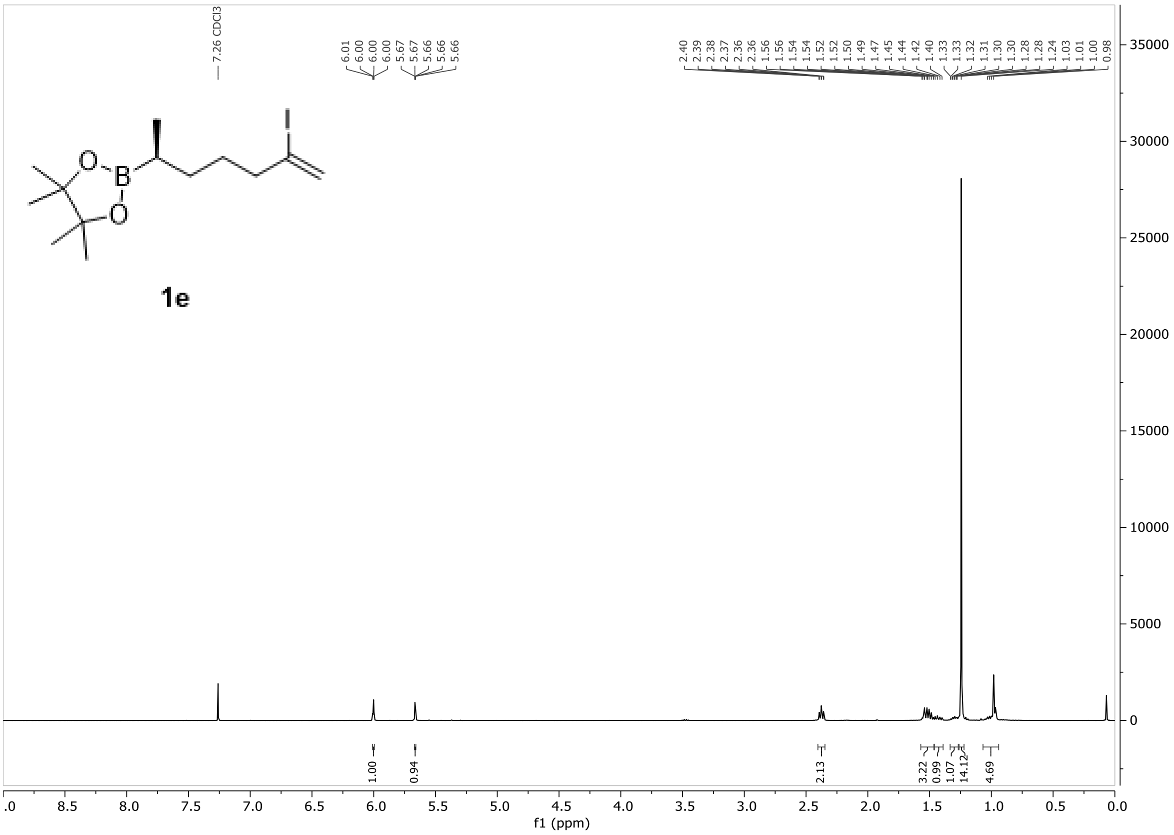

^{13}C NMR (101 MHz, $CDCl_3$) of **1e**

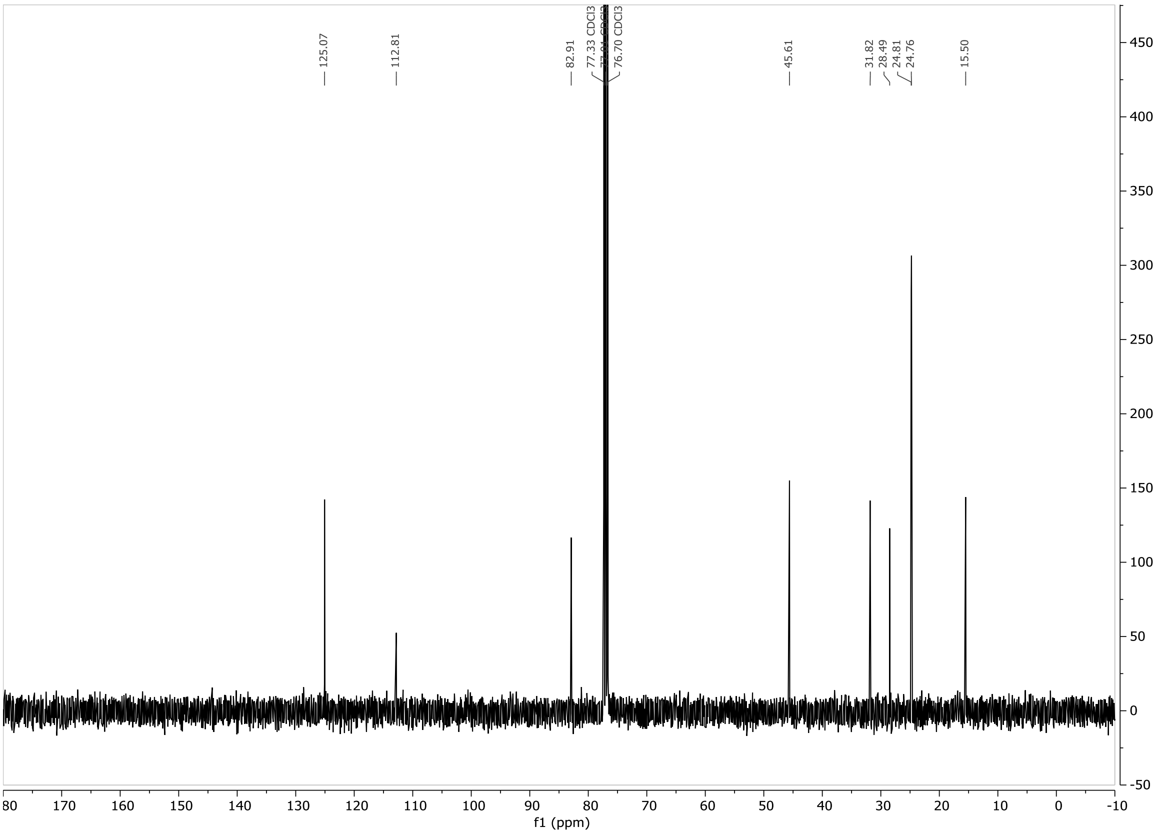

^{1}H NMR (400 MHz, $CDCl_3$) of **1e’** *(<u>see procedure</u>)*

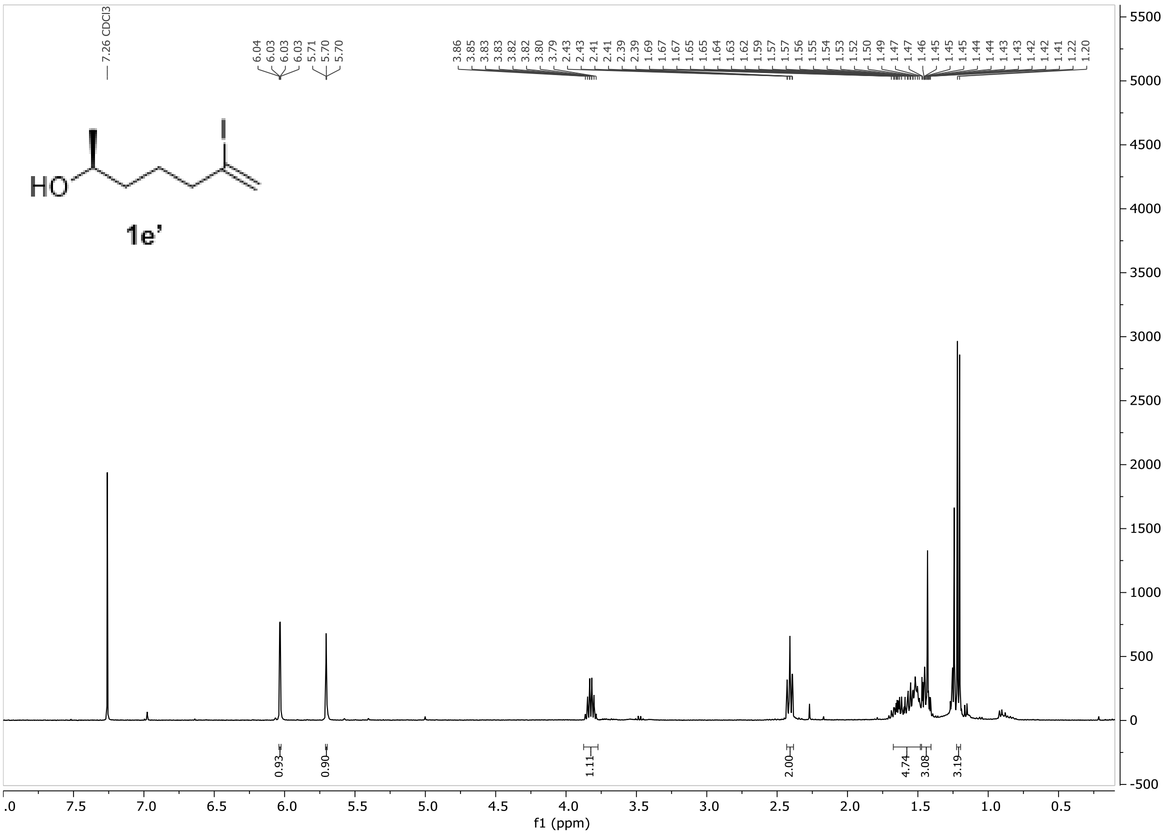

^{13}C NMR (101 MHz, $CDCl_3$) of **1e’**

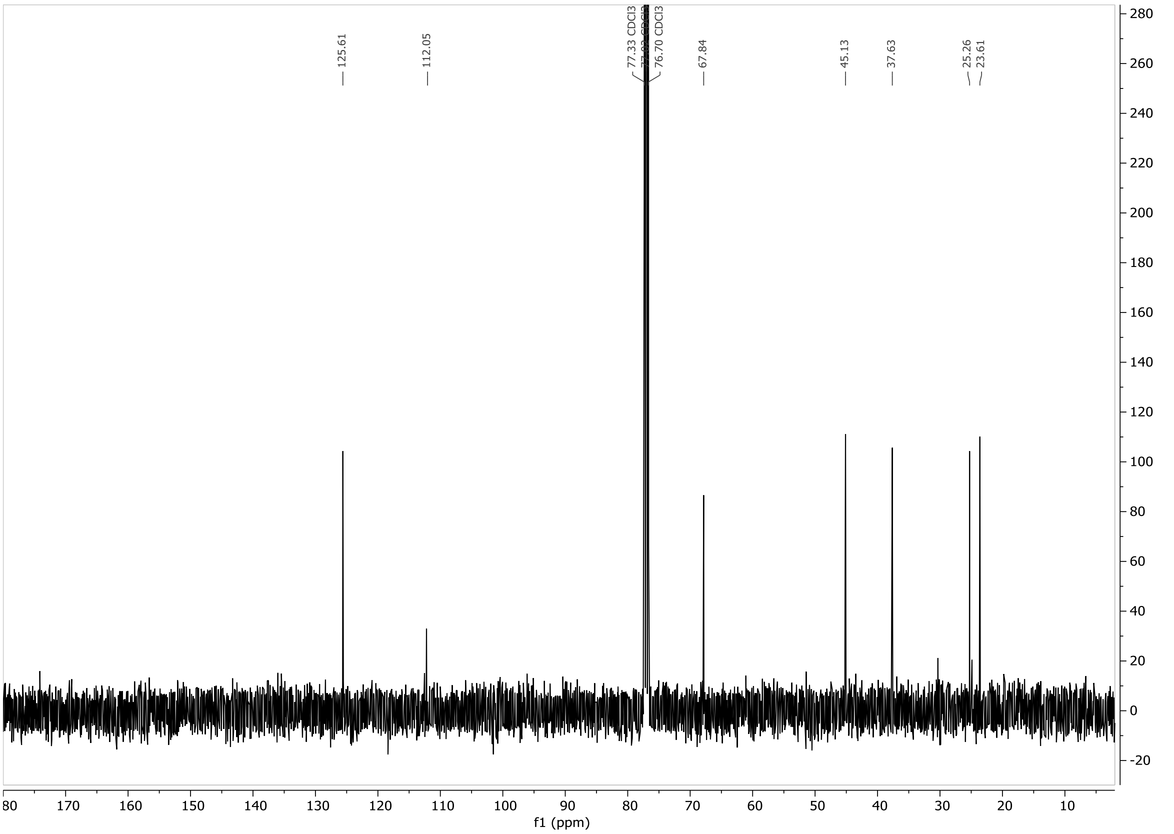

^{1}H NMR (400 MHz, $CDCl_3$) of **1f** *(see procedure)*

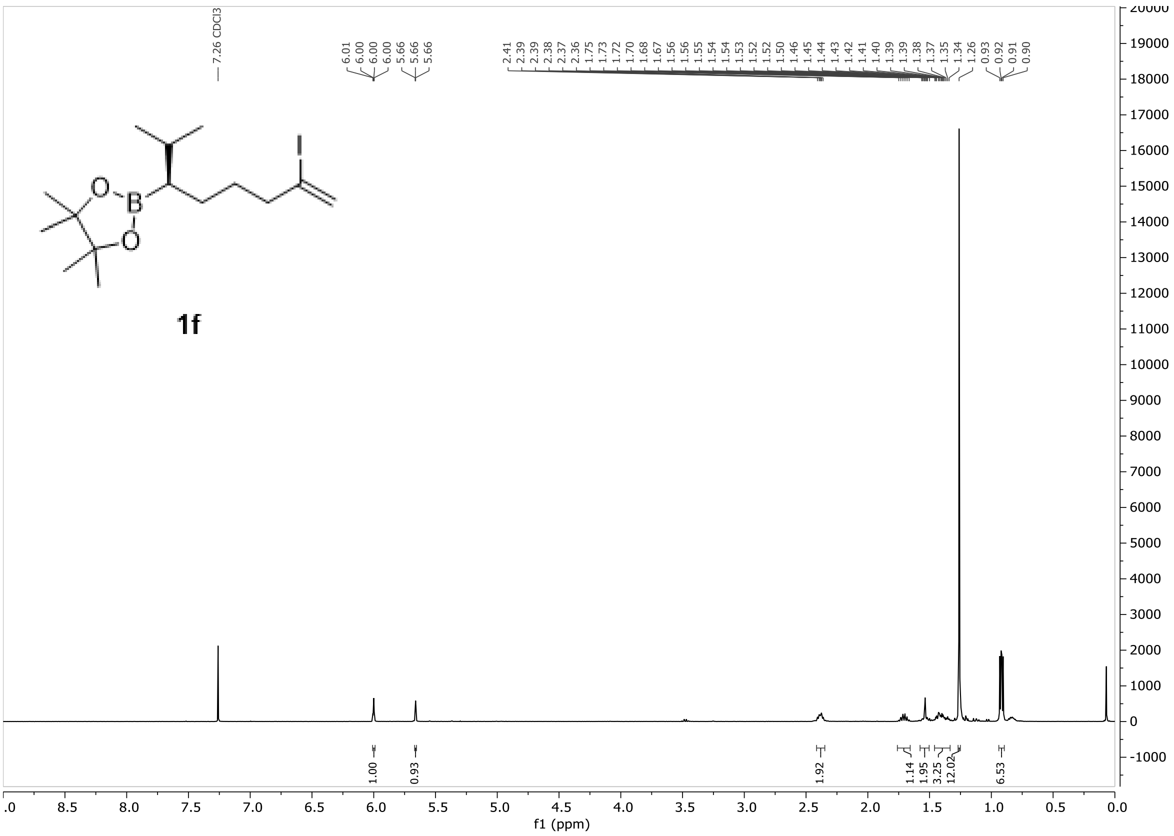

^{13}C NMR (101 MHz, $CDCl_3$) of **1f**

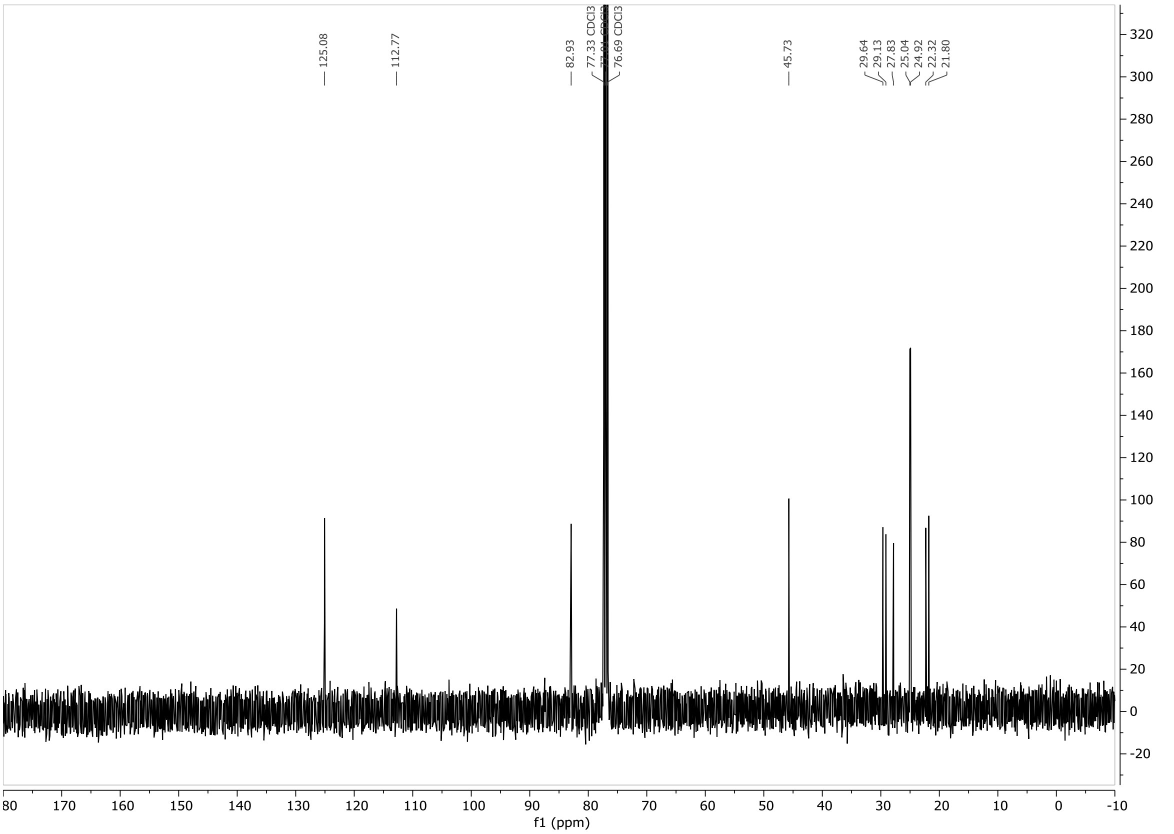

^{1}H NMR (400 MHz, $CDCl_3$) of **1f’** *(see procedure)*

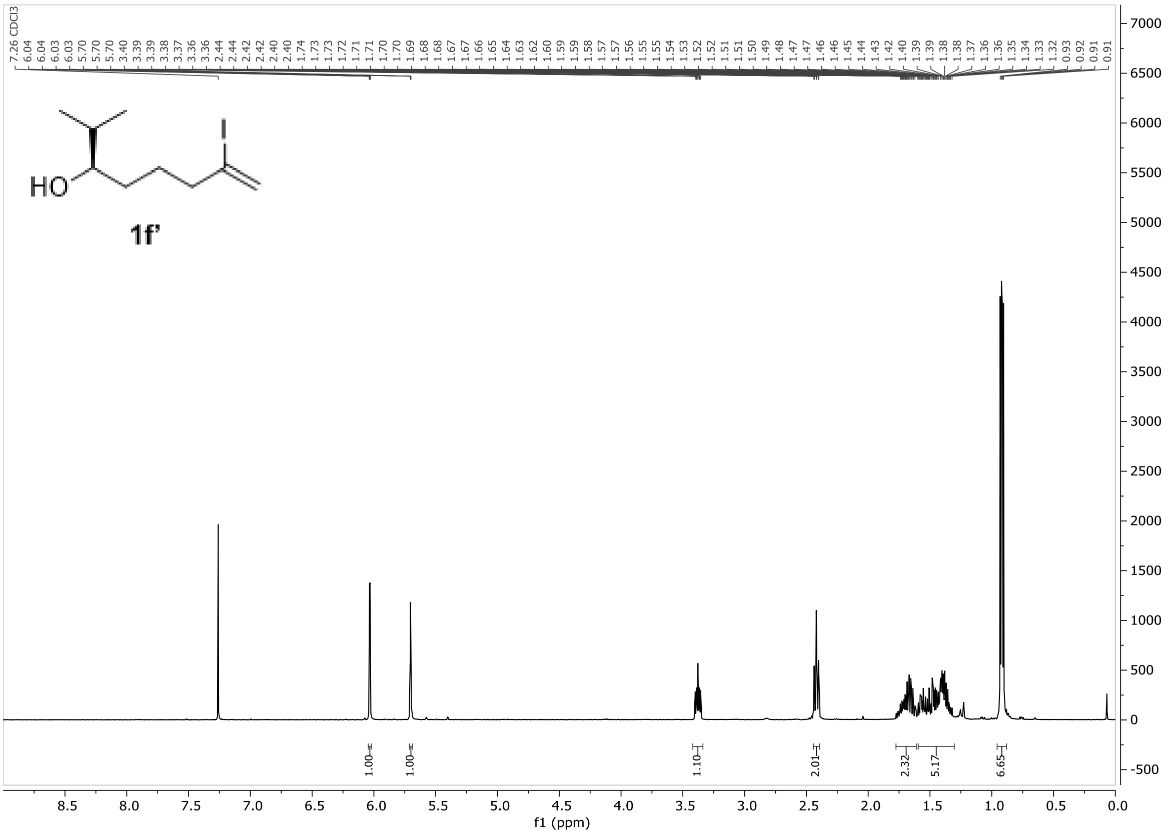

^{13}C NMR (101 MHz, $CDCl_3$) of **1f’**

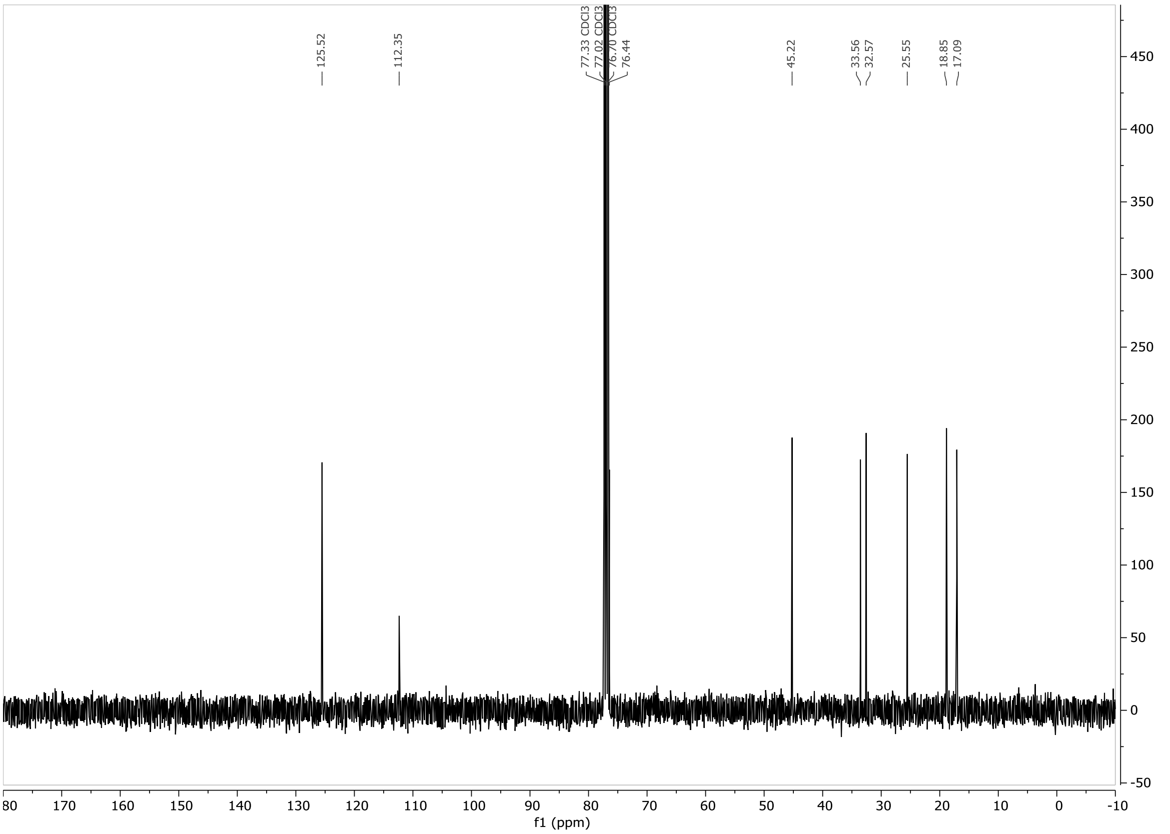

^{1}H NMR (400 MHz, $CDCl_3$) of **1g** *(see procedure)*

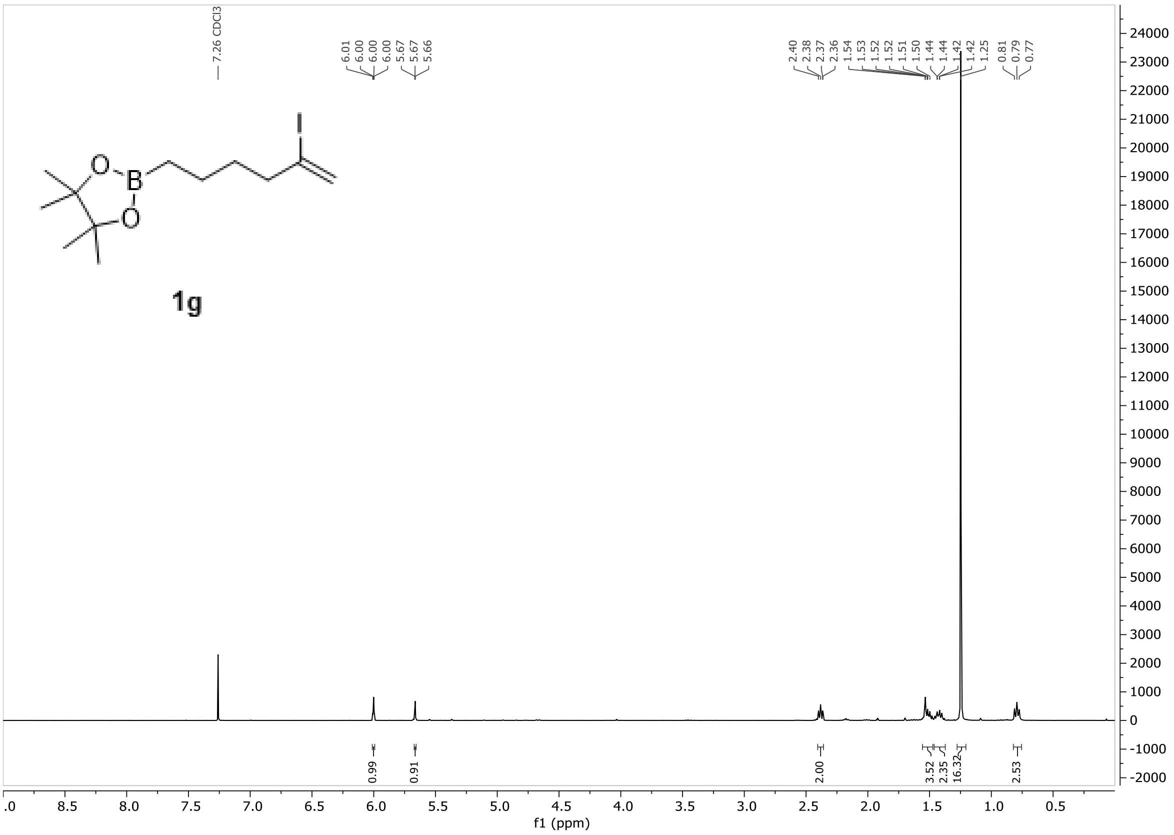

^{13}C NMR (101 MHz, $CDCl_3$) of **1g**

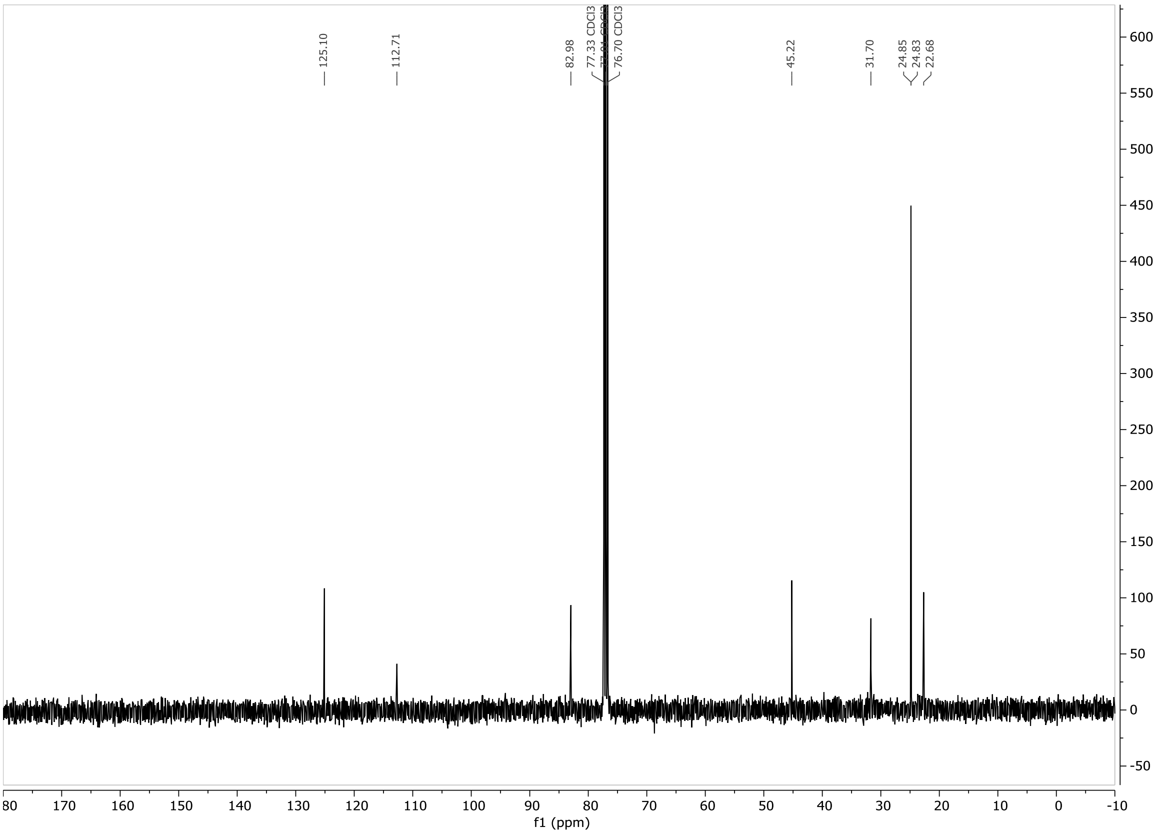

^{1}H NMR (400 MHz, $CDCl_3$) of **3a.d[1]** *(see procedure)*

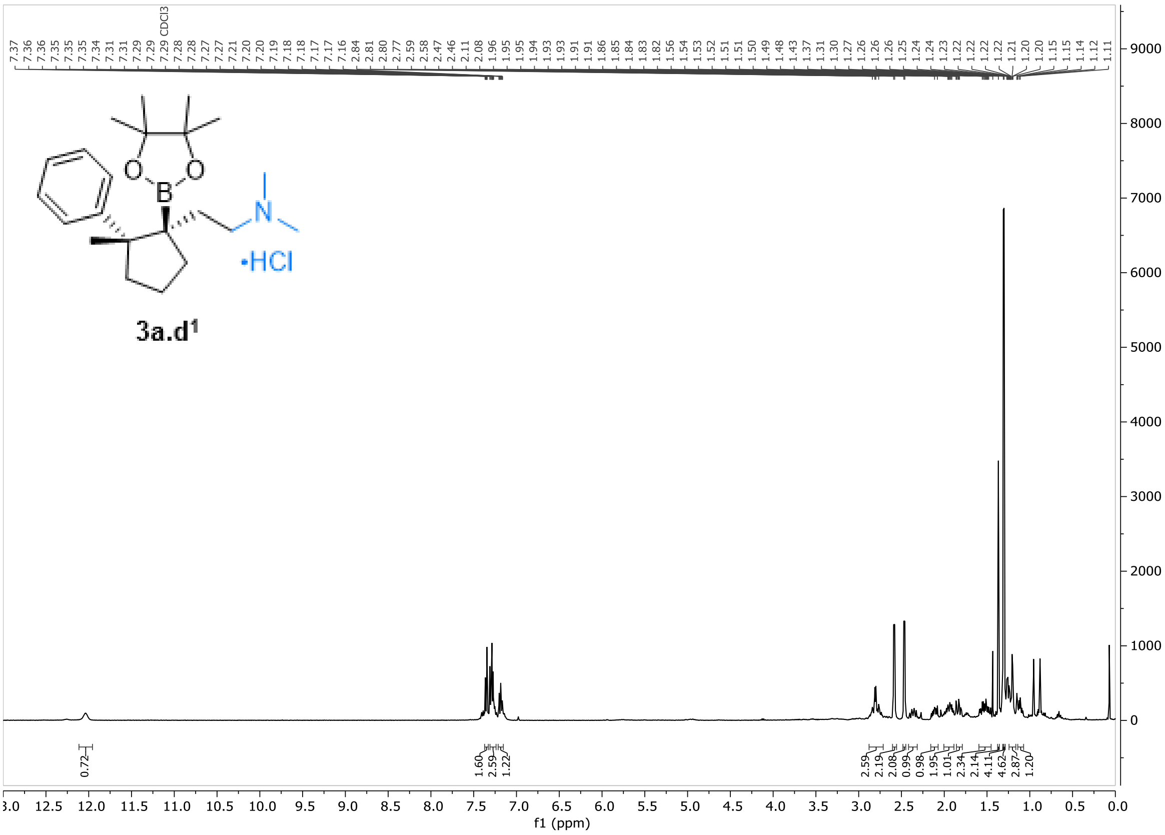

^{13}C NMR (101 MHz, $CDCl_3$) of **3a.d[1]**

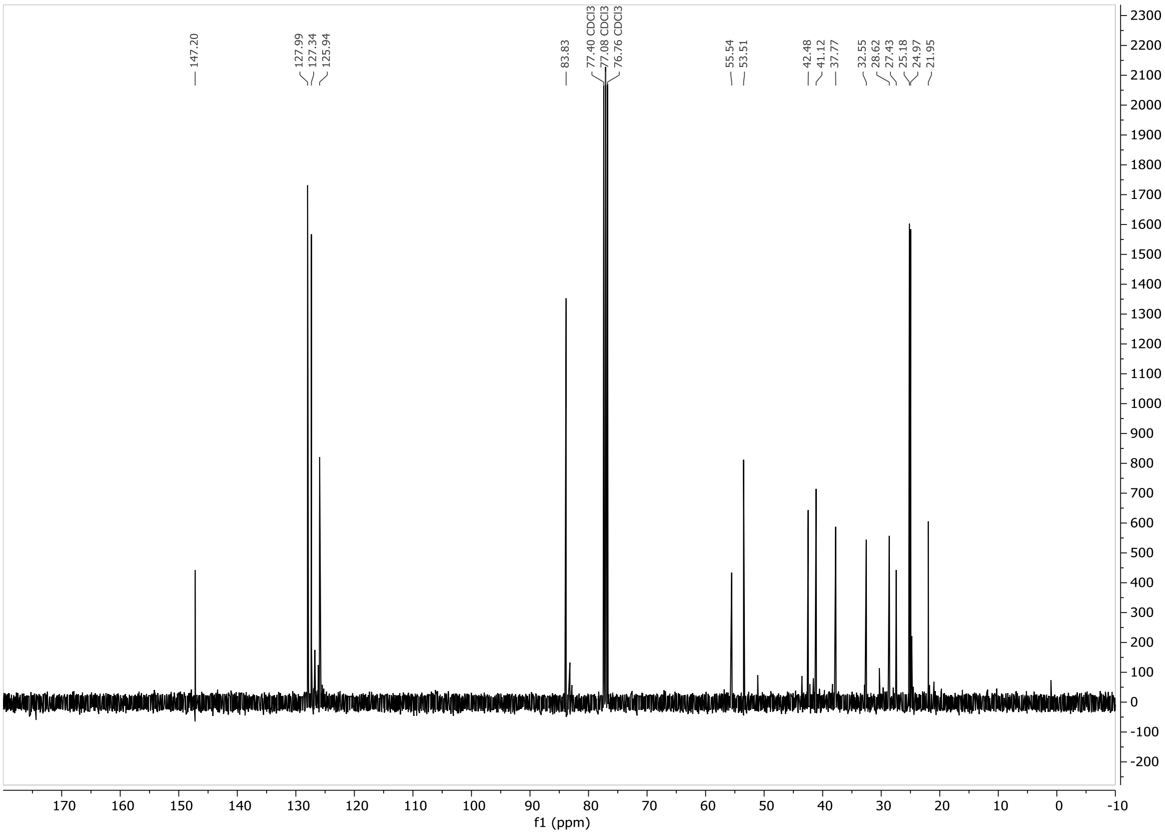

^{1}H NMR (400 MHz, $CDCl_3$) of **3a.d^2** *(<u>see procedure</u>)*

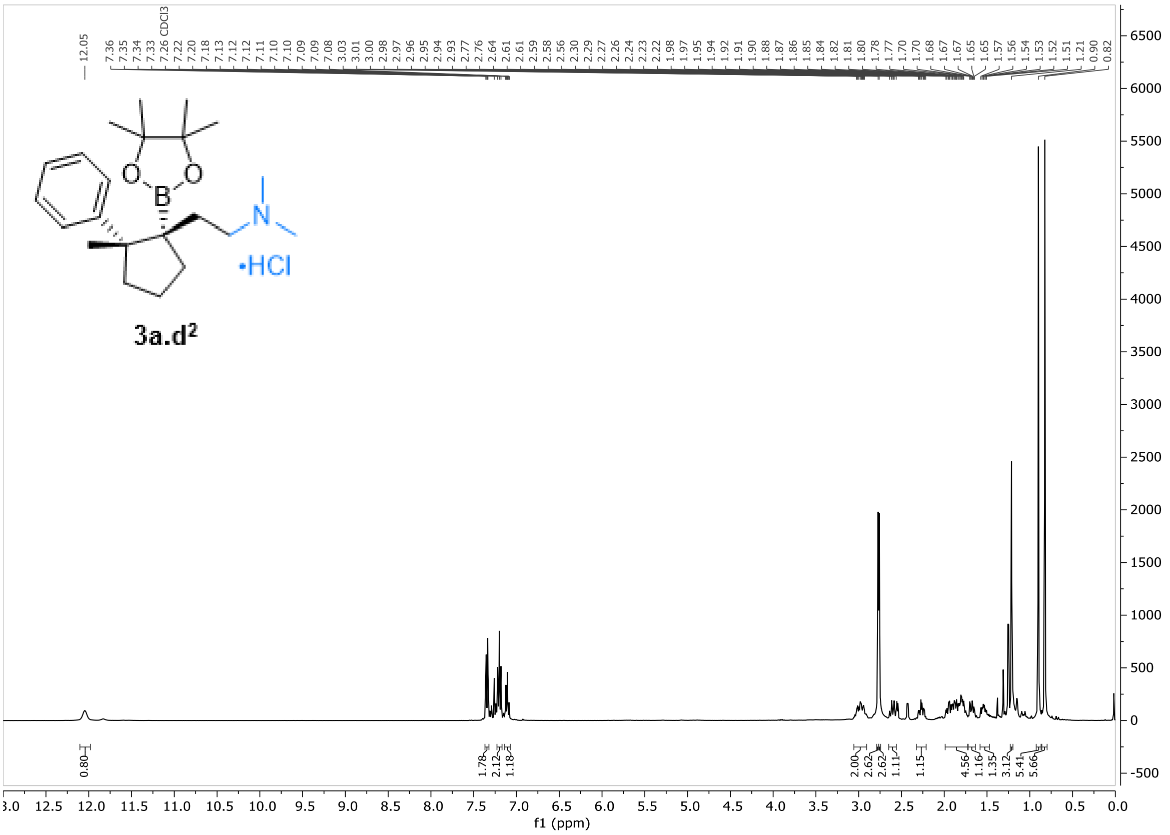

^{13}C NMR (101 MHz, $CDCl_3$) of **3a.d^2**

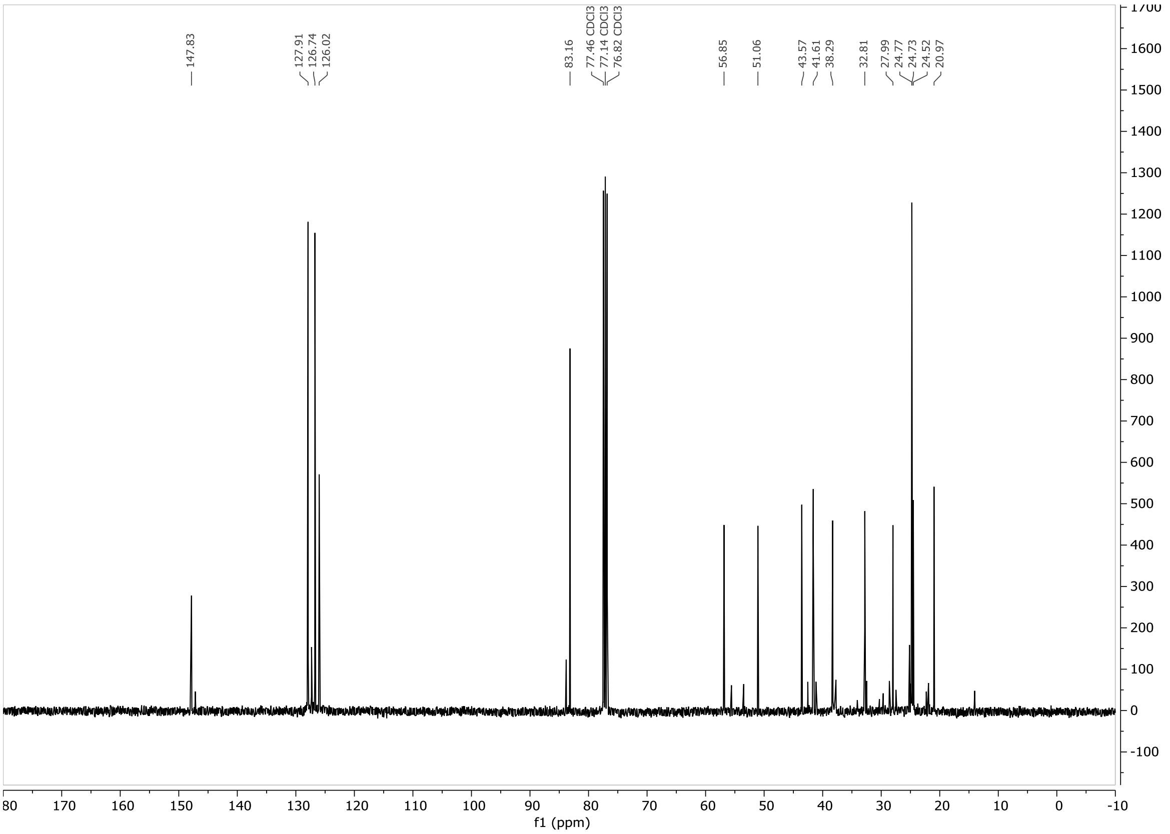

^{1}H NMR (400 MHz, $CDCl_3$) of **3b.d¹** (*see procedure*)

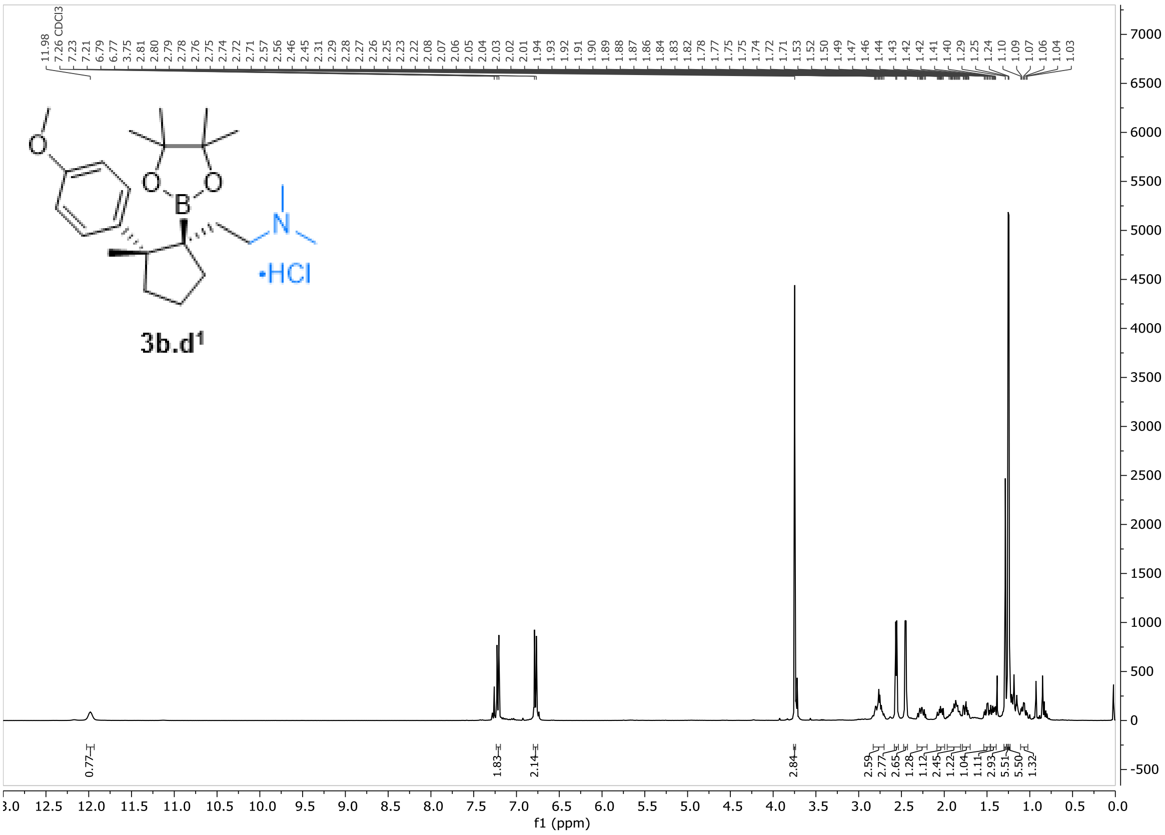

^{13}C NMR (101 MHz, $CDCl_3$) of **3b.d¹**

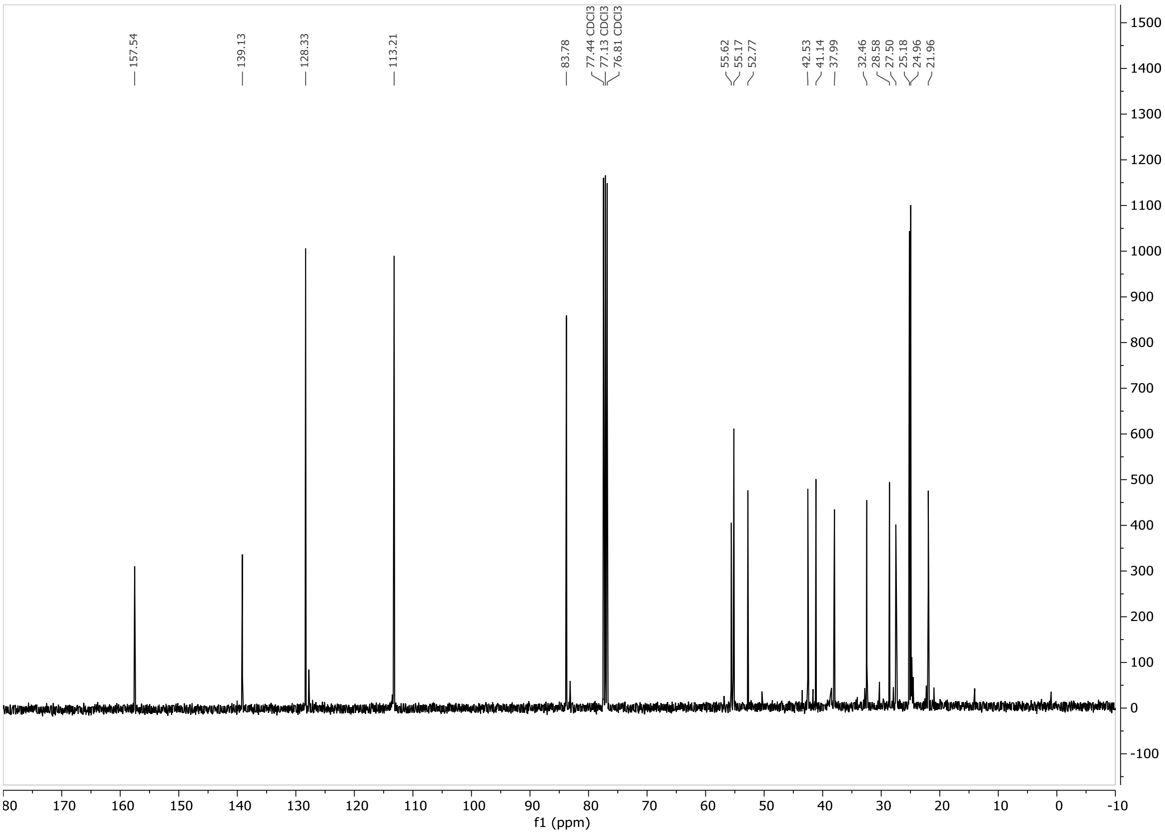

^{1}H NMR (400 MHz, $CDCl_3$) of **3b.d[2]** (*see procedure*)

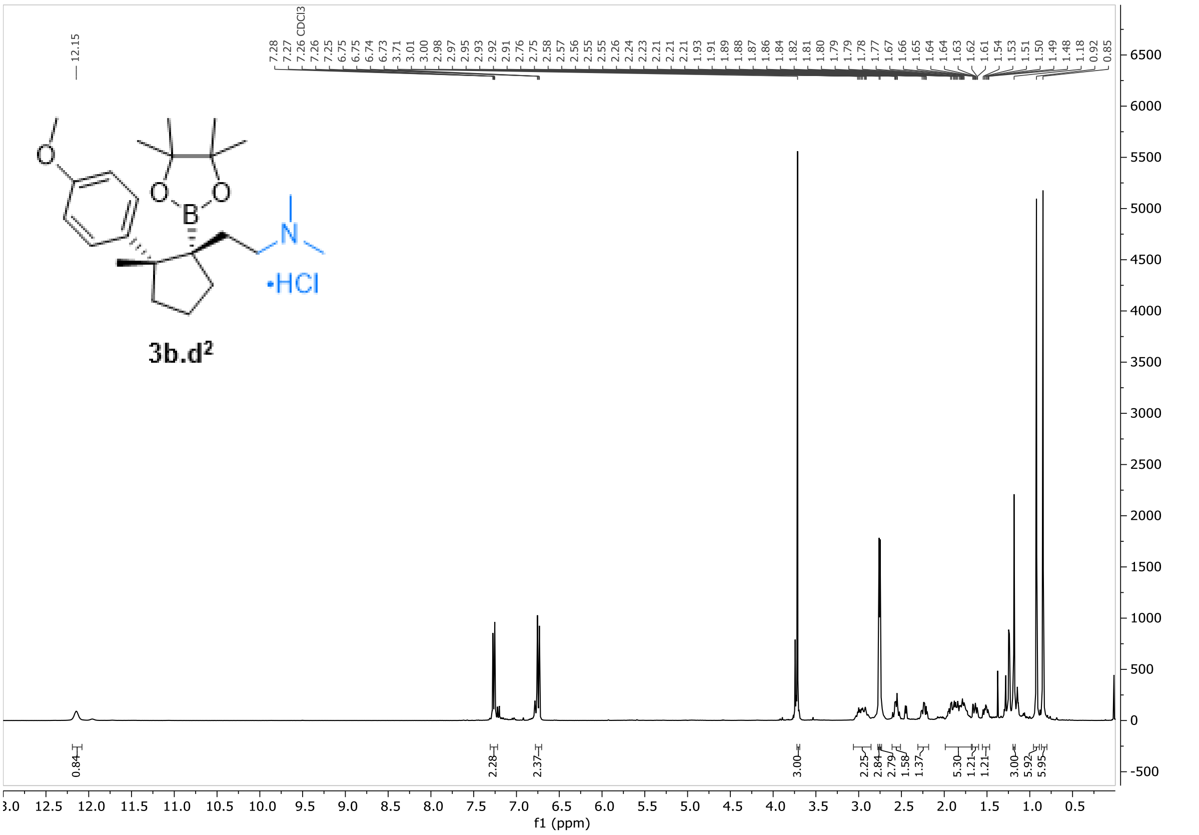

^{13}C NMR (101 MHz, $CDCl_3$) of **3b.d[2]**

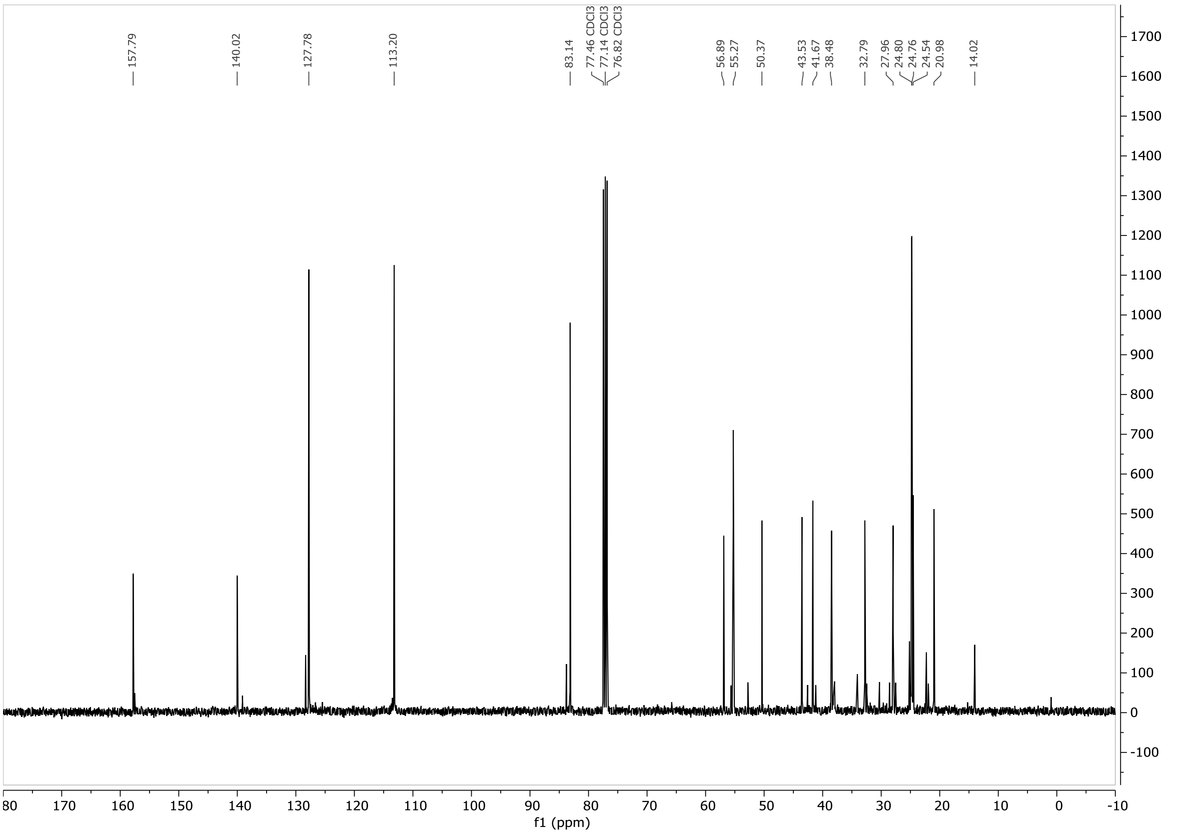

^{1}H NMR (400 MHz, $CDCl_3$) of **3c.d[1]** (*see procedure*)

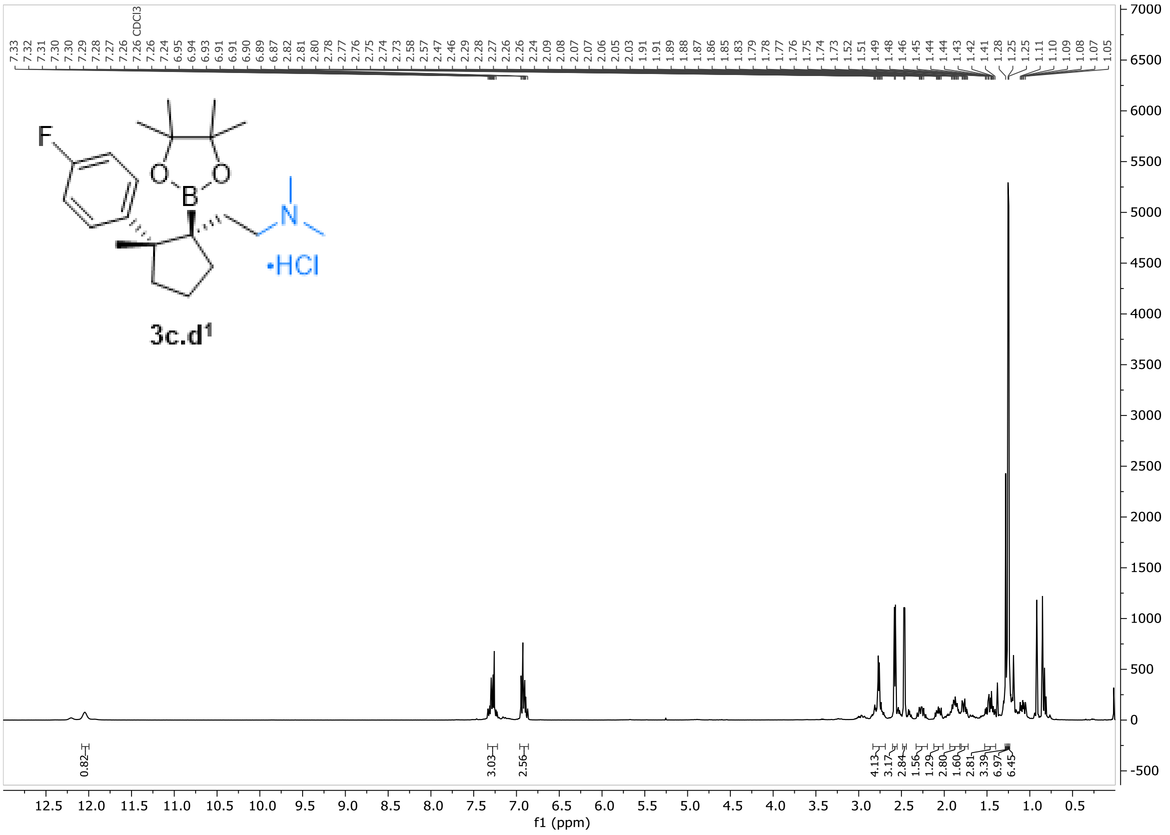

^{13}C NMR (101 MHz, $CDCl_3$) of **3c.d[1]**

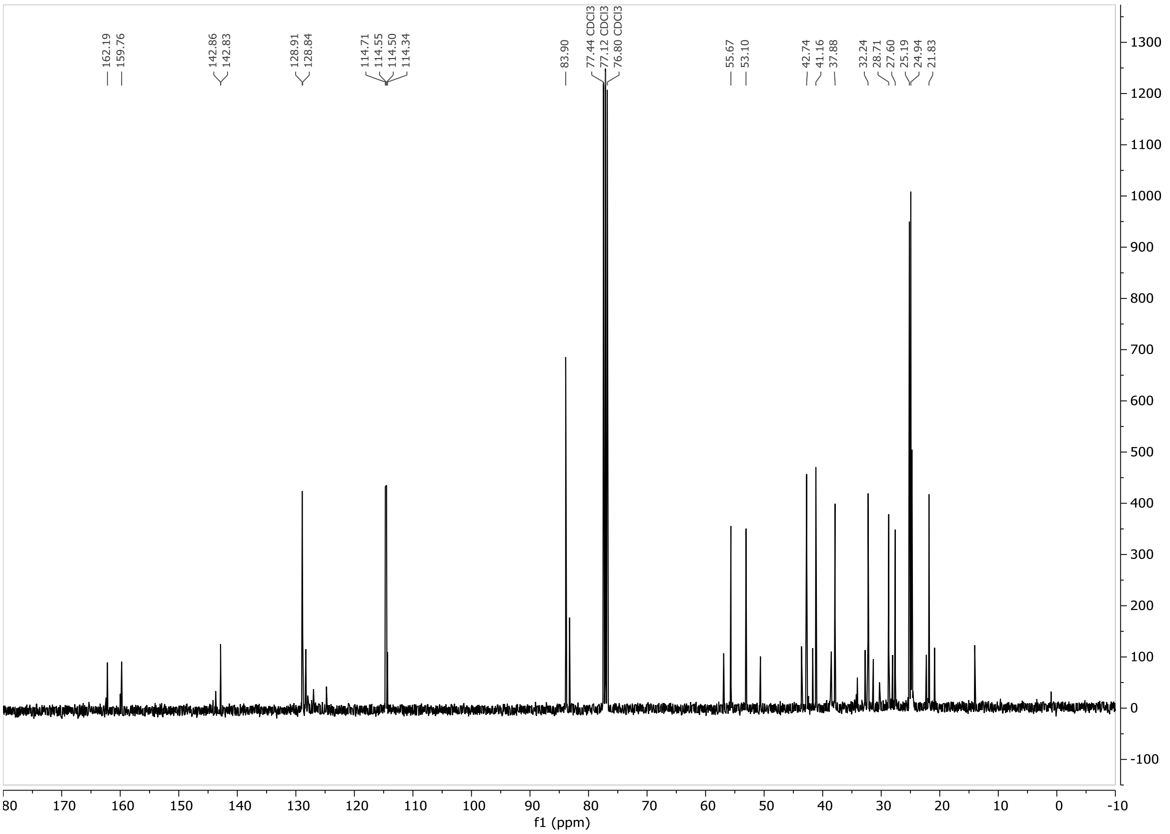

^{1}H NMR (400 MHz, $CDCl_3$) of **3c.d²** (*see procedure*)

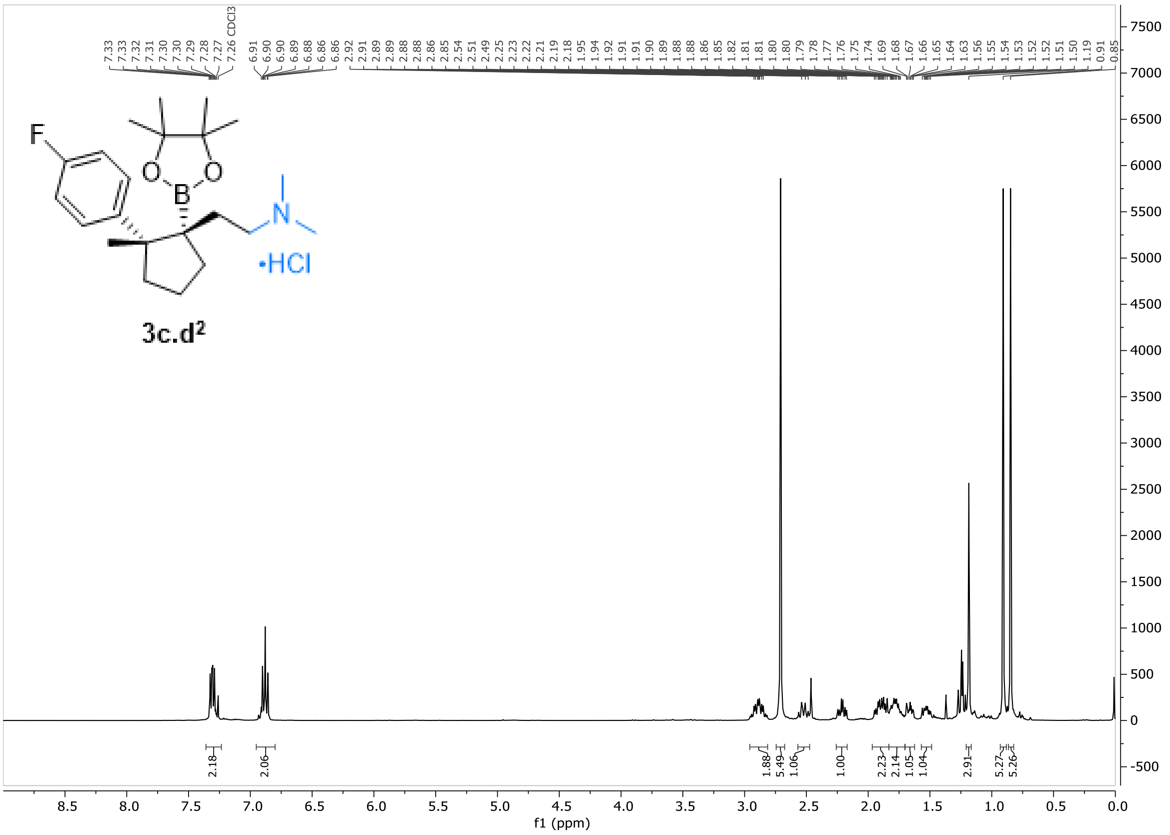

^{13}C NMR (101 MHz, $CDCl_3$) of **3c.d²**

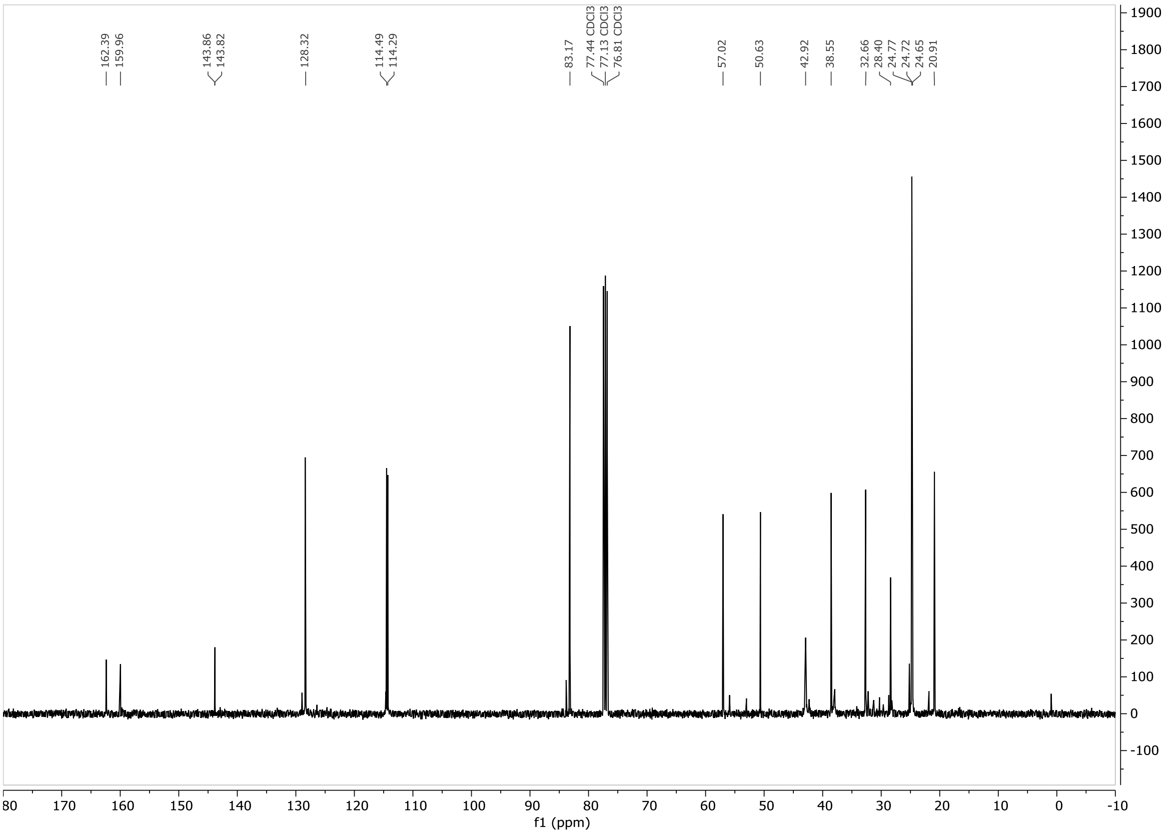

^{1}H NMR (400 MHz, $CDCl_3$) of **3d.d^{2}** *(see procedure)*

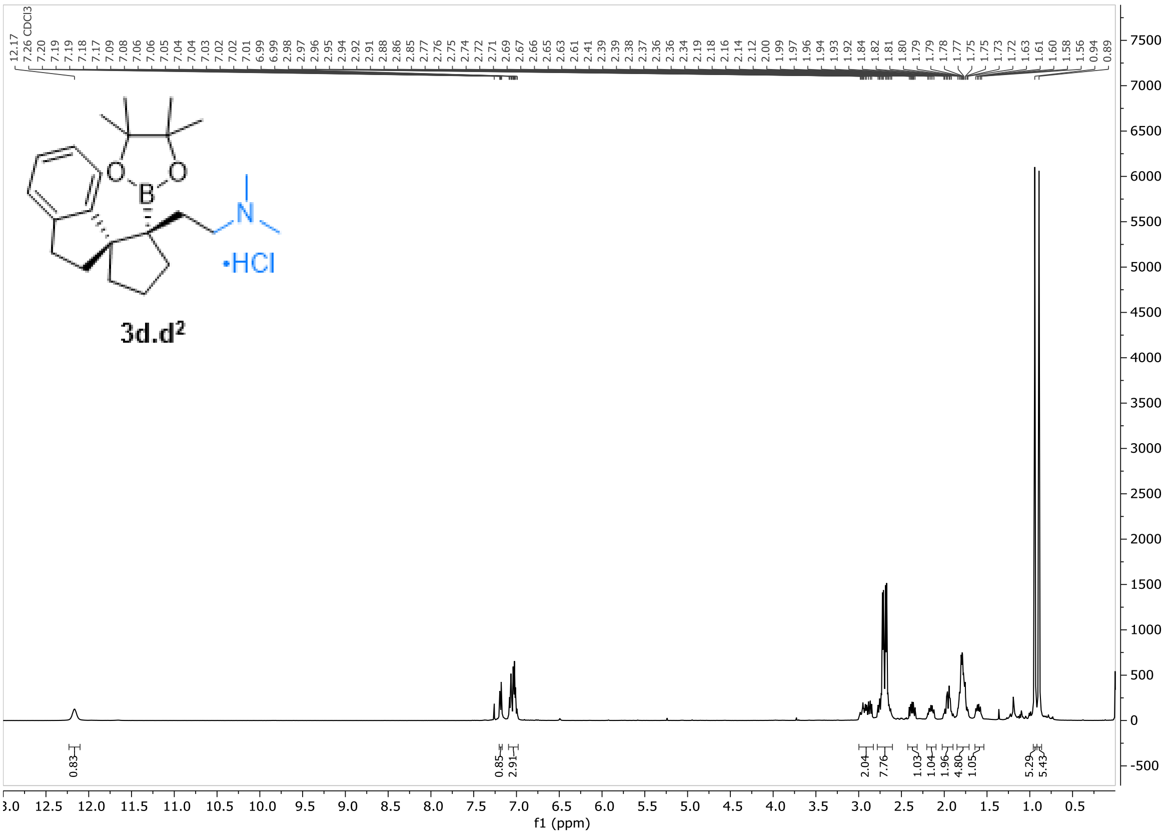

^{13}C NMR (101 MHz, $CDCl_3$) of **3d.d^{2}**

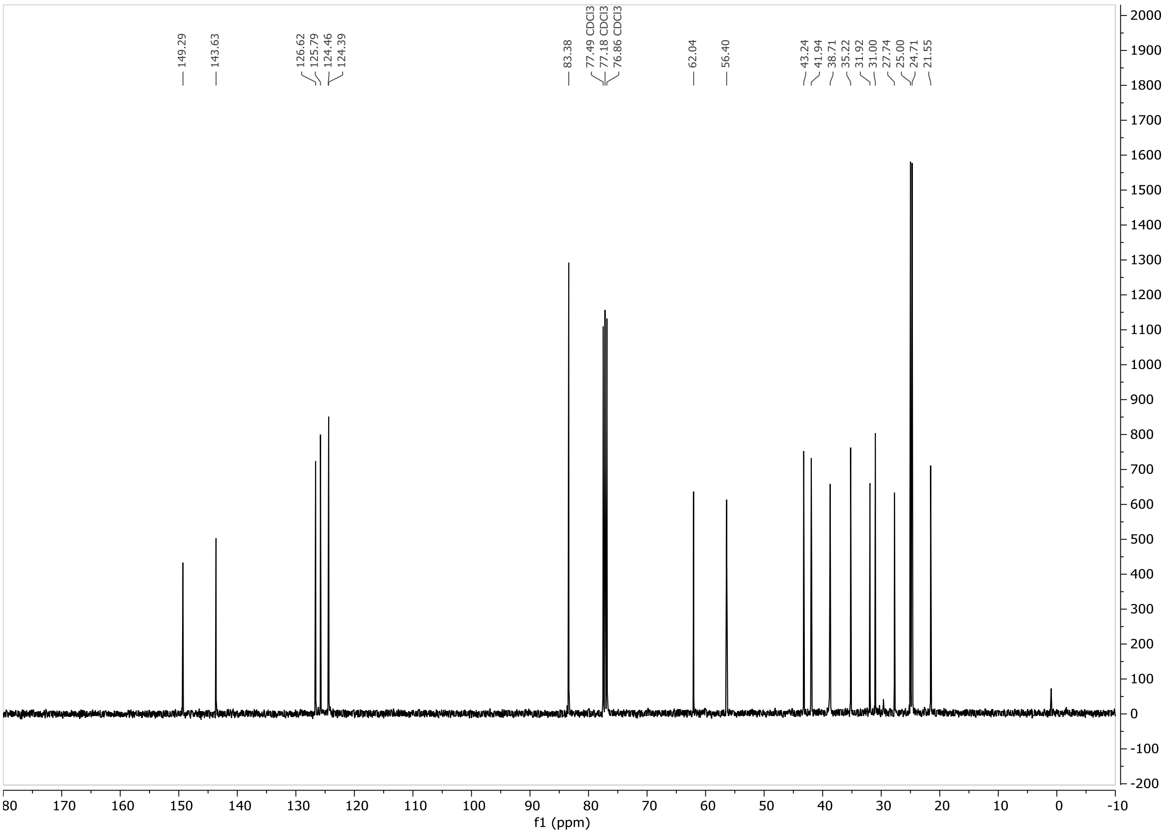

^{1}H NMR (400 MHz, $CDCl_3$) of **3e** *(see procedure)*

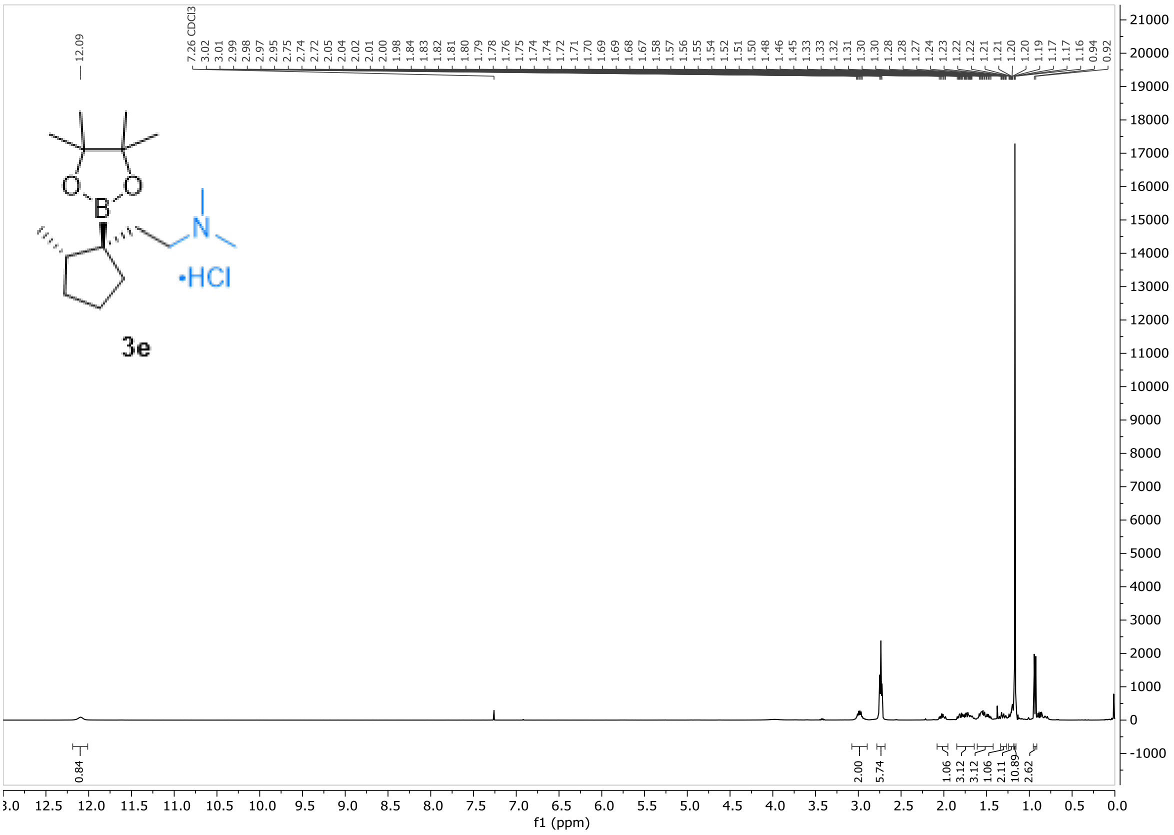

^{13}C NMR (101 MHz, $CDCl_3$) of **3e**

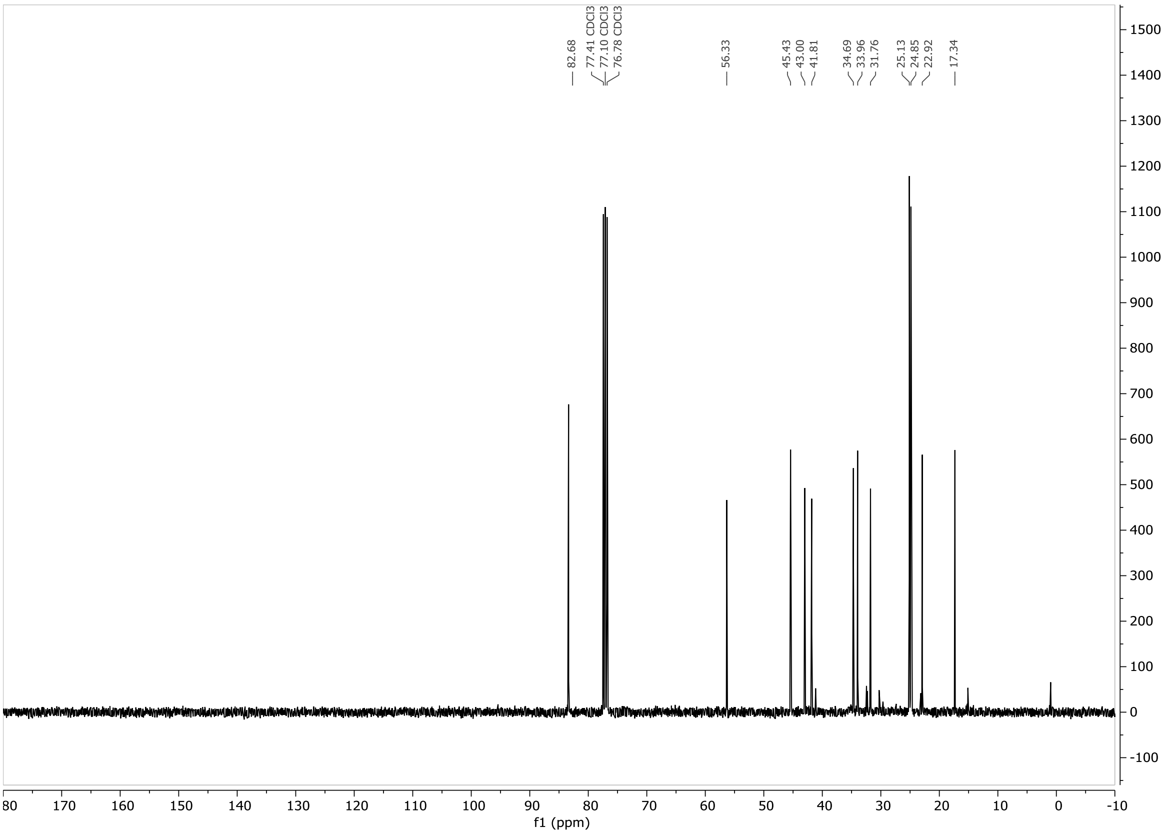

^{1}H NMR (400 MHz, $CDCl_3$) of **3f** *(see procedure)*

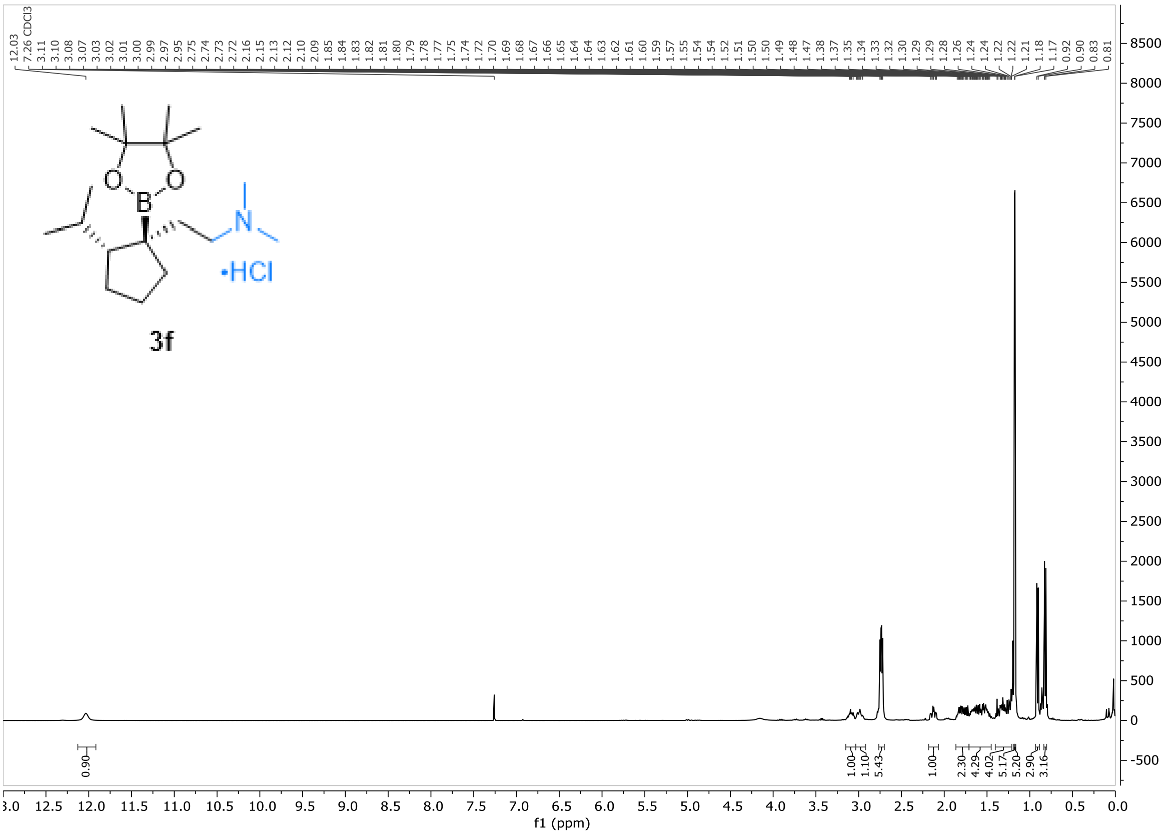

^{13}C NMR (101 MHz, $CDCl_3$) of **3f**

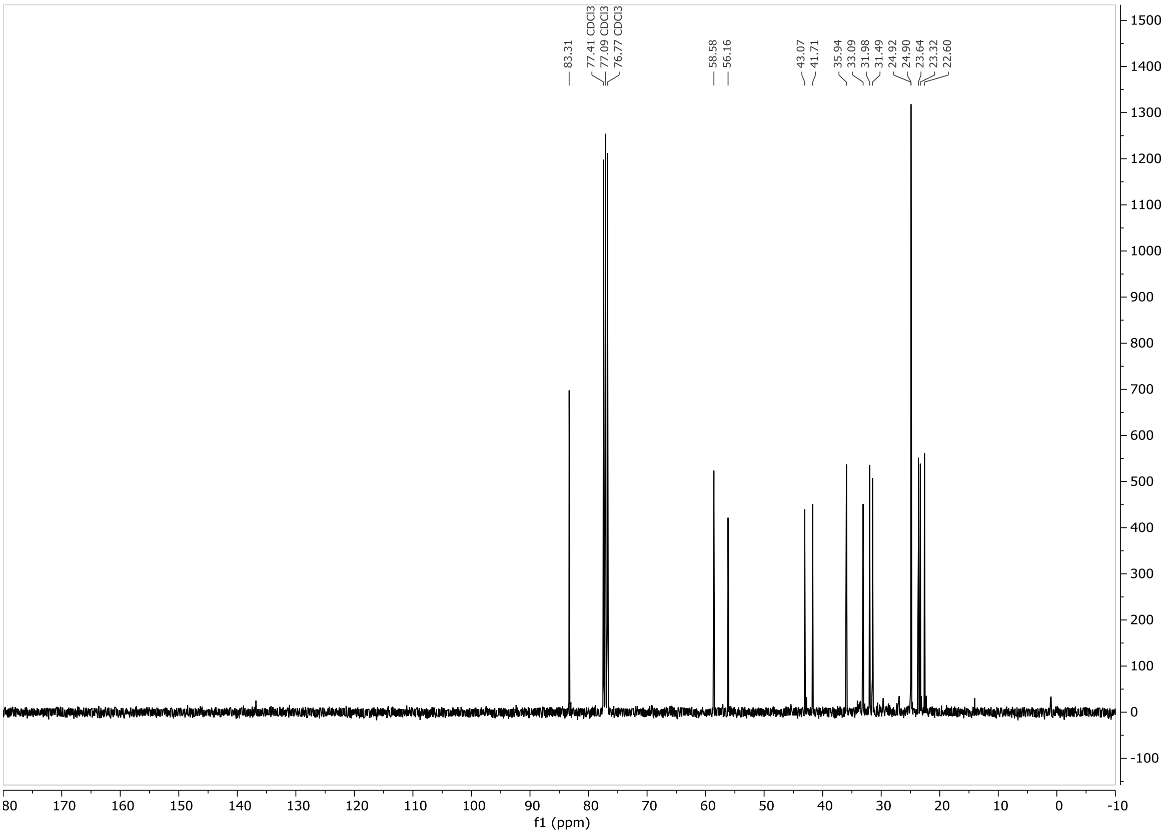

^{1}H NMR (400 MHz, $CDCl_3$) of **3g** *(see procedure)*

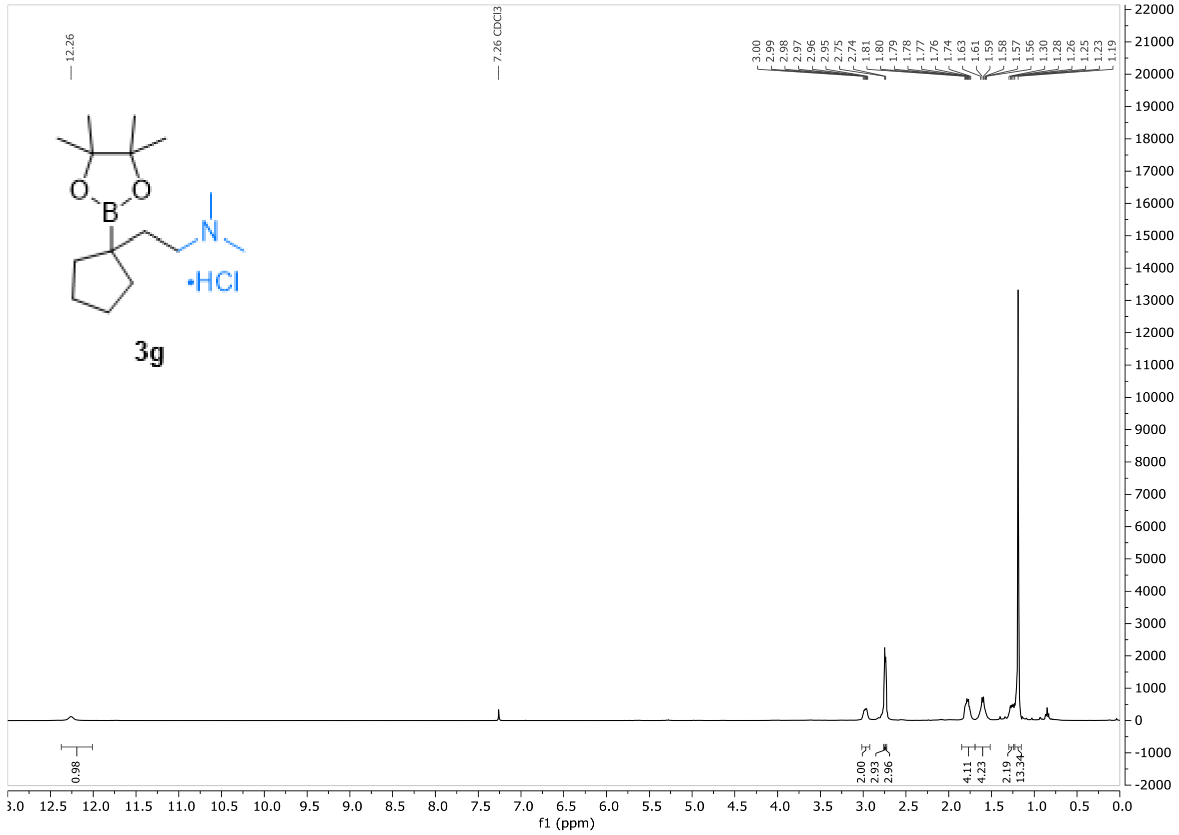

^{13}C NMR (101 MHz, $CDCl_3$) of **3g**

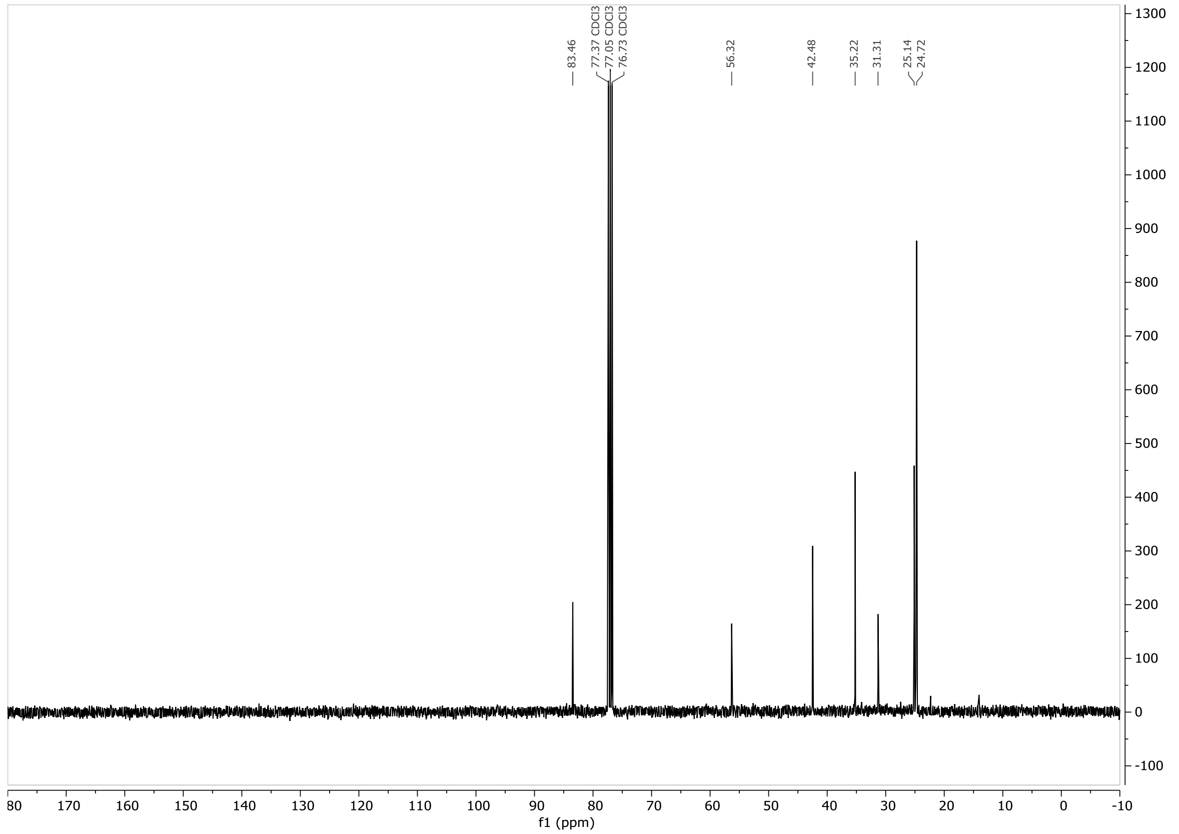

^{1}H NMR (400 MHz, $CDCl_3$) of **3h.d[1]** (*see procedure*)

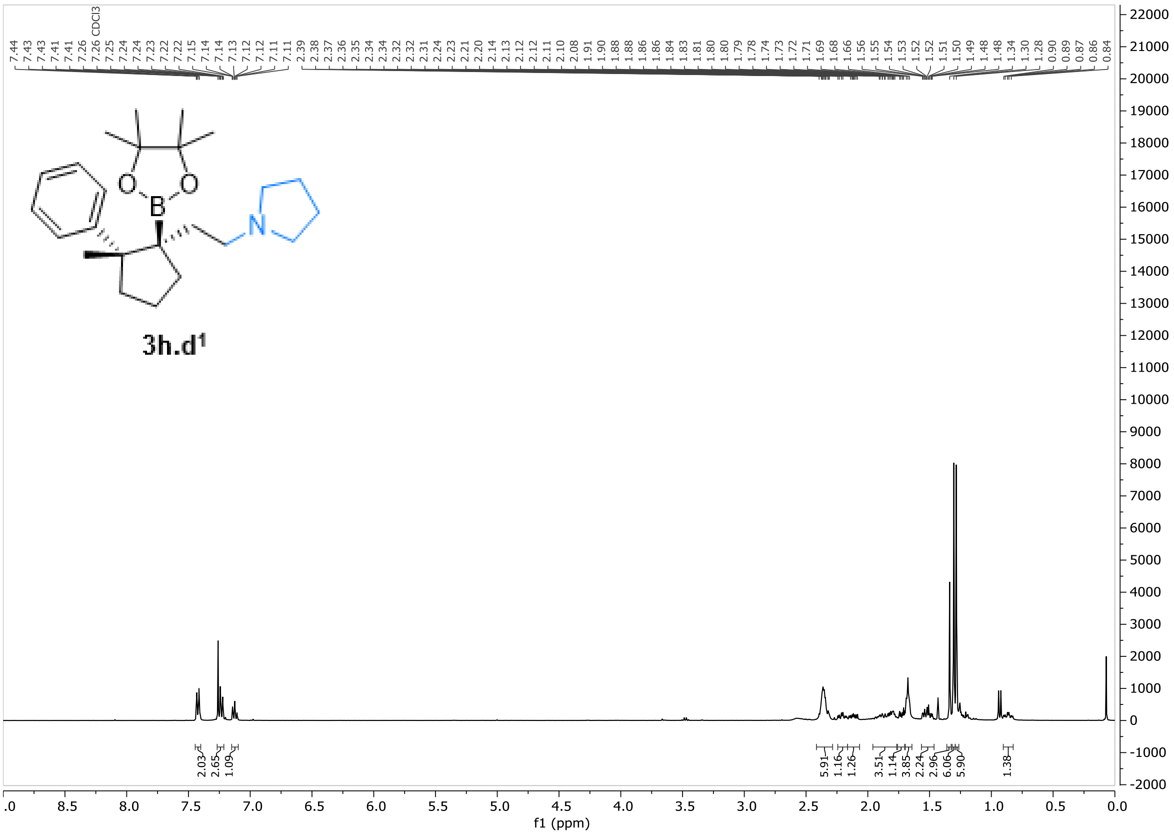

^{13}C NMR (101 MHz, $CDCl_3$) of **3h.d[1]**

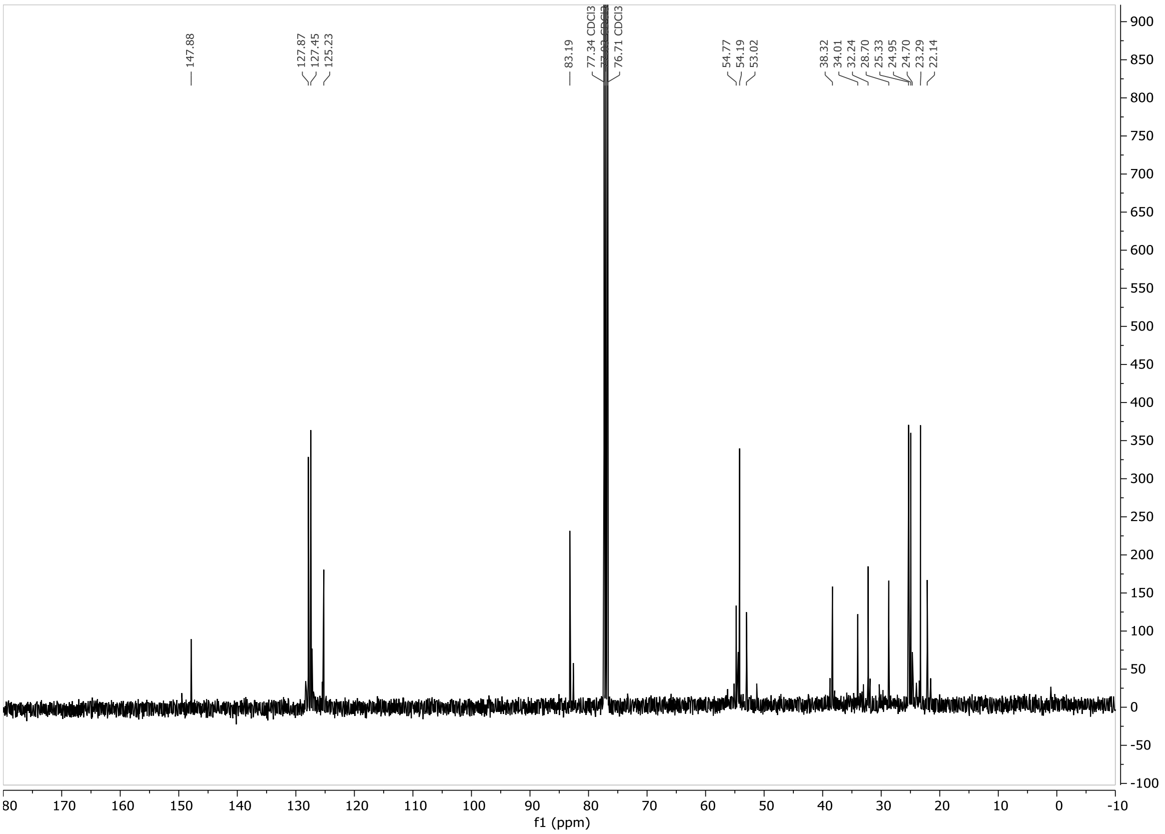

^{1}H NMR (400 MHz, $CDCl_3$) of **3h.d^2** (*see procedure*)

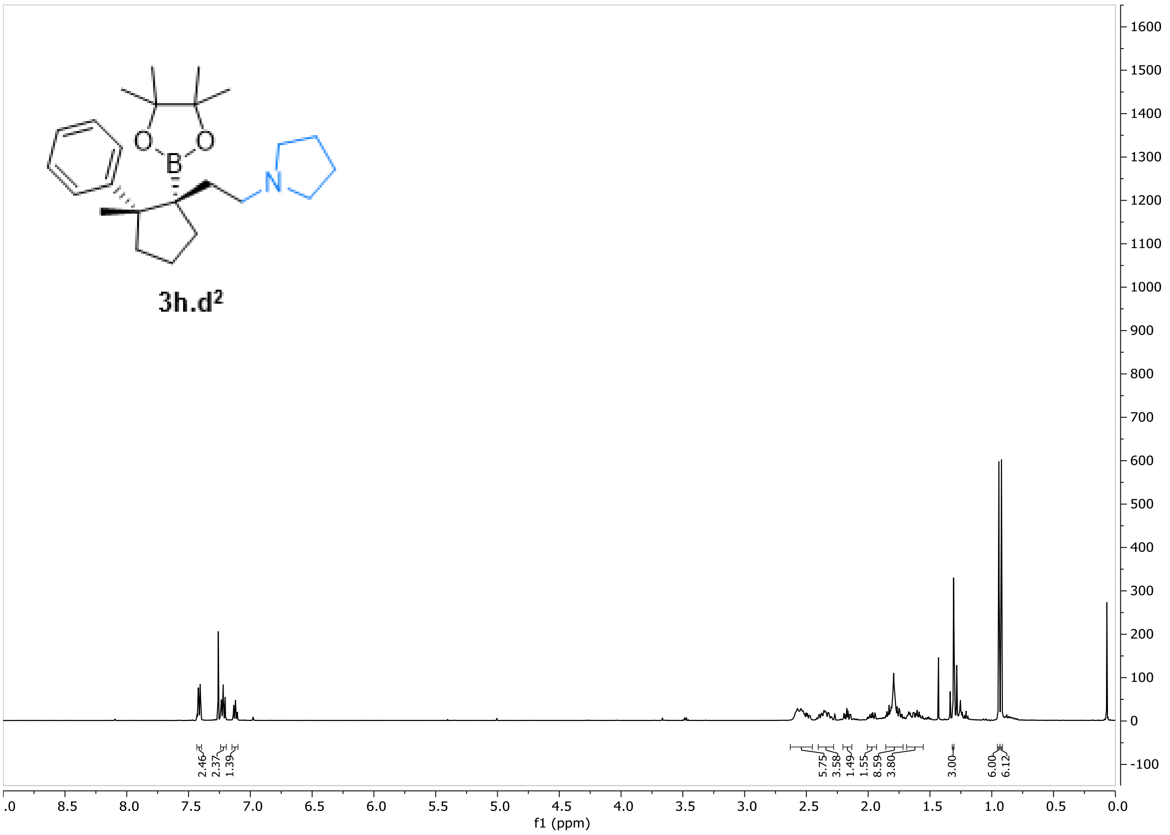

^{13}C NMR (101 MHz, $CDCl_3$) of **3h.d^2**

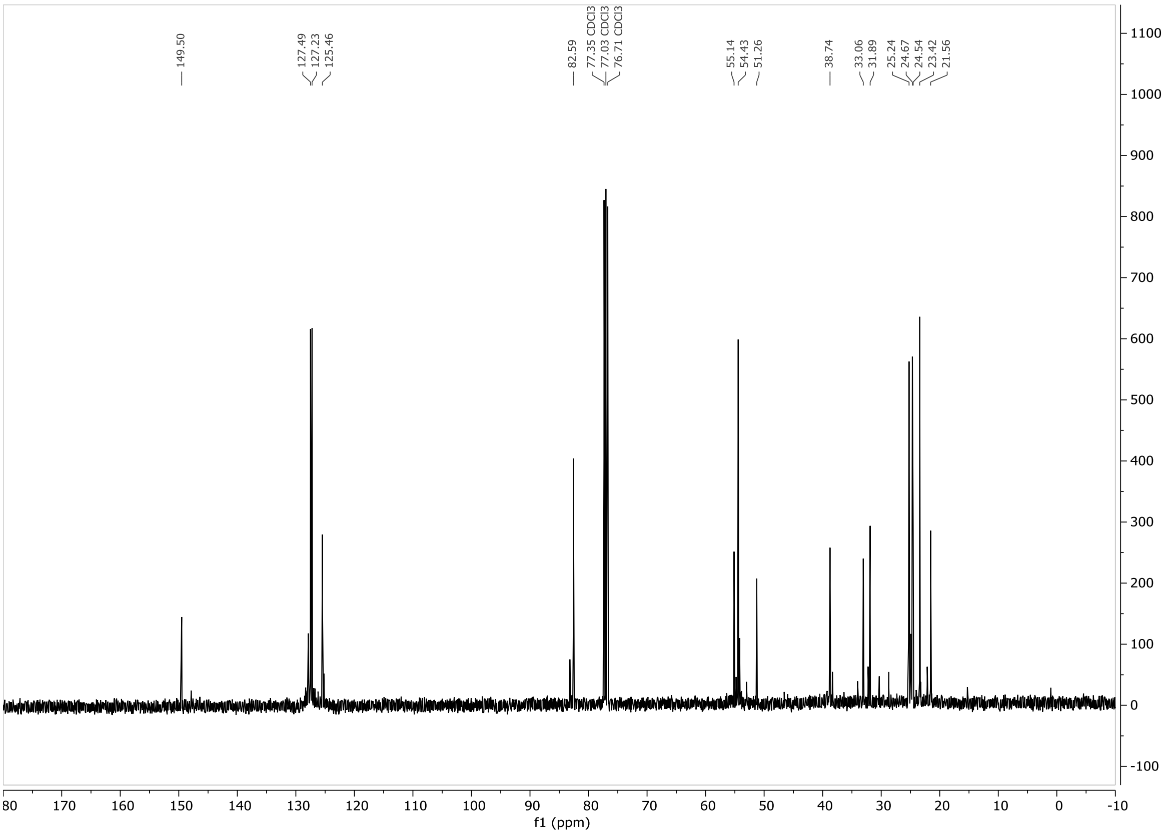

^{1}H NMR (400 MHz, $CDCl_3$) of **3i.d^{1}** *(see procedure)*

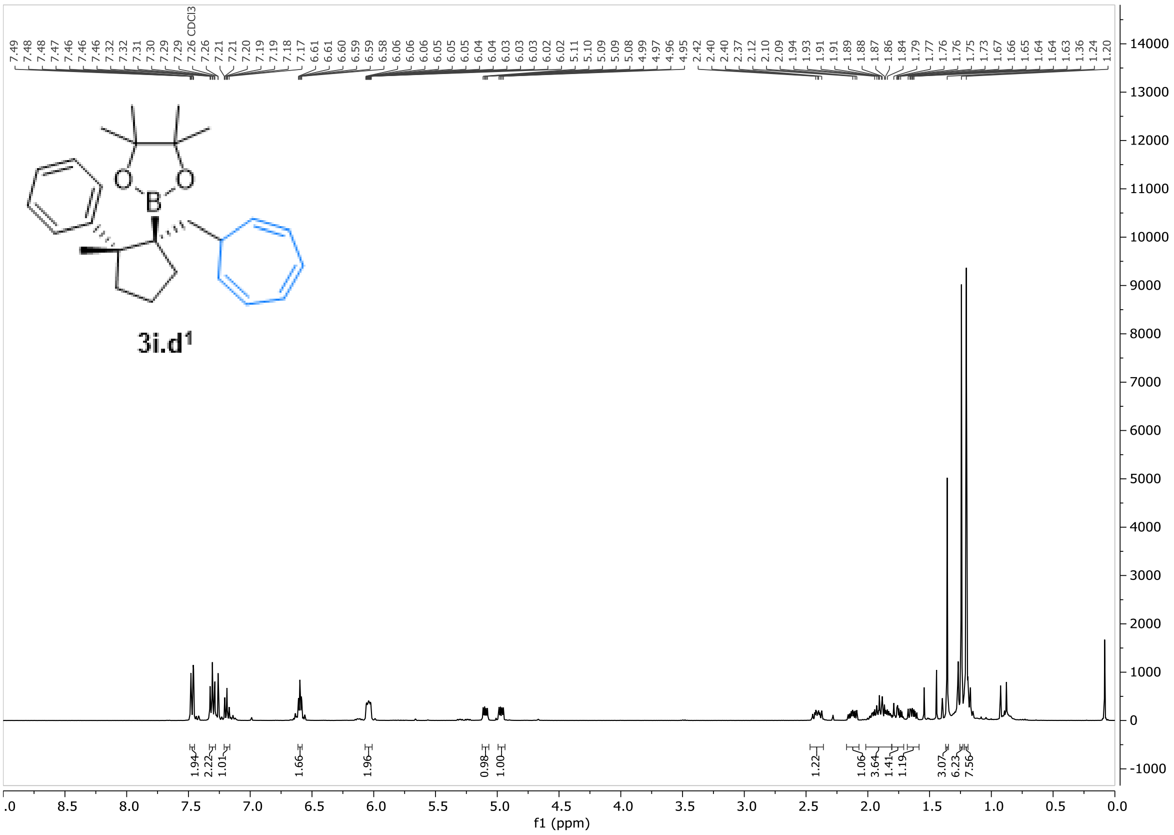

^{13}C NMR (101 MHz, $CDCl_3$) of **3i.d^{1}**

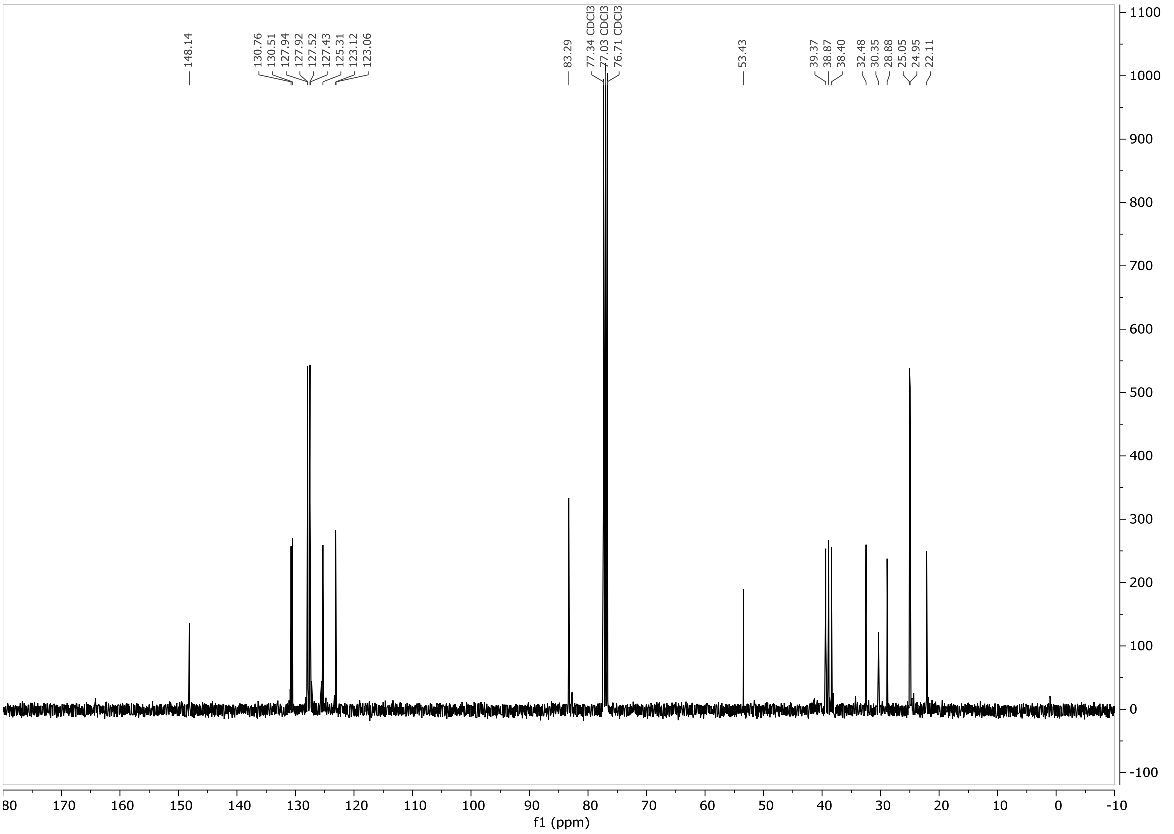

^{1}H NMR (400 MHz, $CDCl_3$) of **3i'.d[1]** (*see procedure*)

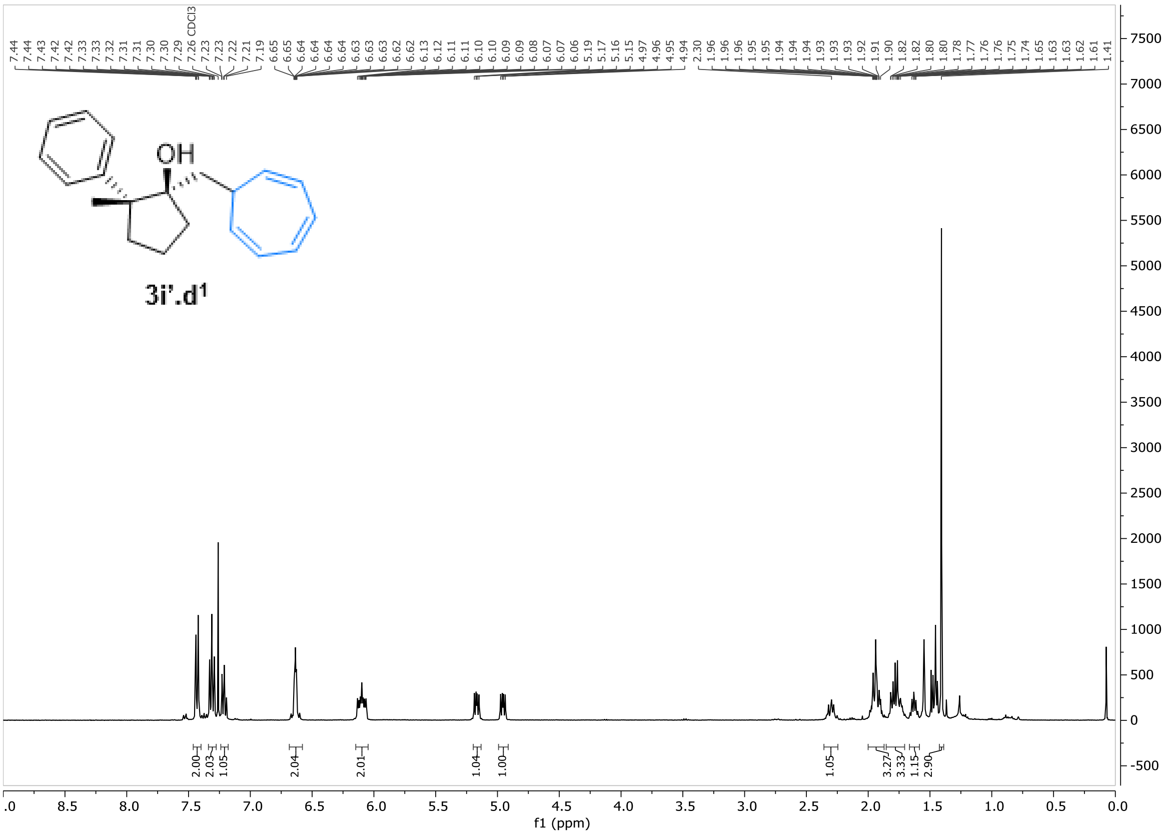

^{13}C NMR (101 MHz, $CDCl_3$) of **3i'.d[1]**

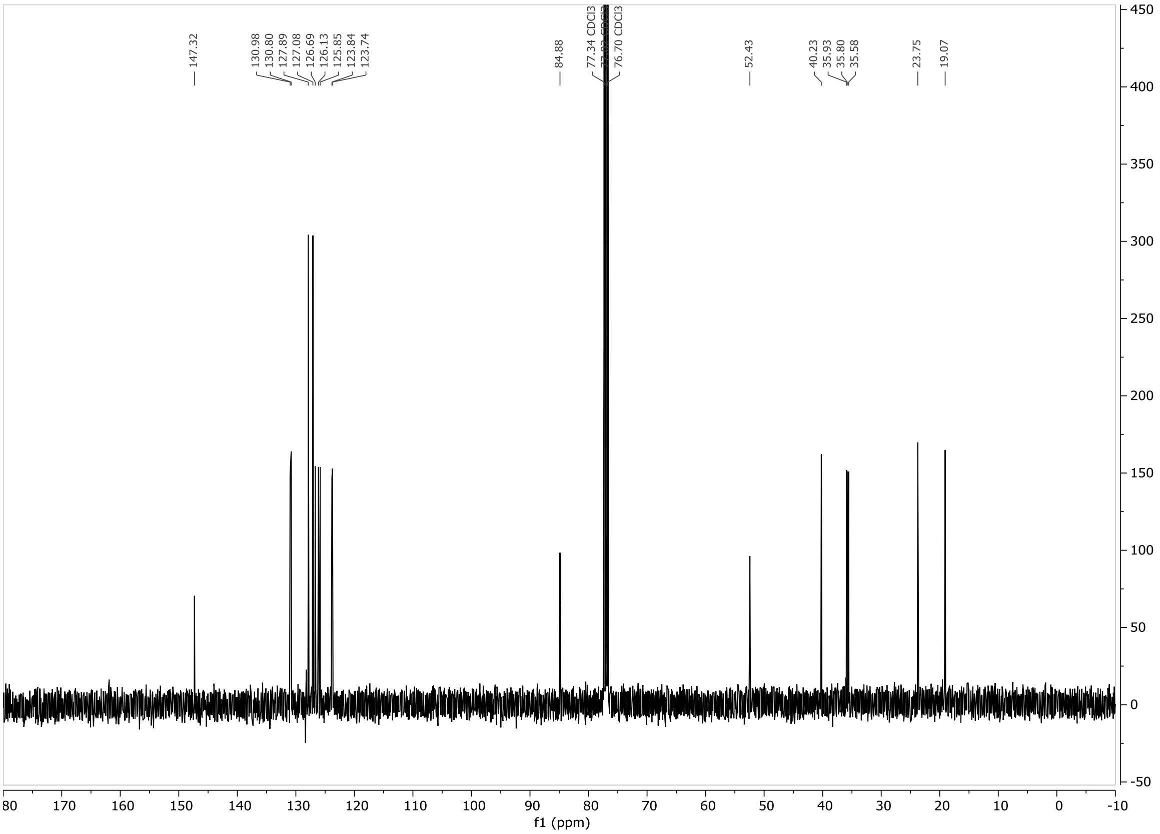

^{1}H NMR (400 MHz, $CDCl_3$) of **5** *(see procedure)*

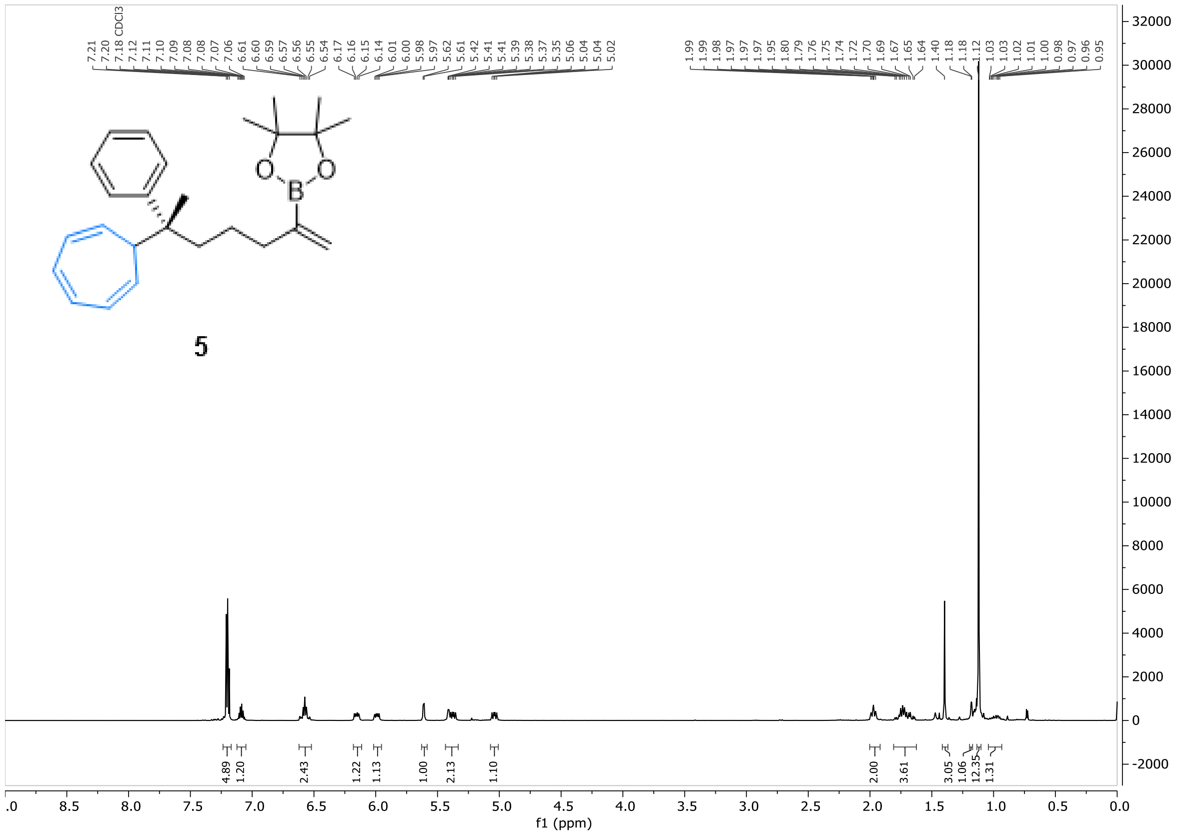

^{13}C NMR (101 MHz, $CDCl_3$) of **5**

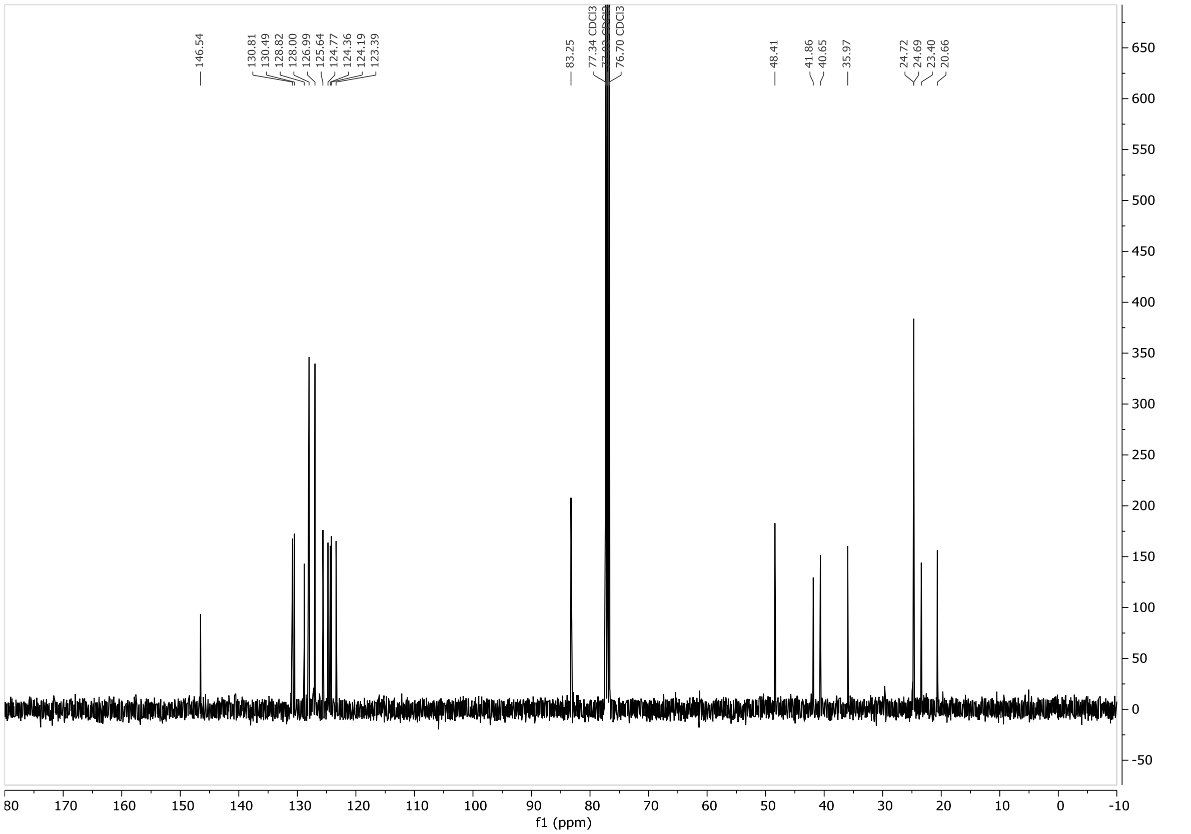

^{1}H NMR (400 MHz, $CDCl_3$) of **5’** *(see procedure)*

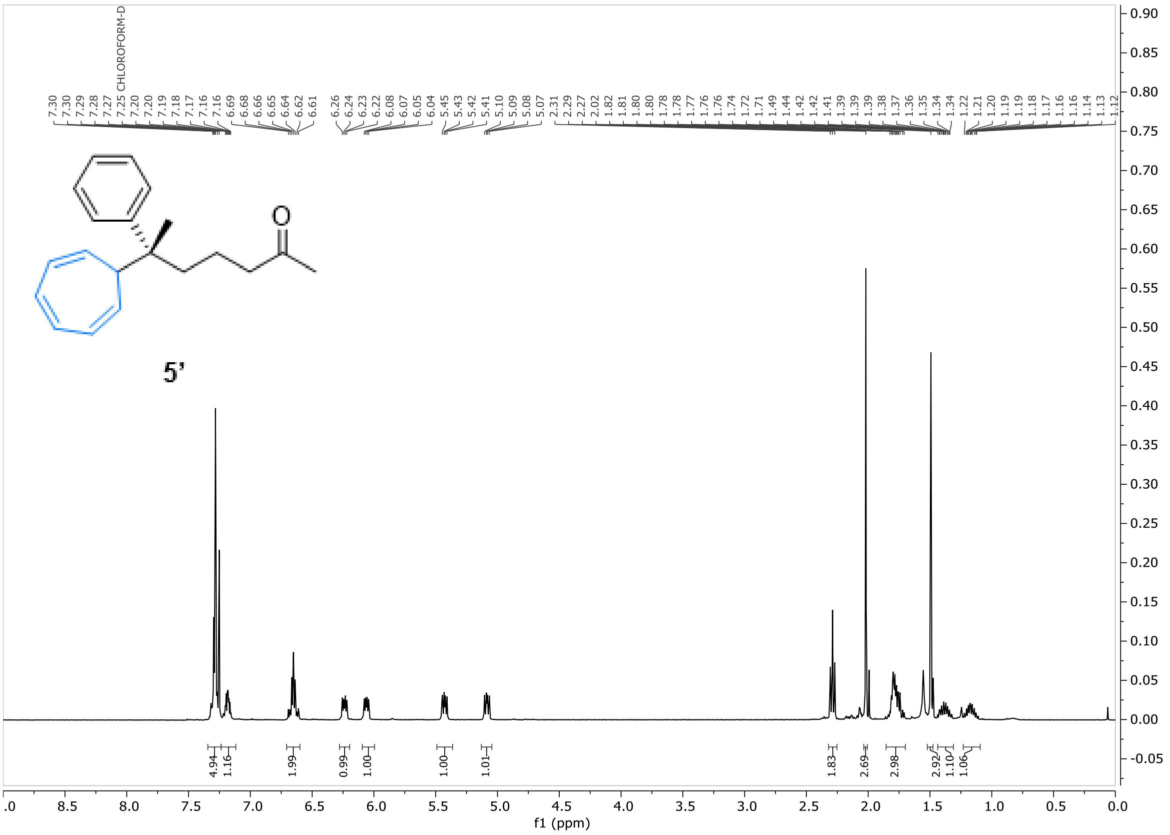

^{13}C NMR (101 MHz, $CDCl_3$) of **5’**

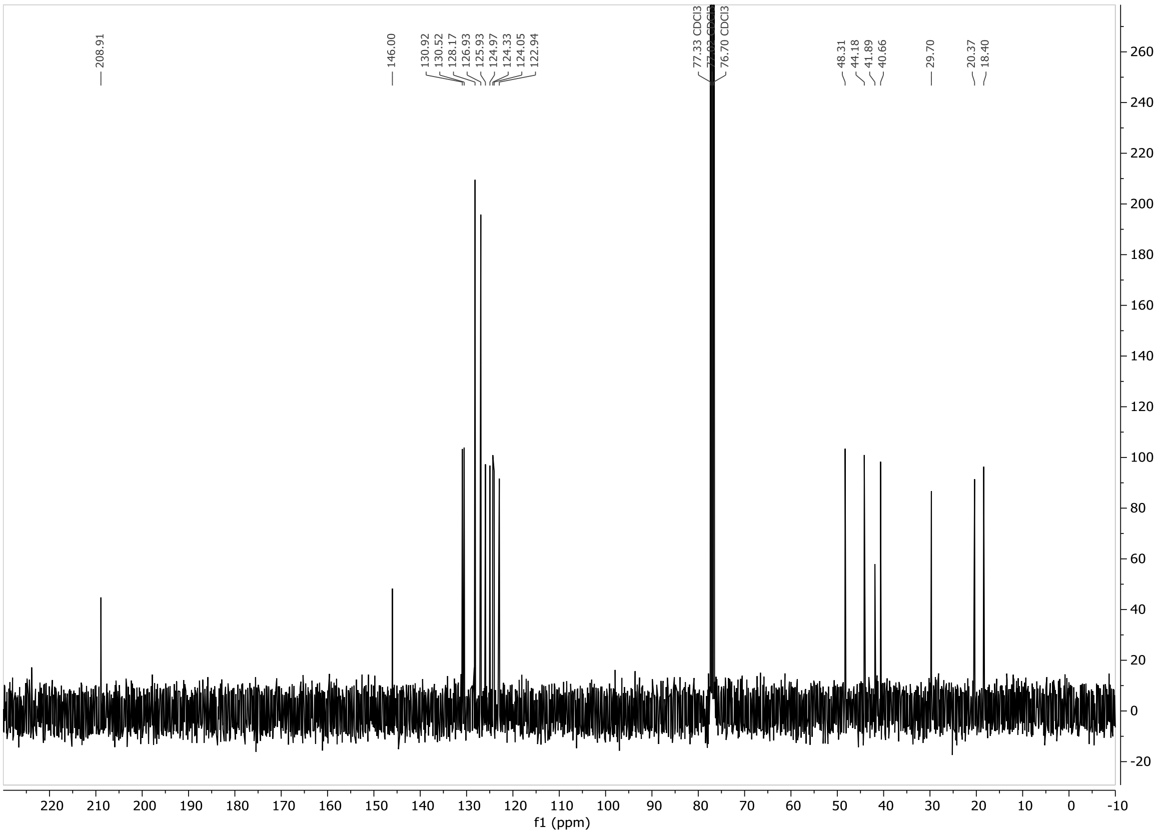

^{1}H NMR (400 MHz, $CDCl_3$) of **3i.d²** *(see procedure)*

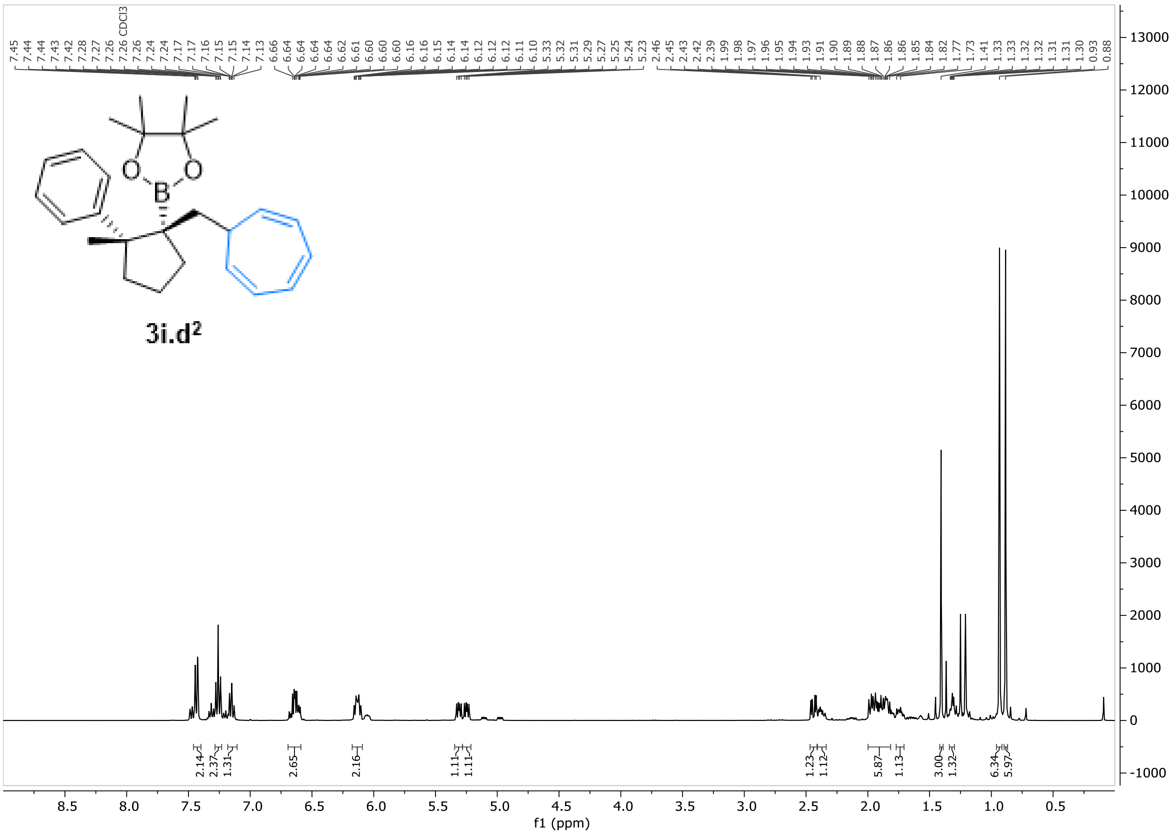

^{13}C NMR (101 MHz, $CDCl_3$) of **3i.d²**

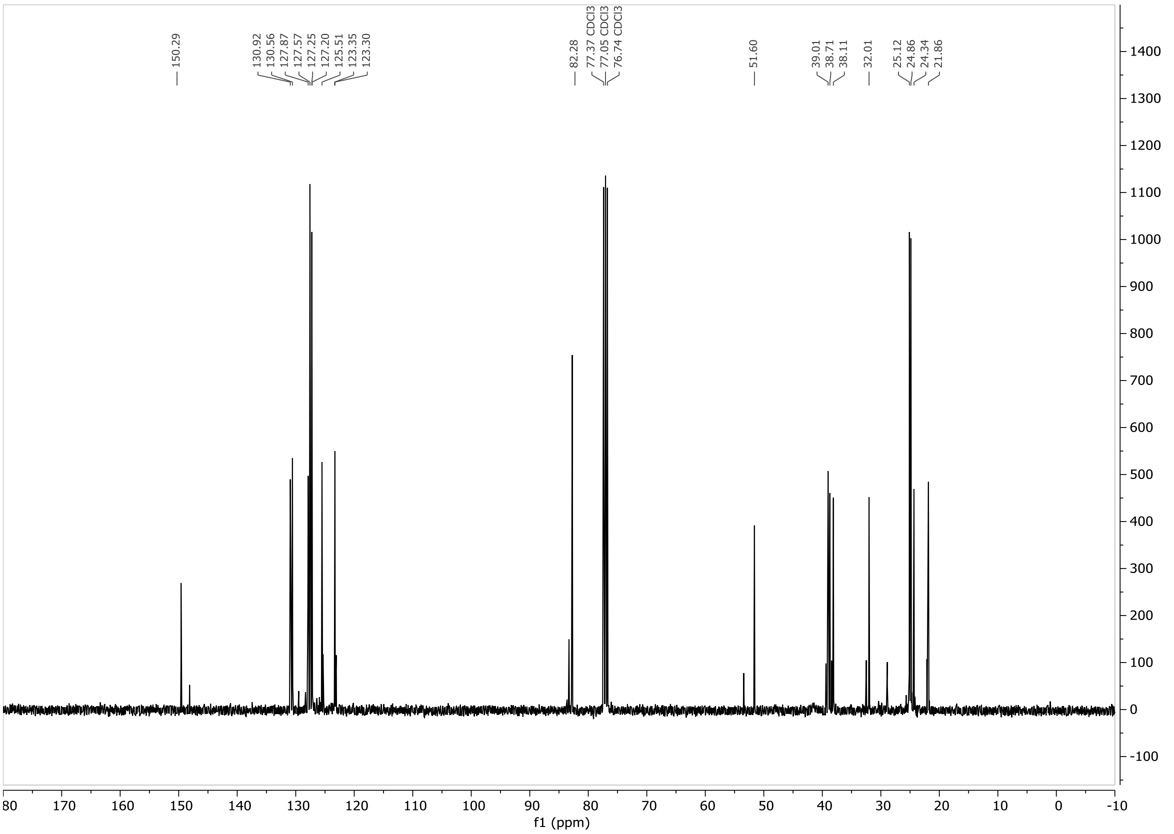

^{1}H NMR (400 MHz, $CDCl_3$) of **3i'.d^2** *(see procedure)*

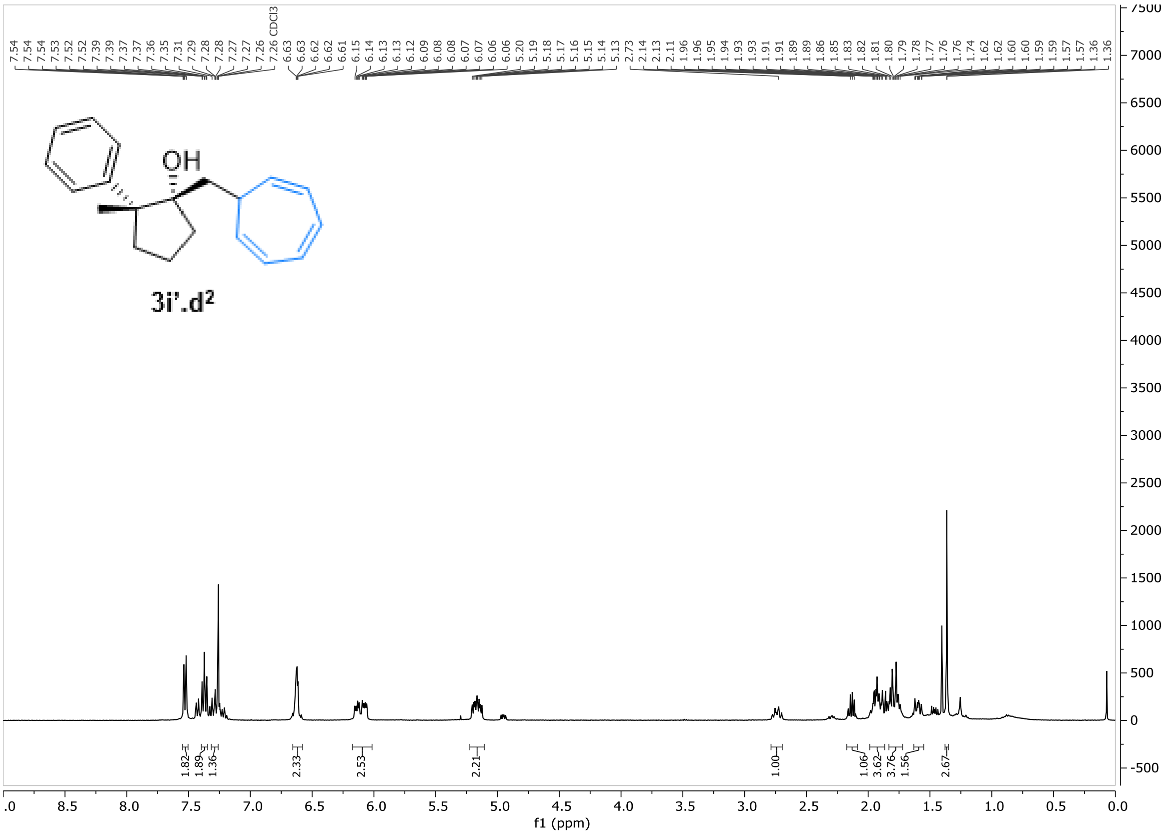

^{13}C NMR (101 MHz, $CDCl_3$) of **3i'.d^2**

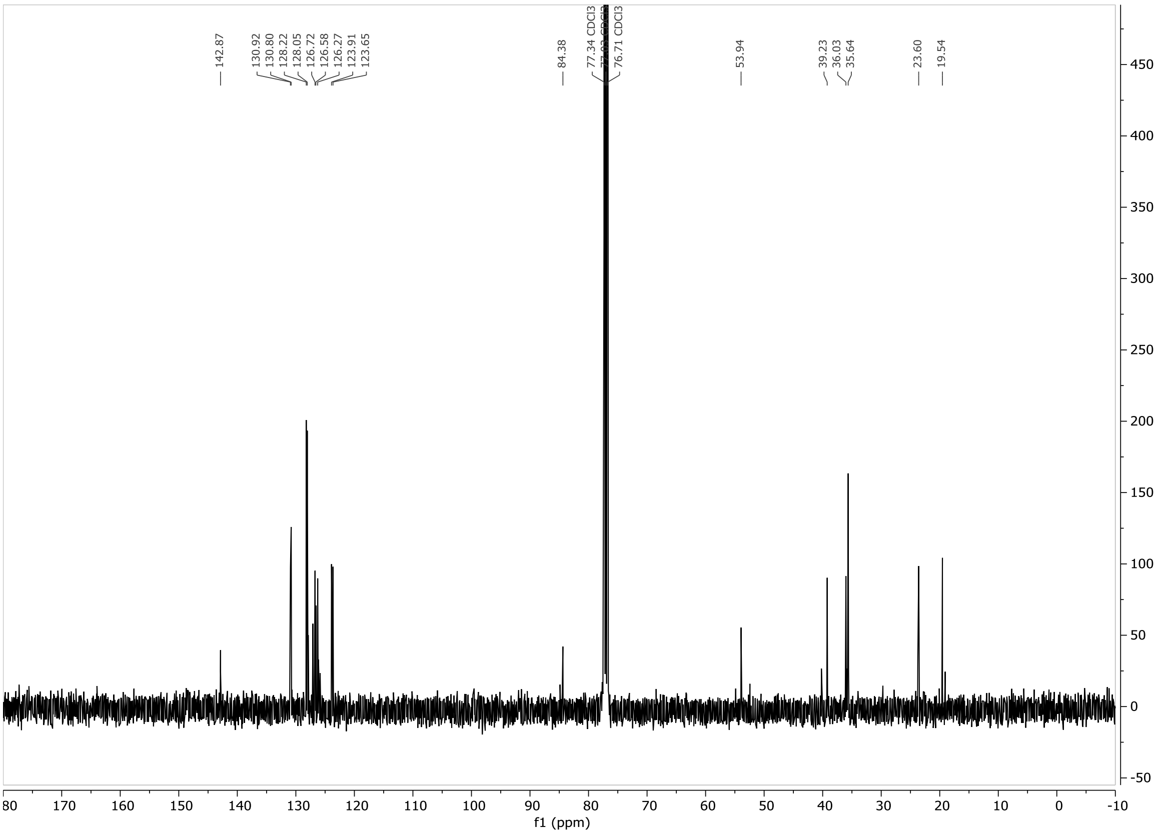

^{1}H NMR (400 MHz, $CDCl_3$) of **3j.d[1]** *(see procedure)*

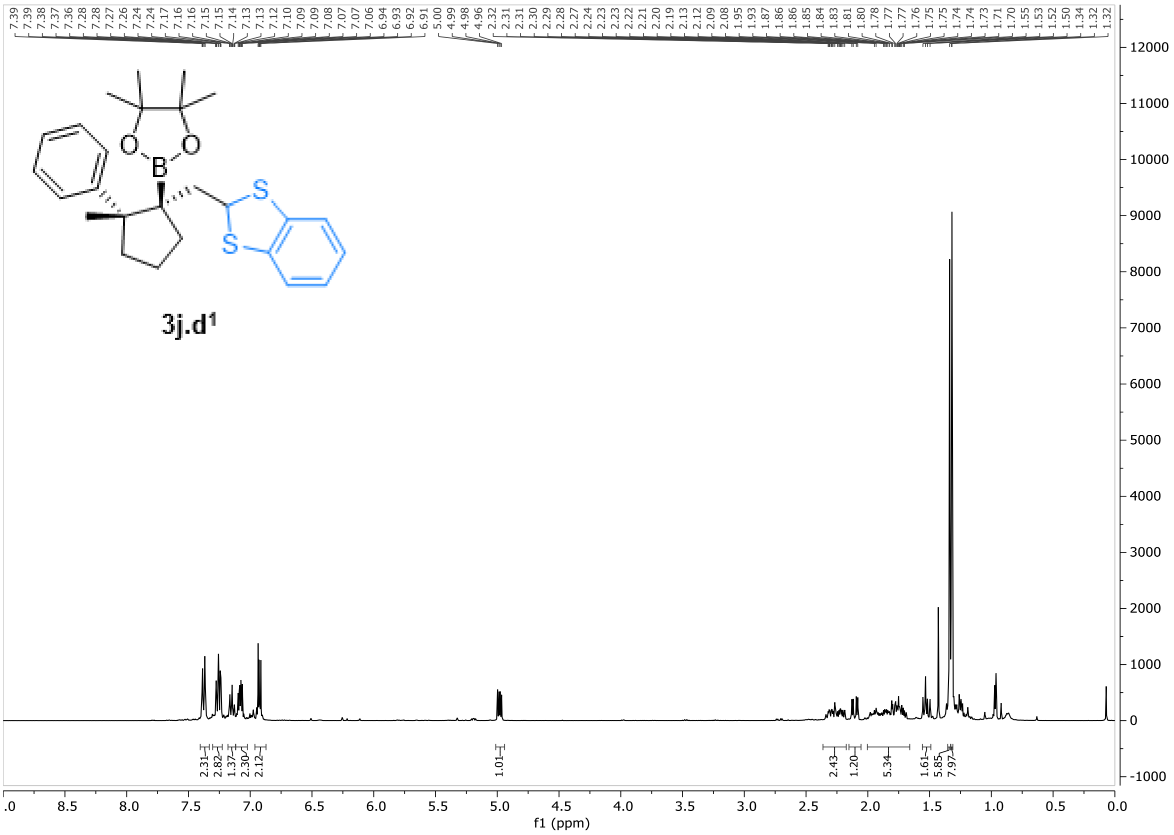

3j.d[1]

^{13}C NMR (101 MHz, $CDCl_3$) of **3j.d[1]**

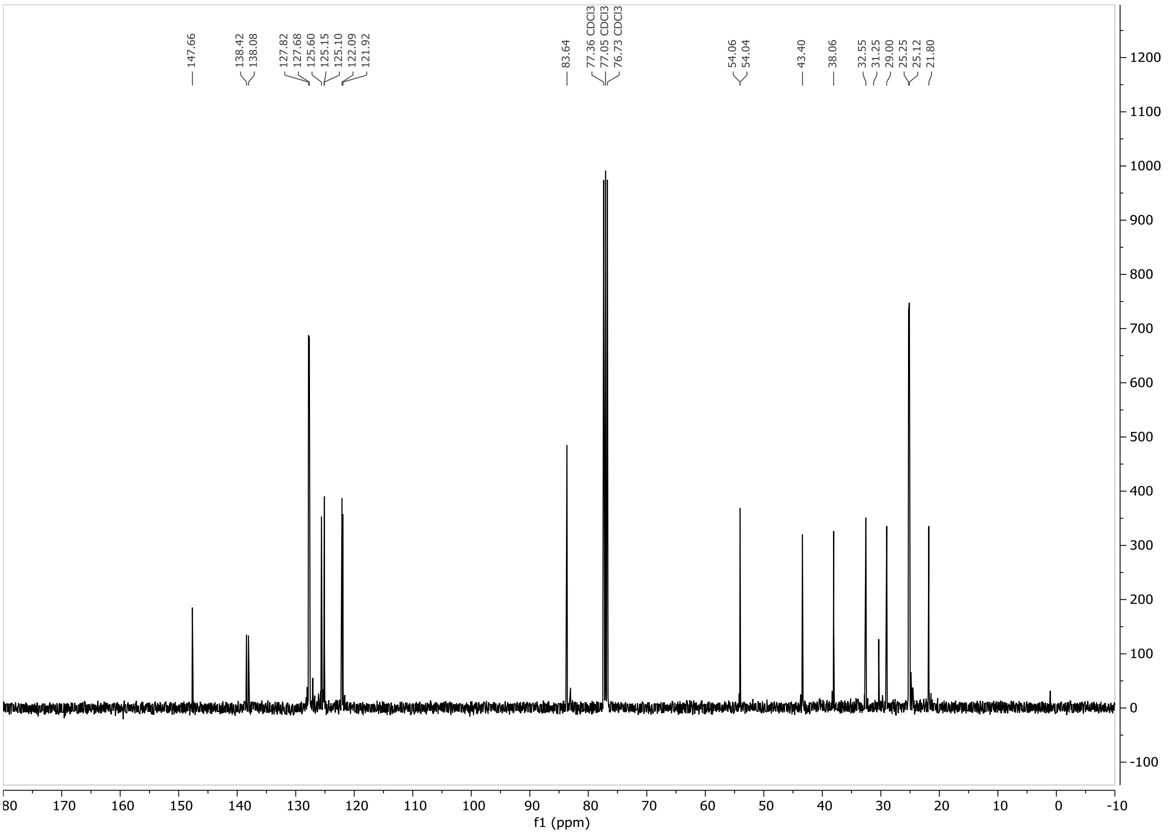

^{1}H NMR (500 MHz, $CDCl_3$) of **3j'.d[1]** *(see procedure)*

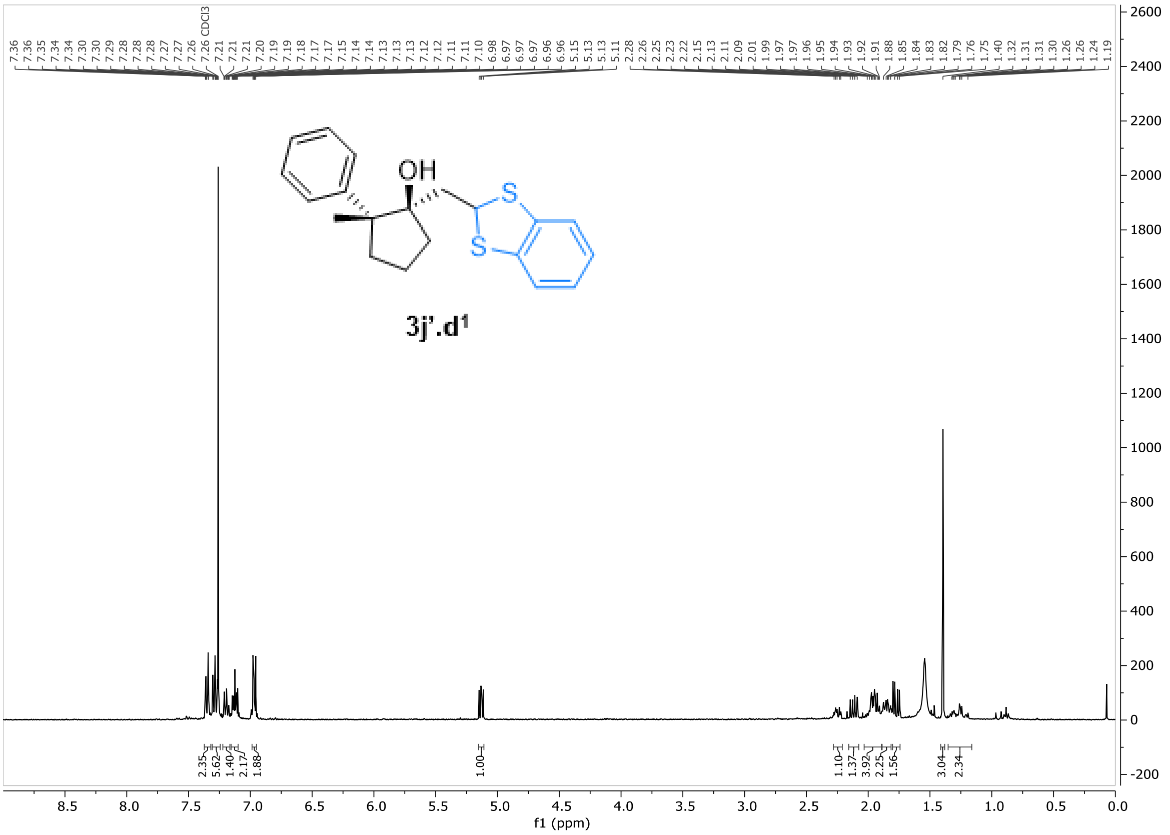

^{13}C NMR (126 MHz, $CDCl_3$) of **3j'.d[1]**

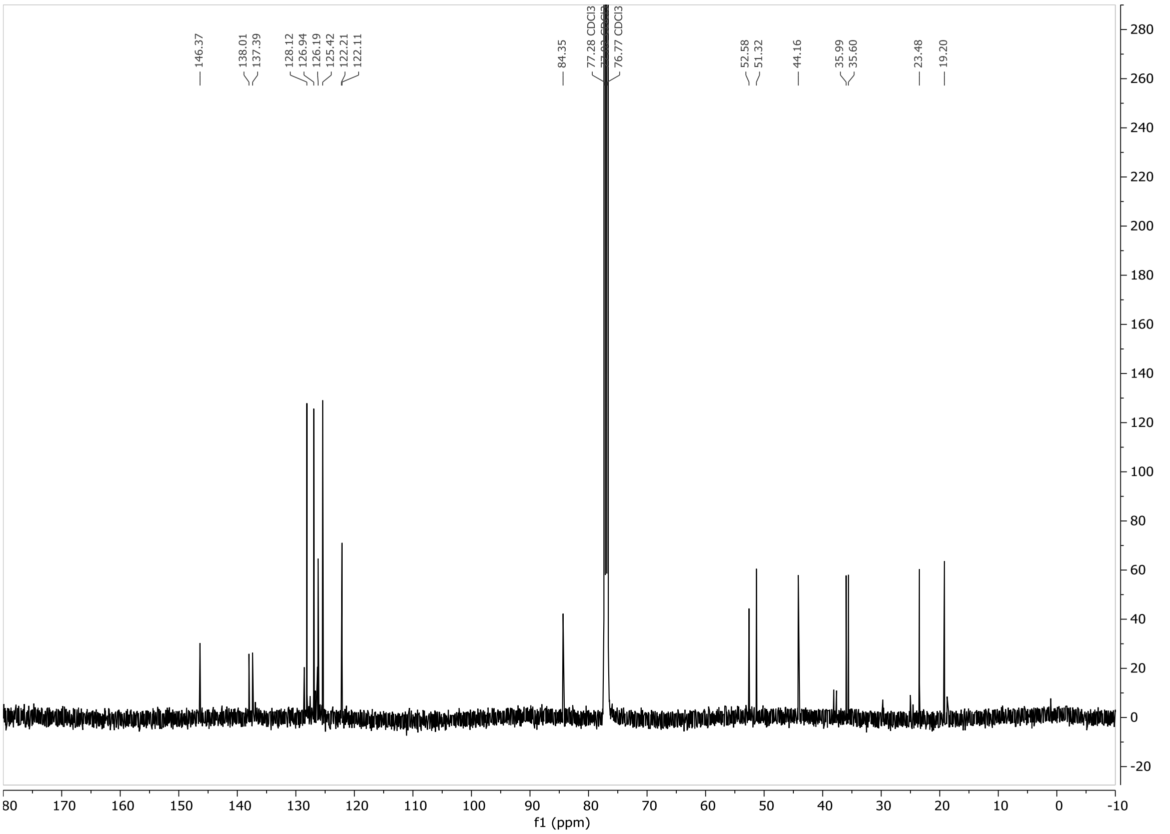

^{1}H NMR (400 MHz, $CDCl_3$) of **3k.d^2** (*see procedure*)

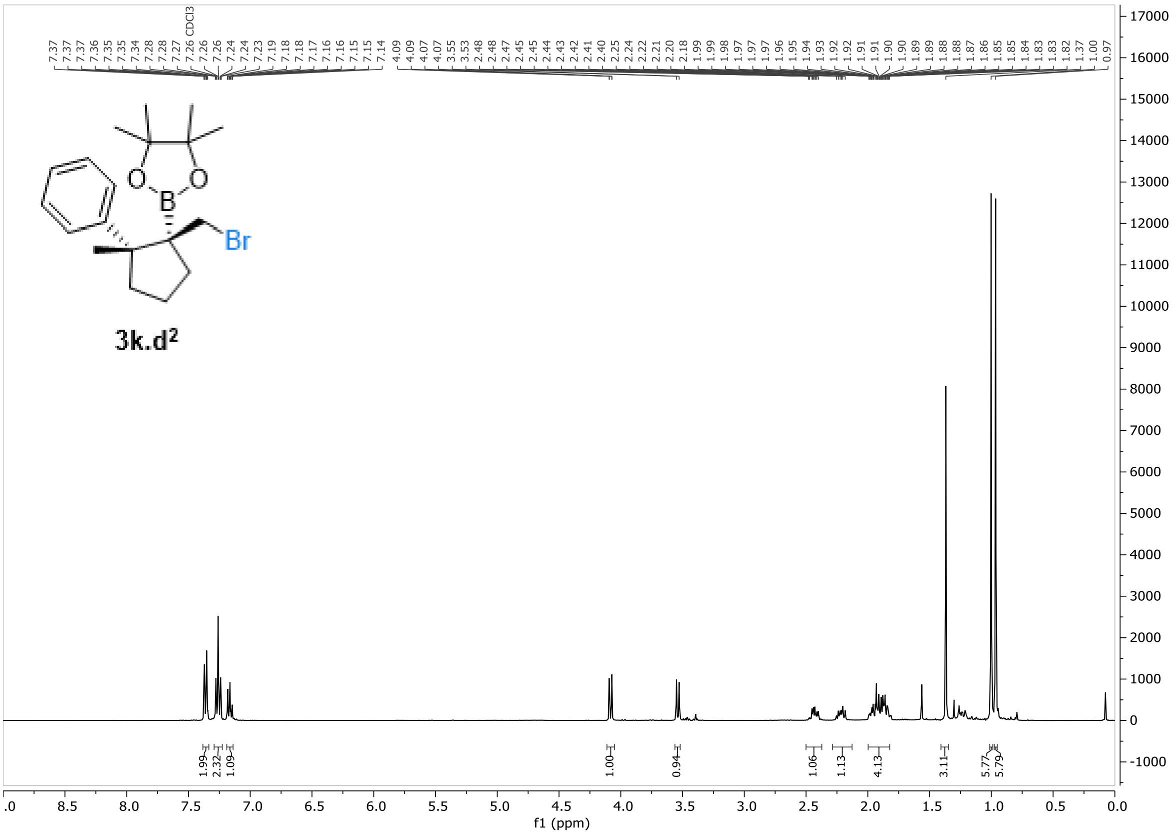

^{13}C NMR (101 MHz, $CDCl_3$) of **3k.d^2**

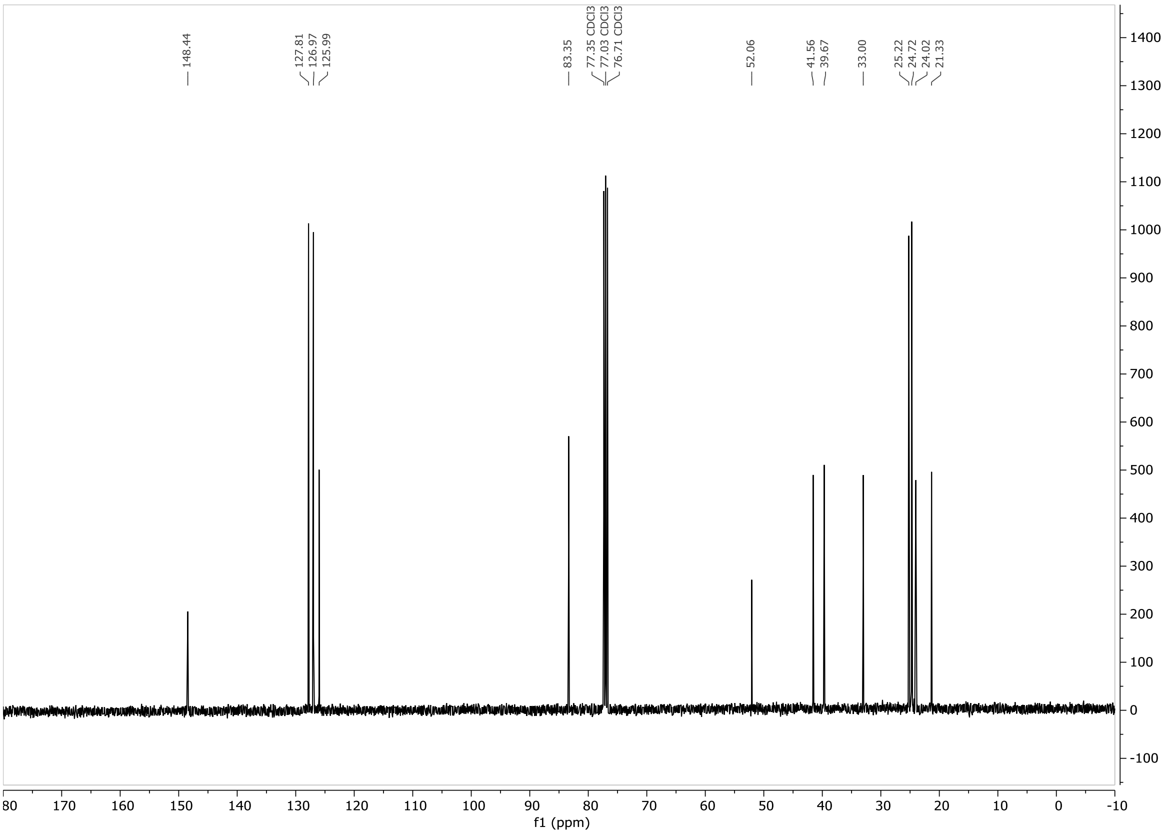

^{1}H NMR (400 MHz, $CDCl_3$) of **3l.d²** *(see procedure)*

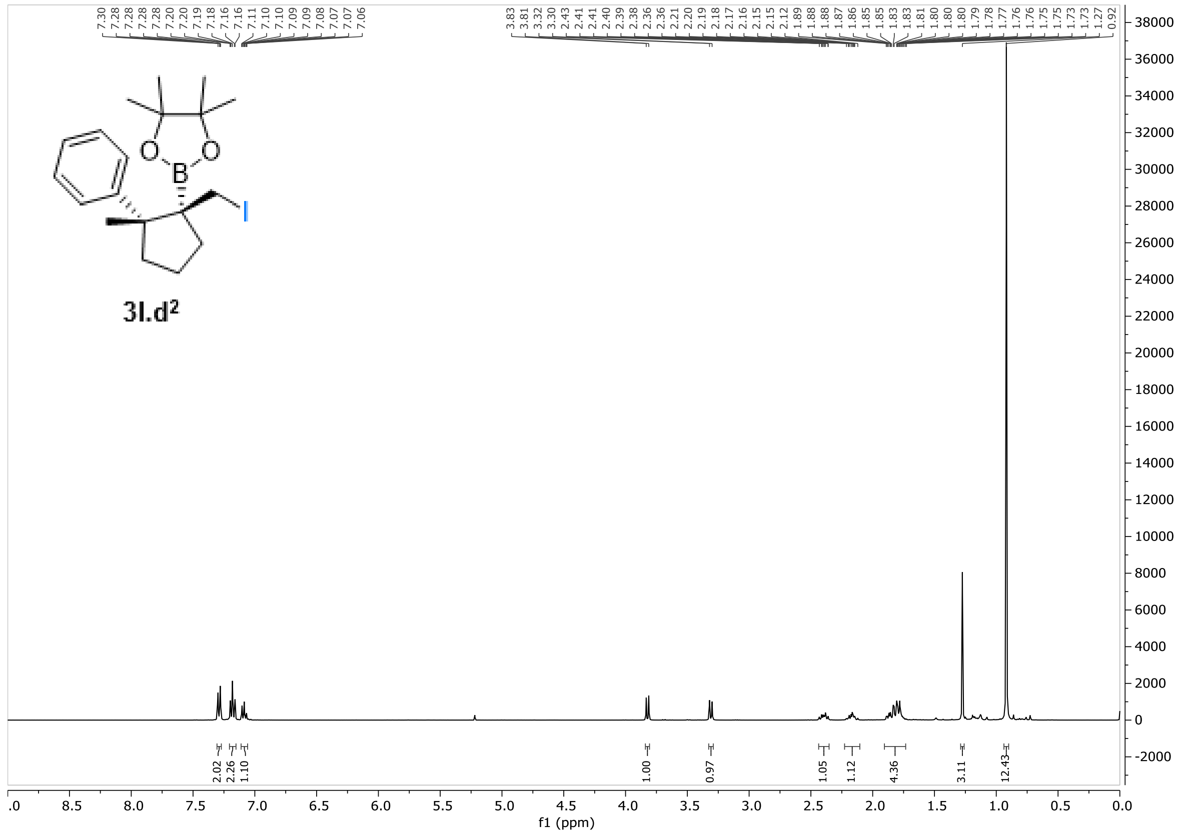

^{13}C NMR (101 MHz, $CDCl_3$) of **3l.d²**

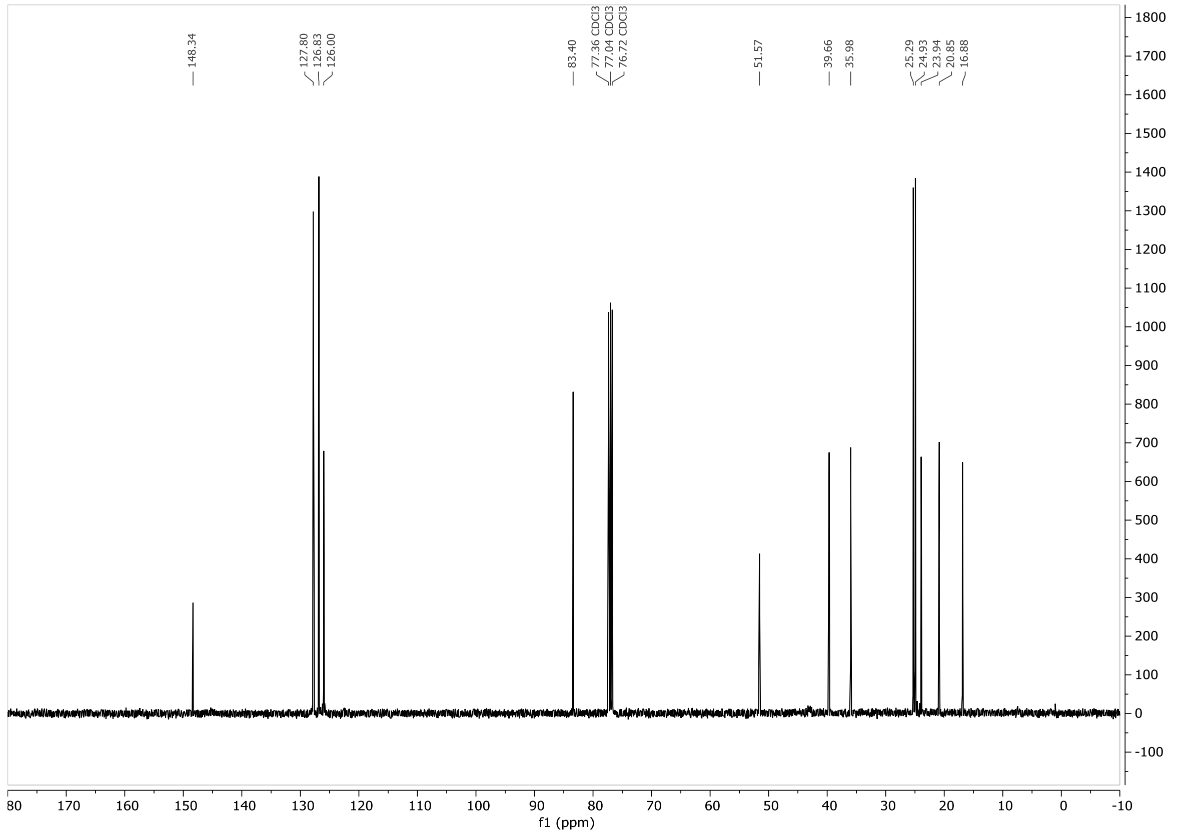

^1H NMR (400 MHz, $CDCl_3$) of **3m.d²** (see procedure)

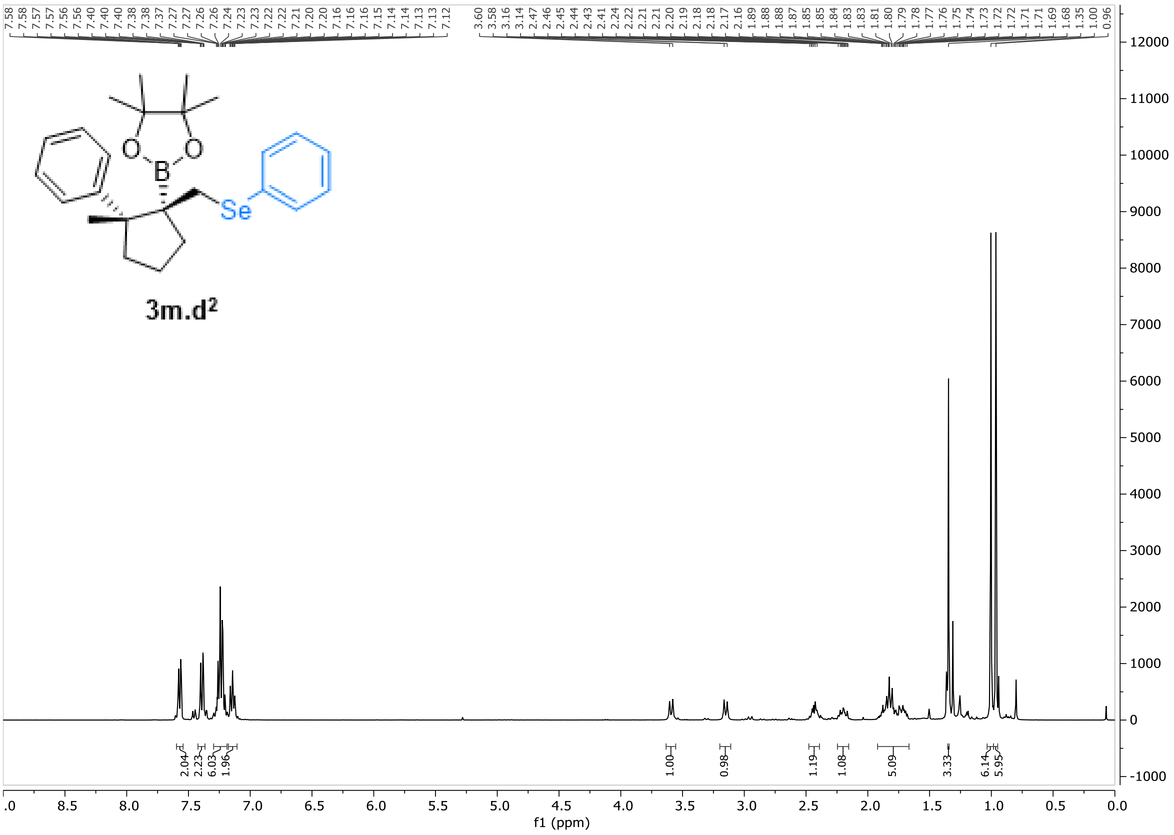

^{13}C NMR (101 MHz, $CDCl_3$) of **3m.d²**

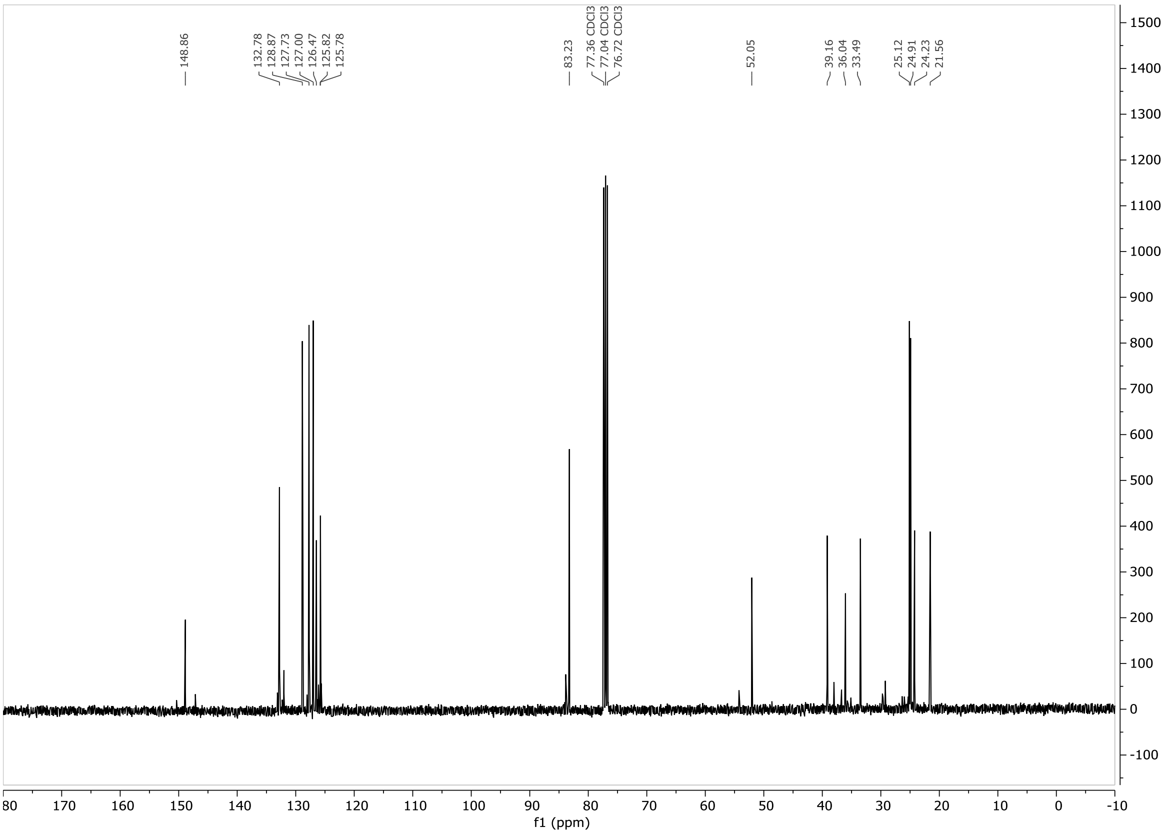

^{1}H NMR (400 MHz, $CDCl_3$) of **3n.d²** (*see procedure*)

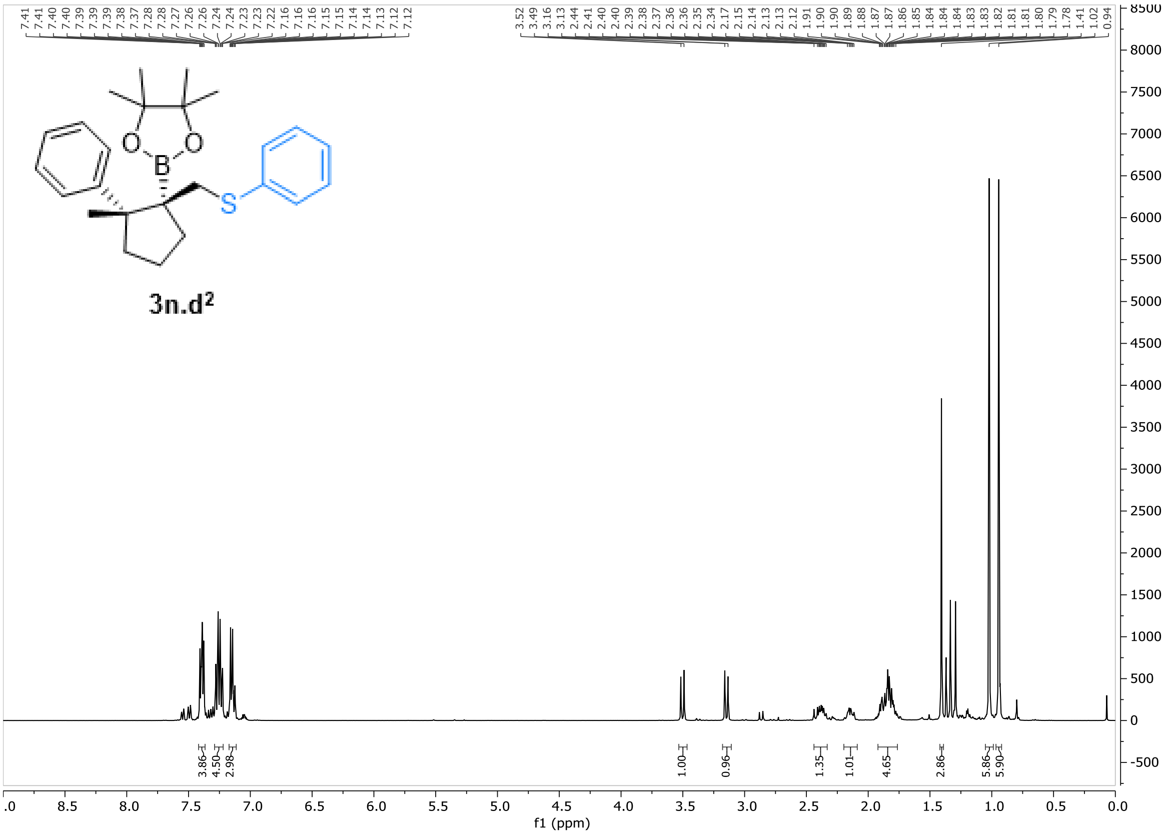

^{13}C NMR (101 MHz, $CDCl_3$) of **3n.d²**

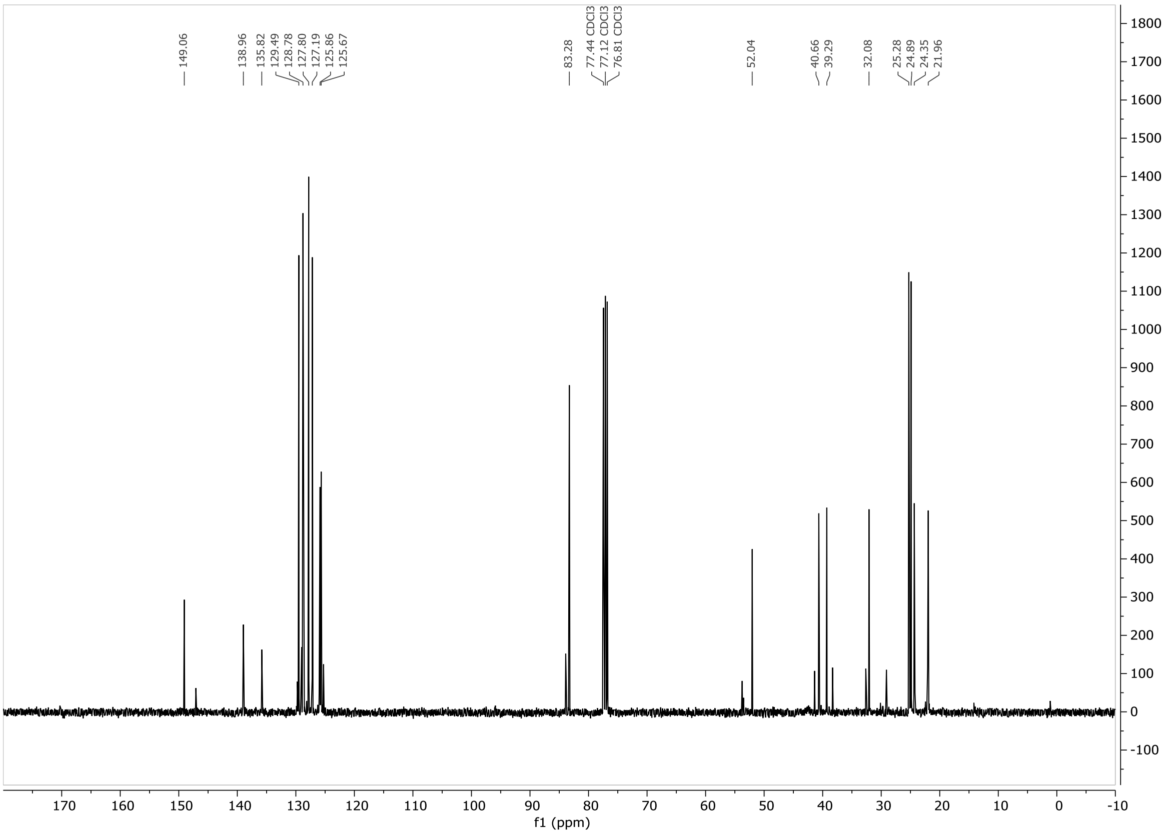

^{1}H NMR (400 MHz, $CDCl_3$) of **3o.d[2]** *(see procedure)*

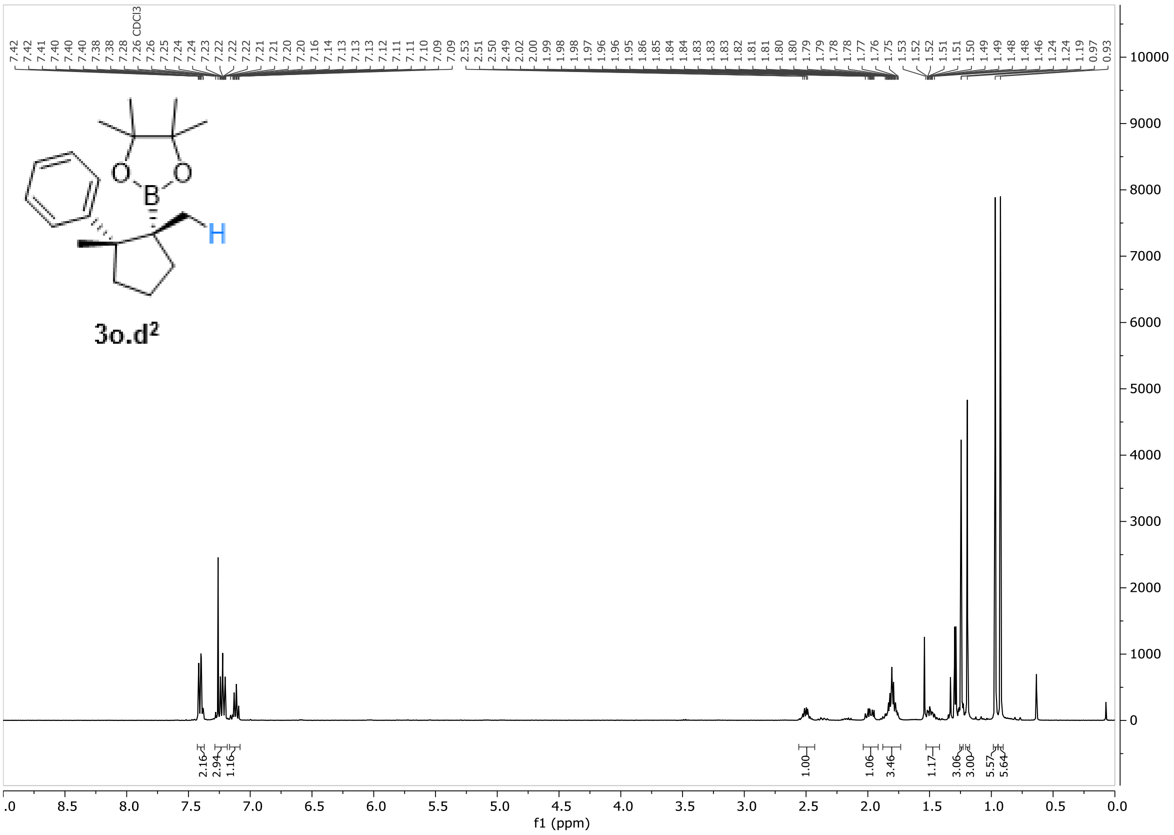

^{13}C NMR (101 MHz, $CDCl_3$) of **3o.d[2]**

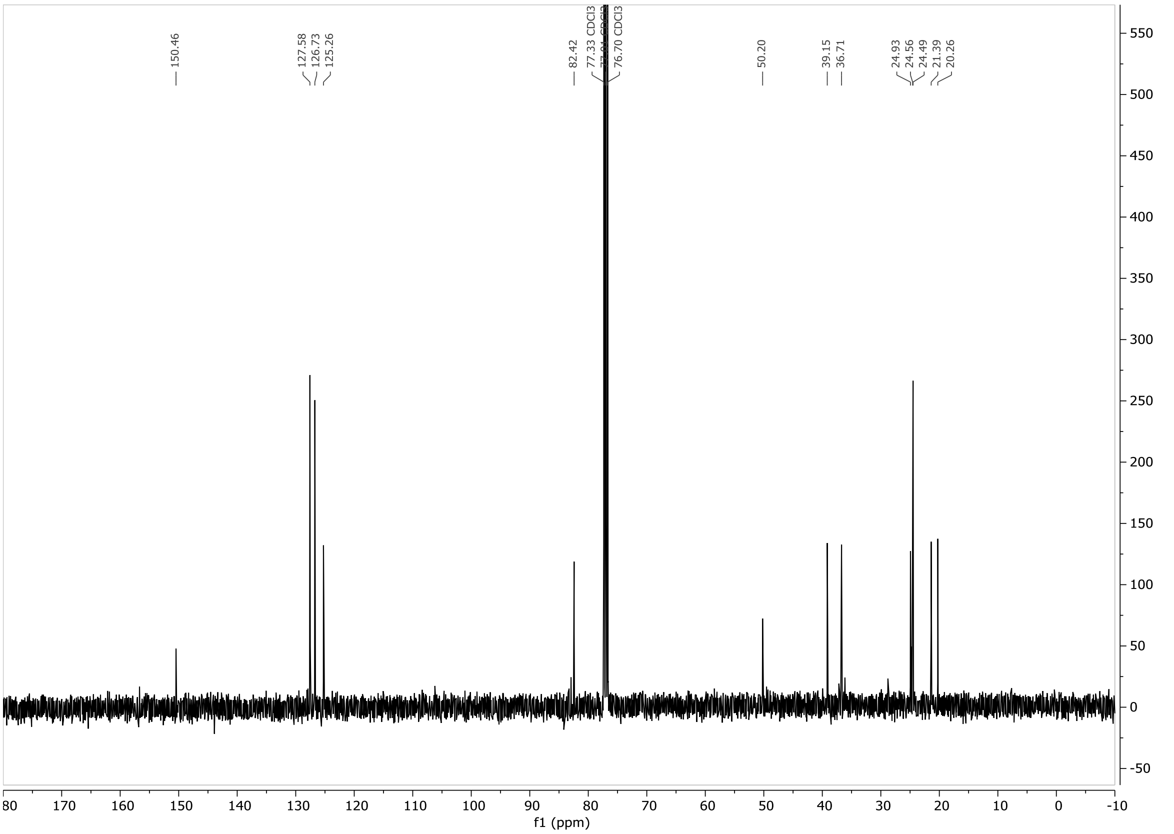

^{1}H NMR (400 MHz, $CDCl_3$) of **3o’.d^2** *(see procedure)*

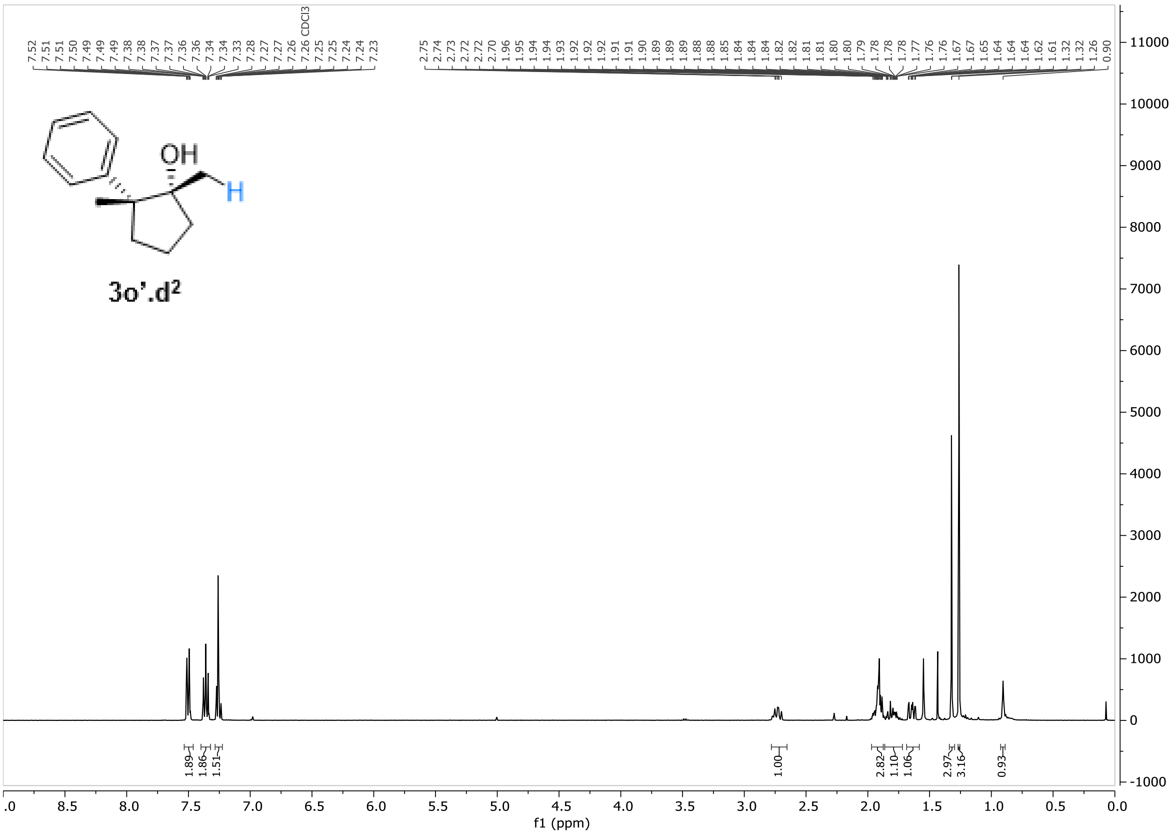

^{13}C NMR (101 MHz, $CDCl_3$) of **3o’.d^2**

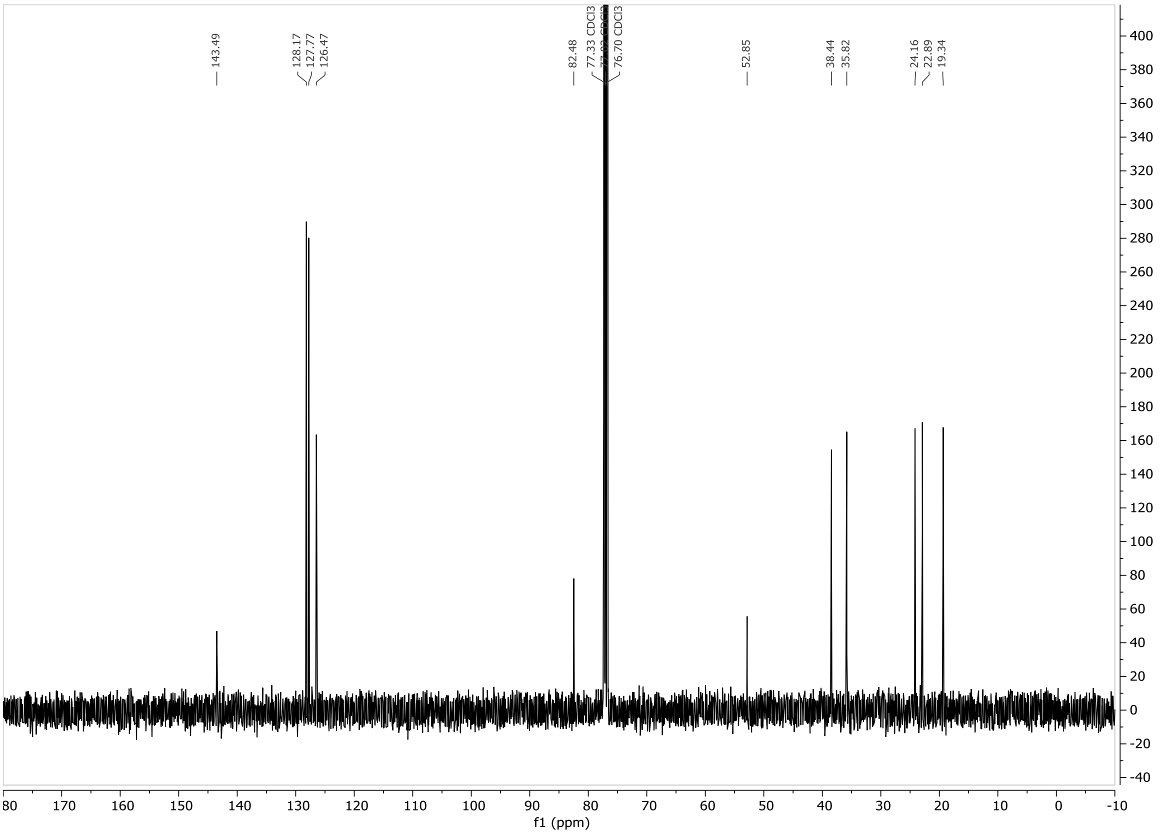

^{1}H NMR (400 MHz, $CDCl_3$) of **7** *(see procedure)*

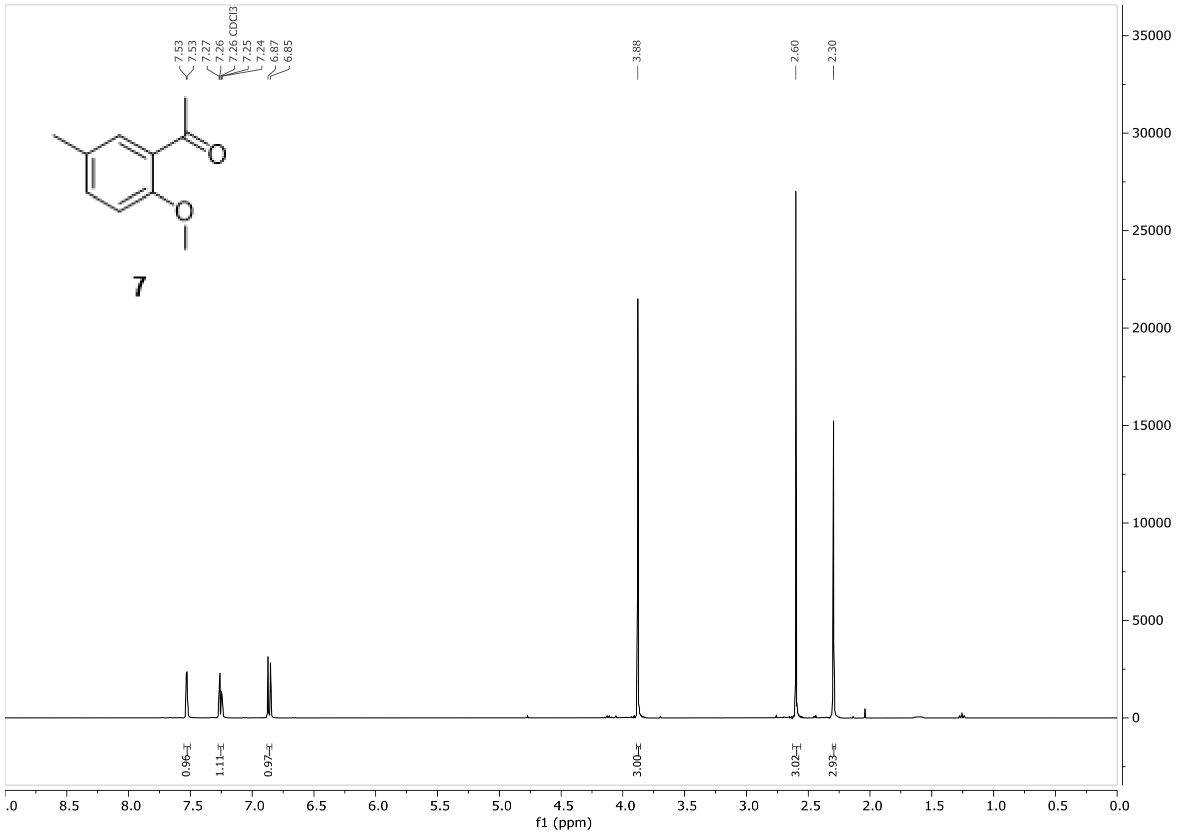

^{13}C NMR (101 MHz, $CDCl_3$) of **7**

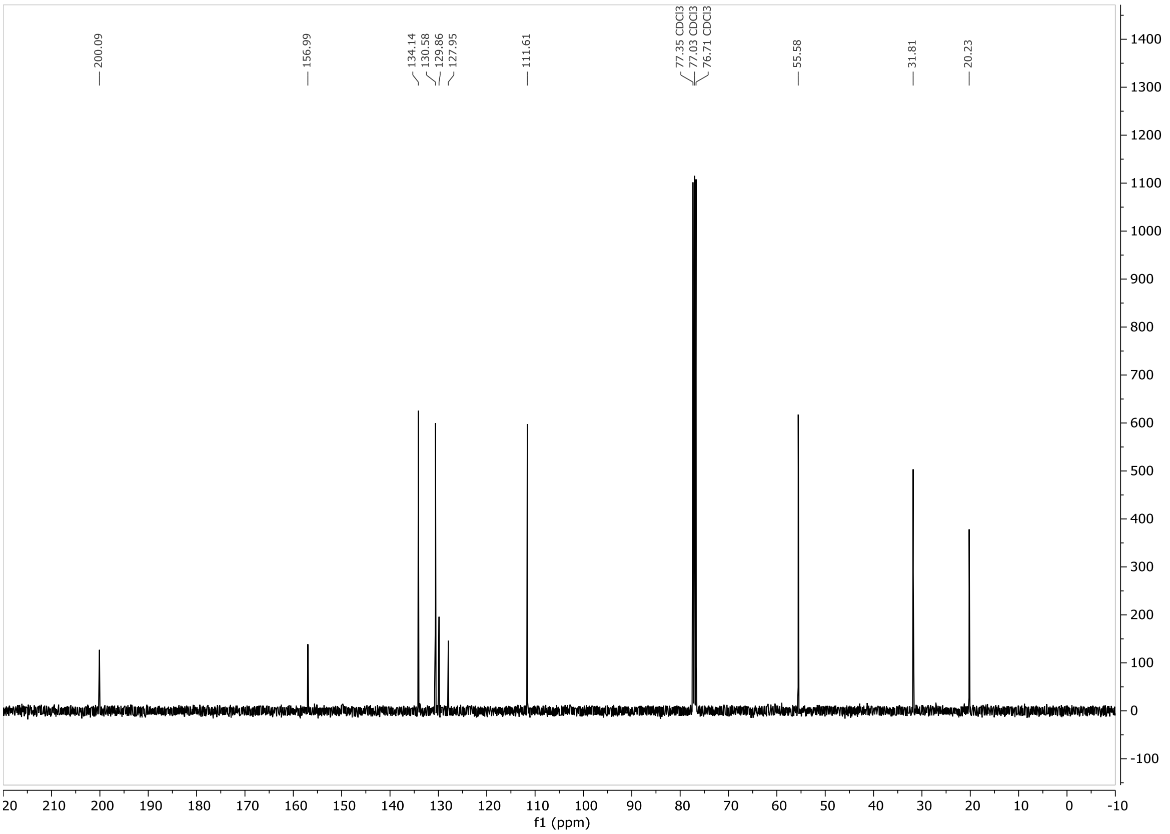

^{1}H NMR (400 MHz, $CDCl_3$) of **8** *(see procedure)*

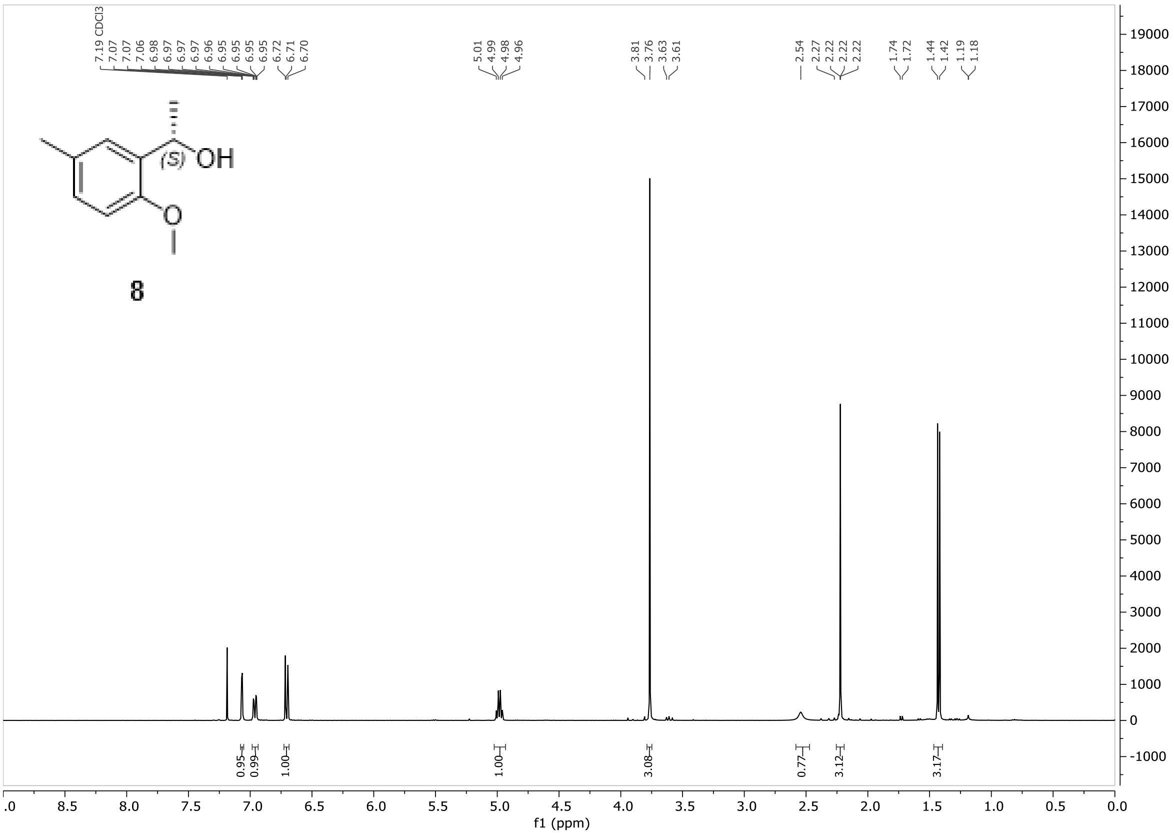

^{13}C NMR (101 MHz, $CDCl_3$) of **8**

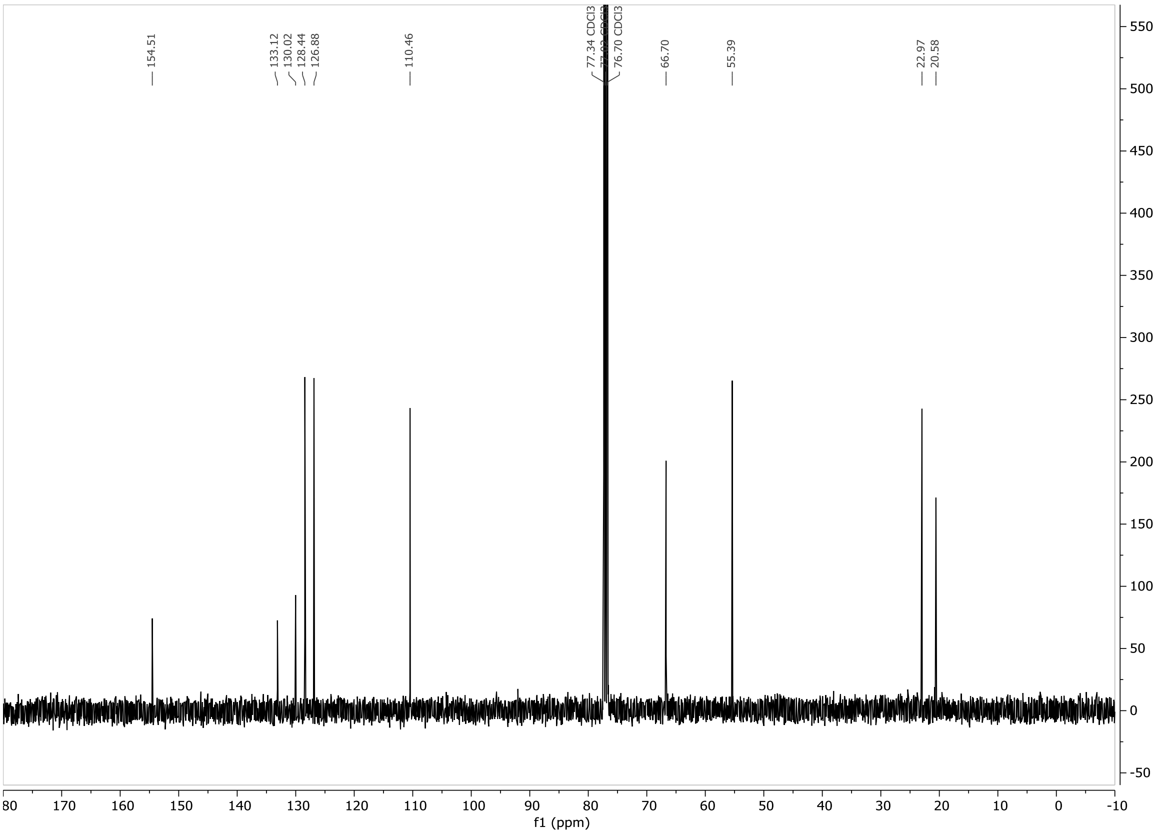

^{1}H NMR (400 MHz, $CDCl_3$) of **9** *(see procedure)*

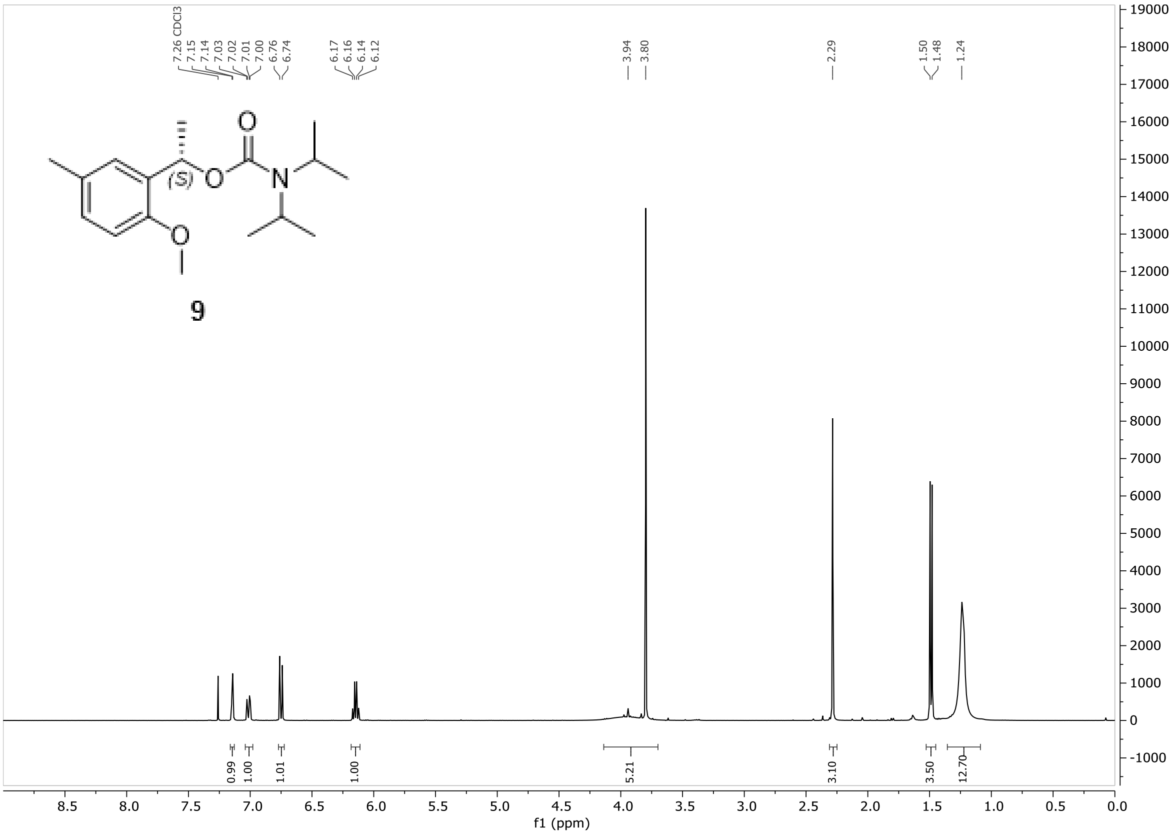

^{13}C NMR (101 MHz, $CDCl_3$) of **9**

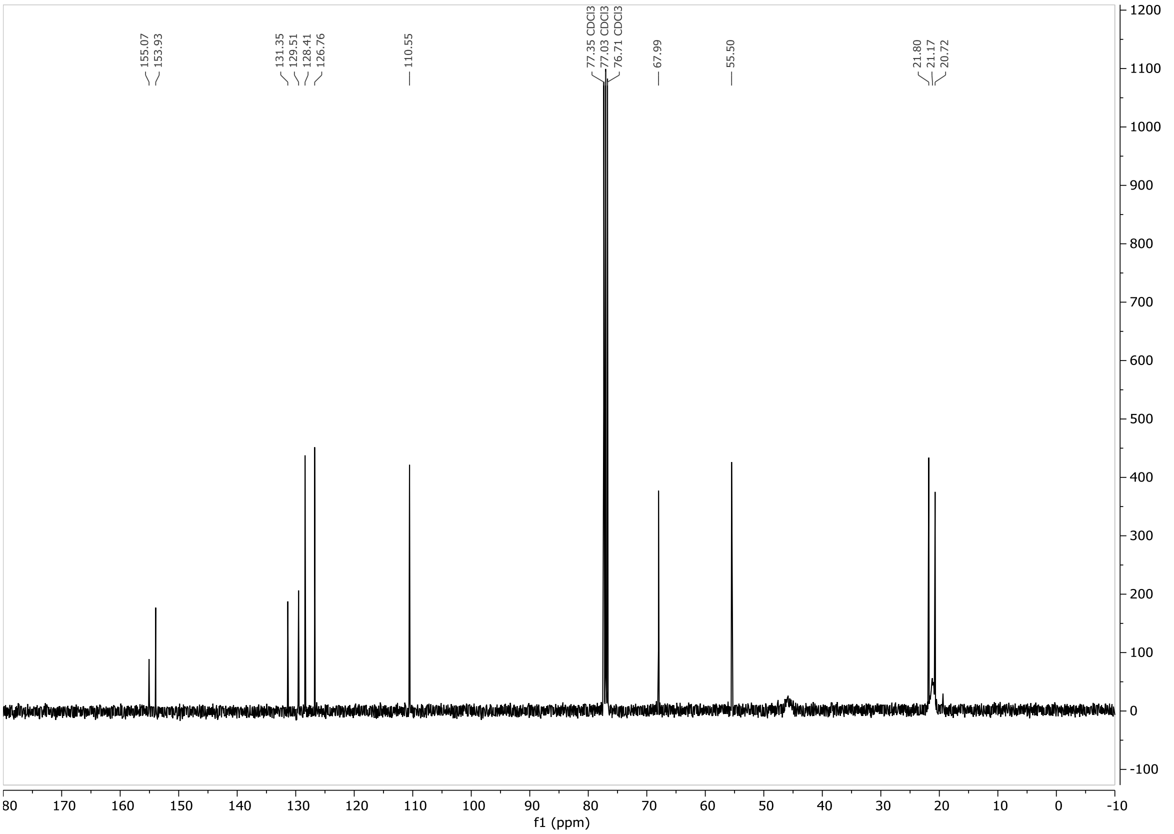

^{1}H NMR (400 MHz, $CDCl_3$) of **1i** *(see procedure)*

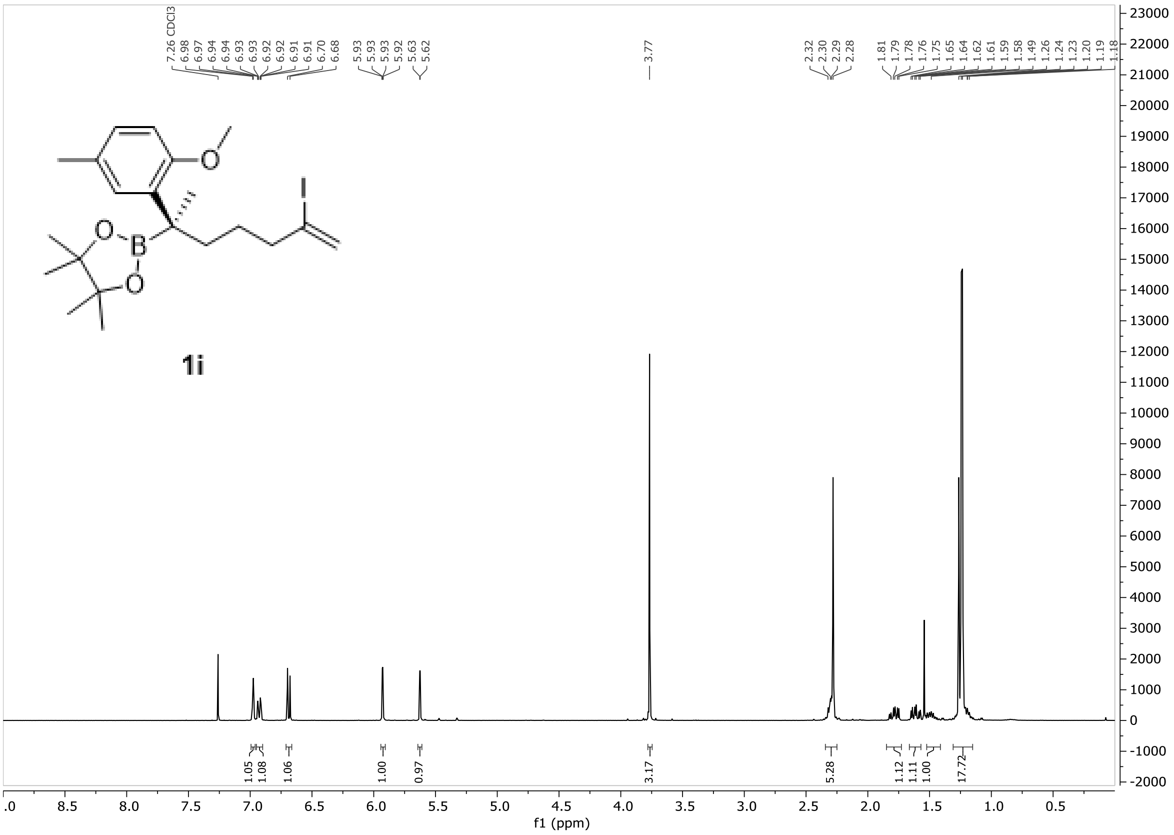

^{13}C NMR (101 MHz, $CDCl_3$) of **1i**

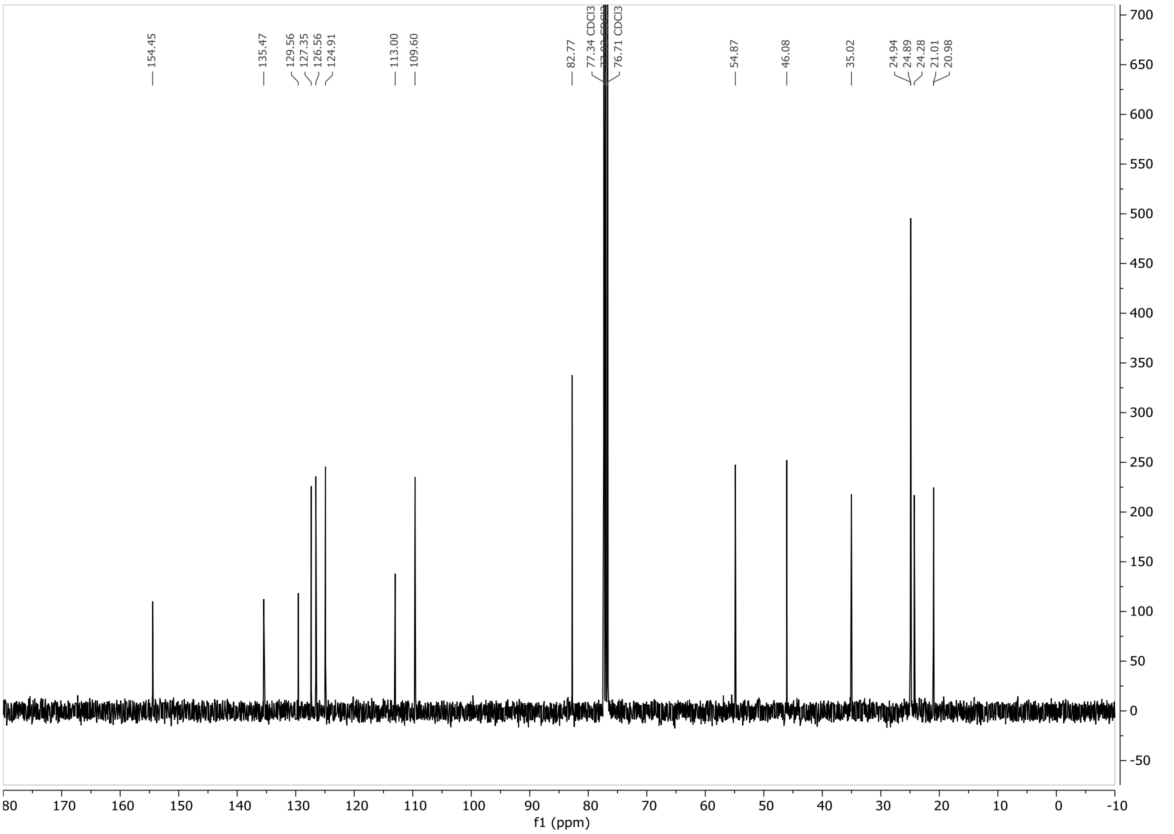

^{1}H NMR (400 MHz, $CDCl_3$) of **1i’** *(<u>see procedure</u>)*

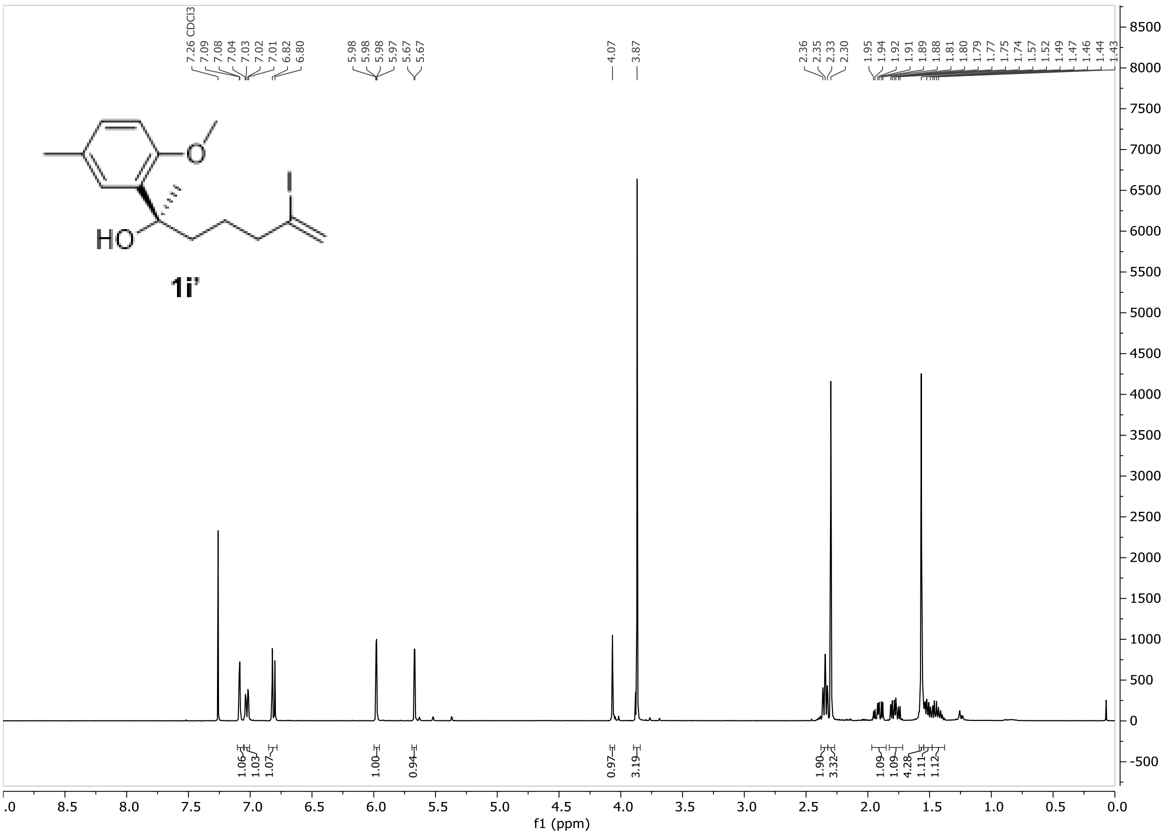

^{13}C NMR (101 MHz, $CDCl_3$) of **1i’**

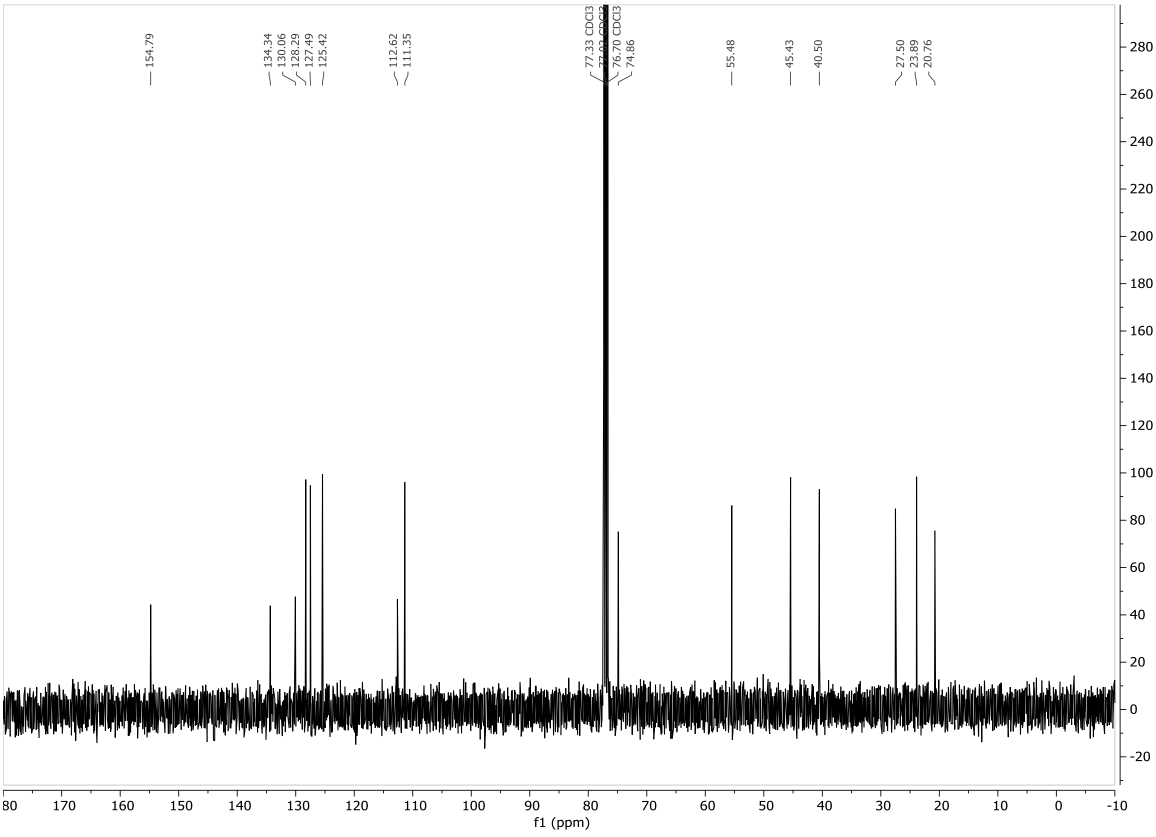

^{1}H NMR (500 MHz, $CDCl_3$) of **3p.d^2** (<u>*see procedure*</u>)

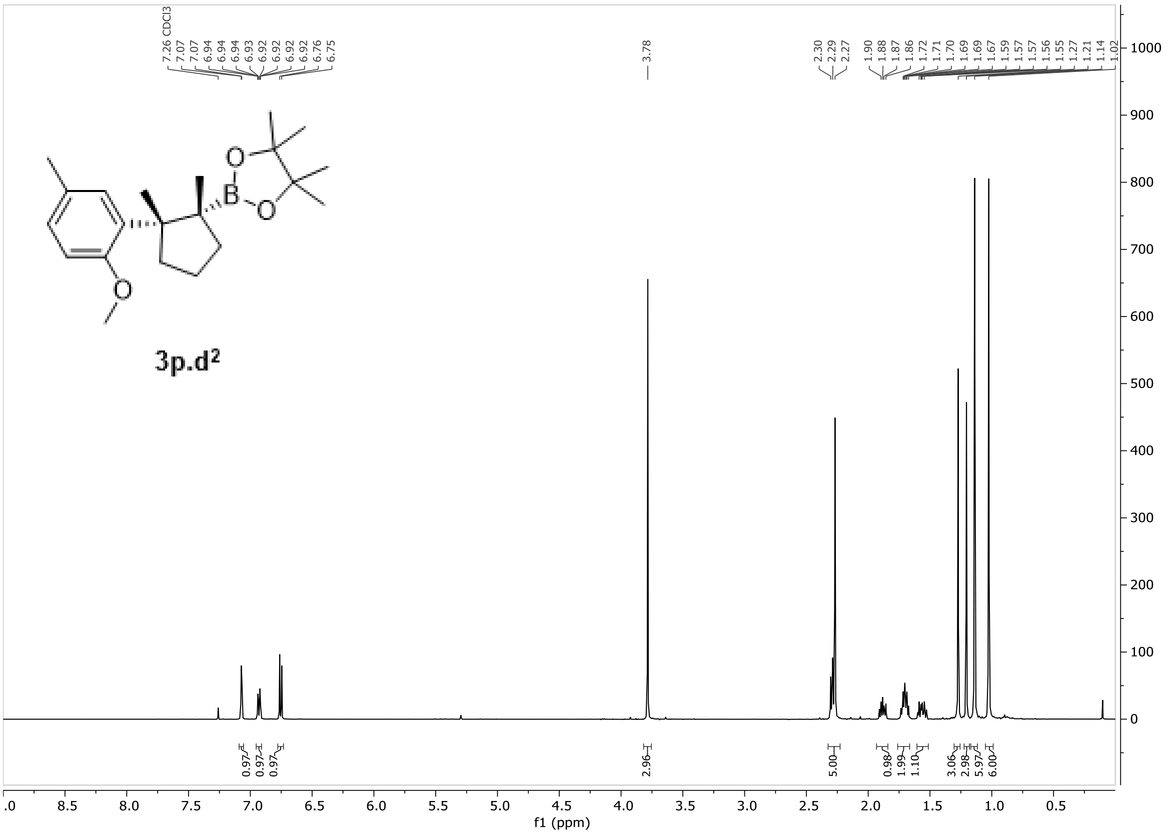

^{13}C NMR (126 MHz, $CDCl_3$) of **3p.d^2**

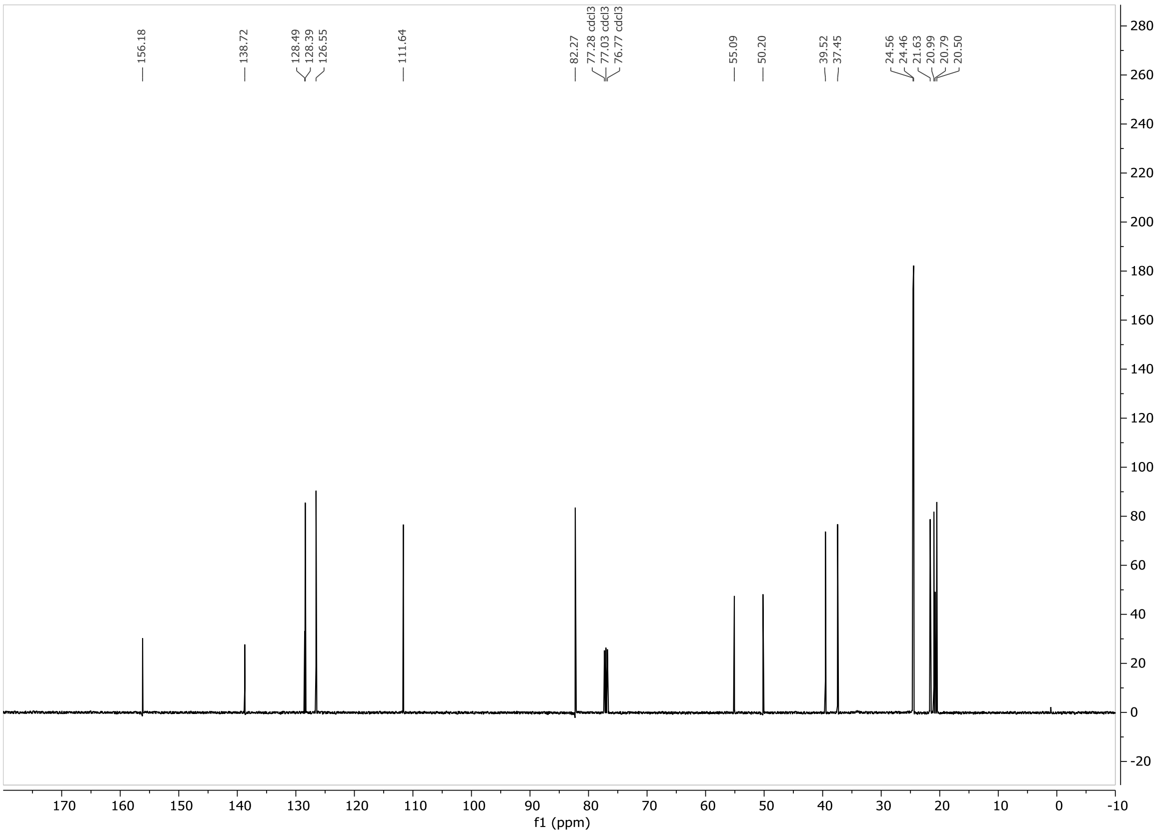

^{1}H NMR (500 MHz, CDCl$_3$) of **3p.d[1]** (<u>*see procedure*</u>)

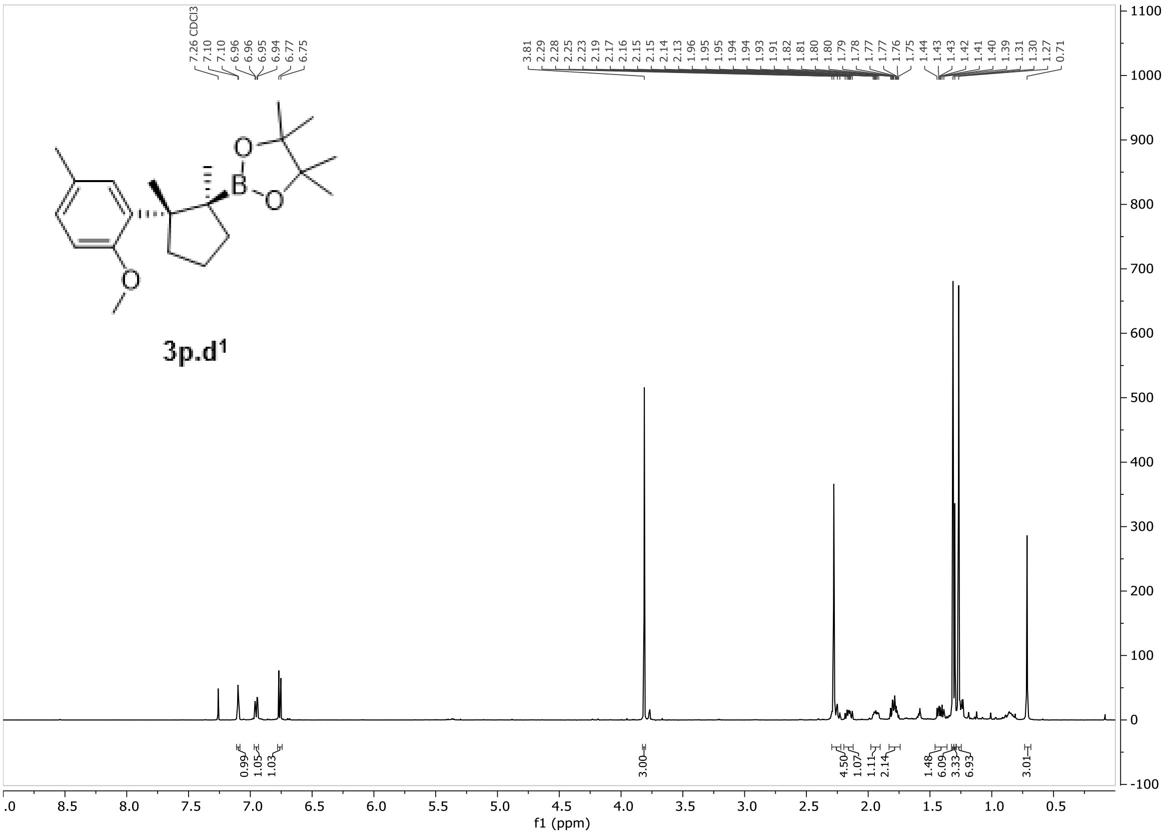

^{13}C NMR (126 MHz, CDCl$_3$) of **3p.d[1]**

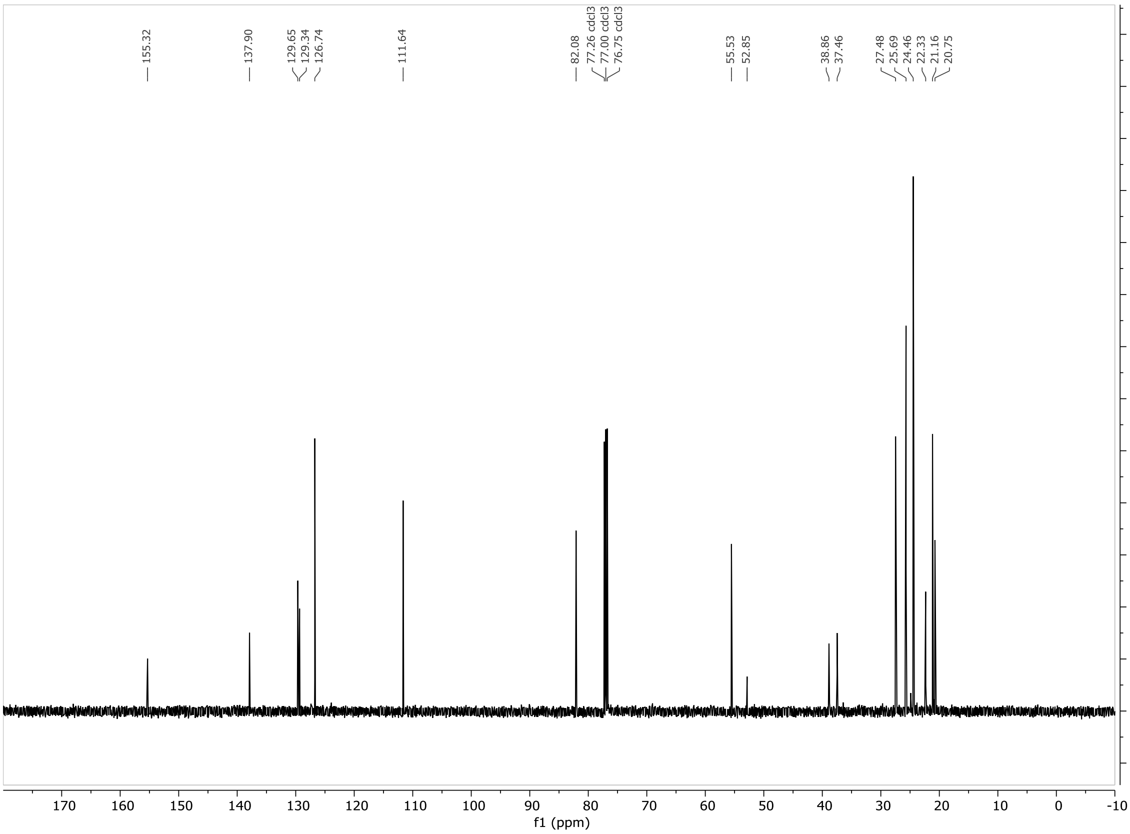

^{1}H NMR (500 MHz, $CDCl_3$) of **11** *(see procedure)*

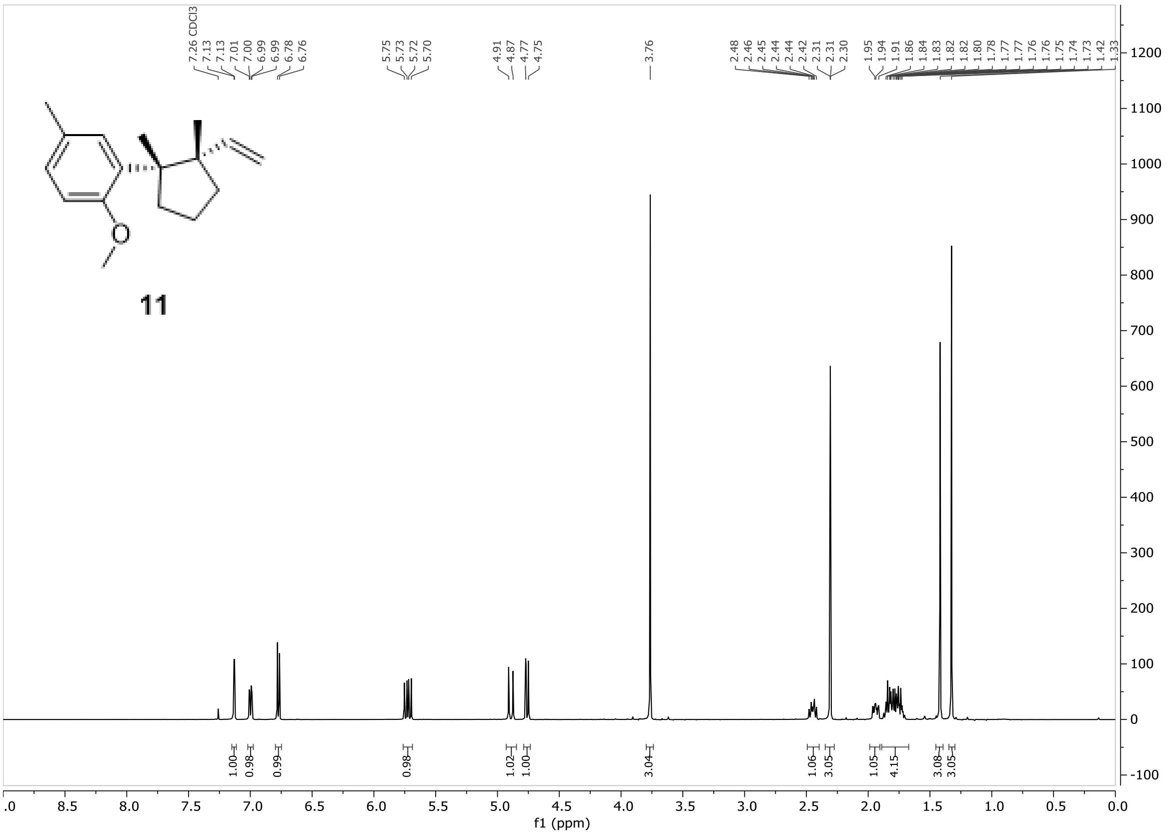

^{13}C NMR (126 MHz, $CDCl_3$) of **11**

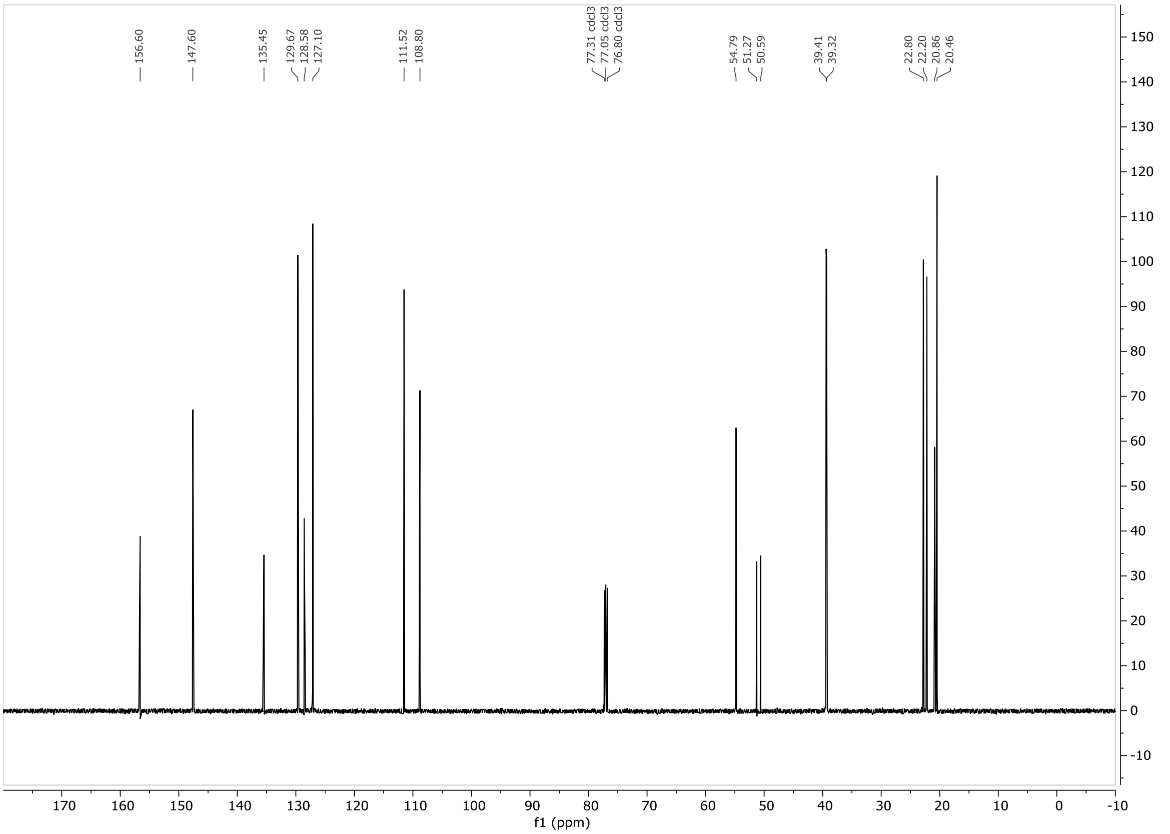

^{1}H NMR (400 MHz, $CDCl_3$) of **12** *(see procedure)*

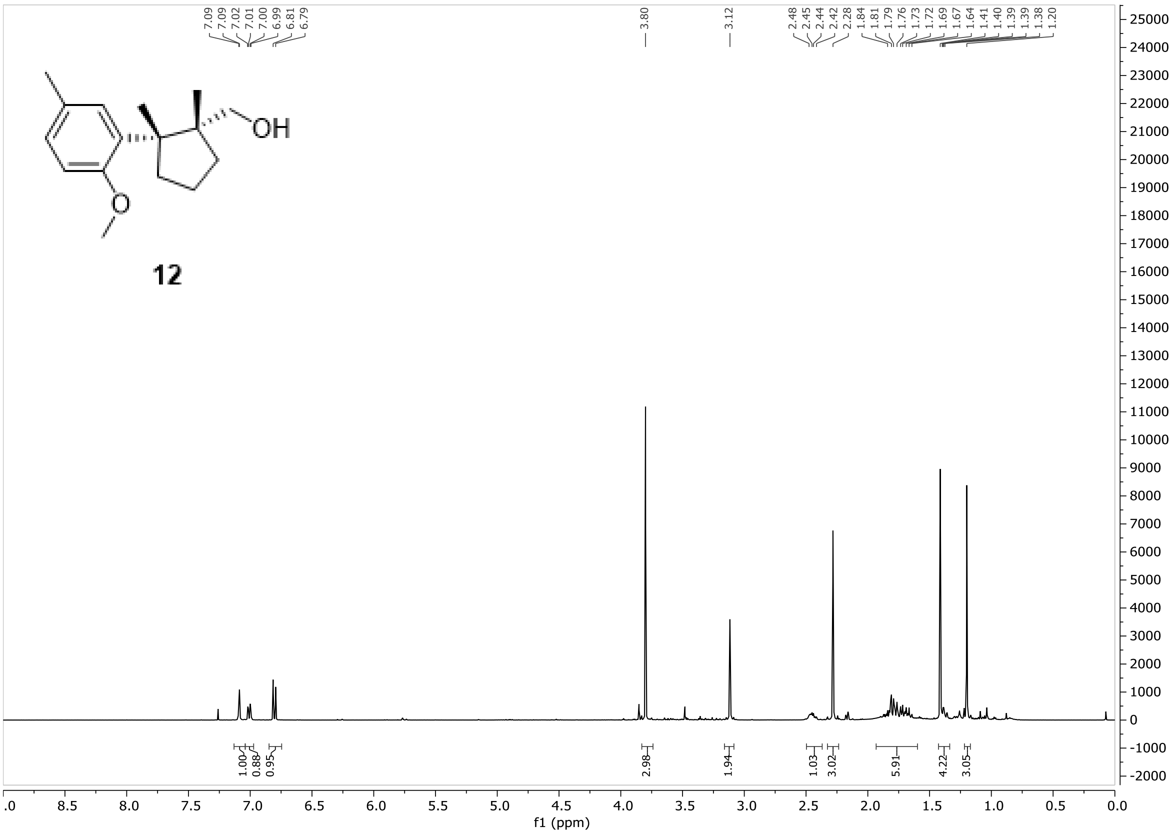

^{13}C NMR (101 MHz, $CDCl_3$) of **12**

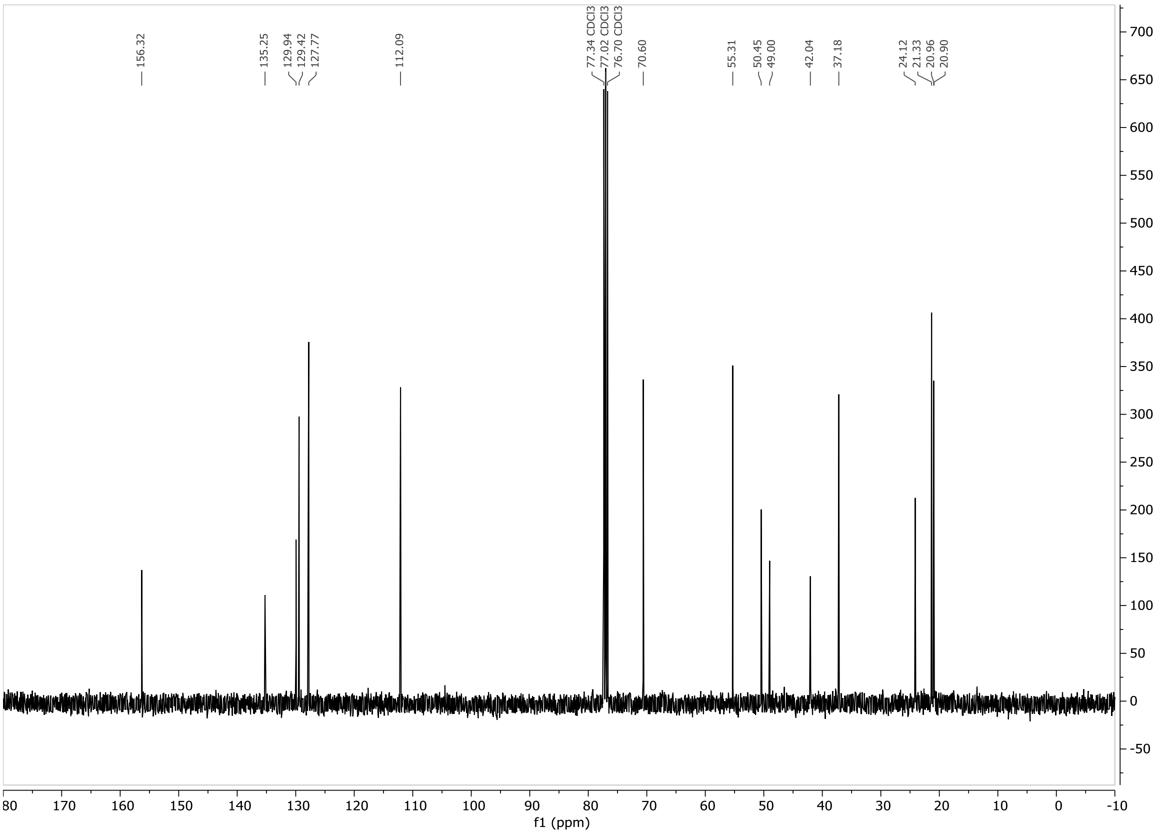

^{1}H NMR (500 MHz, $CDCl_3$) of **13** *(see procedure)*

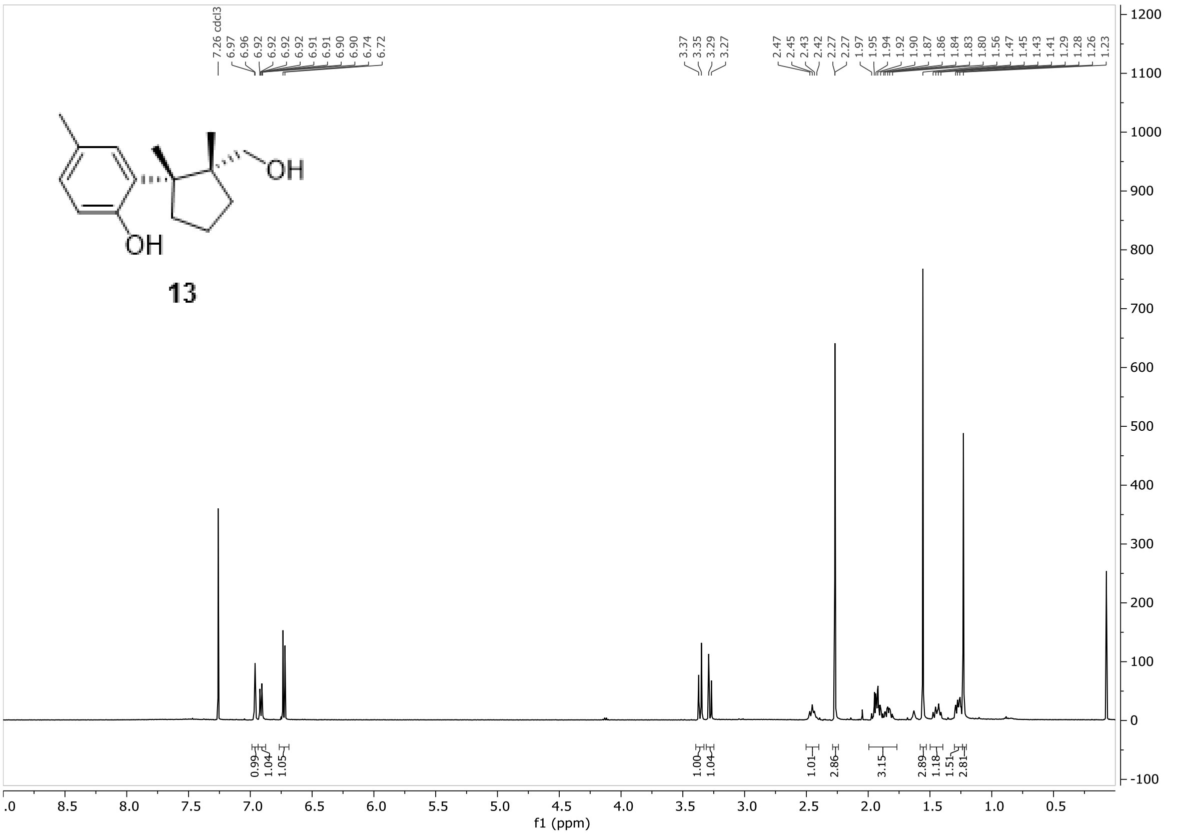

^{13}C NMR (125 MHz, $CDCl_3$) of **13**

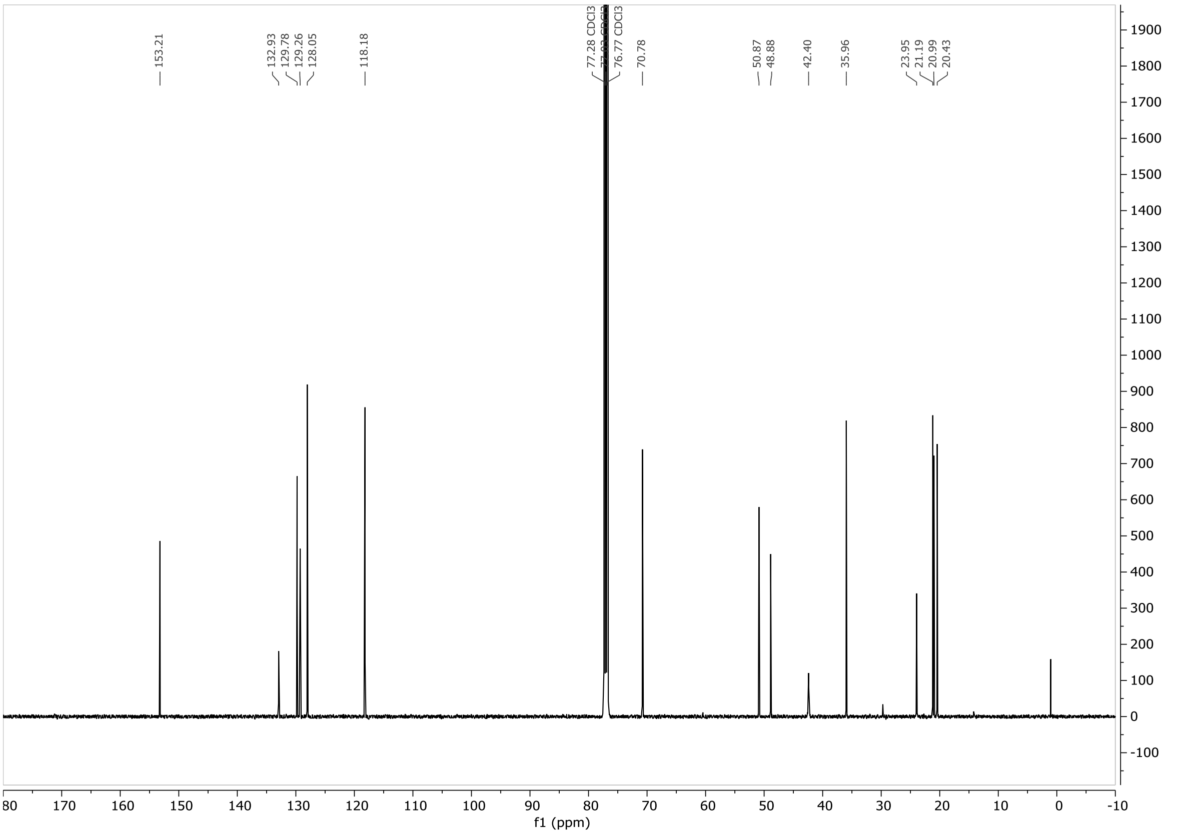

Supplement: Supplementary file 1 — Supporting Information [file ANIE-61-0-s001.pdf]
